# Gut microbiome community profiling of Bornean bats with different feeding guilds

**DOI:** 10.1186/s42523-025-00389-w

**Published:** 2025-03-05

**Authors:** Muhd Amsyari Morni, Julius William-Dee, Emy Ritta Jinggong, Nor Al-Shuhada Sabaruddin, Nur Afiqah Aqilah Azhar, Muhammad Amin Iman, Peter A. Larsen, Jaya Seelan Sathiya Seelan, Lesley Maurice Bilung, Faisal Ali Anwarali Khan

**Affiliations:** 1 Faculty of Resource Science and Technology, Universiti Malaysia Sarawak, Sarawak, 94300 Kota Samarahan Malaysia; 2 Institute of Biodiversity and Environmental Conservation, Universiti Malaysia Sarawak, Sarawak, 94300 Kota Samarahan Malaysia; 3 Department of Veterinary and Biomedical Sciences, University of Minnesota, Saint Paul, MN 55108 United States of America; 4 Institute for Tropical Biology and Conservation, Universiti Malaysia Sabah, Sabah, 88400 Kota Kinabalu Malaysia

**Keywords:** Bats, Bacteria, Borneo, Gut microflora, Insectivorous, Nanopore sequencing, Phytophagous

## Abstract

Bats are extraordinary mammals. They have evolved to consume various dietary sources, such as insects, fruits, nectar, blood, and meat. This diversity has generated considerable interest in the scientific community, resulting in efforts to leverage bats as model organisms to study the correlation between diet and gut microbiome community. Although such studies now commonly use Next Generation Sequencing (NGS), similar studies are early in their development in Southeast Asia, especially in Malaysia, which harbours an incredibly diverse bat fauna. This study provides pioneering NGS metabarcoding information on Bornean bats. By using a high-throughput Nanopore-based 16S rRNA gene sequencing method, Bacillota, Pseudomonadota, and Campylobacterota were found in insectivorous bats and phytophagous bats. Both insectivorous and phytophagous groups harboured no dominant taxon (D = 0.076; D = 0.085). A comparative analysis of gut bacteria functional groups identified eight major groups in both phytophagous and insectivorous bats, with fermentation being the predominant group. The correlation network analysis revealed a negative correlation between the ‘good bacteria’ *Lactobacillus* and various pathogenic bacteria genera, such as *Salmonella* (-0.4124) and *Yersinia* (-0.4654), demonstrating its prebiotic characteristics. This study broadens our understanding of the bat gut microbiome from various diets, with emphasis on new data from Borneo.

**Supplementary Information:**

The online version contains supplementary material available at 10.1186/s42523-025-00389-w.

## Background

The gut microbiome plays crucial roles across many facets of mammalian health. The gut microbiome comprises billions of microorganisms and is one of the most complicated microbial ecosystems [1–3]. This microbial community plays a crucial role in synergizing with the host to enhance energy storage, maintain functional stability, and promote metabolic balance within the gastrointestinal tract [4, 5]. Some examples of bacterial phylums reported in mammals are Bacteroidota, Campylobacterota, Bacillota, and Pseudomonadota [6–8]. The diversity of gut bacteria is important not only for the host’s health but also for the gut microbiota’s products [9, 10]. This includes proteins, small molecular chemicals, and even DNA [11, 12]. The diverse microbiome is substantially influenced by dietary preference [4, 13–16].

Bats are unique in terms of their dietary preferences. Their evolutionary diversity is fascinating, ranging from an insectivorous diet as their ancestral character to a wide range of diets, including blood, meat, nectar, fruit, and diverse omnivorous mixtures [17]. Bats are ranked as the second-most specious group of mammals after the order Rodentia [18]. They are volant, can disperse over long distances, and display a variety of life history traits, ranging from different feeding guilds and roosting practices to unique social behaviours and reproductive strategies. There are more than 1469 species of bats, making up one-fifth of the mammals in the world [19]. Due to their diverse feeding strategies, bats are ideal wild animal models to study the relationship between diets and gut microbiomes [20].

Previous research on bat microbiomes revealed that there is a lot of variety in the microbiomes of both insect-eating (insectivorous) and plant-eating (phytophagous, i.e., both frugivorous and nectarivorous) bats [20–22]. Microbiome composition and diversity are influenced by a variety of endogenous and exogenous variables, including geographical origin, age, genetics, food, and the use of prebiotics and antibiotics [3]. The intestinal bacterial community of insectivorous bats in central-southern Mexico was noted to harbour greater diversity as compared to phytophagous bats from the same area [22]. Phytophagous bats in China had a higher microbial diversity than insectivorous species [20]. A study of guano deposits in the Philippines using metagenomics found that they were mostly made up of Pseudomonadota (61.7%), Actinomycetota (19.4%), Bacteroidota(4.2%), Bacillota (2.7%), Chloroflexota (2.5%), candidate phylum TM7 (2.3%), and Planctomycetota (1.9%) [23].

Recently, high-throughput sequencing methods have become more widely available and cost-effective. This enables researchers to conduct more microbiota studies aimed at demonstrating the connection between a bat’s host and its microbiota. This is especially helpful as studies on the microbiome composition of different bat diet groups with its functional groups are lacking in Southeast Asia, especially Borneo. In this study, the 16 S rRNA gene was sequenced using next-generation sequencing (NGS) to characterise the gut bacteria of healthy phytophagous and insectivorous bats. The aim of this study was to describe the baseline gut microbiome community patterns and functional groups in insectivorous and phytophagous bats.

## Materials and methods

### Faecal sample collection

**Fig. 1:**
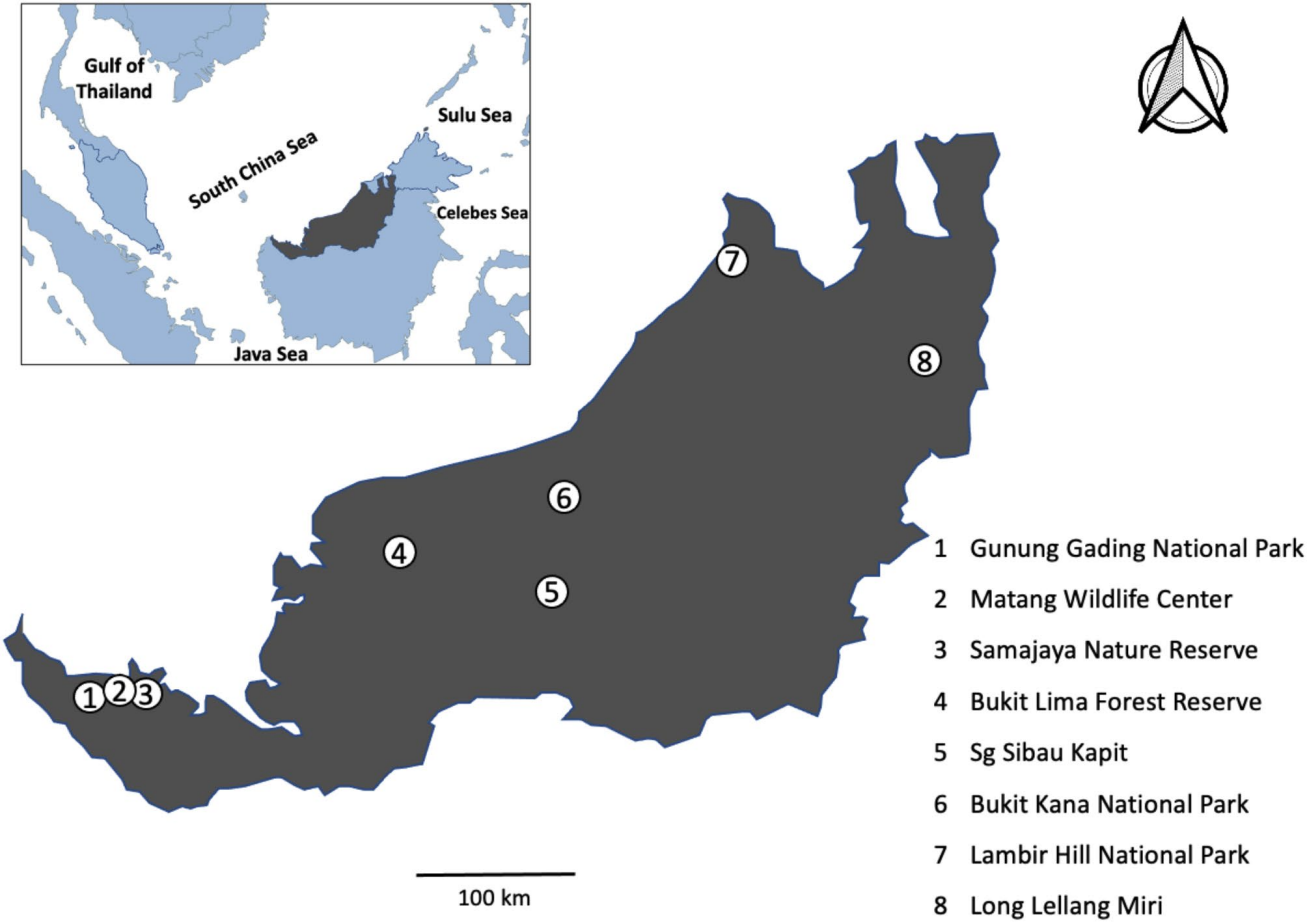
Inset map highlighted in grey shows the Malaysian Borneo boundary. Larger map of Malaysian Borneo on the right shows the collection sites of bats fecal sample, labelled from 1 until 8

Thirty healthy adult bats with different diets were randomly collected using harp traps and mist nets from eight localities throughout Sarawak, Malaysian Borneo (Fig. 1). The sampling was conducted in 2022 and 2023, primarily during the dry season to maximise the number of sampling nights. Each sites was sampled for 5 nights. The trapped bats were separated into two groups based on diet, as indicated in Table 1: 17 for the phytophagous (plant-based diet: frugivorous and nectarivores) group and 13 for the insectivorous (insect-based diet) group. The bats were housed in individual sterilised sacks. Upon defecation, fresh faecal samples were collected using sterilised forceps and transferred into a sterilised faecal tube containing 300 ul of RNAprotect solution (QIAGEN, Germany) for nucleic acid preservation. The workbench was sterilised using a 30% bleach solution between individual bats. The faecal samples were then transferred into a -80 °C deep freezer in the laboratory until the DNA extraction procedure. After collecting faecal samples, the bats were released.

**Table 1:** Details on the Bat samples used in this study

Species/Sample ID	(*n*)	Diet	GenBank BioSample accession number
*Glischropus tylophus*	3	Insectivorous	
GtyloGGNP21005			SAMN40240913
GtyloGGNP21029			SAMN40240914
GtyloLL22016			SAMN40240915
*Hipposideros diadema*	2	Insectivorous	
HdiadBKNP20055			SAMN40240916
HdiadBKNP20090			SAMN40240917
*Kerivoula hardwickii*	1	Insectivorous	
KhardGGNP21015			SAMN40240918
*Kerivoula minuta*	1	Insectivorous	
KminuLHNP22009			SAMN40240919
*Myotis muricola*	1	Insectivorous	
MmuriLL22001			SAMN40240920
*Myotis ridleyi*	1	Insectivorous	
MridlGGNP21001			SAMN40240921
*Rhinolophus acuminatus*	1	Insectivorous	
RacumBKNP20085			SAMN40240922
*Rhinolophus affinis*	1	Insectivorous	
RaffiMWC21024			SAMN40240923
*Rhinolophus philippinensis*	1	Insectivorous	
RphilBKNP20089			SAMN40240924
*Rhinolophus sedulus*	2	Insectivorous	
RseduBKNP20015			SAMN40240925
RseduLHNP22008			SAMN40240926
*Rhinolophus trifoliatus*	1	Insectivorous	
RtrifBKNP20081			SAMN40240927
*Tylonycteris pachypus*	1	Insectivorous	
TpachGGNP21007			SAMN40240928
*Tylonycteris robustula*	1	Insectivorous	
TrobuSSK22024			SAMN40240929
*Balionycteris maculata*	4	Phytophagous	
BmacuBKNP20075			SAMN40240930
BmacuBKNP22031			SAMN40240931
BmacuBKNP22033			SAMN40240932
BmacuMWC21015			SAMN40240933
*Cynopterus brachyotis*	3	Phytophagous	
CbracBKNP20036			SAMN40240934
CbracBLFR22001			SAMN40240935
CbracSSK22005			SAMN40240936
*Dyacopteurs spadiceous*	1	Phytophagous	
DspadLHNP22010			SAMN40240937
*Macroglossus minimus*	5	Phytophagous	
MminiBKNP20052			SAMN40240938
MminiBKNP20077			SAMN40240939
MminiBKNP20094			SAMN40240940
MminiBKNP20095			SAMN40240941
MminiSSK22003			SAMN40240942

### DNA extraction, PCR amplification, and sequencing

The total genomic DNA for each faecal sample was extracted using the QIAmp PowerFecal Pro DNA (QIAGEN, Germany). The sequencing was performed using the MinION Mk1C sequencing platform (Oxford Nanopore Technology, United Kingdom). DNA was amplified using specific barcoded 16S rRNA primers (27F 5’-AGAGTTTGATCCTGGCTCAG-3’ and 1492R 5’-GGTTACCTTGTTACGACTT-3’) by Polymerase Chain Reaction (PCR) with the following cycling conditions: Initial denaturation 1 min @ 95 °C (1 cycle), denaturation 20 s @ 95 °C (25 cycles), annealing 30 s @ 55 °C (25 cycles), extension 2 min @ 65 °C(25 cycles), final extension 5 min @ 65 °C (1 cycle) and hold @ 4 °C. The amplified barcoded 16 S rRNA amplicons were approximately 1500 bp in length. All barcoded libraries were pooled to a total of 100 fmoles in 10 ul mM Tris-HCl pH 8.0 with 50 mM NaCl. The DNA library was then mixed with Sequencing Buffer (SQB), Loading Beads (LB), reagent RAP and Nuclease-free water before being loaded into SpotON sample port of the flowcell to begin sequencing. The detailed protocol can be referred in 16 S Barcoding Kit (SQK-RAB204) handbook (Oxford Nanopore Technology, United Kingdom). The entire process was conducted under sterile conditions to prevent any potential contamination.

### Bioinformatic processing

The resulting FASTQ data was imported into the EPI2ME programme and processed using the FASTQ 16 S procedure. Oxford Nanopore Technology (ONT) offers a cloud-based analytical platform. The readings were filtered using the default settings. Reads with a quality score below the threshold (value < 8) were not used in downstream analyses. 30% was the minimum coverage required. The minimum BLAST p_indent was set at 77%, and the maximum number of hits per sequence was three. Only the readings that passed the quality check were used and subjected to BLAST analysis against the NCBI 16 S bacterial database with sequence similarity threshold of 98% for classification. The bacterial species list for each bat individual was retrieved for downstream processing. The bacteria were ranked systematically up to the phylum level using the BacSyst v2.1 [24] programme in RStudio v4.3.1. Simultaneously, the raw FASTQ files for each bat individual were processed using another pipeline in the Python programming language (Python Software Foundation, https://ww.phyton.org/). This pipeline utilised various packages, including Nanostat [25], Kraken2 v2.1.4 [26], and Krona v2.8.1 [27] to compare the taxonomic assignment results. Both pipelines provide a comparable outcome.

### Statistical analysis

Output data from bioinformatic processing was further analysed downstream to generate an Operational Taxonomic Unit (OTUs) table, metadata file, and taxonomy table. These files were loaded into MicrobiomeAnalyst 2.0 (https://www.microbiomeanalyst.ca/) [28–30]. The metadata underwent rarefication [31] and several data processing steps, such as producing bacterial relative abundance, Good’s coverage measure [20], sparse correlations for compositional data (SparCC) [32–34], non-metric multidimensional scaling (NMDS), and analysis of similarities (ANOSIM) [20, 35]. This study used PAleontological Statistics (PAST) v.4.14 to do statistical analyses of alpha-diversity indices like dominance (D), Simpson (1-*D*), Shannon-Wiener (*H*’), and evenness (e^*H/S*^) [36, 37]. The Functional Annotation of Prokaryotic Taxa (FAPROTAX), a predictive metagenomic tool was used to infer putative functional groups of microbial communities based on taxonomic classifications identified in the samples [38, 39].

## Results

### 16 S rRNA gene sequencing analysis

From 17 insectivorous bats and 13 phytophagous bats, a total of 4,201,593 high-quality 16 S rRNA sequences were generated. The data were normalised, in which each OTU matrix is rarefied from each sample without replacement, resulting in all samples having the same number of total counts [31]. The minimum library size for this study was 15,048 sequences per individual.

**Fig. 2:**
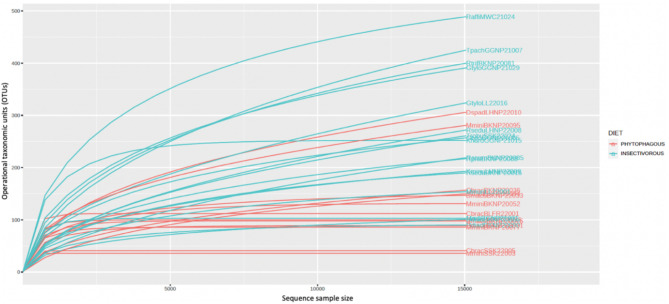
The rarefaction curves of OTUs. The x-axis shows the number of valid sequences per sample and the y-axis shows the observed operational taxonomic units (OTUs). Each line in the graph represents one sample. The diet grouping shown through two different colours. Abbreviation follows Table 1

The OTU rarefaction curves found in this study demonstrated an increase in the number of observed species with increasing sequencing depth. As is typical with sequencing data, the ends of the rarefaction curves flattened out as the number of sequences per sample increased (Fig. 2). Good’s Coverage measures how well each sample is covered by the reference database [20]; in this study, it had a value of 99.6%, meaning that most of the bacterial species in the samples had been identified. The bacterial microbiome was classified into 18 phyla, 43 classes, 100 orders, 210 families, and 1189 genera. The complete list of bacterial species is available in Additional file 1.

### Differences of microbiota diversity between two feeding guilds

Alpha-diversity indices namely dominance (D), Simpson (*1-D*), Shannon-Wiener (*H’*) and Evenness (*e*^*H/S*^) were calculated for each feeding groups and interpreted according to Lombogia et al., 2020 [37] (Table 2). There is no dominance taxon and no taxa evenness displayed by the bacteria community detected in either group. The Simpson (*1-D*) and Shannon (*H’*) values indicates that both phytophagous and insectivorous bats have a high microbial species diversity. Slightly higher in Simpson (*1-D*) value is the phytophagous bats. There is 92.4% of probability for two individuals of bacteria that picked randomly from phytophagous bats microbiome pool will belong to different species. For insectivorous bats, the probability is 91.5%. The highest Shannon index (*H’*) value belongs to phytophagous bats with 3.423 whereas 3.322 for insectivorous bats. As the number of OTUs grows and the distribution of individuals across taxonomic groups becomes more even, the value rises.

**Fig. 3:**
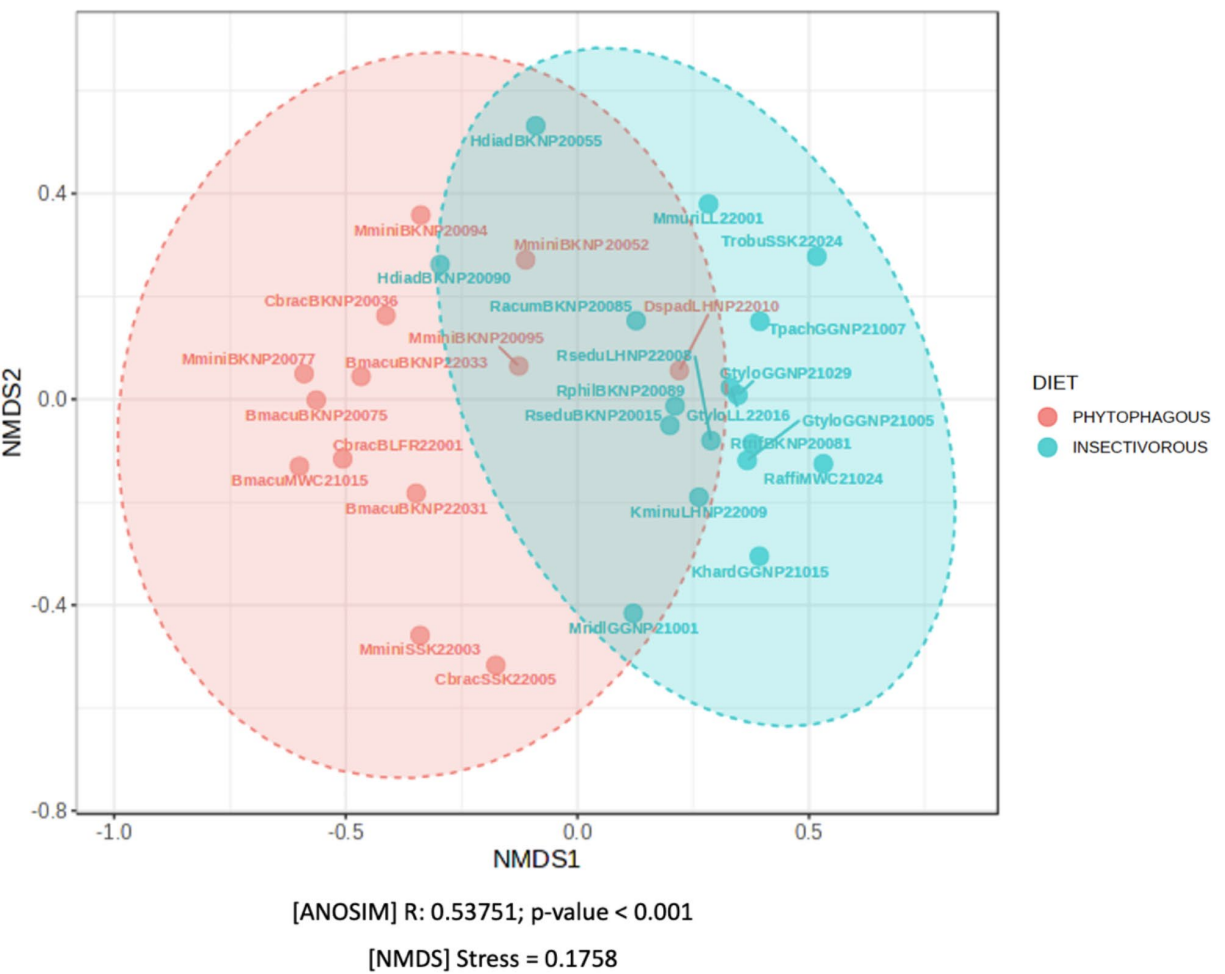
Non-metric multidimensional scaling (NMDS) analysis. Each point on the graph represents a single sample. The distance between two points indicates the degree of dissimilarity. The stress values less than 0.2 imply that the NMDS analysis is valid. The greater their similarity, the closer together the samples are in the graph. The sample ID abbreviation follows Table 1

The beta diversity analyses were performed with the one-way Analysis of Similarity (ANOSIM) yielded an R-value of 0.54 (*p* < 0.001) and the non-metric multidimensional scaling (NMDS) analysis plot shows distinct clustering based on dietary preferences, with minimal overlap between groups (stress value = 0.18) (Fig. 3). These results suggest that the observed differences are driven by diet, rather than individual species variation. The analysis also accounted for species as a factor, and no significant species-specific effects were found to explain the observed differences. Therefore, it can be concluded that the inter-group differences are greater than intra-group differences, which suggests that diet, rather than species composition is the primary factor influencing the bacterial composition variation.

**Table 2:** Table of alpha diversity indices calculated from phytophagous and insectivorous bats. The interpretations are based on lombogia et al., 2020 [37]

Diet preference	Alpha diversity indices	Value	Interpretation
Phytophagous	Dominance, *D*	0.076±0.000	No dominance
	Simpson, *1-D*	0.924±0.000	High diversity
	Shannon, *H’*	3.423±0.000	High diversity
	Evenness *e*^*H/S*^	0.019±0.000	No evenness
Insectivorous	Dominance, *D*	0.085±0.000	No dominance
	Simpson, *1-D*	0.915±0.000	High diversity
	Shannon, *H’*	3.322±0.000	High diversity
	Evenness, *e*^*H/S*^	0.007±0.000	No evenness

### Relative abundance of gut microbiome and functional group distribution

**Fig. 4:**
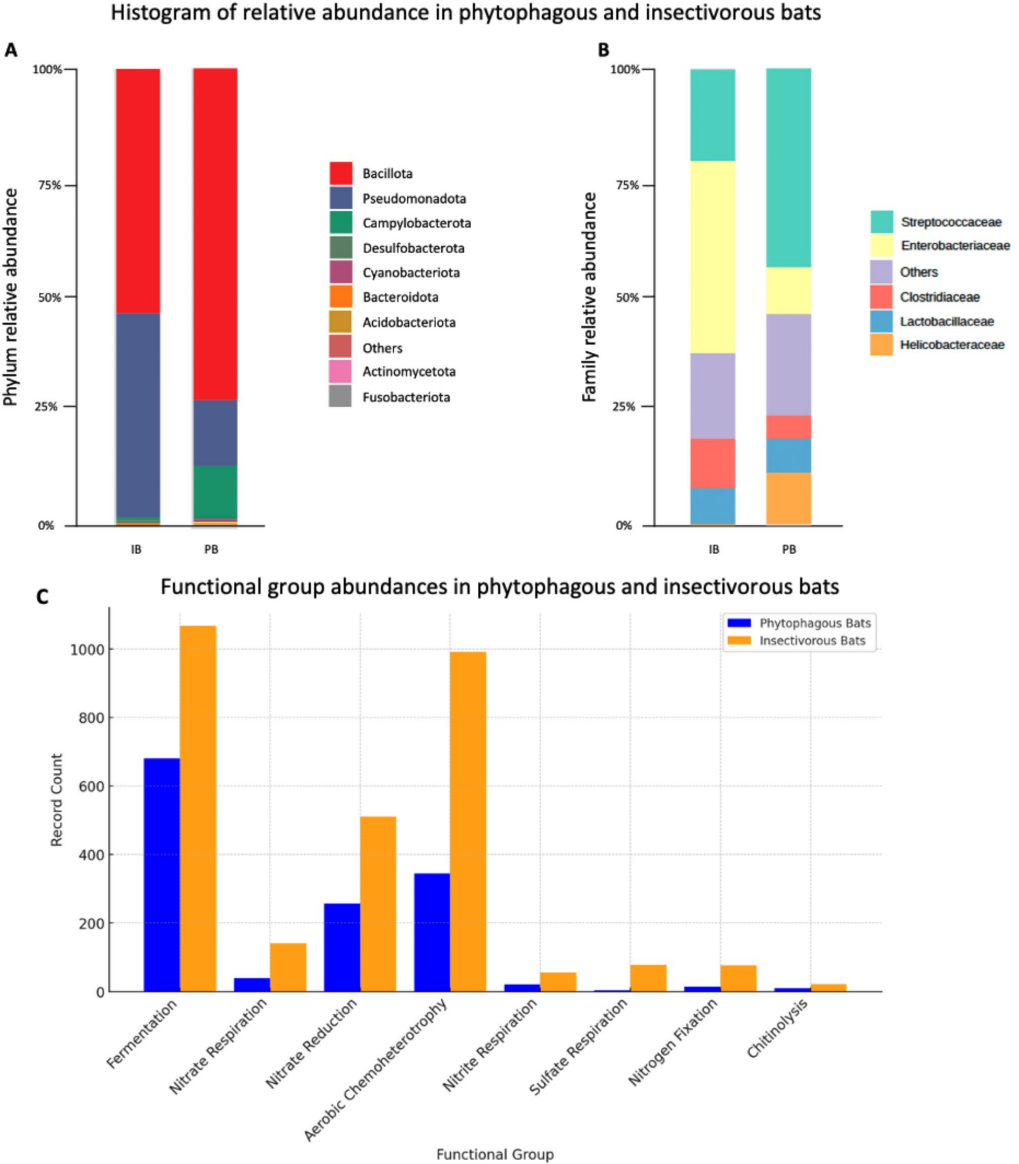
Histogram of relative and functional group abundance. (**A**) Relative abundance of the top 10 phylum. (**B**) Relative abundance of the top 5 families. Other phyla below top 10 and families below top 5 were grouped as “Others”. IB = Insectivorous bats, PB = Phytophagous bats. (**C**) Functional group abundance in phytophagous and insectivorous bats

A total of 27 bacterial phyla were identified from the sample set. To streamline the data presentation, the top 10 phyla and the top five families, ranked by relative abundance, were highlighted in Fig. 4.

The three most common bacterial phyla for both bat diets were Bacillota, Pseudomonadota, and Campylobacterota. Comparing the percentage of relative abundance between phytophagous and insectivorous bats, phytophagous bats host a higher percentage of Bacillota (72% ± 5% vs. 52% ± 3%) and Campylobacterota (11% ± 2%vs. 1% ± 0.5%), whereas insectivorous bats have a higher Pseudomonadota percentage (45% ± 2% vs. 14% ± 1%) (Fig. 4A).

When analyzing the relative abundance of the family, a large percentage of Streptococcaceae was detected in phytophagous bats (46% ± 3% vs. 21% ± 4%), whereas in insectivorous bats, a higher percentage of Enterobacteriaceae (42% ± 1% vs. 10% ± 1%) and Clostridiaceae (12% ± 2% vs. 5% ± 1%) were detected. Both types of diets had a similar percentage of Lactobacillaceae (8% ± 1% vs. 7% ± 1%). Interestingly, compared to insectivorous bats, the Helicobacteraceae family was prominently detected in phytophagous bats (11% ± 2% vs. <1% ± 0.2%) (Fig. 4B).

A comparative analysis of gut bacteria functional groups identified eight major groups in both phytophagous and insectivorous bats. These groups reveal distinct microbial functional patterns associated with each dietary preference. Both bat types show high detection of functional group related to fermentation. A notable difference is seen in the detection of aerobic chemoheterotrophy where phytophagous bats have 344 detections, while insectivorous bats have 991 (Fig. 4C; Table 3).

**Table 3:** The functional groups detected from both dietary groups

Functional Group	PB	IB	Family	Phylum
Fermentation	680	1068	Lactobacillaceae, Enterobacteriaceae	Bacillota, Pseudomonadota
Nitrate respiration	38	141	Enterobacteriaceae, Pseudomonadaceae	Pseudomonadota
Nitrate reduction	257	511	Enterobacteriaceae, Rhodobacteraceae	Proteobacteria, Pseudomonadota
Aerobic chemoheterotrophy	344	991	Bacillaceae, Enterococcaceae	Bacillota, Firmicutes
Nitrite respiration	20	55	Comamonadaceae, Enterobacteriaceae	Pseudomonadota
Sulfate respiration	3	77	Desulfovibrionaceae, Desulfobacteraceae	Proteobacteria, Firmicutes
Nitrogen fixation	14	76	Rhizobiaceae, Bradyrhizobiaceae	Proteobacteria
Chitinolysis	9	22	Bacillaceae, Enterobacteriaceae	Bacillota, Firmicutes

*PB = Phytophagous bats, IB = Insectivorous bats

### Bacterial correlation network

**Fig. 5:**
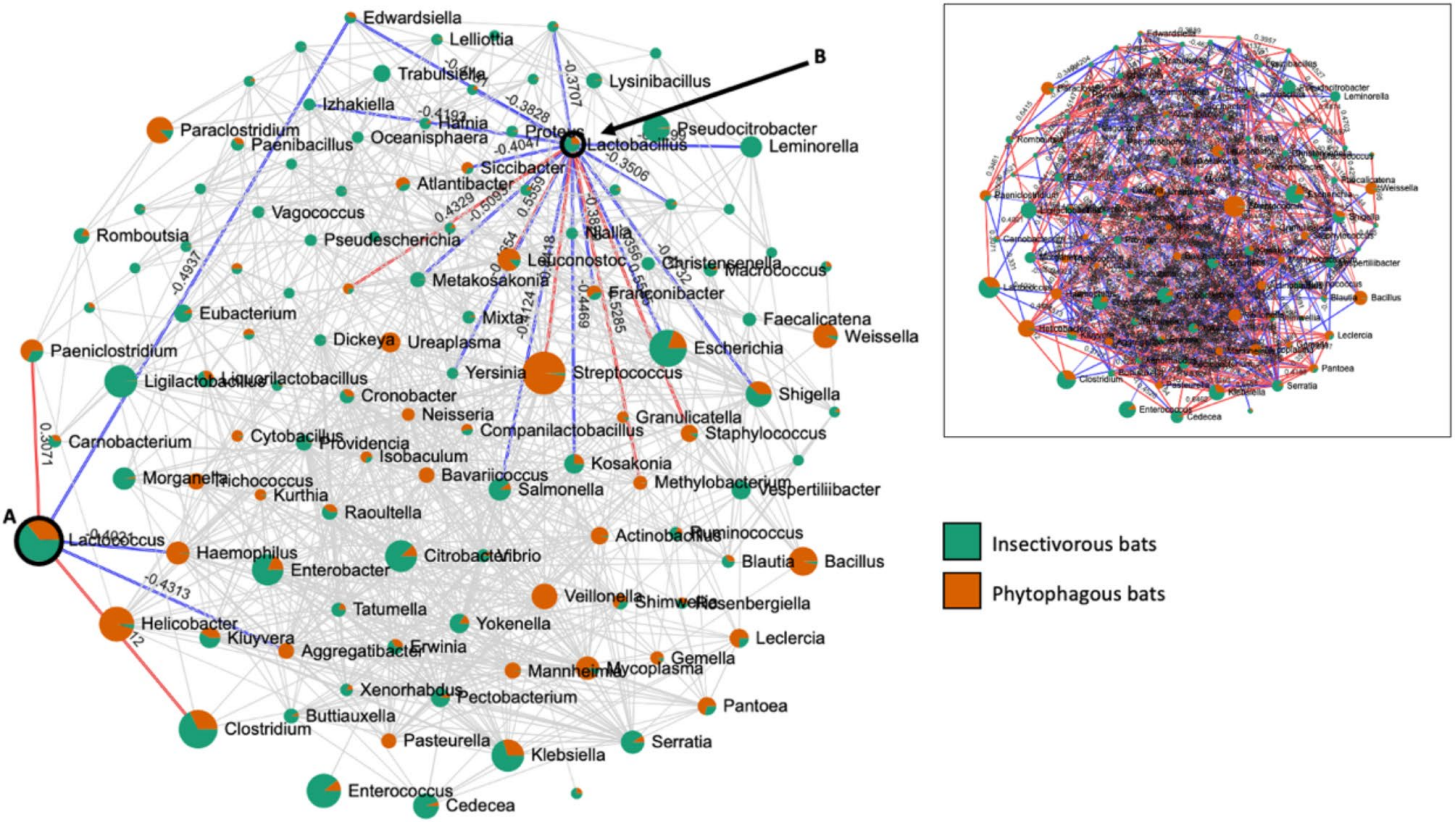
The SparCC correlation network. The image shows the correlation network of bacteria genera harboured by the bats. Node labelled A represent *Lactococcus* and node B represent *Lactobacillus*. The blue bar represents negative correlations, while red bar represents positive correlations. The digit on the bar showed the correlation value. The small inset figure displayed all correlations values for 117 nodes

An analysis of bacterial correlation networks using SparCC correlation coefficients produced a correlation network (Fig. 5). This study had successfully identified a total of 117 bacterial genera nodes that showed microbial associations within the bat’s microbiome community. The range of absolute correlation coefficient values was from − 0.6594 to 0.8583. The nodes represent different genera of bacteria, while the edges represent correlation coefficients between different genera. The size of the nodes represents abundance and is colored according to their respective diet groups. Due to the high number of correlations, only correlation networks of randomly selected *Lactococcus* and *Lactobacillus* were highlighted to show examples of positive and negative correlations. The full list of bacteria genus correlation tables can be referred to in Additional file 2.

## Discussions

### The microbiota diversity between the two feeding guilds

The results presented in this study were obtained from 17 insectivorous and 13 phytophagous bats. Although the number of phytophagous bat individuals studied here is fewer than those from the insectivorous group, a slightly more diverse microbiome was recovered from the phytophagous bats as compared to the insectivorous bats (Table 2). Due to the low number of samples, this data needed to be treated with caution, but nevertheless, it provides a baseline information on gut microbiome studies in Bornean bats. The plant-based diet contains complex plant materials such as hemicellulose, lignin derivatives, and insoluble starches. As a result, digestive systems will require enzymes from a wide range of microbe species, sustaining a rich biodiversity in the digestive system environment [21].

The high microbiome diversity detected in insectivorous bats was also documented in previous bat-microbe studies [e.g., 20, 22]. The nutrient requirements for these bats to survive in the wild are enormous; thus, during foraging, each bat individual is estimated to consume 61–84% of their body mass, or at least 4.8 g of arthropods per night [40, 41]. This large amount of insect consumption resulted in a diet that is heavy in proteins, fats, and nutrients. This condition could foster and make it an ideal environment for the growth of bacteria, thus contributing to the high bacterial diversity observed in this study. Similar diversity was observed for both diet groups, showing a relatively balanced bacterial composition.

### The relative and functional abundance of gut bacteria in bats

Fermentation was one of the most dominant functional groups in both phytophagous and insectivorous bats, with higher records in insectivorous bats (1068 vs. 680). This aligns with the presence of Bacillota in both groups, which are known for their role in carbohydrate fermentation [42, 43]. Despite the higher prevalence of Bacillota in phytophagous bats (72% ± 5% vs. 52% ± 3%), insectivorous bats exhibited a higher overall fermentation function, likely due to the substantial presence of Enterobacteriaceae (42% ± 1% vs. 10% ± 1%), a family that contributes to sugar metabolism [44, 45]. This indicates that functional dominance is influenced not just by phylum-level abundance but also by specific bacterial families and their metabolic activity.

Insectivorous bats exhibited significantly higher nitrate respiration (141 vs. 38) and nitrate reduction (511 vs. 257) compared to phytophagous bats. This corresponds with their higher relative abundance of Pseudomonadota (45% ± 2% vs. 14% ± 1%) and Enterobacteriaceae, both of which contain taxa involved in nitrogen cycling [46, 47]. The high nitrate-processing capability in insectivorous bats suggests an adaptation to their nitrogen-rich diet, derived from protein-heavy insect consumption [48, 49]. Conversely, the lower levels of nitrate respiration and reduction in phytophagous bats may reflect their carbohydrate-dominant diet, which produces less nitrogenous waste [50–52].

Aerobic chemoheterotrophy was substantially higher in insectivorous bats (991 vs. 344), indicating a greater metabolic flexibility in utilizing organic compounds. The functional potential for this process is associated with Bacillaceae, a family within Bacillota [53–55]. While phytophagous bats had a greater overall Bacillota representation, insectivorous bats exhibited higher functional activity, likely due to the greater need for diverse metabolic pathways to break down insect-derived macromolecules.

Nitrite and sulfate respiration were also higher in insectivorous bats (55 vs. 20 and 77 vs. 3, respectively). The families Comamonadaceae and Enterobacteriaceae (Pseudomonadota) contributed to nitrite respiration [56–60], while Desulfovibrionaceae and Desulfobacteraceae (Proteobacteria, Bacillota) were responsible for sulfate respiration [61, 62]. The greater sulfate-reducing capacity in insectivorous bats aligns with their higher intake of protein, which contains sulfur-rich amino acids [63–65]. In contrast, phytophagous bats, consuming plant-based diets with lower sulfur content, demonstrated minimal sulfate respiration activity [66–68].

Chitinolysis was recorded at a higher rate in insectivorous bats (22 vs. 9), reflecting their consumption of insect exoskeletons composed of chitin [69]. This function is primarily associated with Bacillaceae and Enterobacteriaceae (Bacillota) [53, 69–73]. The relatively low chitinolytic activity in phytophagous bats further reinforces the link between diet and microbiome functional roles, as plant-based diets do not necessitate chitin degradation, but rather more on degradation of plant polysaccharides [74].

Overall, these findings highlight that while bacterial phylum- and family-level abundances provide an overview of microbial composition, they do not always predict functional activity. The functional adaptations observed in insectivorous and phytophagous bats reflect their dietary specializations, with insectivorous bats demonstrating greater metabolic flexibility in nitrogen cycling, sulfate respiration, and chitinolysis, while phytophagous bats exhibit a microbiome more specialized in carbohydrate fermentation. These results emphasize the importance of integrating taxonomic and functional analyses for a comprehensive understanding of gut microbiome ecology in bats.

### Bacterial correlation network analysis

The correlation network analysis was used and managed to interpret the complex microbial interaction within two different bat diet preferences (Fig. 5). For example, the genus *Lactococcus* is positively related to *Clostridium*,* Helicobacter*, and *Paeniclostridium.* Suggesting that they are somehow aiding each other’s growth and/or survival in the bat gut. Previous research suggests that metabolic cross-feeding and co-survival in the environment may be the key elements generating the favourable correlations observed between microbiomes [75, 76].

The majority of members within the genus *Lactobacillus* are probiotic bacteria, which is a good bacterium [77–81]. The correlation network analysis revealed a negative relationship with 13 other bacterial genera. Some notable negative correlations are with the known pathogenic *Brenneria*, *Edwardsiella*, *Escherichia*, *Kosakonia*, *Leminorella*, *Metakosakonia*, *Pluralibacter*, *Salmonella*, *Shigella*, and *Yersinia*. The negative association suggests the presence of competition for the available resources, which signals its efforts to displace the surrounding bacteria [76, 82]. The significance of both good and bad bacteria in the gut microbiome is pivotal for various physiological functions involving an enormous community of microbiomes.

## Conclusions

This study successfully determined the gut microbiome community profiles of insectivorous and phytophagous bats in Borneo. The bacterial composition naturally correlates to the dietary preferences of bats. These data indicate that though bacterial phylum- and family-level abundances offer a general understanding of microbial composition, they do not dependably indicate the functional activity. Through correlation network analysis, it has been established that probiotic bacteria such as *Lactobacillus* play a crucial function in bats. They are negatively correlated with several well-known bacterial pathogens, such as *Salmonella* and *Yersinia*, indicating that they compete with these pathogens for resources. While the sample size is small, this study provides valuable insights into the functional diversity of gut microbiomes in insectivorous and phytophagous bats in Borneo, a region where such data is particularly scarce. The findings highlight how dietary preferences are linked to distinct functional groups of bacterial communities, contributing to the understanding of microbial ecology in these bat species. This research fills a significant data gap in Borneo, offering a foundational basis for future studies to explore the ecological and ecosystem roles of gut microbiomes in bats. Future research should focus on expanding sample sizes for more robust conclusions as well as investigating the potential implications of gut microbiomes in areas such as zoonotic disease transmission, biodiversity conservation, and their role in ecosystem health. This provides pathways for future research that will elucidate the nature of the symbiotic relationship between microflora communities and hosts.

## Electronic supplementary material

Below is the link to the electronic supplementary material.

Supplementary Material 1

Supplementary Material 2

## Data Availability

All data that support the findings of this study are available in main text. The datasets generated and analysed during the current study are already deposited in GenBank database, corresponding to the accession number in Table 1 (https://www.ncbi.nlm.nih.gov/bioproject/PRJNA1083214).
