# Supplementary material for: Gut microbiome community profiling of Bornean bats with different feeding guilds
Source: Anim Microbiome. 2025 Mar 5;7:21. doi: 10.1186/s42523-025-00389-w (PMC11881492; doi:10.1186/s42523-025-00389-w)
Supplement: Supplementary file 1 — Supplementary Material 1 [file 42523_2025_389_MOESM1_ESM.pdf]

Additional file 2

The correlation network between all studied bacterial genus.

This table showed the positive and negative correlation networks between each bacterial genus, together with its P-value.

Taxon1	Taxon2	Correlation	P-value
Acinetobacter	*Bacillus*	0.3842	0.0198
Acinetobacter	*Citrobacter*	-0.3164	0.0396
Acinetobacter	*Erwinia*	-0.364	0.0297
Acinetobacter	*Escherichia*	-0.3858	0.0198
Acinetobacter	*Franconibacter*	-0.3573	0.0396
Acinetobacter	*Gemella*	0.434	0.0099
Acinetobacter	*Granulicatella*	0.509	0.0099
Acinetobacter	*Kurthia*	0.479	0.0099
Acinetobacter	*Leuconostoc*	0.3239	0.0198
Acinetobacter	*Paraclostridium*	0.3369	0.0297
Acinetobacter	*Pasteurella*	0.3037	0.0297
Acinetobacter	*Providencia*	-0.3425	0.0495
Acinetobacter	*Salmonella*	-0.4629	0.0297
Acinetobacter	*Serratia*	-0.4625	0.0099
Acinetobacter	*Shigella*	-0.3197	0.0297
Acinetobacter	*Streptococcus*	0.5407	0.0099
Acinetobacter	*Trichococcus*	0.3749	0.0198
Actinobacillus	*Bavariicoccus*	0.5328	0.0099
Actinobacillus	*Citrobacter*	-0.4215	0.0099
Actinobacillus	*Clostridium*	-0.3908	0.0198
Actinobacillus	*Enterobacter*	-0.3114	0.0297
Actinobacillus	*Granulicatella*	0.3462	0.0396
Actinobacillus	*Haemophilus*	0.5853	0.0099
Actinobacillus	*Helicobacter*	0.5115	0.0099
Actinobacillus	*Klebsiella*	-0.3311	0.0495
Actinobacillus	*Kurthia*	0.3967	0.0198
Actinobacillus	*Lactococcus*	-0.3535	0.0198
Actinobacillus	*Mannheimia*	0.669	0.0099
Actinobacillus	*Mixta*	-0.3471	0.0099
Actinobacillus	*Mycoplasma*	0.5849	0.0099
Actinobacillus	*Paenibacillus*	0.4342	0.0099
Actinobacillus	*Pasteurella*	0.4786	0.0099
Actinobacillus	*Shigella*	-0.3606	0.0396
Actinobacillus	*Streptococcus*	0.4182	0.0297
Actinobacillus	*Trichococcus*	0.469	0.0099
Actinobacillus	*Ureaplasma*	0.4727	0.0099

Atlantibacter	*Cronobacter*	0.3334	0.0495
Atlantibacter	*Franconibacter*	0.3631	0.0495
Atlantibacter	*Haemophilus*	-0.3576	0.0198
Atlantibacter	*Izhakiella*	0.4682	0.0099
Atlantibacter	*Pseudescherichia*	0.493	0.0099
Atlantibacter	*Siccibacter*	0.4172	0.0297
Bacillus	*Acinetobacter*	0.3842	0.0198
Bacillus	*Cedecea*	-0.4438	0.0099
Bacillus	*Christensenella*	-0.3824	0.0297
Bacillus	*Citrobacter*	-0.4	0.0396
Bacillus	*Enterobacter*	-0.4726	0.0099
Bacillus	*Escherichia*	-0.5801	0.0099
Bacillus	*Granulicatella*	0.3959	0.0198
Bacillus	*Kosakonia*	-0.3351	0.0396
Bacillus	*Kurthia*	0.3566	0.0198
Bacillus	*Macrococcus*	0.3296	0.0495
Bacillus	*Pseudocitrobacter*	-0.397	0.0495
Bacillus	*Salmonella*	-0.3804	0.0495
Bacillus	*Shimwellia*	0.335	0.0297
Bacillus	*Staphylococcus*	0.5592	0.0099
Bacillus	*Streptococcus*	0.4191	0.0198
Bacillus	*Trichococcus*	0.3344	0.0396
Bacillus	*Weissella*	0.4446	0.0099
Bacillus	*Yokenella*	-0.4931	0.0099
Bavariicoccus	*Actinobacillus*	0.5328	0.0099
Bavariicoccus	*Clostridium*	-0.4016	0.0297
Bavariicoccus	*Cronobacter*	-0.3919	0.0198
Bavariicoccus	*Dickeya*	-0.3574	0.0099
Bavariicoccus	*Haemophilus*	0.5733	0.0099
Bavariicoccus	*Ligilactobacillus*	0.4203	0.0099
Bavariicoccus	*Mannheimia*	0.4553	0.0099
Bavariicoccus	*Mycoplasma*	0.3801	0.0198
Bavariicoccus	*Paenibacillus*	0.4385	0.0198
Bavariicoccus	*Pasteurella*	0.5518	0.0099
Bavariicoccus	*Providencia*	-0.3582	0.0297
Bavariicoccus	*Pseudescherichia*	-0.347	0.0297
Bavariicoccus	*Raoultella*	-0.4196	0.0198
Bavariicoccus	*Serratia*	-0.3736	0.0396
Bavariicoccus	*Shigella*	-0.3707	0.0297
Bavariicoccus	*Shimwellia*	-0.3483	0.0396
Bavariicoccus	*Streptococcus*	0.3608	0.0198
Bavariicoccus	*Tatumella*	-0.4053	0.0198
Bavariicoccus	*Ureaplasma*	0.5308	0.0198

Biostraticola	*Franconibacter*	0.3466	0.0495
Biostraticola	*Gibbsiella*	0.507	0.0099
Biostraticola	*Photobacterium*	0.3853	0.0198
Biostraticola	*Proteus*	0.3399	0.0099
Biostraticola	*Pseudescherichia*	0.3338	0.0297
Blautia	*Christensenella*	0.5776	0.0099
Blautia	*Enterococcus*	0.3819	0.0297
Blautia	*Fournierella*	0.6397	0.0099
Blautia	*Metakosakonia*	-0.3645	0.0198
Blautia	*Ruminococcus*	0.7408	0.0099
Blautia	*Serratia*	-0.3613	0.0297
Blautia	*Shigella*	-0.4126	0.0297
Blautia	*Vibrio*	-0.3768	0.0297
Budvicia	*Hafnia*	0.5166	0.0099
Budvicia	*Listeria*	-0.3745	0.0198
Budvicia	*Pluralibacter*	0.4375	0.0297
Budvicia	*Vagococcus*	-0.3248	0.0396
Budvicia	*Yersinia*	0.3834	0.0396
Buttiauxella	*Franconibacter*	-0.3205	0.0495
Buttiauxella	*Klebsiella*	0.3821	0.0099
Buttiauxella	*Metakosakonia*	-0.3676	0.0198
Buttiauxella	*Pectobacterium*	0.4766	0.0099
Carnobacterium	*Cedecea*	0.3084	0.0495
Carnobacterium	*Eubacterium*	-0.3595	0.0198
Carnobacterium	*Isobaculum*	0.3129	0.0297
Carnobacterium	*Lactobacillus*	0.3806	0.0297
Carnobacterium	*Ligilactobacillus*	0.3648	0.0297
Carnobacterium	*Paenibacillus*	0.3511	0.0396
Carnobacterium	*Pluralibacter*	-0.3568	0.0396
Carnobacterium	*Siccibacter*	-0.3527	0.0396
Carnobacterium	*Vagococcus*	0.5132	0.0099
Carnobacterium	*Yersinia*	-0.3969	0.0099
Cedecea	*Bacillus*	-0.4438	0.0099
Cedecea	*Carnobacterium*	0.3084	0.0495
Cedecea	*Clostridium*	-0.3432	0.0396
Cedecea	*Enterobacter*	0.3631	0.0396
Cedecea	*Granulicatella*	-0.3918	0.0099
Cedecea	*Klebsiella*	0.4756	0.0099
Cedecea	*Leclercia*	0.4063	0.0099
Cedecea	*Salmonella*	0.4032	0.0198
Cedecea	*Yokenella*	0.6762	0.0099
Chania	*Edwardsiella*	0.3566	0.0396
Chania	*Escherichia*	0.4078	0.0198

Chania	*Pluralibacter*	0.5963	0.0099
Chania	*Proteus*	0.347	0.0198
Chania	*Pseudomonas*	-0.3227	0.0495
Chania	*Shigella*	0.3818	0.0297
Chania	*Streptococcus*	-0.3947	0.0198
Christensenella	*Bacillus*	-0.3824	0.0297
Christensenella	*Blautia*	0.5776	0.0099
Christensenella	*Enterococcus*	0.3349	0.0396
Christensenella	*Eubacterium*	0.3672	0.0297
Christensenella	*Fournierella*	0.525	0.0099
Christensenella	*Helicobacter*	-0.3081	0.0396
Christensenella	*Pectobacterium*	0.3249	0.0396
Christensenella	*Proteus*	-0.3087	0.0297
Christensenella	*Pseudocitrobacter*	0.4891	0.0099
Christensenella	*Raoultella*	0.3583	0.0297
Christensenella	*Rosenbergiella*	-0.452	0.0099
Christensenella	*Ruminococcus*	0.6056	0.0099
Christensenella	*Staphylococcus*	-0.3469	0.0297
Citrobacter	*Acinetobacter*	-0.3164	0.0396
Citrobacter	*Actinobacillus*	-0.4215	0.0099
Citrobacter	*Bacillus*	-0.4	0.0396
Citrobacter	*Enterobacter*	0.5026	0.0099
Citrobacter	*Escherichia*	0.4639	0.0198
Citrobacter	*Granulicatella*	-0.4637	0.0099
Citrobacter	*Helicobacter*	-0.3729	0.0396
Citrobacter	*Kurthia*	-0.4204	0.0099
Citrobacter	*Leclercia*	0.3693	0.0297
Citrobacter	*Marinilactibacillus*	-0.3332	0.0495
Citrobacter	*Pectobacterium*	0.4695	0.0099
Citrobacter	*Pseudocitrobacter*	0.3168	0.0495
Citrobacter	*Raoultella*	0.4536	0.0198
Citrobacter	*Salmonella*	0.3954	0.0099
Citrobacter	*Serratia*	0.4353	0.0198
Citrobacter	*Shigella*	0.3888	0.0297
Citrobacter	*Streptococcus*	-0.412	0.0198
Citrobacter	*Tatumella*	0.3978	0.0099
Citrobacter	*Ureaplasma*	-0.3508	0.0297
Citrobacter	*Yokenella*	0.3335	0.0495
Clostridium	*Actinobacillus*	-0.3908	0.0198
Clostridium	*Bavariicoccus*	-0.4016	0.0297
Clostridium	*Cedecea*	-0.3432	0.0396
Clostridium	*Haemophilus*	-0.3511	0.0198
Clostridium	*Lactococcus*	0.4624	0.0099

Clostridium	*Mannheimia*	-0.3495	0.0495
Clostridium	*Paeniclostridium*	0.4639	0.0198
Clostridium	*Paraclostridium*	0.3745	0.0198
Clostridium	*Romboutsia*	0.5493	0.0099
Cronobacter	*Atlantibacter*	0.3334	0.0495
Cronobacter	*Bavariicoccus*	-0.3919	0.0198
Cronobacter	*Dickeya*	0.3321	0.0198
Cronobacter	*Enterobacter*	0.3379	0.0198
Cronobacter	*Escherichia*	0.4095	0.0198
Cronobacter	*Franconibacter*	0.4267	0.0198
Cronobacter	*Granulicatella*	-0.3617	0.0099
Cronobacter	*Haemophilus*	-0.3605	0.0297
Cronobacter	*Kosakonia*	0.5063	0.0099
Cronobacter	*Metakosakonia*	0.3907	0.0099
Cronobacter	*Mixta*	0.4012	0.0099
Cronobacter	*Mycoplasma*	-0.3953	0.0297
Cronobacter	*Pasteurella*	-0.315	0.0396
Cronobacter	*Ruminococcus*	-0.3173	0.0396
Cronobacter	*Salmonella*	0.618	0.0099
Cronobacter	*Serratia*	0.3406	0.0198
Cronobacter	*Shigella*	0.6403	0.0099
Cronobacter	*Streptococcus*	-0.3182	0.0396
Cronobacter	*Vibrio*	0.4224	0.0099
Cronobacter	*Xenorhabdus*	0.4922	0.0198
Dickeya	*Bavariicoccus*	-0.3574	0.0099
Dickeya	*Cronobacter*	0.3321	0.0198
Dickeya	*Izhakiella*	0.4064	0.0297
Dickeya	*Kluyvera*	0.4305	0.0198
Dickeya	*Morganella*	0.3963	0.0099
Dickeya	*Mycoplasma*	-0.3846	0.0198
Dickeya	*Paraclostridium*	-0.3942	0.0297
Dickeya	*Photobacterium*	-0.3548	0.0198
Dickeya	*Providencia*	0.5505	0.0099
Dickeya	*Pseudomonas*	0.3816	0.0396
Dickeya	*Raoultella*	0.3699	0.0198
Dickeya	*Romboutsia*	-0.3419	0.0297
Dickeya	*Tatumella*	0.4993	0.0198
Dickeya	*Vibrio*	0.3813	0.0297
Dickeya	*Xenorhabdus*	0.3542	0.0297
Edwardsiella	*Chania*	0.3566	0.0396
Edwardsiella	*Erwinia*	0.377	0.0297
Edwardsiella	*Kluyvera*	0.4033	0.0099
Edwardsiella	*Lactococcus*	-0.4313	0.0099

Edwardsiella	*Pectobacterium*	0.3396	0.0099
Edwardsiella	*Raoultella*	0.3045	0.0396
Enterobacter	*Actinobacillus*	-0.3114	0.0297
Enterobacter	*Bacillus*	-0.4726	0.0099
Enterobacter	*Cedecea*	0.3631	0.0396
Enterobacter	*Citrobacter*	0.5026	0.0099
Enterobacter	*Cronobacter*	0.3379	0.0198
Enterobacter	*Erwinia*	0.3618	0.0297
Enterobacter	*Escherichia*	0.3999	0.0297
Enterobacter	*Klebsiella*	0.5679	0.0099
Enterobacter	*Kosakonia*	0.4822	0.0099
Enterobacter	*Kurthia*	-0.4709	0.0099
Enterobacter	*Leclercia*	0.3925	0.0099
Enterobacter	*Leuconostoc*	-0.3497	0.0198
Enterobacter	*Mycoplasma*	-0.3497	0.0297
Enterobacter	*Pasteurella*	-0.4231	0.0198
Enterobacter	*Pectobacterium*	0.459	0.0198
Enterobacter	*Salmonella*	0.538	0.0099
Enterobacter	*Serratia*	0.5062	0.0099
Enterobacter	*Shigella*	0.3925	0.0396
Enterobacter	*Staphylococcus*	-0.4373	0.0198
Enterobacter	*Trichococcus*	-0.4412	0.0099
Enterobacter	*Weissella*	-0.3407	0.0297
Enterobacter	*Yokenella*	0.5015	0.0099
Enterococcus	*Blautia*	0.3819	0.0297
Enterococcus	*Christensenella*	0.3349	0.0396
Enterococcus	*Erwinia*	-0.5718	0.0198
Enterococcus	*Fournierella*	0.3284	0.0396
Enterococcus	*Helicobacter*	-0.3331	0.0495
Enterococcus	*Lactococcus*	0.4315	0.0297
Enterococcus	*Serratia*	-0.3241	0.0297
Enterococcus	*Vibrio*	-0.4023	0.0297
Enterococcus	*Xenorhabdus*	-0.5466	0.0099
Erwinia	*Acinetobacter*	-0.364	0.0297
Erwinia	*Edwardsiella*	0.377	0.0297
Erwinia	*Enterobacter*	0.3618	0.0297
Erwinia	*Enterococcus*	-0.5718	0.0198
Erwinia	*Granulicatella*	-0.3057	0.0396
Erwinia	*Kluyvera*	0.7023	0.0099
Erwinia	*Pasteurella*	-0.3845	0.0198
Erwinia	*Pectobacterium*	0.6341	0.0099
Erwinia	*Providencia*	0.4059	0.0198
Erwinia	*Raoultella*	0.4259	0.0099

Erwinia	*Salmonella*	0.5556	0.0099
Erwinia	*Serratia*	0.6181	0.0099
Erwinia	*Shimwellia*	0.4046	0.0198
Erwinia	*Tatumella*	0.5327	0.0099
Erwinia	*Ureaplasma*	-0.339	0.0099
Erwinia	*Vibrio*	0.5508	0.0099
Erwinia	*Xenorhabdus*	0.5617	0.0099
Escherichia	*Acinetobacter*	-0.3858	0.0198
Escherichia	*Bacillus*	-0.5801	0.0099
Escherichia	*Chania*	0.4078	0.0198
Escherichia	*Citrobacter*	0.4639	0.0198
Escherichia	*Cronobacter*	0.4095	0.0198
Escherichia	*Enterobacter*	0.3999	0.0297
Escherichia	*Franconibacter*	0.5597	0.0099
Escherichia	*Kosakonia*	0.4767	0.0099
Escherichia	*Kurthia*	-0.3559	0.0198
Escherichia	*Marinilactibacillus*	-0.4071	0.0198
Escherichia	*Metakosakonia*	0.4047	0.0099
Escherichia	*Mixta*	0.4378	0.0099
Escherichia	*Pluralibacter*	0.3897	0.0099
Escherichia	*Pseudocitrobacter*	0.3549	0.0297
Escherichia	*Salmonella*	0.4244	0.0099
Escherichia	*Shigella*	0.73	0.0099
Escherichia	*Siccibacter*	0.3027	0.0396
Escherichia	*Streptococcus*	-0.4507	0.0198
Escherichia	*Trichococcus*	-0.332	0.0396
Escherichia	*Weissella*	-0.3619	0.0099
Escherichia	*Yokenella*	0.3462	0.0396
Eubacterium	*Carnobacterium*	-0.3595	0.0198
Eubacterium	*Christensenella*	0.3672	0.0297
Eubacterium	*Melissococcus*	-0.3057	0.0495
Eubacterium	*Yersinia*	0.419	0.0099
Fournierella	*Blautia*	0.6397	0.0099
Fournierella	*Christensenella*	0.525	0.0099
Fournierella	*Enterococcus*	0.3284	0.0396
Fournierella	*Ligilactobacillus*	0.3729	0.0198
Fournierella	*Pseudocitrobacter*	0.423	0.0099
Fournierella	*Ruminococcus*	0.4433	0.0099
Fournierella	*Vibrio*	-0.3089	0.0396
Franconibacter	*Acinetobacter*	-0.3573	0.0396
Franconibacter	*Atlantibacter*	0.3631	0.0495
Franconibacter	*Biostraticola*	0.3466	0.0495
Franconibacter	*Buttiauxella*	-0.3205	0.0495

Franconibacter	*Cronobacter*	0.4267	0.0198
Franconibacter	*Escherichia*	0.5597	0.0099
Franconibacter	*Kosakonia*	0.5417	0.0099
Franconibacter	*Metakosakonia*	0.4274	0.0198
Franconibacter	*Pantoea*	0.3392	0.0297
Franconibacter	*Proteus*	0.3235	0.0495
Franconibacter	*Shigella*	0.6309	0.0099
Franconibacter	*Yokenella*	0.327	0.0198
Gemella	*Acinetobacter*	0.434	0.0099
Gemella	*Kurthia*	0.5025	0.0099
Gemella	*Marinilactibacillus*	0.448	0.0099
Gemella	*Streptococcus*	0.3431	0.0495
Gemella	*Trichococcus*	0.3153	0.0297
Gibbsiella	*Biostraticola*	0.507	0.0099
Gibbsiella	*Ligilactobacillus*	-0.4298	0.0099
Gibbsiella	*Macrococcus*	-0.3564	0.0297
Gibbsiella	*Photobacterium*	0.3761	0.0396
Gibbsiella	*Proteus*	0.5386	0.0099
Granulicatella	*Acinetobacter*	0.509	0.0099
Granulicatella	*Actinobacillus*	0.3462	0.0396
Granulicatella	*Bacillus*	0.3959	0.0198
Granulicatella	*Cedecea*	-0.3918	0.0099
Granulicatella	*Citrobacter*	-0.4637	0.0099
Granulicatella	*Cronobacter*	-0.3617	0.0099
Granulicatella	*Erwinia*	-0.3057	0.0396
Granulicatella	*Isobaculum*	0.4071	0.0198
Granulicatella	*Kurthia*	0.3282	0.0297
Granulicatella	*Macrococcus*	0.3354	0.0297
Granulicatella	*Salmonella*	-0.386	0.0297
Granulicatella	*Shimwellia*	-0.3206	0.0396
Granulicatella	*Tatumella*	-0.3672	0.0396
Granulicatella	*Trichococcus*	0.3423	0.0396
Granulicatella	*Ureaplasma*	0.3226	0.0297
Granulicatella	*Yokenella*	-0.3469	0.0198
Haemophilus	*Actinobacillus*	0.5853	0.0099
Haemophilus	*Atlantibacter*	-0.3576	0.0198
Haemophilus	*Bavariicoccus*	0.5733	0.0099
Haemophilus	*Clostridium*	-0.3511	0.0198
Haemophilus	*Cronobacter*	-0.3605	0.0297
Haemophilus	*Helicobacter*	0.3988	0.0099
Haemophilus	*Klebsiella*	-0.4184	0.0198
Haemophilus	*Kurthia*	0.4754	0.0099
Haemophilus	*Mannheimia*	0.6277	0.0099

Haemophilus	*Mycoplasma*	0.5735	0.0099
Haemophilus	*Paenibacillus*	0.4343	0.0198
Haemophilus	*Pasteurella*	0.655	0.0099
Haemophilus	*Raoultella*	-0.3884	0.0198
Haemophilus	*Shimwellia*	-0.4644	0.0198
Haemophilus	*Streptococcus*	0.5493	0.0099
Haemophilus	*Trichococcus*	0.553	0.0099
Haemophilus	*Ureaplasma*	0.5417	0.0099
Hafnia	*Budvicia*	0.5166	0.0099
Hafnia	*Ligilactobacillus*	-0.3019	0.0495
Hafnia	*Photobacterium*	0.4773	0.0099
Hafnia	*Pluralibacter*	0.3037	0.0198
Hafnia	*Ruminococcus*	-0.3083	0.0495
Hafnia	*Vagococcus*	-0.3519	0.0099
Hafnia	*Yersinia*	0.5751	0.0099
Helicobacter	*Actinobacillus*	0.5115	0.0099
Helicobacter	*Christensenella*	-0.3081	0.0396
Helicobacter	*Citrobacter*	-0.3729	0.0396
Helicobacter	*Enterococcus*	-0.3331	0.0495
Helicobacter	*Haemophilus*	0.3988	0.0099
Helicobacter	*Mycoplasma*	0.5608	0.0099
Helicobacter	*Pasteurella*	0.377	0.0297
Helicobacter	*Raoultella*	-0.4213	0.0099
Helicobacter	*Siccibacter*	0.3731	0.0297
Helicobacter	*Staphylococcus*	0.3803	0.0099
Helicobacter	*Streptococcus*	0.3966	0.0198
Helicobacter	*Trichococcus*	0.318	0.0297
Helicobacter	*Ureaplasma*	0.539	0.0099
Isobaculum	*Carnobacterium*	0.3129	0.0297
Isobaculum	*Granulicatella*	0.4071	0.0198
Isobaculum	*Klebsiella*	-0.367	0.0099
Isobaculum	*Ligilactobacillus*	0.5186	0.0099
Isobaculum	*Listeria*	0.3871	0.0099
Isobaculum	*Phytobacter*	0.3137	0.0297
Isobaculum	*Pseudescherichia*	0.3677	0.0198
Izhakiella	*Atlantibacter*	0.4682	0.0099
Izhakiella	*Dickeya*	0.4064	0.0297
Izhakiella	*Metakosakonia*	0.3624	0.0495
Izhakiella	*Mixta*	0.4453	0.0099
Izhakiella	*Pseudescherichia*	0.5416	0.0099
Izhakiella	*Siccibacter*	0.3745	0.0099
Izhakiella	*Staphylococcus*	-0.3478	0.0495
Klebsiella	*Actinobacillus*	-0.3311	0.0495

Klebsiella	*Buttiauxella*	0.3821	0.0099
Klebsiella	*Cedecea*	0.4756	0.0099
Klebsiella	*Enterobacter*	0.5679	0.0099
Klebsiella	*Haemophilus*	-0.4184	0.0198
Klebsiella	*Isobaculum*	-0.367	0.0099
Klebsiella	*Kluyvera*	0.3384	0.0495
Klebsiella	*Kurthia*	-0.4172	0.0099
Klebsiella	*Leclercia*	0.4041	0.0198
Klebsiella	*Mannheimia*	-0.3949	0.0099
Klebsiella	*Pectobacterium*	0.4446	0.0099
Klebsiella	*Raoultella*	0.3959	0.0198
Klebsiella	*Salmonella*	0.408	0.0297
Klebsiella	*Tatumella*	0.3057	0.0297
Klebsiella	*Trichococcus*	-0.5835	0.0099
Klebsiella	*Yokenella*	0.3911	0.0198
Kluyvera	*Dickeya*	0.4305	0.0198
Kluyvera	*Edwardsiella*	0.4033	0.0099
Kluyvera	*Erwinia*	0.7023	0.0099
Kluyvera	*Klebsiella*	0.3384	0.0495
Kluyvera	*Ligilactobacillus*	-0.4051	0.0297
Kluyvera	*Moellerella*	-0.3681	0.0396
Kluyvera	*Paeniclostridium*	-0.3265	0.0297
Kluyvera	*Pantoea*	0.3544	0.0396
Kluyvera	*Pectobacterium*	0.5224	0.0099
Kluyvera	*Providencia*	0.4508	0.0198
Kluyvera	*Raoultella*	0.6313	0.0099
Kluyvera	*Salmonella*	0.4193	0.0099
Kluyvera	*Serratia*	0.4746	0.0198
Kluyvera	*Shimwellia*	0.3899	0.0099
Kluyvera	*Tatumella*	0.6855	0.0099
Kluyvera	*Vibrio*	0.3782	0.0099
Kluyvera	*Xenorhabdus*	0.4662	0.0099
Kosakonia	*Bacillus*	-0.3351	0.0396
Kosakonia	*Cronobacter*	0.5063	0.0099
Kosakonia	*Enterobacter*	0.4822	0.0099
Kosakonia	*Escherichia*	0.4767	0.0099
Kosakonia	*Franconibacter*	0.5417	0.0099
Kosakonia	*Lactobacillus*	-0.4506	0.0396
Kosakonia	*Leuconostoc*	-0.3615	0.0297
Kosakonia	*Mannheimia*	-0.4052	0.0396
Kosakonia	*Paenibacillus*	-0.4608	0.0198
Kosakonia	*Pasteurella*	-0.4061	0.0297
Kosakonia	*Pectobacterium*	0.322	0.0495

Kosakonia	*Salmonella*	0.4303	0.0297
Kosakonia	*Serratia*	0.503	0.0099
Kosakonia	*Shigella*	0.6063	0.0099
Kosakonia	*Vibrio*	0.4423	0.0198
Kosakonia	*Xenorhabdus*	0.3264	0.0396
Kosakonia	*Yokenella*	0.3191	0.0495
Kurthia	*Acinetobacter*	0.479	0.0099
Kurthia	*Actinobacillus*	0.3967	0.0198
Kurthia	*Bacillus*	0.3566	0.0198
Kurthia	*Citrobacter*	-0.4204	0.0099
Kurthia	*Enterobacter*	-0.4709	0.0099
Kurthia	*Escherichia*	-0.3559	0.0198
Kurthia	*Gemella*	0.5025	0.0099
Kurthia	*Granulicatella*	0.3282	0.0297
Kurthia	*Haemophilus*	0.4754	0.0099
Kurthia	*Klebsiella*	-0.4172	0.0099
Kurthia	*Mannheimia*	0.4371	0.0099
Kurthia	*Marinilactibacillus*	0.3841	0.0099
Kurthia	*Pasteurella*	0.4108	0.0099
Kurthia	*Pectobacterium*	-0.3939	0.0297
Kurthia	*Salmonella*	-0.3653	0.0495
Kurthia	*Serratia*	-0.3429	0.0297
Kurthia	*Staphylococcus*	0.3493	0.0198
Kurthia	*Streptococcus*	0.5494	0.0099
Kurthia	*Trichococcus*	0.6954	0.0099
Kurthia	*Ureaplasma*	0.3235	0.0495
Kurthia	*Yokenella*	-0.3475	0.0198
Lactobacillus	*Carnobacterium*	0.3806	0.0297
Lactobacillus	*Kosakonia*	-0.4506	0.0396
Lactobacillus	*Leuconostoc*	0.3972	0.0297
Lactobacillus	*Macrococcus*	0.348	0.0396
Lactobacillus	*Salmonella*	-0.344	0.0099
Lactobacillus	*Serratia*	-0.3241	0.0396
Lactobacillus	*Shigella*	-0.4345	0.0099
Lactobacillus	*Yersinia*	-0.3317	0.0495
Lactococcus	*Actinobacillus*	-0.3535	0.0198
Lactococcus	*Clostridium*	0.4624	0.0099
Lactococcus	*Edwardsiella*	-0.4313	0.0099
Lactococcus	*Enterococcus*	0.4315	0.0297
Lactococcus	*Paeniclostridium*	0.4284	0.0099
Leclercia	*Cedecea*	0.4063	0.0099
Leclercia	*Citrobacter*	0.3693	0.0297
Leclercia	*Enterobacter*	0.3925	0.0099

Leclercia	*Klebsiella*	0.4041	0.0198
Leclercia	*Pantoea*	0.3673	0.0396
Leclercia	*Serratia*	0.3868	0.0198
Leclercia	*Yokenella*	0.3624	0.0396
Leuconostoc	*Acinetobacter*	0.3239	0.0198
Leuconostoc	*Enterobacter*	-0.3497	0.0198
Leuconostoc	*Kosakonia*	-0.3615	0.0297
Leuconostoc	*Lactobacillus*	0.3972	0.0297
Leuconostoc	*Macrococcus*	0.3851	0.0099
Leuconostoc	*Providencia*	-0.3312	0.0396
Leuconostoc	*Streptococcus*	0.3526	0.0198
Leuconostoc	*Trichococcus*	0.3384	0.0396
Leuconostoc	*Weissella*	0.5311	0.0099
Ligilactobacillus	*Bavariicoccus*	0.4203	0.0099
Ligilactobacillus	*Carnobacterium*	0.3648	0.0297
Ligilactobacillus	*Fournierella*	0.3729	0.0198
Ligilactobacillus	*Gibbsiella*	-0.4298	0.0099
Ligilactobacillus	*Hafnia*	-0.3019	0.0495
Ligilactobacillus	*Isobaculum*	0.5186	0.0099
Ligilactobacillus	*Kluyvera*	-0.4051	0.0297
Ligilactobacillus	*Paenibacillus*	0.3331	0.0297
Ligilactobacillus	*Photobacterium*	-0.3354	0.0495
Ligilactobacillus	*Shigella*	-0.3149	0.0396
Ligilactobacillus	*Tatumella*	-0.3643	0.0297
Ligilactobacillus	*Xenorhabdus*	-0.3133	0.0495
Listeria	*Budvicia*	-0.3745	0.0198
Listeria	*Isobaculum*	0.3871	0.0099
Listeria	*Macrococcus*	0.4791	0.0099
Listeria	*Marinilactibacillus*	0.5106	0.0099
Listeria	*Providencia*	0.3041	0.0396
Listeria	*Vagococcus*	0.3114	0.0396
Macrococcus	*Bacillus*	0.3296	0.0495
Macrococcus	*Gibbsiella*	-0.3564	0.0297
Macrococcus	*Granulicatella*	0.3354	0.0297
Macrococcus	*Lactobacillus*	0.348	0.0396
Macrococcus	*Leuconostoc*	0.3851	0.0099
Macrococcus	*Listeria*	0.4791	0.0099
Macrococcus	*Melissococcus*	0.4999	0.0198
Macrococcus	*Pseudocitrobacter*	-0.3604	0.0495
Macrococcus	*Staphylococcus*	0.4111	0.0198
Macrococcus	*Weissella*	0.4192	0.0198
Mannheimia	*Actinobacillus*	0.669	0.0099
Mannheimia	*Bavariicoccus*	0.4553	0.0099

Mannheimia	*Clostridium*	-0.3495	0.0495
Mannheimia	*Haemophilus*	0.6277	0.0099
Mannheimia	*Klebsiella*	-0.3949	0.0099
Mannheimia	*Kosakonia*	-0.4052	0.0396
Mannheimia	*Kurthia*	0.4371	0.0099
Mannheimia	*Mycoplasma*	0.4437	0.0099
Mannheimia	*Paenibacillus*	0.3936	0.0198
Mannheimia	*Pasteurella*	0.4241	0.0099
Mannheimia	*Raoultella*	-0.3714	0.0396
Mannheimia	*Serratia*	-0.4255	0.0099
Mannheimia	*Tatumella*	-0.3074	0.0495
Mannheimia	*Ureaplasma*	0.3493	0.0099
Marinilactibacillus	*Citrobacter*	-0.3332	0.0495
Marinilactibacillus	*Escherichia*	-0.4071	0.0198
Marinilactibacillus	*Gemella*	0.448	0.0099
Marinilactibacillus	*Kurthia*	0.3841	0.0099
Marinilactibacillus	*Listeria*	0.5106	0.0099
Marinilactibacillus	*Pluralibacter*	-0.3475	0.0198
Marinilactibacillus	*Shigella*	-0.3829	0.0198
Marinilactibacillus	*Streptococcus*	0.3223	0.0297
Marinilactibacillus	*Trichococcus*	0.4474	0.0099
Marinilactibacillus	*Vagococcus*	0.3304	0.0297
Melissococcus	*Eubacterium*	-0.3057	0.0495
Melissococcus	*Macrococcus*	0.4999	0.0198
Metakosakonia	*Blautia*	-0.3645	0.0198
Metakosakonia	*Buttiauxella*	-0.3676	0.0198
Metakosakonia	*Cronobacter*	0.3907	0.0099
Metakosakonia	*Escherichia*	0.4047	0.0099
Metakosakonia	*Franconibacter*	0.4274	0.0198
Metakosakonia	*Izhakiella*	0.3624	0.0495
Metakosakonia	*Mixta*	0.5871	0.0099
Metakosakonia	*Paenibacillus*	-0.3167	0.0495
Metakosakonia	*Ruminococcus*	-0.3828	0.0198
Metakosakonia	*Salmonella*	0.3717	0.0099
Metakosakonia	*Shigella*	0.4389	0.0198
Metakosakonia	*Siccibacter*	0.4481	0.0099
Mixta	*Actinobacillus*	-0.3471	0.0099
Mixta	*Cronobacter*	0.4012	0.0099
Mixta	*Escherichia*	0.4378	0.0099
Mixta	*Izhakiella*	0.4453	0.0099
Mixta	*Metakosakonia*	0.5871	0.0099
Mixta	*Pluralibacter*	0.3627	0.0297
Mixta	*Pseudescherichia*	0.4189	0.0198

Mixta	*Salmonella*	0.4866	0.0198
Mixta	*Shigella*	0.5015	0.0099
Moellerella	*Kluyvera*	-0.3681	0.0396
Moellerella	*Paeniclostridium*	0.3546	0.0198
Moellerella	*Paraclostridium*	0.3262	0.0099
Moellerella	*Romboutsia*	0.3687	0.0396
Moellerella	*Shimwellia*	-0.3746	0.0297
Moellerella	*Tatumella*	-0.4287	0.0099
Morganella	*Dickeya*	0.3963	0.0099
Morganella	*Proteus*	0.3168	0.0495
Morganella	*Providencia*	0.7478	0.0099
Morganella	*Rosenbergiella*	0.3979	0.0099
Morganella	*Serratia*	0.3889	0.0495
Morganella	*Vibrio*	0.4214	0.0099
Mycoplasma	*Actinobacillus*	0.5849	0.0099
Mycoplasma	*Bavariicoccus*	0.3801	0.0198
Mycoplasma	*Cronobacter*	-0.3953	0.0297
Mycoplasma	*Dickeya*	-0.3846	0.0198
Mycoplasma	*Enterobacter*	-0.3497	0.0297
Mycoplasma	*Haemophilus*	0.5735	0.0099
Mycoplasma	*Helicobacter*	0.5608	0.0099
Mycoplasma	*Mannheimia*	0.4437	0.0099
Mycoplasma	*Pasteurella*	0.6328	0.0099
Mycoplasma	*Photobacterium*	0.311	0.0495
Mycoplasma	*Providencia*	-0.3199	0.0495
Mycoplasma	*Salmonella*	-0.4857	0.0099
Mycoplasma	*Serratia*	-0.4153	0.0396
Mycoplasma	*Shimwellia*	-0.3647	0.0198
Mycoplasma	*Staphylococcus*	0.3469	0.0198
Mycoplasma	*Streptococcus*	0.4261	0.0099
Mycoplasma	*Ureaplasma*	0.6229	0.0099
Mycoplasma	*Vagococcus*	-0.4097	0.0297
Mycoplasma	*Vibrio*	-0.3932	0.0198
Paenibacillus	*Actinobacillus*	0.4342	0.0099
Paenibacillus	*Bavariicoccus*	0.4385	0.0198
Paenibacillus	*Carnobacterium*	0.3511	0.0396
Paenibacillus	*Haemophilus*	0.4343	0.0198
Paenibacillus	*Kosakonia*	-0.4608	0.0198
Paenibacillus	*Ligilactobacillus*	0.3331	0.0297
Paenibacillus	*Mannheimia*	0.3936	0.0198
Paenibacillus	*Metakosakonia*	-0.3167	0.0495
Paenibacillus	*Pasteurella*	0.4318	0.0099
Paenibacillus	*Shigella*	-0.3484	0.0297

Paenibacillus	*Vibrio*	-0.337	0.0495
Paeniclostridium	*Clostridium*	0.4639	0.0198
Paeniclostridium	*Kluyvera*	-0.3265	0.0297
Paeniclostridium	*Lactococcus*	0.4284	0.0099
Paeniclostridium	*Moellerella*	0.3546	0.0198
Paeniclostridium	*Paraclostridium*	0.4382	0.0198
Paeniclostridium	*Romboutsia*	0.5205	0.0099
Paeniclostridium	*Rosenbergiella*	0.3739	0.0099
Pantoea	*Franconibacter*	0.3392	0.0297
Pantoea	*Kluyvera*	0.3544	0.0396
Pantoea	*Leclercia*	0.3673	0.0396
Pantoea	*Shimwellia*	0.3379	0.0198
Paraclostridium	*Acinetobacter*	0.3369	0.0297
Paraclostridium	*Clostridium*	0.3745	0.0198
Paraclostridium	*Dickeya*	-0.3942	0.0297
Paraclostridium	*Moellerella*	0.3262	0.0099
Paraclostridium	*Paeniclostridium*	0.4382	0.0198
Paraclostridium	*Romboutsia*	0.6427	0.0099
Paraclostridium	*Xenorhabdus*	-0.4331	0.0198
Pasteurella	*Acinetobacter*	0.3037	0.0297
Pasteurella	*Actinobacillus*	0.4786	0.0099
Pasteurella	*Bavariicoccus*	0.5518	0.0099
Pasteurella	*Cronobacter*	-0.315	0.0396
Pasteurella	*Enterobacter*	-0.4231	0.0198
Pasteurella	*Erwinia*	-0.3845	0.0198
Pasteurella	*Haemophilus*	0.655	0.0099
Pasteurella	*Helicobacter*	0.377	0.0297
Pasteurella	*Kosakonia*	-0.4061	0.0297
Pasteurella	*Kurthia*	0.4108	0.0099
Pasteurella	*Mannheimia*	0.4241	0.0099
Pasteurella	*Mycoplasma*	0.6328	0.0099
Pasteurella	*Paenibacillus*	0.4318	0.0099
Pasteurella	*Pectobacterium*	-0.4388	0.0198
Pasteurella	*Providencia*	-0.3939	0.0495
Pasteurella	*Raoultella*	-0.3572	0.0297
Pasteurella	*Salmonella*	-0.419	0.0099
Pasteurella	*Serratia*	-0.5208	0.0099
Pasteurella	*Streptococcus*	0.5268	0.0099
Pasteurella	*Trichococcus*	0.4351	0.0099
Pasteurella	*Ureaplasma*	0.6275	0.0099
Pasteurella	*Vibrio*	-0.3526	0.0396
Pasteurella	*Yokenella*	-0.373	0.0396
Pectobacterium	*Buttiauxella*	0.4766	0.0099

Pectobacterium	*Christensenella*	0.3249	0.0396
Pectobacterium	*Citrobacter*	0.4695	0.0099
Pectobacterium	*Edwardsiella*	0.3396	0.0099
Pectobacterium	*Enterobacter*	0.459	0.0198
Pectobacterium	*Erwinia*	0.6341	0.0099
Pectobacterium	*Klebsiella*	0.4446	0.0099
Pectobacterium	*Kluyvera*	0.5224	0.0099
Pectobacterium	*Kosakonia*	0.322	0.0495
Pectobacterium	*Kurthia*	-0.3939	0.0297
Pectobacterium	*Pasteurella*	-0.4388	0.0198
Pectobacterium	*Providencia*	0.4203	0.0198
Pectobacterium	*Pseudocitrobacter*	0.4279	0.0198
Pectobacterium	*Raoultella*	0.65	0.0099
Pectobacterium	*Salmonella*	0.5301	0.0099
Pectobacterium	*Serratia*	0.414	0.0198
Pectobacterium	*Streptococcus*	-0.4921	0.0099
Pectobacterium	*Tatumella*	0.394	0.0198
Pectobacterium	*Trichococcus*	-0.4712	0.0099
Pectobacterium	*Ureaplasma*	-0.4338	0.0099
Pectobacterium	*Vibrio*	0.4001	0.0099
Pectobacterium	*Xenorhabdus*	0.5049	0.0099
Pectobacterium	*Yokenella*	0.4971	0.0099
Photobacterium	*Biostraticola*	0.3853	0.0198
Photobacterium	*Dickeya*	-0.3548	0.0198
Photobacterium	*Gibbsiella*	0.3761	0.0396
Photobacterium	*Hafnia*	0.4773	0.0099
Photobacterium	*Ligilactobacillus*	-0.3354	0.0495
Photobacterium	*Mycoplasma*	0.311	0.0495
Photobacterium	*Phytobacter*	0.5858	0.0099
Photobacterium	*Pluralibacter*	0.3706	0.0099
Photobacterium	*Vagococcus*	-0.4566	0.0099
Phytobacter	*Isobaculum*	0.3137	0.0297
Phytobacter	*Photobacterium*	0.5858	0.0099
Phytobacter	*Pseudescherichia*	0.5157	0.0099
Phytobacter	*Pseudomonas*	-0.3742	0.0198
Pluralibacter	*Budvicia*	0.4375	0.0297
Pluralibacter	*Carnobacterium*	-0.3568	0.0396
Pluralibacter	*Chania*	0.5963	0.0099
Pluralibacter	*Escherichia*	0.3897	0.0099
Pluralibacter	*Hafnia*	0.3037	0.0198
Pluralibacter	*Marinilactibacillus*	-0.3475	0.0198
Pluralibacter	*Mixta*	0.3627	0.0297
Pluralibacter	*Photobacterium*	0.3706	0.0099

Pluralibacter	*Pseudescherichia*	0.3341	0.0099
Pluralibacter	*Pseudocitrobacter*	0.5008	0.0099
Pluralibacter	*Shimwellia*	0.439	0.0099
Pluralibacter	*Streptococcus*	-0.3956	0.0198
Pluralibacter	*Vagococcus*	-0.386	0.0198
Pluralibacter	*Yokenella*	0.3162	0.0495
Proteus	*Biostraticola*	0.3399	0.0099
Proteus	*Chania*	0.347	0.0198
Proteus	*Christensenella*	-0.3087	0.0297
Proteus	*Franconibacter*	0.3235	0.0495
Proteus	*Gibbsiella*	0.5386	0.0099
Proteus	*Morganella*	0.3168	0.0495
Proteus	*Providencia*	0.4384	0.0099
Proteus	*Rosenbergiella*	0.4688	0.0099
Proteus	*Ruminococcus*	-0.3348	0.0495
Proteus	*Vibrio*	0.3142	0.0495
Providencia	*Acinetobacter*	-0.3425	0.0495
Providencia	*Bavariicoccus*	-0.3582	0.0297
Providencia	*Dickeya*	0.5505	0.0099
Providencia	*Erwinia*	0.4059	0.0198
Providencia	*Kluyvera*	0.4508	0.0198
Providencia	*Leuconostoc*	-0.3312	0.0396
Providencia	*Listeria*	0.3041	0.0396
Providencia	*Morganella*	0.7478	0.0099
Providencia	*Mycoplasma*	-0.3199	0.0495
Providencia	*Pasteurella*	-0.3939	0.0495
Providencia	*Pectobacterium*	0.4203	0.0198
Providencia	*Proteus*	0.4384	0.0099
Providencia	*Raoultella*	0.5158	0.0198
Providencia	*Serratia*	0.4387	0.0099
Providencia	*Tatumella*	0.4987	0.0099
Providencia	*Ureaplasma*	-0.3793	0.0297
Providencia	*Vibrio*	0.5619	0.0099
Providencia	*Xenorhabdus*	0.5304	0.0099
Pseudescherichia	*Atlantibacter*	0.493	0.0099
Pseudescherichia	*Bavariicoccus*	-0.347	0.0297
Pseudescherichia	*Biostraticola*	0.3338	0.0297
Pseudescherichia	*Isobaculum*	0.3677	0.0198
Pseudescherichia	*Izhakiella*	0.5416	0.0099
Pseudescherichia	*Mixta*	0.4189	0.0198
Pseudescherichia	*Phytobacter*	0.5157	0.0099
Pseudescherichia	*Pluralibacter*	0.3341	0.0099
Pseudocitrobacter	*Bacillus*	-0.397	0.0495

Pseudocitrobacter	*Christensenella*	0.4891	0.0099
Pseudocitrobacter	*Citrobacter*	0.3168	0.0495
Pseudocitrobacter	*Escherichia*	0.3549	0.0297
Pseudocitrobacter	*Fournierella*	0.423	0.0099
Pseudocitrobacter	*Macrococcus*	-0.3604	0.0495
Pseudocitrobacter	*Pectobacterium*	0.4279	0.0198
Pseudocitrobacter	*Pluralibacter*	0.5008	0.0099
Pseudocitrobacter	*Raoultella*	0.3397	0.0396
Pseudocitrobacter	*Ruminococcus*	0.3277	0.0495
Pseudocitrobacter	*Yokenella*	0.372	0.0297
Pseudomonas	*Chania*	-0.3227	0.0495
Pseudomonas	*Dickeya*	0.3816	0.0396
Pseudomonas	*Phytobacter*	-0.3742	0.0198
Raoultella	*Bavariicoccus*	-0.4196	0.0198
Raoultella	*Christensenella*	0.3583	0.0297
Raoultella	*Citrobacter*	0.4536	0.0198
Raoultella	*Dickeya*	0.3699	0.0198
Raoultella	*Edwardsiella*	0.3045	0.0396
Raoultella	*Erwinia*	0.4259	0.0099
Raoultella	*Haemophilus*	-0.3884	0.0198
Raoultella	*Helicobacter*	-0.4213	0.0099
Raoultella	*Klebsiella*	0.3959	0.0198
Raoultella	*Kluyvera*	0.6313	0.0099
Raoultella	*Mannheimia*	-0.3714	0.0396
Raoultella	*Pasteurella*	-0.3572	0.0297
Raoultella	*Pectobacterium*	0.65	0.0099
Raoultella	*Providencia*	0.5158	0.0198
Raoultella	*Pseudocitrobacter*	0.3397	0.0396
Raoultella	*Serratia*	0.3548	0.0396
Raoultella	*Shimwellia*	0.3615	0.0297
Raoultella	*Streptococcus*	-0.3605	0.0495
Raoultella	*Tatumella*	0.6603	0.0099
Raoultella	*Trichococcus*	-0.3376	0.0396
Raoultella	*Ureaplasma*	-0.4147	0.0099
Raoultella	*Vibrio*	0.3166	0.0297
Raoultella	*Yersinia*	0.4311	0.0099
Raoultella	*Yokenella*	0.355	0.0396
Romboutsia	*Clostridium*	0.5493	0.0099
Romboutsia	*Dickeya*	-0.3419	0.0297
Romboutsia	*Moellerella*	0.3687	0.0396
Romboutsia	*Paeniclostridium*	0.5205	0.0099
Romboutsia	*Paraclostridium*	0.6427	0.0099
Rosenbergiella	*Christensenella*	-0.452	0.0099

Rosenbergiella	*Morganella*	0.3979	0.0099
Rosenbergiella	*Paeniclostridium*	0.3739	0.0099
Rosenbergiella	*Proteus*	0.4688	0.0099
Rosenbergiella	*Weissella*	0.3939	0.0099
Ruminococcus	*Blautia*	0.7408	0.0099
Ruminococcus	*Christensenella*	0.6056	0.0099
Ruminococcus	*Cronobacter*	-0.3173	0.0396
Ruminococcus	*Fournierella*	0.4433	0.0099
Ruminococcus	*Hafnia*	-0.3083	0.0495
Ruminococcus	*Metakosakonia*	-0.3828	0.0198
Ruminococcus	*Proteus*	-0.3348	0.0495
Ruminococcus	*Pseudocitrobacter*	0.3277	0.0495
Ruminococcus	*Vibrio*	-0.3984	0.0099
Salmonella	*Acinetobacter*	-0.4629	0.0297
Salmonella	*Bacillus*	-0.3804	0.0495
Salmonella	*Cedecea*	0.4032	0.0198
Salmonella	*Citrobacter*	0.3954	0.0099
Salmonella	*Cronobacter*	0.618	0.0099
Salmonella	*Enterobacter*	0.538	0.0099
Salmonella	*Erwinia*	0.5556	0.0099
Salmonella	*Escherichia*	0.4244	0.0099
Salmonella	*Granulicatella*	-0.386	0.0297
Salmonella	*Klebsiella*	0.408	0.0297
Salmonella	*Kluyvera*	0.4193	0.0099
Salmonella	*Kosakonia*	0.4303	0.0297
Salmonella	*Kurthia*	-0.3653	0.0495
Salmonella	*Lactobacillus*	-0.344	0.0099
Salmonella	*Metakosakonia*	0.3717	0.0099
Salmonella	*Mixta*	0.4866	0.0198
Salmonella	*Mycoplasma*	-0.4857	0.0099
Salmonella	*Pasteurella*	-0.419	0.0099
Salmonella	*Pectobacterium*	0.5301	0.0099
Salmonella	*Serratia*	0.451	0.0198
Salmonella	*Shigella*	0.5488	0.0099
Salmonella	*Siccibacter*	0.3291	0.0396
Salmonella	*Staphylococcus*	-0.3939	0.0396
Salmonella	*Streptococcus*	-0.5499	0.0099
Salmonella	*Tatumella*	0.3474	0.0297
Salmonella	*Trichococcus*	-0.4962	0.0198
Salmonella	*Vibrio*	0.3822	0.0297
Salmonella	*Yokenella*	0.6827	0.0099
Serratia	*Acinetobacter*	-0.4625	0.0099
Serratia	*Bavariicoccus*	-0.3736	0.0396

Serratia	*Blautia*	-0.3613	0.0297
Serratia	*Citrobacter*	0.4353	0.0198
Serratia	*Cronobacter*	0.3406	0.0198
Serratia	*Enterobacter*	0.5062	0.0099
Serratia	*Enterococcus*	-0.3241	0.0297
Serratia	*Erwinia*	0.6181	0.0099
Serratia	*Kluyvera*	0.4746	0.0198
Serratia	*Kosakonia*	0.503	0.0099
Serratia	*Kurthia*	-0.3429	0.0297
Serratia	*Lactobacillus*	-0.3241	0.0396
Serratia	*Leclercia*	0.3868	0.0198
Serratia	*Mannheimia*	-0.4255	0.0099
Serratia	*Morganella*	0.3889	0.0495
Serratia	*Mycoplasma*	-0.4153	0.0396
Serratia	*Pasteurella*	-0.5208	0.0099
Serratia	*Pectobacterium*	0.414	0.0198
Serratia	*Providencia*	0.4387	0.0099
Serratia	*Raoultella*	0.3548	0.0396
Serratia	*Salmonella*	0.451	0.0198
Serratia	*Shigella*	0.4591	0.0099
Serratia	*Streptococcus*	-0.378	0.0297
Serratia	*Tatumella*	0.344	0.0099
Serratia	*Ureaplasma*	-0.4753	0.0099
Serratia	*Vibrio*	0.4248	0.0099
Serratia	*Xenorhabdus*	0.4406	0.0198
Shigella	*Acinetobacter*	-0.3197	0.0297
Shigella	*Actinobacillus*	-0.3606	0.0396
Shigella	*Bavariicoccus*	-0.3707	0.0297
Shigella	*Blautia*	-0.4126	0.0297
Shigella	*Chania*	0.3818	0.0297
Shigella	*Citrobacter*	0.3888	0.0297
Shigella	*Cronobacter*	0.6403	0.0099
Shigella	*Enterobacter*	0.3925	0.0396
Shigella	*Escherichia*	0.73	0.0099
Shigella	*Franconibacter*	0.6309	0.0099
Shigella	*Kosakonia*	0.6063	0.0099
Shigella	*Lactobacillus*	-0.4345	0.0099
Shigella	*Ligilactobacillus*	-0.3149	0.0396
Shigella	*Marinilactibacillus*	-0.3829	0.0198
Shigella	*Metakosakonia*	0.4389	0.0198
Shigella	*Mixta*	0.5015	0.0099
Shigella	*Paenibacillus*	-0.3484	0.0297
Shigella	*Salmonella*	0.5488	0.0099

Shigella	*Serratia*	0.4591	0.0099
Shigella	*Siccibacter*	0.3395	0.0495
Shigella	*Streptococcus*	-0.345	0.0297
Shimwellia	*Bacillus*	0.335	0.0297
Shimwellia	*Bavariicoccus*	-0.3483	0.0396
Shimwellia	*Erwinia*	0.4046	0.0198
Shimwellia	*Granulicatella*	-0.3206	0.0396
Shimwellia	*Haemophilus*	-0.4644	0.0198
Shimwellia	*Kluyvera*	0.3899	0.0099
Shimwellia	*Moellerella*	-0.3746	0.0297
Shimwellia	*Mycoplasma*	-0.3647	0.0198
Shimwellia	*Pantoea*	0.3379	0.0198
Shimwellia	*Pluralibacter*	0.439	0.0099
Shimwellia	*Raoultella*	0.3615	0.0297
Shimwellia	*Tatumella*	0.4391	0.0198
Shimwellia	*Ureaplasma*	-0.3842	0.0297
Shimwellia	*Vibrio*	0.3415	0.0396
Siccibacter	*Atlantibacter*	0.4172	0.0297
Siccibacter	*Carnobacterium*	-0.3527	0.0396
Siccibacter	*Escherichia*	0.3027	0.0396
Siccibacter	*Helicobacter*	0.3731	0.0297
Siccibacter	*Izhakiella*	0.3745	0.0099
Siccibacter	*Metakosakonia*	0.4481	0.0099
Siccibacter	*Salmonella*	0.3291	0.0396
Siccibacter	*Shigella*	0.3395	0.0495
Siccibacter	*Vagococcus*	-0.4111	0.0198
Staphylococcus	*Bacillus*	0.5592	0.0099
Staphylococcus	*Christensenella*	-0.3469	0.0297
Staphylococcus	*Enterobacter*	-0.4373	0.0198
Staphylococcus	*Helicobacter*	0.3803	0.0099
Staphylococcus	*Izhakiella*	-0.3478	0.0495
Staphylococcus	*Kurthia*	0.3493	0.0198
Staphylococcus	*Macrococcus*	0.4111	0.0198
Staphylococcus	*Mycoplasma*	0.3469	0.0198
Staphylococcus	*Salmonella*	-0.3939	0.0396
Staphylococcus	*Streptococcus*	0.4265	0.0099
Staphylococcus	*Trichococcus*	0.4805	0.0099
Staphylococcus	*Weissella*	0.436	0.0099
Staphylococcus	*Yokenella*	-0.4568	0.0198
Streptococcus	*Acinetobacter*	0.5407	0.0099
Streptococcus	*Actinobacillus*	0.4182	0.0297
Streptococcus	*Bacillus*	0.4191	0.0198
Streptococcus	*Bavariicoccus*	0.3608	0.0198

Streptococcus	*Chania*	-0.3947	0.0198
Streptococcus	*Citrobacter*	-0.412	0.0198
Streptococcus	*Cronobacter*	-0.3182	0.0396
Streptococcus	*Escherichia*	-0.4507	0.0198
Streptococcus	*Gemella*	0.3431	0.0495
Streptococcus	*Haemophilus*	0.5493	0.0099
Streptococcus	*Helicobacter*	0.3966	0.0198
Streptococcus	*Kurthia*	0.5494	0.0099
Streptococcus	*Leuconostoc*	0.3526	0.0198
Streptococcus	*Marinilactibacillus*	0.3223	0.0297
Streptococcus	*Mycoplasma*	0.4261	0.0099
Streptococcus	*Pasteurella*	0.5268	0.0099
Streptococcus	*Pectobacterium*	-0.4921	0.0099
Streptococcus	*Pluralibacter*	-0.3956	0.0198
Streptococcus	*Raoultella*	-0.3605	0.0495
Streptococcus	*Salmonella*	-0.5499	0.0099
Streptococcus	*Serratia*	-0.378	0.0297
Streptococcus	*Shigella*	-0.345	0.0297
Streptococcus	*Staphylococcus*	0.4265	0.0099
Streptococcus	*Tatumella*	-0.3303	0.0297
Streptococcus	*Trichococcus*	0.555	0.0198
Streptococcus	*Ureaplasma*	0.5659	0.0099
Streptococcus	*Yokenella*	-0.5505	0.0099
Tatumella	*Bavariicoccus*	-0.4053	0.0198
Tatumella	*Citrobacter*	0.3978	0.0099
Tatumella	*Dickeya*	0.4993	0.0198
Tatumella	*Erwinia*	0.5327	0.0099
Tatumella	*Granulicatella*	-0.3672	0.0396
Tatumella	*Klebsiella*	0.3057	0.0297
Tatumella	*Kluyvera*	0.6855	0.0099
Tatumella	*Ligilactobacillus*	-0.3643	0.0297
Tatumella	*Mannheimia*	-0.3074	0.0495
Tatumella	*Moellerella*	-0.4287	0.0099
Tatumella	*Pectobacterium*	0.394	0.0198
Tatumella	*Providencia*	0.4987	0.0099
Tatumella	*Raoultella*	0.6603	0.0099
Tatumella	*Salmonella*	0.3474	0.0297
Tatumella	*Serratia*	0.344	0.0099
Tatumella	*Shimwellia*	0.4391	0.0198
Tatumella	*Streptococcus*	-0.3303	0.0297
Tatumella	*Ureaplasma*	-0.3913	0.0099
Tatumella	*Vibrio*	0.4467	0.0297
Trichococcus	*Acinetobacter*	0.3749	0.0198

Trichococcus	*Actinobacillus*	0.469	0.0099
Trichococcus	*Bacillus*	0.3344	0.0396
Trichococcus	*Enterobacter*	-0.4412	0.0099
Trichococcus	*Escherichia*	-0.332	0.0396
Trichococcus	*Gemella*	0.3153	0.0297
Trichococcus	*Granulicatella*	0.3423	0.0396
Trichococcus	*Haemophilus*	0.553	0.0099
Trichococcus	*Helicobacter*	0.318	0.0297
Trichococcus	*Klebsiella*	-0.5835	0.0099
Trichococcus	*Kurthia*	0.6954	0.0099
Trichococcus	*Leuconostoc*	0.3384	0.0396
Trichococcus	*Marinilactibacillus*	0.4474	0.0099
Trichococcus	*Pasteurella*	0.4351	0.0099
Trichococcus	*Pectobacterium*	-0.4712	0.0099
Trichococcus	*Raoultella*	-0.3376	0.0396
Trichococcus	*Salmonella*	-0.4962	0.0198
Trichococcus	*Staphylococcus*	0.4805	0.0099
Trichococcus	*Streptococcus*	0.555	0.0198
Trichococcus	*Weissella*	0.3955	0.0099
Trichococcus	*Yokenella*	-0.4188	0.0198
Ureaplasma	*Actinobacillus*	0.4727	0.0099
Ureaplasma	*Bavariicoccus*	0.5308	0.0198
Ureaplasma	*Citrobacter*	-0.3508	0.0297
Ureaplasma	*Erwinia*	-0.339	0.0099
Ureaplasma	*Granulicatella*	0.3226	0.0297
Ureaplasma	*Haemophilus*	0.5417	0.0099
Ureaplasma	*Helicobacter*	0.539	0.0099
Ureaplasma	*Kurthia*	0.3235	0.0495
Ureaplasma	*Mannheimia*	0.3493	0.0099
Ureaplasma	*Mycoplasma*	0.6229	0.0099
Ureaplasma	*Pasteurella*	0.6275	0.0099
Ureaplasma	*Pectobacterium*	-0.4338	0.0099
Ureaplasma	*Providencia*	-0.3793	0.0297
Ureaplasma	*Raoultella*	-0.4147	0.0099
Ureaplasma	*Serratia*	-0.4753	0.0099
Ureaplasma	*Shimwellia*	-0.3842	0.0297
Ureaplasma	*Streptococcus*	0.5659	0.0099
Ureaplasma	*Tatumella*	-0.3913	0.0099
Ureaplasma	*Vibrio*	-0.4072	0.0099
Vagococcus	*Budvicia*	-0.3248	0.0396
Vagococcus	*Carnobacterium*	0.5132	0.0099
Vagococcus	*Hafnia*	-0.3519	0.0099
Vagococcus	*Listeria*	0.3114	0.0396

Vagococcus	*Marinilactibacillus*	0.3304	0.0297
Vagococcus	*Mycoplasma*	-0.4097	0.0297
Vagococcus	*Photobacterium*	-0.4566	0.0099
Vagococcus	*Pluralibacter*	-0.386	0.0198
Vagococcus	*Siccibacter*	-0.4111	0.0198
Vagococcus	*Yersinia*	-0.349	0.0198
Vibrio	*Blautia*	-0.3768	0.0297
Vibrio	*Cronobacter*	0.4224	0.0099
Vibrio	*Dickeya*	0.3813	0.0297
Vibrio	*Enterococcus*	-0.4023	0.0297
Vibrio	*Erwinia*	0.5508	0.0099
Vibrio	*Fournierella*	-0.3089	0.0396
Vibrio	*Kluyvera*	0.3782	0.0099
Vibrio	*Kosakonia*	0.4423	0.0198
Vibrio	*Morganella*	0.4214	0.0099
Vibrio	*Mycoplasma*	-0.3932	0.0198
Vibrio	*Paenibacillus*	-0.337	0.0495
Vibrio	*Pasteurella*	-0.3526	0.0396
Vibrio	*Pectobacterium*	0.4001	0.0099
Vibrio	*Proteus*	0.3142	0.0495
Vibrio	*Providencia*	0.5619	0.0099
Vibrio	*Raoultella*	0.3166	0.0297
Vibrio	*Ruminococcus*	-0.3984	0.0099
Vibrio	*Salmonella*	0.3822	0.0297
Vibrio	*Serratia*	0.4248	0.0099
Vibrio	*Shimwellia*	0.3415	0.0396
Vibrio	*Tatumella*	0.4467	0.0297
Vibrio	*Ureaplasma*	-0.4072	0.0099
Vibrio	*Xenorhabdus*	0.4424	0.0099
Weissella	*Bacillus*	0.4446	0.0099
Weissella	*Enterobacter*	-0.3407	0.0297
Weissella	*Escherichia*	-0.3619	0.0099
Weissella	*Leuconostoc*	0.5311	0.0099
Weissella	*Macrococcus*	0.4192	0.0198
Weissella	*Rosenbergiella*	0.3939	0.0099
Weissella	*Staphylococcus*	0.436	0.0099
Weissella	*Trichococcus*	0.3955	0.0099
Weissella	*Yokenella*	-0.432	0.0297
Xenorhabdus	*Cronobacter*	0.4922	0.0198
Xenorhabdus	*Dickeya*	0.3542	0.0297
Xenorhabdus	*Enterococcus*	-0.5466	0.0099
Xenorhabdus	*Erwinia*	0.5617	0.0099
Xenorhabdus	*Kluyvera*	0.4662	0.0099

Xenorhabdus	*Kosakonia*	0.3264	0.0396
Xenorhabdus	*Ligilactobacillus*	-0.3133	0.0495
Xenorhabdus	*Paraclostridium*	-0.4331	0.0198
Xenorhabdus	*Pectobacterium*	0.5049	0.0099
Xenorhabdus	*Providencia*	0.5304	0.0099
Xenorhabdus	*Serratia*	0.4406	0.0198
Xenorhabdus	*Vibrio*	0.4424	0.0099
Yersinia	*Budvicia*	0.3834	0.0396
Yersinia	*Carnobacterium*	-0.3969	0.0099
Yersinia	*Eubacterium*	0.419	0.0099
Yersinia	*Hafnia*	0.5751	0.0099
Yersinia	*Lactobacillus*	-0.3317	0.0495
Yersinia	*Raoultella*	0.4311	0.0099
Yersinia	*Vagococcus*	-0.349	0.0198
Yokenella	*Bacillus*	-0.4931	0.0099
Yokenella	*Cedecea*	0.6762	0.0099
Yokenella	*Citrobacter*	0.3335	0.0495
Yokenella	*Enterobacter*	0.5015	0.0099
Yokenella	*Escherichia*	0.3462	0.0396
Yokenella	*Franconibacter*	0.327	0.0198
Yokenella	*Granulicatella*	-0.3469	0.0198
Yokenella	*Klebsiella*	0.3911	0.0198
Yokenella	*Kosakonia*	0.3191	0.0495
Yokenella	*Kurthia*	-0.3475	0.0198
Yokenella	*Leclercia*	0.3624	0.0396
Yokenella	*Pasteurella*	-0.373	0.0396
Yokenella	*Pectobacterium*	0.4971	0.0099
Yokenella	*Pluralibacter*	0.3162	0.0495
Yokenella	*Pseudocitrobacter*	0.372	0.0297
Yokenella	*Raoultella*	0.355	0.0396
Yokenella	*Salmonella*	0.6827	0.0099
Yokenella	*Staphylococcus*	-0.4568	0.0198
Yokenella	*Streptococcus*	-0.5505	0.0099
Yokenella	*Trichococcus*	-0.4188	0.0198
Yokenella	*Weissella*	-0.432	0.0297
