# Supplementary material for: Gut microbiome community profiling of Bornean bats with different feeding guilds
Source: Anim Microbiome. 2025 Mar 5;7:21. doi: 10.1186/s42523-025-00389-w (PMC11881492; doi:10.1186/s42523-025-00389-w)
Supplement: Supplementary file 2 — Supplementary Material 2 [file 42523_2025_389_MOESM2_ESM.pdf]

Additional file 1

List of bacteria detected from sample set

Phylum	Family	*Genus*	*Species*
Acidobacteriota	Acidobacteriaceae	*Edaphobacter*	*Edaphobacter aggregans*
Acidobacteriota	Acidobacteriaceae	*Edaphobacter*	*Edaphobacter dinghuensis*
Acidobacteriota	Acidobacteriaceae	*Edaphobacter*	*Edaphobacter modestus*
Acidobacteriota	Acidobacteriaceae	*Granulicella*	*Granulicella arctica*
Acidobacteriota	Acidobacteriaceae	*Granulicella*	*Granulicella cerasi*
Acidobacteriota	Acidobacteriaceae	*Granulicella*	*Granulicella mallensis*
Acidobacteriota	Acidobacteriaceae	*Granulicella*	*Granulicella paludicola*
Acidobacteriota	Blastocatellaceae	*Tellurimicrobium*	*Tellurimicrobium multivorans*
Acidobacteriota	Acidobacteriaceae	*Terriglobus*	*Terriglobus aquaticus*
Acidobacteriota	Acidobacteriaceae	*Terriglobus*	*Terriglobus roseus*
Acidobacteriota	Vicinamibacteraceae	*Vicinamibacter*	*Vicinamibacter silvestris*
Actinomycetota	Acidimicrobiaceae	*Aciditerrimonas*	*Aciditerrimonas ferrireducens*
Actinomycetota	Pseudonocardiaceae	*Actinoalloteichus*	*Actinoalloteichus cyanogriseus*
Actinomycetota	Pseudonocardiaceae	*Actinoalloteichus*	*Actinoalloteichus hoggarensis*
Actinomycetota	Actinomycetaceae	*Actinomyces*	*Actinomyces graevenitzii*
Actinomycetota	Actinomycetaceae	*Actinomyces*	*Actinomyces marimammalium*
Actinomycetota	Actinomycetaceae	*Actinomyces*	*Actinomyces polynesiensis*
Actinomycetota	Actinomycetaceae	*Actinomyces*	*Actinomyces urogenitalis*
Actinomycetota	Pseudonocardiaceae	*Actinomycetospora*	*Actinomycetospora succinea*
Actinomycetota	Pseudonocardiaceae	*Actinophytocola*	*Actinophytocola xinjiangensis*
Actinomycetota	Eggerthellaceae	*Adlercreutzia*	*Adlercreutzia caecimuris*
Actinomycetota	Nocardioidaceae	*Aeromicrobium*	*Aeromicrobium erythreum*
Actinomycetota	Microbacteriaceae	*Agrococcus*	*Agrococcus terreus*
Actinomycetota	Microbacteriaceae	*Agromyces*	*Agromyces ramosus*

Actinomycetota	Microbacteriaceae	*Agromyces*	*Agromyces terreus*
Actinomycetota	Pseudonocardiaceae	*Allosaccharopolyspora*	*Allosaccharopolyspora coralli*
Actinomycetota	Microbacteriaceae	*Amnibacterium*	*Amnibacterium kyonggiense*
Actinomycetota	Pseudonocardiaceae	*Amycolatopsis*	*Amycolatopsis decaplanina*
Actinomycetota	Pseudonocardiaceae	*Amycolatopsis*	*Amycolatopsis magusensis*
Actinomycetota	Pseudonocardiaceae	*Amycolatopsis*	*Amycolatopsis methanolica*
Actinomycetota	Pseudonocardiaceae	*Amycolatopsis*	*Amycolatopsis sacchari*
Actinomycetota	Angustibacteraceae	*Angustibacter*	*Angustibacter speluncae*
Actinomycetota	Micrococcaceae	*Arthrobacter*	*Arthrobacter ginkgonis*
Actinomycetota	Micrococcaceae	*Arthrobacter*	*Arthrobacter pascens*
Actinomycetota	Micrococcaceae	*Arthrobacter*	*Arthrobacter pokkalii*
Actinomycetota	Micrococcaceae	*Arthrobacter*	*Arthrobacter psychrochitiniphilus*
Actinomycetota	Dermabacteraceae	*Brachybacterium*	*Brachybacterium conglomeratum*
Actinomycetota	Dermabacteraceae	*Brachybacterium*	*Brachybacterium hainanense*
Actinomycetota	Dermabacteraceae	*Brachybacterium*	*Brachybacterium huguangmaarense*
Actinomycetota	Dermabacteraceae	*Brachybacterium*	*Brachybacterium nesterenkovii*
Actinomycetota	Dermabacteraceae	*Brachybacterium*	*Brachybacterium paraconglomeratum*
Actinomycetota	Dermabacteraceae	*Brachybacterium*	*Brachybacterium phenoliresistens*
Actinomycetota	Dermabacteraceae	*Brachybacterium*	*Brachybacterium squillarum*
Actinomycetota	Brevibacteriaceae	*Brevibacterium*	*Brevibacterium frigoritolerans*
Actinomycetota	Brevibacteriaceae	*Brevibacterium*	*Brevibacterium oceani*
Actinomycetota	Brevibacteriaceae	*Brevibacterium*	*Brevibacterium sanguinis*
Actinomycetota	Brevibacteriaceae	*Brevibacterium*	*Brevibacterium sediminis*
Actinomycetota	Catenulisporaceae	*Catenulispora*	*Catenulispora fulva*
Actinomycetota	Cellulomonadaceae	*Cellulosimicrobium*	*Cellulosimicrobium cellulans*
Actinomycetota	Solirubrobacteraceae	*Conexibacter*	*Conexibacter arvalis*
Actinomycetota	Solirubrobacteraceae	*Conexibacter*	*Conexibacter stalactiti*
Actinomycetota	Solirubrobacteraceae	*Conexibacter*	*Conexibacter woesei*

Actinomycetota	Mycobacteriaceae	*Corynebacterium*	*Corynebacterium accolens*
Actinomycetota	Mycobacteriaceae	*Corynebacterium*	*Corynebacterium appendicis*
Actinomycetota	Mycobacteriaceae	*Corynebacterium*	*Corynebacterium atypicum*
Actinomycetota	Mycobacteriaceae	*Corynebacterium*	*Corynebacterium aurimucosum*
Actinomycetota	Mycobacteriaceae	*Corynebacterium*	*Corynebacterium bovis*
Actinomycetota	Mycobacteriaceae	*Corynebacterium*	*Corynebacterium caspium*
Actinomycetota	Mycobacteriaceae	*Corynebacterium*	*Corynebacterium confusum*
Actinomycetota	Mycobacteriaceae	*Corynebacterium*	*Corynebacterium cystitidis*
Actinomycetota	Mycobacteriaceae	*Corynebacterium*	*Corynebacterium efficiens*
Actinomycetota	Mycobacteriaceae	*Corynebacterium*	*Corynebacterium falsenii*
Actinomycetota	Mycobacteriaceae	*Corynebacterium*	*Corynebacterium flavescens*
Actinomycetota	Mycobacteriaceae	*Corynebacterium*	*Corynebacterium glyciniphilum*
Actinomycetota	Mycobacteriaceae	*Corynebacterium*	*Corynebacterium godavarianum*
Actinomycetota	Mycobacteriaceae	*Corynebacterium*	*Corynebacterium halotolerans*
Actinomycetota	Mycobacteriaceae	*Corynebacterium*	*Corynebacterium jeikeium*
Actinomycetota	Mycobacteriaceae	*Corynebacterium*	*Corynebacterium lipophiloflavum*
Actinomycetota	Mycobacteriaceae	*Corynebacterium*	*Corynebacterium macginleyi*
Actinomycetota	Mycobacteriaceae	*Corynebacterium*	*Corynebacterium massiliense*
Actinomycetota	Mycobacteriaceae	*Corynebacterium*	*Corynebacterium mastitidis*
Actinomycetota	Mycobacteriaceae	*Corynebacterium*	*Corynebacterium pilosum*
Actinomycetota	Mycobacteriaceae	*Corynebacterium*	*Corynebacterium tapiri*
Actinomycetota	Mycobacteriaceae	*Corynebacterium*	*Corynebacterium terpenotabidum*
Actinomycetota	Mycobacteriaceae	*Corynebacterium*	*Corynebacterium tuberculostearicum*
Actinomycetota	Mycobacteriaceae	*Corynebacterium*	*Corynebacterium urealyticum*
Actinomycetota	Mycobacteriaceae	*Corynebacterium*	*Corynebacterium variabile*
Actinomycetota	Microbacteriaceae	*Cryobacterium*	*Cryobacterium tepidiphilum*
Actinomycetota	Cryptosporangiaceae	*Cryptosporangium*	*Cryptosporangium eucalypti*
Actinomycetota	Microbacteriaceae	*Curtobacterium*	*Curtobacterium albidum*

Actinomycetota	Microbacteriaceae	*Curtobacterium*	*Curtobacterium citreum*
Actinomycetota	Microbacteriaceae	*Curtobacterium*	*Curtobacterium luteum*
Actinomycetota	Microbacteriaceae	*Curtobacterium*	*Curtobacterium oceanosedimentum*
Actinomycetota	Microbacteriaceae	*Curtobacterium*	*Curtobacterium plantarum*
Actinomycetota	Microbacteriaceae	*Curtobacterium*	*Curtobacterium pusillum*
Actinomycetota	Propionibacteriaceae	*Cutibacterium*	*Cutibacterium granulosum*
Actinomycetota	Dermatophilaceae	*Dermacoccus*	*Dermacoccus abyssi*
Actinomycetota	Mycobacteriaceae	*Dietzia*	*Dietzia timorensis*
Actinomycetota	Eggerthellaceae	*Eggerthella*	*Eggerthella timonensis*
Actinomycetota	Dermatophilaceae	*Flexivirga*	*Flexivirga alba*
Actinomycetota	Dermatophilaceae	*Flexivirga*	*Flexivirga endophytica*
Actinomycetota	Actinomycetaceae	*Fudania*	*Fudania jinshanensis*
Actinomycetota	Micrococcaceae	*Galactobacter*	*Galactobacter valiniphilus*
Actinomycetota	Mycobacteriaceae	*Gordonia*	*Gordonia alkaliphila*
Actinomycetota	Mycobacteriaceae	*Gordonia*	*Gordonia araii*
Actinomycetota	Mycobacteriaceae	*Gordonia*	*Gordonia bronchialis*
Actinomycetota	Mycobacteriaceae	*Gordonia*	*Gordonia effusa*
Actinomycetota	Mycobacteriaceae	*Gordonia*	*Gordonia hongkongensis*
Actinomycetota	Mycobacteriaceae	*Gordonia*	*Gordonia humi*
Actinomycetota	Mycobacteriaceae	*Gordonia*	*Gordonia iterans*
Actinomycetota	Mycobacteriaceae	*Gordonia*	*Gordonia kroppenstedtii*
Actinomycetota	Mycobacteriaceae	*Gordonia*	*Gordonia namibiensis*
Actinomycetota	Mycobacteriaceae	*Gordonia*	*Gordonia phthalatica*
Actinomycetota	Mycobacteriaceae	*Gordonia*	*Gordonia polyisoprenivorans*
Actinomycetota	Mycobacteriaceae	*Gordonia*	*Gordonia rubripertincta*
Actinomycetota	Mycobacteriaceae	*Gordonia*	*Gordonia shandongensis*
Actinomycetota	Mycobacteriaceae	*Gordonia*	*Gordonia soli*
Actinomycetota	Microbacteriaceae	*Gulosibacter*	*Gulosibacter bifidus*

Actinomycetota	Microbacteriaceae	*Gulosibacter*	*Gulosibacter chungangensis*
Actinomycetota	Microbacteriaceae	*Herbiconiux*	*Herbiconiux ginsengi*
Actinomycetota	Microbacteriaceae	*Humibacter*	*Humibacter albus*
Actinomycetota	Microbacteriaceae	*Humibacter*	*Humibacter soli*
Actinomycetota	Ilumatobacteraceae	*Ilumatobacter*	*Ilumatobacter fluminis*
Actinomycetota	Ilumatobacteraceae	*Ilumatobacter*	*Ilumatobacter nonamiensis*
Actinomycetota	Dermatophilaceae	*Intrasporangium*	*Intrasporangium chromatireducens*
Actinomycetota	Cellulomonadaceae	*Isoptericola*	*Isoptericola cucumis*
Actinomycetota	Dermatophilaceae	*Janibacter*	*Janibacter indicus*
Actinomycetota	Jatrophihabitantaceae	*Jatrophihabitans*	*Jatrophihabitans endophyticus*
Actinomycetota	Jatrophihabitantaceae	*Jatrophihabitans*	*Jatrophihabitans soli*
Actinomycetota	Streptomycetaceae	*Kitasatospora*	*Kitasatospora atroaurantiaca*
Actinomycetota	Streptomycetaceae	*Kitasatospora*	*Kitasatospora cystarginea*
Actinomycetota	Streptomycetaceae	*Kitasatospora*	*Kitasatospora griseola*
Actinomycetota	Streptomycetaceae	*Kitasatospora*	*Kitasatospora setae*
Actinomycetota	Streptomycetaceae	*Kitasatospora*	*Kitasatospora terrestris*
Actinomycetota	Dermatophilaceae	*Knoellia*	*Knoellia remsis*
Actinomycetota	Micrococcaceae	*Kocuria*	*Kocuria dechangensis*
Actinomycetota	Micrococcaceae	*Kocuria*	*Kocuria indica*
Actinomycetota	Micrococcaceae	*Kocuria*	*Kocuria koreensis*
Actinomycetota	Micrococcaceae	*Kocuria*	*Kocuria rosea*
Actinomycetota	Dermatophilaceae	*Kytococcus*	*Kytococcus sedentarius*
Actinomycetota	Microbacteriaceae	*Leifsonia*	*Leifsonia lichenia*
Actinomycetota	Microbacteriaceae	*Leifsonia*	*Leifsonia naganoensis*
Actinomycetota	Microbacteriaceae	*Leifsonia*	*Leifsonia poae*
Actinomycetota	Microbacteriaceae	*Leifsonia*	*Leifsonia shinshuensis*
Actinomycetota	Microbacteriaceae	*Leifsonia*	*Leifsonia xyli*
Actinomycetota	Microbacteriaceae	*Leucobacter*	*Leucobacter aridicollis*

Actinomycetota	Microbacteriaceae	*Leucobacter*	*Leucobacter chromiiresistens*
Actinomycetota	Microbacteriaceae	*Leucobacter*	*Leucobacter populi*
Actinomycetota	Microbacteriaceae	*Leucobacter*	*Leucobacter tardus*
Actinomycetota	Cellulomonadaceae	*Luteimicrobium*	*Luteimicrobium album*
Actinomycetota	Nocardioidaceae	*Marmoricola*	*Marmoricola pocheonensis*
Actinomycetota	Microbacteriaceae	*Microbacterium*	*Microbacterium arabinogalactanolyticum*
Actinomycetota	Microbacteriaceae	*Microbacterium*	*Microbacterium aureliae*
Actinomycetota	Microbacteriaceae	*Microbacterium*	*Microbacterium barkeri*
Actinomycetota	Microbacteriaceae	*Microbacterium*	*Microbacterium deminutum*
Actinomycetota	Microbacteriaceae	*Microbacterium*	*Microbacterium deserti*
Actinomycetota	Microbacteriaceae	*Microbacterium*	*Microbacterium esteraromaticum*
Actinomycetota	Microbacteriaceae	*Microbacterium*	*Microbacterium foliorum*
Actinomycetota	Microbacteriaceae	*Microbacterium*	*Microbacterium ginsengisoli*
Actinomycetota	Microbacteriaceae	*Microbacterium*	*Microbacterium hatanonis*
Actinomycetota	Microbacteriaceae	*Microbacterium*	*Microbacterium hominis*
Actinomycetota	Microbacteriaceae	*Microbacterium*	*Microbacterium invictum*
Actinomycetota	Microbacteriaceae	*Microbacterium*	*Microbacterium jejuense*
Actinomycetota	Microbacteriaceae	*Microbacterium*	*Microbacterium kyungheense*
Actinomycetota	Microbacteriaceae	*Microbacterium*	*Microbacterium lacus*
Actinomycetota	Microbacteriaceae	*Microbacterium*	*Microbacterium marinum*
Actinomycetota	Microbacteriaceae	*Microbacterium*	*Microbacterium neimengense*
Actinomycetota	Microbacteriaceae	*Microbacterium*	*Microbacterium paraoxydans*
Actinomycetota	Microbacteriaceae	*Microbacterium*	*Microbacterium phyllosphaerae*
Actinomycetota	Microbacteriaceae	*Microbacterium*	*Microbacterium pseudoresistens*
Actinomycetota	Microbacteriaceae	*Microbacterium*	*Microbacterium saccharophilum*
Actinomycetota	Microbacteriaceae	*Microbacterium*	*Microbacterium schleiferi*
Actinomycetota	Microbacteriaceae	*Microbacterium*	*Microbacterium suaedae*
Actinomycetota	Microbacteriaceae	*Microbacterium*	*Microbacterium terregens*

Actinomycetota	Microbacteriaceae	*Microbacterium*	*Microbacterium trichothecenolyticum*
Actinomycetota	Microbacteriaceae	*Microbacterium*	*Microbacterium ulmi*
Actinomycetota	Microbacteriaceae	*Microbacterium*	*Microbacterium xylanilyticum*
Actinomycetota	Microbacteriaceae	*Microbacterium*	*Microbacterium yannicii*
Actinomycetota	Micrococcaceae	*Micrococcus*	*Micrococcus luteus*
Actinomycetota	Propionibacteriaceae	*Microlunatus*	*Microlunatus aurantiacus*
Actinomycetota	Propionibacteriaceae	*Microlunatus*	*Microlunatus ginsengisoli*
Actinomycetota	Propionibacteriaceae	*Microlunatus*	*Microlunatus phosphovorus*
Actinomycetota	Micromonosporaceae	*Micromonospora*	*Micromonospora pattaloongensis*
Actinomycetota	Propionibacteriaceae	*Micropruina*	*Micropruina glycogenica*
Actinomycetota	Mycobacteriaceae	*Mycobacterium*	*Mycobacterium asiaticum*
Actinomycetota	Mycobacteriaceae	*Mycobacterium*	*Mycobacterium chelonae*
Actinomycetota	Mycobacteriaceae	*Mycobacterium*	*Mycobacterium cookii*
Actinomycetota	Mycobacteriaceae	*Mycobacterium*	*Mycobacterium haemophilum*
Actinomycetota	Mycobacteriaceae	*Mycobacterium*	*Mycobacterium palauense*
Actinomycetota	Mycobacteriaceae	*Mycobacterium*	*Mycobacterium porcinum*
Actinomycetota	Mycobacteriaceae	*Mycobacteroides*	*Mycobacteroides abscessus*
Actinomycetota	Mycobacteriaceae	*Mycobacteroides*	*Mycobacteroides immunogenum*
Actinomycetota	Mycobacteriaceae	*Mycolicibacterium*	*Mycolicibacterium fortuitum*
Actinomycetota	Mycobacteriaceae	*Mycolicibacterium*	*Mycolicibacterium gilvum*
Actinomycetota	Mycobacteriaceae	*Mycolicibacterium*	*Mycolicibacterium insubricum*
Actinomycetota	Mycobacteriaceae	*Mycolicibacterium*	*Mycolicibacterium madagascariense*
Actinomycetota	Mycobacteriaceae	*Mycolicibacterium*	*Mycolicibacterium mageritense*
Actinomycetota	Mycobacteriaceae	*Mycolicibacterium*	*Mycolicibacterium mucogenicum;Mycolicibacterium*
Actinomycetota	Mycobacteriaceae	*Mycolicibacterium*	*Mycolicibacterium murale*
Actinomycetota	Mycobacteriaceae	*Mycolicibacterium*	*Mycolicibacterium neoaurum*
Actinomycetota	Mycobacteriaceae	*Mycolicibacterium*	*Mycolicibacterium parafortuitum*

Actinomycetota	Mycobacteriaceae	*Mycolicibacterium*	*Mycolicibacterium psychrotolerans*
Actinomycetota	Mycobacteriaceae	*Mycolicibacterium*	*Mycolicibacterium smegmatis*
Actinomycetota	Mycobacteriaceae	*Mycolicibacterium*	*Mycolicibacterium sphagni*
Actinomycetota	Mycobacteriaceae	*Mycolicibacterium*	*Mycolicibacterium wolinskyi*
Actinomycetota	Nakamurellaceae	*Nakamurella*	*Nakamurella flavida*
Actinomycetota	Nakamurellaceae	*Nakamurella*	*Nakamurella lactea*
Actinomycetota	Nakamurellaceae	*Nakamurella*	*Nakamurella multipartita*
Actinomycetota	Propionibacteriaceae	*Naumannella*	*Naumannella huperziae*
Actinomycetota	Micrococcaceae	*Nesterenkonia*	*Nesterenkonia alba*
Actinomycetota	Micrococcaceae	*Nesterenkonia*	*Nesterenkonia cremea*
Actinomycetota	Micrococcaceae	*Nesterenkonia*	*Nesterenkonia lacusekhoensis*
Actinomycetota	Micrococcaceae	*Nesterenkonia*	*Nesterenkonia suensis*
Actinomycetota	Mycobacteriaceae	*Nocardia*	*Nocardia aobensis*
Actinomycetota	Mycobacteriaceae	*Nocardia*	*Nocardia artemisiae*
Actinomycetota	Mycobacteriaceae	*Nocardia*	*Nocardia carnea*
Actinomycetota	Mycobacteriaceae	*Nocardia*	*Nocardia exalbida*
Actinomycetota	Mycobacteriaceae	*Nocardia*	*Nocardia gamkensis*
Actinomycetota	Mycobacteriaceae	*Nocardia*	*Nocardia higoensis*
Actinomycetota	Mycobacteriaceae	*Nocardia*	*Nocardia kroppenstedtii*
Actinomycetota	Mycobacteriaceae	*Nocardia*	*Nocardia lijiangensis*
Actinomycetota	Mycobacteriaceae	*Nocardia*	*Nocardia paucivorans*
Actinomycetota	Mycobacteriaceae	*Nocardia*	*Nocardia pneumoniae*
Actinomycetota	Mycobacteriaceae	*Nocardia*	*Nocardia rayongensis*
Actinomycetota	Mycobacteriaceae	*Nocardia*	*Nocardia terpenica*
Actinomycetota	Mycobacteriaceae	*Nocardia*	*Nocardia vaccinii*
Actinomycetota	Mycobacteriaceae	*Nocardia*	*Nocardia wallacei*
Actinomycetota	Nocardioidaceae	*Nocardioides*	*Nocardioides bigeumensis*
Actinomycetota	Nocardioidaceae	*Nocardioides*	*Nocardioides cavernae*

Actinomycetota	Nocardioidaceae	*Nocardioides*	*Nocardioides daecheongensis*
Actinomycetota	Nocardioidaceae	*Nocardioides*	*Nocardioides daedukensis*
Actinomycetota	Nocardioidaceae	*Nocardioides*	*Nocardioides dubius*
Actinomycetota	Nocardioidaceae	*Nocardioides*	*Nocardioides humi*
Actinomycetota	Nocardioidaceae	*Nocardioides*	*Nocardioides immobilis*
Actinomycetota	Nocardioidaceae	*Nocardioides*	*Nocardioides islandensis*
Actinomycetota	Nocardioidaceae	*Nocardioides*	*Nocardioides kribbensis*
Actinomycetota	Nocardioidaceae	*Nocardioides*	*Nocardioides lianchengensis*
Actinomycetota	Nocardioidaceae	*Nocardioides*	*Nocardioides marinquilinus*
Actinomycetota	Nocardioidaceae	*Nocardioides*	*Nocardioides panacihumi*
Actinomycetota	Nocardioidaceae	*Nocardioides*	*Nocardioides panacisoli*
Actinomycetota	Nocardioidaceae	*Nocardioides*	*Nocardioides soli*
Actinomycetota	Nocardioidaceae	*Nocardioides*	*Nocardioides taihuensis*
Actinomycetota	Nocardioidaceae	*Nocardioides*	*Nocardioides tritolerans*
Actinomycetota	Streptosporangiaceae	*Nonomuraea*	*Nonomuraea roseola*
Actinomycetota	Atopobiaceae	*Olegusella*	*Olegusella massiliensis*
Actinomycetota	Atopobiaceae	*Olsenella*	*Olsenella profusa*
Actinomycetota	Micrococcaceae	*Paenarthrobacter*	*Paenarthrobacter nicotinovorans*
Actinomycetota	Parviterribacteraceae	*Parviterribacter*	*Parviterribacter kavangonensis*
Actinomycetota	Solirubrobacteraceae	*Patulibacter*	*Patulibacter americanus*
Actinomycetota	Solirubrobacteraceae	*Patulibacter*	*Patulibacter brassicae*
Actinomycetota	Solirubrobacteraceae	*Patulibacter*	*Patulibacter ginsengiterrae*
Actinomycetota	Solirubrobacteraceae	*Patulibacter*	*Patulibacter minatonensis*
Actinomycetota	Micromonosporaceae	*Pilimelia*	*Pilimelia columellifera*
Actinomycetota	Cellulomonadaceae	*Promicromonospora*	*Promicromonospora alba*
Actinomycetota	Cellulomonadaceae	*Promicromonospora*	*Promicromonospora viridis*
Actinomycetota	Propionibacteriaceae	*Propionibacterium*	*Propionibacterium acidifaciens*
Actinomycetota	Propionibacteriaceae	*Propionibacterium*	*Propionibacterium cyclohexanicum*

Actinomycetota	Propionibacteriaceae	*Propionibacterium*	*Propionibacterium freudenreichii*
Actinomycetota	Microbacteriaceae	*Protaetiibacter*	*Protaetiibacter larvae*
Actinomycetota	Micrococcaceae	*Pseudarthrobacter*	*Pseudarthrobacter chlorophenolicus*
Actinomycetota	Micrococcaceae	*Pseudarthrobacter*	*Pseudarthrobacter defluvii*
Actinomycetota	Microbacteriaceae	*Pseudoclavibacter*	*Pseudoclavibacter chungangensis*
Actinomycetota	Microbacteriaceae	*Pseudoclavibacter*	*Pseudoclavibacter endophyticus*
Actinomycetota	Microbacteriaceae	*Pseudoclavibacter*	*Pseudoclavibacter helvolus*
Actinomycetota	Micrococcaceae	*Pseudoglutamicibacter*	*Pseudoglutamicibacter cumminsii*
Actinomycetota	Microbacteriaceae	*Pseudolysinimonas*	*Pseudolysinimonas kribbensis*
Actinomycetota	Pseudonocardiaceae	*Pseudonocardia*	*Pseudonocardia alaniniphila*
Actinomycetota	Pseudonocardiaceae	*Pseudonocardia*	*Pseudonocardia asaccharolytica*
Actinomycetota	Pseudonocardiaceae	*Pseudonocardia*	*Pseudonocardia carboxydivorans*
Actinomycetota	Pseudonocardiaceae	*Pseudonocardia*	*Pseudonocardia chloroethenivorans*
Actinomycetota	Pseudonocardiaceae	*Pseudonocardia*	*Pseudonocardia hydrocarbonoxydans*
Actinomycetota	Pseudonocardiaceae	*Pseudonocardia*	*Pseudonocardia nigra*
Actinomycetota	Pseudonocardiaceae	*Pseudonocardia*	*Pseudonocardia seranimata*
Actinomycetota	Pseudonocardiaceae	*Pseudonocardia*	*Pseudonocardia spinosa*
Actinomycetota	Pseudonocardiaceae	*Pseudonocardia*	*Pseudonocardia xinjiangensis*
Actinomycetota	Quadrisphaeraceae	*Quadrisphaera*	*Quadrisphaera granulorum*
Actinomycetota	Eggerthellaceae	*Raoultibacter*	*Raoultibacter massiliensis*
Actinomycetota	Eggerthellaceae	*Raoultibacter*	*Raoultibacter timonensis*
Actinomycetota	Microbacteriaceae	*Rathayibacter*	*Rathayibacter rathayi*
Actinomycetota	Microbacteriaceae	*Rathayibacter*	*Rathayibacter tritici*
Actinomycetota	Iamiaceae	*Rhabdothermincola*	*Rhabdothermincola sediminis*
Actinomycetota	Mycobacteriaceae	*Rhodococcus*	*Rhodococcus agglutinans*
Actinomycetota	Mycobacteriaceae	*Rhodococcus*	*Rhodococcus hoagii*
Actinomycetota	Mycobacteriaceae	*Rhodococcus*	*Rhodococcus rhodnii*
Actinomycetota	Mycobacteriaceae	*Rhodococcus*	*Rhodococcus rhodochrous*

Actinomycetota	Micrococcaceae	*Rothia*	*Rothia amarae*
Actinomycetota	Micrococcaceae	*Rothia*	*Rothia endophytica*
Actinomycetota	Micrococcaceae	*Rothia*	*Rothia koreensis*
Actinomycetota	Micrococcaceae	*Rothia*	*Rothia kristinae*
Actinomycetota	Rubrobacteraceae	*Rubrobacter*	*Rubrobacter radiotolerans*
Actinomycetota	Rubrobacteraceae	*Rubrobacter*	*Rubrobacter spartanus*
Actinomycetota	Rubrobacteraceae	*Rubrobacter*	*Rubrobacter taiwanensis*
Actinomycetota	Rubrobacteraceae	*Rubrobacter*	*Rubrobacter xylanophilus*
Actinomycetota	Pseudonocardiaceae	*Saccharomonospora*	*Saccharomonospora viridis*
Actinomycetota	Pseudonocardiaceae	*Saccharopolyspora*	*Saccharopolyspora cavernae*
Actinomycetota	Pseudonocardiaceae	*Saccharopolyspora*	*Saccharopolyspora cebuensis*
Actinomycetota	Pseudonocardiaceae	*Saccharopolyspora*	*Saccharopolyspora erythraea*
Actinomycetota	Pseudonocardiaceae	*Saccharopolyspora*	*Saccharopolyspora gloriosae*
Actinomycetota	Pseudonocardiaceae	*Saccharopolyspora*	*Saccharopolyspora gregorii*
Actinomycetota	Pseudonocardiaceae	*Saccharopolyspora*	*Saccharopolyspora halophila*
Actinomycetota	Pseudonocardiaceae	*Saccharopolyspora*	*Saccharopolyspora halotolerans*
Actinomycetota	Pseudonocardiaceae	*Saccharopolyspora*	*Saccharopolyspora hordei*
Actinomycetota	Pseudonocardiaceae	*Saccharopolyspora*	*Saccharopolyspora qijiaojingensis*
Actinomycetota	Pseudonocardiaceae	*Saccharopolyspora*	*Saccharopolyspora rectivirgula*
Actinomycetota	Pseudonocardiaceae	*Saccharopolyspora*	*Saccharopolyspora spongiae*
Actinomycetota	Pseudonocardiaceae	*Saccharothrix*	*Saccharothrix ecbatanensis*
Actinomycetota	Pseudonocardiaceae	*Salinifilum*	*Salinifilum aidingensis*
Actinomycetota	Actinomycetaceae	*Schaalia*	*Schaalia canis*
Actinomycetota	Actinomycetaceae	*Schaalia*	*Schaalia cardiffensis*
Actinomycetota	Actinomycetaceae	*Schaalia*	*Schaalia hyovaginalis*
Actinomycetota	Actinomycetaceae	*Schaalia*	*Schaalia meyeri*
Actinomycetota	Actinomycetaceae	*Schaalia*	*Schaalia naturae*
Actinomycetota	Actinomycetaceae	*Schaalia*	*Schaalia odontolytica*

Actinomycetota	Actinomycetaceae	*Schaalia*	*Schaalia suimastitidis*
Actinomycetota	Actinomycetaceae	*Schaalia*	*Schaalia vaccimaxillae*
Actinomycetota	Micrococcaceae	*Sinomonas*	*Sinomonas halotolerans*
Actinomycetota	Mycobacteriaceae	*Skermania*	*Skermania piniformis*
Actinomycetota	Solirubrobacteraceae	*Solirubrobacter*	*Solirubrobacter ginsenosidimutans*
Actinomycetota	Solirubrobacteraceae	*Solirubrobacter*	*Solirubrobacter soli*
Actinomycetota	Streptomycetaceae	*Streptacidiphilus*	*Streptacidiphilus carbonis*
Actinomycetota	Streptomycetaceae	*Streptomyces*	*Streptomyces abikoensis*
Actinomycetota	Streptomycetaceae	*Streptomyces*	*Streptomyces acidiscabies*
Actinomycetota	Streptomycetaceae	*Streptomyces*	*Streptomyces adustus*
Actinomycetota	Streptomycetaceae	*Streptomyces*	*Streptomyces aegyptia*
Actinomycetota	Streptomycetaceae	*Streptomyces*	*Streptomyces albiaxialis*
Actinomycetota	Streptomycetaceae	*Streptomyces*	*Streptomyces albosporeus*
Actinomycetota	Streptomycetaceae	*Streptomyces*	*Streptomyces albulus*
Actinomycetota	Streptomycetaceae	*Streptomyces*	*Streptomyces anulatus*
Actinomycetota	Streptomycetaceae	*Streptomyces*	*Streptomyces atratus*
Actinomycetota	Streptomycetaceae	*Streptomyces*	*Streptomyces atriruber*
Actinomycetota	Streptomycetaceae	*Streptomyces*	*Streptomyces aurantiacus*
Actinomycetota	Streptomycetaceae	*Streptomyces*	*Streptomyces avidinii*
Actinomycetota	Streptomycetaceae	*Streptomyces*	*Streptomyces axinellae*
Actinomycetota	Streptomycetaceae	*Streptomyces*	*Streptomyces azureus*
Actinomycetota	Streptomycetaceae	*Streptomyces*	*Streptomyces bacillaris*
Actinomycetota	Streptomycetaceae	*Streptomyces*	*Streptomyces badius*
Actinomycetota	Streptomycetaceae	*Streptomyces*	*Streptomyces baliensis*
Actinomycetota	Streptomycetaceae	*Streptomyces*	*Streptomyces beijiangensis*
Actinomycetota	Streptomycetaceae	*Streptomyces*	*Streptomyces bikiniensis*
Actinomycetota	Streptomycetaceae	*Streptomyces*	*Streptomyces bobili*
Actinomycetota	Streptomycetaceae	*Streptomyces*	*Streptomyces bryophytorum*

Actinomycetota	Streptomycetaceae	*Streptomyces*	*Streptomyces burgazadensis*
Actinomycetota	Streptomycetaceae	*Streptomyces*	*Streptomyces californicus*
Actinomycetota	Streptomycetaceae	*Streptomyces*	*Streptomyces cameroonensis*
Actinomycetota	Streptomycetaceae	*Streptomyces*	*Streptomyces camponoticapitis*
Actinomycetota	Streptomycetaceae	*Streptomyces*	*Streptomyces candidus*
Actinomycetota	Streptomycetaceae	*Streptomyces*	*Streptomyces canescens*
Actinomycetota	Streptomycetaceae	*Streptomyces*	*Streptomyces carminius*
Actinomycetota	Streptomycetaceae	*Streptomyces*	*Streptomyces cavourensis*
Actinomycetota	Streptomycetaceae	*Streptomyces*	*Streptomyces celluloflavus*
Actinomycetota	Streptomycetaceae	*Streptomyces*	*Streptomyces champavatii*
Actinomycetota	Streptomycetaceae	*Streptomyces*	*Streptomyces cinereus*
Actinomycetota	Streptomycetaceae	*Streptomyces*	*Streptomyces cinnabarigriseus*
Actinomycetota	Streptomycetaceae	*Streptomyces*	*Streptomyces cinnamoneus*
Actinomycetota	Streptomycetaceae	*Streptomyces*	*Streptomyces cirratus*
Actinomycetota	Streptomycetaceae	*Streptomyces*	*Streptomyces clavifer*
Actinomycetota	Streptomycetaceae	*Streptomyces*	*Streptomyces clavuligerus*
Actinomycetota	Streptomycetaceae	*Streptomyces*	*Streptomyces coacervatus*
Actinomycetota	Streptomycetaceae	*Streptomyces*	*Streptomyces curacoi*
Actinomycetota	Streptomycetaceae	*Streptomyces*	*Streptomyces cyaneus*
Actinomycetota	Streptomycetaceae	*Streptomyces*	*Streptomyces drozdowiczii*
Actinomycetota	Streptomycetaceae	*Streptomyces*	*Streptomyces enissocaesilis*
Actinomycetota	Streptomycetaceae	*Streptomyces*	*Streptomyces finlayi*
Actinomycetota	Streptomycetaceae	*Streptomyces*	*Streptomyces flavidovirens*
Actinomycetota	Streptomycetaceae	*Streptomyces*	*Streptomyces flavofuscus*
Actinomycetota	Streptomycetaceae	*Streptomyces*	*Streptomyces flavovariabilis*
Actinomycetota	Streptomycetaceae	*Streptomyces*	*Streptomyces formicae*
Actinomycetota	Streptomycetaceae	*Streptomyces*	*Streptomyces fradiae*
Actinomycetota	Streptomycetaceae	*Streptomyces*	*Streptomyces fukangensis*

Actinomycetota	Streptomycetaceae	*Streptomyces*	*Streptomyces fulvissimus*
Actinomycetota	Streptomycetaceae	*Streptomyces*	*Streptomyces fulvorobeus*
Actinomycetota	Streptomycetaceae	*Streptomyces*	*Streptomyces fuscichromogenes*
Actinomycetota	Streptomycetaceae	*Streptomyces*	*Streptomyces galbus*
Actinomycetota	Streptomycetaceae	*Streptomyces*	*Streptomyces galilaeus*
Actinomycetota	Streptomycetaceae	*Streptomyces*	*Streptomyces gardneri*
Actinomycetota	Streptomycetaceae	*Streptomyces*	*Streptomyces gelaticus*
Actinomycetota	Streptomycetaceae	*Streptomyces*	*Streptomyces glauciniger*
Actinomycetota	Streptomycetaceae	*Streptomyces*	*Streptomyces globisporus*
Actinomycetota	Streptomycetaceae	*Streptomyces*	*Streptomyces globosus*
Actinomycetota	Streptomycetaceae	*Streptomyces*	*Streptomyces graminisoli*
Actinomycetota	Streptomycetaceae	*Streptomyces*	*Streptomyces graminofaciens*
Actinomycetota	Streptomycetaceae	*Streptomyces*	*Streptomyces griseoruber*
Actinomycetota	Streptomycetaceae	*Streptomyces*	*Streptomyces hiroshimensis*
Actinomycetota	Streptomycetaceae	*Streptomyces*	*Streptomyces hygroscopicus*
Actinomycetota	Streptomycetaceae	*Streptomyces*	*Streptomyces intermedius*
Actinomycetota	Streptomycetaceae	*Streptomyces*	*Streptomyces inusitatus*
Actinomycetota	Streptomycetaceae	*Streptomyces*	*Streptomyces kasugaensis*
Actinomycetota	Streptomycetaceae	*Streptomyces*	*Streptomyces katrae*
Actinomycetota	Streptomycetaceae	*Streptomyces*	*Streptomyces laurentii*
Actinomycetota	Streptomycetaceae	*Streptomyces*	*Streptomyces lavendulae*
Actinomycetota	Streptomycetaceae	*Streptomyces*	*Streptomyces lilacinus*
Actinomycetota	Streptomycetaceae	*Streptomyces*	*Streptomyces litmocidini*
Actinomycetota	Streptomycetaceae	*Streptomyces*	*Streptomyces lushanensis*
Actinomycetota	Streptomycetaceae	*Streptomyces*	*Streptomyces luteogriseus*
Actinomycetota	Streptomycetaceae	*Streptomyces*	*Streptomyces luteosporeus*
Actinomycetota	Streptomycetaceae	*Streptomyces*	*Streptomyces maoxianensis*
Actinomycetota	Streptomycetaceae	*Streptomyces*	*Streptomyces melanogenes*

Actinomycetota	Streptomycetaceae	*Streptomyces*	*Streptomyces mobaraensis*
Actinomycetota	Streptomycetaceae	*Streptomyces*	*Streptomyces narbonensis*
Actinomycetota	Streptomycetaceae	*Streptomyces*	*Streptomyces netropsis*
Actinomycetota	Streptomycetaceae	*Streptomyces*	*Streptomyces nitrosporeus*
Actinomycetota	Streptomycetaceae	*Streptomyces*	*Streptomyces niveus*
Actinomycetota	Streptomycetaceae	*Streptomyces*	*Streptomyces oceani*
Actinomycetota	Streptomycetaceae	*Streptomyces*	*Streptomyces odonnellii*
Actinomycetota	Streptomycetaceae	*Streptomyces*	*Streptomyces olivoreticuli*
Actinomycetota	Streptomycetaceae	*Streptomyces*	*Streptomyces ovatisporus*
Actinomycetota	Streptomycetaceae	*Streptomyces*	*Streptomyces parvus*
Actinomycetota	Streptomycetaceae	*Streptomyces*	*Streptomyces peucetius*
Actinomycetota	Streptomycetaceae	*Streptomyces*	*Streptomyces plicatus*
Actinomycetota	Streptomycetaceae	*Streptomyces*	*Streptomyces plumbiresistens*
Actinomycetota	Streptomycetaceae	*Streptomyces*	*Streptomyces pluricolorescens*
Actinomycetota	Streptomycetaceae	*Streptomyces*	*Streptomyces polyrhachis*
Actinomycetota	Streptomycetaceae	*Streptomyces*	*Streptomyces pratensis*
Actinomycetota	Streptomycetaceae	*Streptomyces*	*Streptomyces pseudoechinosporeus*
Actinomycetota	Streptomycetaceae	*Streptomyces*	*Streptomyces pulveraceus*
Actinomycetota	Streptomycetaceae	*Streptomyces*	*Streptomyces purpureus*
Actinomycetota	Streptomycetaceae	*Streptomyces*	*Streptomyces racemochromogenes*
Actinomycetota	Streptomycetaceae	*Streptomyces*	*Streptomyces rectiverticillatus*
Actinomycetota	Streptomycetaceae	*Streptomyces*	*Streptomyces rhizosphaerihabitans*
Actinomycetota	Streptomycetaceae	*Streptomyces*	*Streptomyces rishiriensis*
Actinomycetota	Streptomycetaceae	*Streptomyces*	*Streptomyces rubiginosohelvolus*
Actinomycetota	Streptomycetaceae	*Streptomyces*	*Streptomyces sampsonii*
Actinomycetota	Streptomycetaceae	*Streptomyces*	*Streptomyces sanglieri*
Actinomycetota	Streptomycetaceae	*Streptomyces*	*Streptomyces scopuliridis*
Actinomycetota	Streptomycetaceae	*Streptomyces*	*Streptomyces seoulensis*

Actinomycetota	Streptomycetaceae	*Streptomyces*	*Streptomyces septatus*
Actinomycetota	Streptomycetaceae	*Streptomyces*	*Streptomyces sparsus*
Actinomycetota	Streptomycetaceae	*Streptomyces*	*Streptomyces spiralis*
Actinomycetota	Streptomycetaceae	*Streptomyces*	*Streptomyces sporoverrucosus*
Actinomycetota	Streptomycetaceae	*Streptomyces*	*Streptomyces stramineus*
Actinomycetota	Streptomycetaceae	*Streptomyces*	*Streptomyces subrutilus*
Actinomycetota	Streptomycetaceae	*Streptomyces*	*Streptomyces sudanensis*
Actinomycetota	Streptomycetaceae	*Streptomyces*	*Streptomyces sundarbansensis*
Actinomycetota	Streptomycetaceae	*Streptomyces*	*Streptomyces syringium*
Actinomycetota	Streptomycetaceae	*Streptomyces*	*Streptomyces tanashiensis*
Actinomycetota	Streptomycetaceae	*Streptomyces*	*Streptomyces termitum*
Actinomycetota	Streptomycetaceae	*Streptomyces*	*Streptomyces vastus*
Actinomycetota	Streptomycetaceae	*Streptomyces*	*Streptomyces venezuelae*
Actinomycetota	Streptomycetaceae	*Streptomyces*	*Streptomyces violaceorectus*
Actinomycetota	Streptomycetaceae	*Streptomyces*	*Streptomyces virginiae*
Actinomycetota	Streptomycetaceae	*Streptomyces*	*Streptomyces werraensis*
Actinomycetota	Streptomycetaceae	*Streptomyces*	*Streptomyces xanthochromogenes*
Actinomycetota	Streptomycetaceae	*Streptomyces*	*Streptomyces xiaopingdaonensis*
Actinomycetota	Streptomycetaceae	*Streptomyces*	*Streptomyces yangpuensis*
Actinomycetota	Streptomycetaceae	*Streptomyces*	*Streptomyces yatensis*
Actinomycetota	Streptomycetaceae	*Streptomyces*	*Streptomyces yerevanensis*
Actinomycetota	Streptomycetaceae	*Streptomyces*	*Streptomyces yunnanensis*
Actinomycetota	Streptomycetaceae	*Streptomyces*	*Streptomyces zagrosensis*
Actinomycetota	Streptomycetaceae	*Streptomyces*	*Streptomyces zaomyceticus*
Actinomycetota	Streptosporangiaceae	*Streptosporangium*	*Streptosporangium nanhuense*
Actinomycetota	Dermatophilaceae	*Terrabacter*	*Terrabacter carboxydivorans*
Actinomycetota	Dermatophilaceae	*Terrabacter*	*Terrabacter ginsenosidimutans*
Actinomycetota	Dermatophilaceae	*Terrabacter*	*Terrabacter terrigena*

Actinomycetota	Propionibacteriaceae	*Tessaracoccus*	*Tessaracoccus aquimaris*
Actinomycetota	Propionibacteriaceae	*Tessaracoccus*	*Tessaracoccus defluvii*
Actinomycetota	Propionibacteriaceae	*Tessaracoccus*	*Tessaracoccus oleiagri*
Actinomycetota	Propionibacteriaceae	*Tessaracoccus*	*Tessaracoccus rhinocerotis*
Actinomycetota	Dermatophilaceae	*Tetrasphaera*	*Tetrasphaera duodecadis*
Actinomycetota	Dermatophilaceae	*Tetrasphaera*	*Tetrasphaera elongata*
Actinomycetota	Dermatophilaceae	*Tetrasphaera*	*Tetrasphaera japonica*
Actinomycetota	Thermoleophilaceae	*Thermoleophilum*	*Thermoleophilum minutum*
Actinomycetota	Mycobacteriaceae	*Williamsia*	*Williamsia serinedens*
Actinomycetota	Promicromonosporaceae	*Xylanimicrobium*	*Xylanimicrobium pachnodae*
Actinomycetota	Micrococcaceae	*Yaniella*	*Yaniella flava*
Aquificota	Desulfurobacteriaceae	*Desulfurobacterium*	*Desulfurobacterium indicum*
Armatimonadota	Fimbriimonadaceae	*Fimbriimonas*	*Fimbriimonas ginsengisoli*
Bacillota	Aerococcaceae	*Abiotrophia*	*Abiotrophia defectiva*
Bacillota	Staphylococcaceae	*Abyssicoccus*	*Abyssicoccus albus*
Bacillota	Lachnospiraceae	*Abyssivirga*	*Abyssivirga alkaniphila*
Bacillota	Ruminococcaceae	*Acetanaerobacterium*	*Acetanaerobacterium elongatum*
Bacillota	Lachnospiraceae	*Acetatifactor*	*Acetatifactor muris*
Bacillota	Lactobacillaceae	*Acetilactobacillus*	*Acetilactobacillus jinshanensis*
Bacillota	Acetivibrionaceae	*Acetivibrio*	*Acetivibrio aldrichii*
Bacillota	Acetivibrionaceae	*Acetivibrio*	*Acetivibrio alkalicellulosi*
Bacillota	Acetivibrionaceae	*Acetivibrio*	*Acetivibrio cellulolyticus*
Bacillota	Acetivibrionaceae	*Acetivibrio*	*Acetivibrio clariflavus*
Bacillota	Acetivibrionaceae	*Acetivibrio*	*Acetivibrio saccincola*
Bacillota	Acetivibrionaceae	*Acetivibrio*	*Acetivibrio straminisolvens*
Bacillota	Acetivibrionaceae	*Acetivibrio*	*Acetivibrio thermocellus*
Bacillota	Filifactoraceae	*Acetoanaerobium*	*Acetoanaerobium pronyense*
Bacillota	Filifactoraceae	*Acetoanaerobium*	*Acetoanaerobium sticklandii*

Bacillota	Eubacteriaceae	*Acetobacterium*	*Acetobacterium carbinolicum*
Bacillota	Eubacteriaceae	*Acetobacterium*	*Acetobacterium fimetarium*
Bacillota	Eubacteriaceae	*Acetobacterium*	*Acetobacterium paludosum*
Bacillota	Eubacteriaceae	*Acetobacterium*	*Acetobacterium tundrae*
Bacillota	Eubacteriaceae	*Acetobacterium*	*Acetobacterium wieringae*
Bacillota	Eubacteriaceae	*Acetobacterium*	*Acetobacterium woodii*
Bacillota	Acetonemaceae	*Acetonema*	*Acetonema longum*
Bacillota	Acholeplasmataceae	*Acholeplasma*	*Acholeplasma palmae*
Bacillota	Acidaminobacteraceae	*Acidaminobacter*	*Acidaminobacter hydrogenoformans*
Bacillota	Acutalibacteraceae	*Acutalibacter*	*Acutalibacter muris*
Bacillota	Aerococcaceae	*Aerococcus*	*Aerococcus christensenii*
Bacillota	Aerococcaceae	*Aerococcus*	*Aerococcus sanguinicola*
Bacillota	Aerococcaceae	*Aerococcus*	*Aerococcus suis*
Bacillota	Aerococcaceae	*Aerococcus*	*Aerococcus urinae*
Bacillota	Aerococcaceae	*Aerococcus*	*Aerococcus urinaeequi*
Bacillota	Aerococcaceae	*Aerococcus*	*Aerococcus urinaehominis*
Bacillota	Aerococcaceae	*Aerococcus*	*Aerococcus vaginalis*
Bacillota	Aerococcaceae	*Aerococcus*	*Aerococcus viridans*
Bacillota	Butyricicoccaceae	*Agathobaculum*	*Agathobaculum butyriciproducens*
Bacillota	Lactobacillaceae	*Agrilactobacillus*	*Agrilactobacillus composti*
Bacillota	Alicyclobacillaceae	*Alicyclobacillus*	*Alicyclobacillus contaminans*
Bacillota	Alicyclobacillaceae	*Alicyclobacillus*	*Alicyclobacillus fastidiosus*
Bacillota	Alicyclobacillaceae	*Alicyclobacillus*	*Alicyclobacillus pomorum*
Bacillota	Alicyclobacillaceae	*Alicyclobacillus*	*Alicyclobacillus shizuokensis*
Bacillota	Alkalibacillaceae	*Alkalibacillus*	*Alkalibacillus filiformis*
Bacillota	Alkalibacillaceae	*Alkalibacillus*	*Alkalibacillus haloalkaliphilus*
Bacillota	Alkalibacillaceae	*Alkalibacillus*	*Alkalibacillus silvisoli*
Bacillota	Alkalibacteraceae	*Alkalibacter*	*Alkalibacter saccharofermentans*

Bacillota	Carnobacteriaceae	*Alkalibacterium*	*Alkalibacterium gilvum*
Bacillota	Carnobacteriaceae	*Alkalibacterium*	*Alkalibacterium iburiense*
Bacillota	Carnobacteriaceae	*Alkalibacterium*	*Alkalibacterium indicireducens*
Bacillota	Carnobacteriaceae	*Alkalibacterium*	*Alkalibacterium olivapovliticus*
Bacillota	Carnobacteriaceae	*Alkalibacterium*	*Alkalibacterium pelagium*
Bacillota	Carnobacteriaceae	*Alkalibacterium*	*Alkalibacterium psychrotolerans*
Bacillota	Carnobacteriaceae	*Alkalibacterium*	*Alkalibacterium putridalgicola*
Bacillota	Carnobacteriaceae	*Alkalibacterium*	*Alkalibacterium subtropicum*
Bacillota	Carnobacteriaceae	*Alkalibacterium*	*Alkalibacterium thalassium*
Bacillota	Alkalibacteraceae	*Alkalibaculum*	*Alkalibaculum bacchi*
Bacillota	Salisediminibacteriaceae	*Alkalicoccus*	*Alkalicoccus chagannorensis*
Bacillota	Salisediminibacteriaceae	*Alkalicoccus*	*Alkalicoccus daliensis*
Bacillota	Bacillaceae	*Alkalihalobacillus*	*Alkalihalobacillus akibai*
Bacillota	Bacillaceae	*Alkalihalobacillus*	*Alkalihalobacillus alcalophilus*
Bacillota	Bacillaceae	*Alkalihalobacillus*	*Alkalihalobacillus algicola*
Bacillota	Bacillaceae	*Alkalihalobacillus*	*Alkalihalobacillus alkalisediminis*
Bacillota	Bacillaceae	*Alkalihalobacillus*	*Alkalihalobacillus bogoriensis*
Bacillota	Bacillaceae	*Alkalihalobacillus*	*Alkalihalobacillus caeni*
Bacillota	Bacillaceae	*Alkalihalobacillus*	*Alkalihalobacillus clausii*
Bacillota	Bacillaceae	*Alkalihalobacillus*	*Alkalihalobacillus gibsonii*
Bacillota	Bacillaceae	*Alkalihalobacillus*	*Alkalihalobacillus halodurans*
Bacillota	Bacillaceae	*Alkalihalobacillus*	*Alkalihalobacillus hemicellulosilyticus*
Bacillota	Bacillaceae	*Alkalihalobacillus*	*Alkalihalobacillus lehensis*
Bacillota	Bacillaceae	*Alkalihalobacillus*	*Alkalihalobacillus lindianensis*
Bacillota	Bacillaceae	*Alkalihalobacillus*	*Alkalihalobacillus murimartini*
Bacillota	Bacillaceae	*Alkalihalobacillus*	*Alkalihalobacillus okhensis*
Bacillota	Bacillaceae	*Alkalihalobacillus*	*Alkalihalobacillus pseudalcaliphilus*
Bacillota	Bacillaceae	*Alkalihalobacillus*	*Alkalihalobacillus pseudofirmus*

Bacillota	Bacillaceae	*Alkalihalobacillus*	*Alkalihalobacillus shacheensis*
Bacillota	Bacillaceae	*Alkalihalobacillus*	*Alkalihalobacillus trypoxylicola*
Bacillota	Bacillaceae	*Alkalihalobacillus*	*Alkalihalobacillus wakoensis*
Bacillota	Bacillaceae	*Alkalihalobacillus*	*Alkalihalobacillus xiaoxiensis*
Bacillota	Bacillaceae	*Alkalihalobacterium*	*Alkalihalobacterium elongatum*
Bacillota	Bacillaceae	*Alkalilactibacillus*	*Alkalilactibacillus ikkensis*
Bacillota	Natronincolaceae	*Alkaliphilus*	*Alkaliphilus crotonatoxidans*
Bacillota	Natronincolaceae	*Alkaliphilus*	*Alkaliphilus hydrothermalis*
Bacillota	Natronincolaceae	*Alkaliphilus*	*Alkaliphilus metalliredigens*
Bacillota	Natronincolaceae	*Alkaliphilus*	*Alkaliphilus namsaraevii*
Bacillota	Natronincolaceae	*Alkaliphilus*	*Alkaliphilus peptidifermentans*
Bacillota	Natronincolaceae	*Alkaliphilus*	*Alkaliphilus transvaalensis*
Bacillota	Carnobacteriaceae	*Alloiococcus*	*Alloiococcus otitis*
Bacillota	Incertae	*Altericista*	*Altericista lacusladogae*
Bacillota	Anaerovoracaceae	*Aminipila*	*Aminipila butyrica*
Bacillota	Amphibacillaceae	*Amphibacillus*	*Amphibacillus jilinensis*
Bacillota	Lactobacillaceae	*Amylolactobacillus*	*Amylolactobacillus amylophilus*
Bacillota	Lactobacillaceae	*Amylolactobacillus*	*Amylolactobacillus amylotrophicus*
Bacillota	Sporomusaceae	*Anaeroarcus*	*Anaeroarcus burkinensis*
Bacillota	Anaerobacillaceae	*Anaerobacillus*	*Anaerobacillus alkalidiazotrophicus*
Bacillota	Anaerobacillaceae	*Anaerobacillus*	*Anaerobacillus alkalilacustris*
Bacillota	Anaerobacillaceae	*Anaerobacillus*	*Anaerobacillus arseniciselenatis*
Bacillota	Acetivibrionaceae	*Anaerobacterium*	*Anaerobacterium chartisolvens*
Bacillota	Lachnospiraceae	*Anaerobium*	*Anaerobium acetethylicum*
Bacillota	Proteinivoraceae	*Anaerobranca*	*Anaerobranca horikoshii*
Bacillota	Helcococcaceae	*Anaerococcus*	*Anaerococcus prevotii*
Bacillota	Lachnospiraceae	*Anaerocolumna*	*Anaerocolumna aminovalerica*
Bacillota	Lachnospiraceae	*Anaerocolumna*	*Anaerocolumna cellulosilytica*

Bacillota	Lachnospiraceae	*Anaerocolumna*	*Anaerocolumna jejuensis*
Bacillota	Lachnospiraceae	*Anaerocolumna*	*Anaerocolumna xylanovorans*
Bacillota	Ruminococcaceae	*Anaerofilum*	*Anaerofilum agile*
Bacillota	Ruminococcaceae	*Anaerofilum*	*Anaerofilum pentosovorans*
Bacillota	Anaerofustaceae	*Anaerofustis*	*Anaerofustis stercorihominis*
Bacillota	Acutalibacteraceae	*Anaeromassilibacillus*	*Anaeromassilibacillus senegalensis*
Bacillota	Anaeromusaceae	*Anaeromusa*	*Anaeromusa acidaminophila*
Bacillota	Sporomusaceae	*Anaerosinus*	*Anaerosinus glycerini*
Bacillota	Thermosinaceae	*Anaerospora*	*Anaerospora hongkongensis*
Bacillota	Lachnospiraceae	*Anaerosporobacter*	*Anaerosporobacter mobilis*
Bacillota	Acetonemaceae	*Anaerosporomusa*	*Anaerosporomusa subterranea*
Bacillota	Lachnospiraceae	*Anaerostipes*	*Anaerostipes butyraticus*
Bacillota	Lachnospiraceae	*Anaerostipes*	*Anaerostipes caccae*
Bacillota	Lachnospiraceae	*Anaerostipes*	*Anaerostipes hadrus*
Bacillota	Lachnospiraceae	*Anaerotaenia*	*Anaerotaenia torta*
Bacillota	Eubacteriaceae	*Anaerotalea*	*Anaerotalea alkaliphila*
Bacillota	Anaerotignaceae	*Anaerotignum*	*Anaerotignum aminivorans*
Bacillota	Anaerotignaceae	*Anaerotignum*	*Anaerotignum faecicola*
Bacillota	Anaerotignaceae	*Anaerotignum*	*Anaerotignum lactatifermentans*
Bacillota	Anaerotignaceae	*Anaerotignum*	*Anaerotignum neopropionicum*
Bacillota	Anaerotignaceae	*Anaerotignum*	*Anaerotignum propionicum*
Bacillota	Ruminococcaceae	*Anaerotruncus*	*Anaerotruncus colihominis*
Bacillota	Ruminococcaceae	*Anaerotruncus*	*Anaerotruncus rubiinfantis*
Bacillota	Anaerovoracaceae	*Anaerovorax*	*Anaerovorax odorimutans*
Bacillota	Incertae	*Ancylothrix*	*Ancylothrix rivularis*
Bacillota	Gottschalkiaceae	*Andreesenia*	*Andreesenia angusta*
Bacillota	Aneurinibacillaceae	*Aneurinibacillus*	*Aneurinibacillus aneurinilyticus*
Bacillota	Aneurinibacillaceae	*Aneurinibacillus*	*Aneurinibacillus danicus*

Bacillota	Aneurinibacillaceae	*Aneurinibacillus*	*Aneurinibacillus migulanus*
Bacillota	Aneurinibacillaceae	*Aneurinibacillus*	*Aneurinibacillus sediminis*
Bacillota	Aneurinibacillaceae	*Aneurinibacillus*	*Aneurinibacillus soli*
Bacillota	Aneurinibacillaceae	*Aneurinibacillus*	*Aneurinibacillus thermoaerophilus*
Bacillota	Aneurinibacillaceae	*Aneurinibacillus*	*Aneurinibacillus tyrosinisolvens*
Bacillota	Anoxybacillaceae	*Anoxybacillus*	*Anoxybacillus amylolyticus*
Bacillota	Anoxybacillaceae	*Anoxybacillus*	*Anoxybacillus caldiproteolyticus*
Bacillota	Anoxybacillaceae	*Anoxybacillus*	*Anoxybacillus calidus*
Bacillota	Anoxybacillaceae	*Anoxybacillus*	*Anoxybacillus contaminans*
Bacillota	Anoxybacillaceae	*Anoxybacillus*	*Anoxybacillus eryuanensis*
Bacillota	Anoxybacillaceae	*Anoxybacillus*	*Anoxybacillus flavithermus*
Bacillota	Anoxybacillaceae	*Anoxybacillus*	*Anoxybacillus geothermalis*
Bacillota	Anoxybacillaceae	*Anoxybacillus*	*Anoxybacillus kaynarcensis*
Bacillota	Anoxybacillaceae	*Anoxybacillus*	*Anoxybacillus kestanbolensis*
Bacillota	Anoxybacillaceae	*Anoxybacillus*	*Anoxybacillus thermarum*
Bacillota	Anoxybacillaceae	*Anoxybacillus*	*Anoxybacillus vitaminiphilus*
Bacillota	Clostridiaceae	*Anoxynatronum*	*Anoxynatronum buryatiense*
Bacillota	Lactobacillaceae	*Apilactobacillus*	*Apilactobacillus apinorum*
Bacillota	Lactobacillaceae	*Apilactobacillus*	*Apilactobacillus kunkeei*
Bacillota	Lactobacillaceae	*Apilactobacillus*	*Apilactobacillus ozensis*
Bacillota	Amphibacillaceae	*Aquibacillus*	*Aquibacillus halophilus*
Bacillota	Peptostreptococcaceae	*Asaccharospora*	*Asaccharospora irregularis*
Bacillota	Aerococcaceae	*Atopobacter*	*Atopobacter phocae*
Bacillota	Carnobacteriaceae	*Atopostipes*	*Atopostipes suicloacalis*
Bacillota	Bacillaceae	*Aureibacillus*	*Aureibacillus halotolerans*
Bacillota	Bacillaceae	*Bacillus*	*Bacillus acidiceler*
Bacillota	Bacillaceae	*Bacillus*	*Bacillus acidicola*
Bacillota	Bacillaceae	*Bacillus*	*Bacillus aeolius*

Bacillota	Bacillaceae	*Bacillus*	*Bacillus aerius*
Bacillota	Bacillaceae	*Bacillus*	*Bacillus aerophilus*
Bacillota	Bacillaceae	*Bacillus*	*Bacillus akibai*
Bacillota	Bacillaceae	*Bacillus*	*Bacillus alcalophilus*
Bacillota	Bacillaceae	*Bacillus*	*Bacillus algicola*
Bacillota	Bacillaceae	*Bacillus*	*Bacillus alkalisediminis*
Bacillota	Bacillaceae	*Bacillus*	*Bacillus alkalitelluris*
Bacillota	Bacillaceae	*Bacillus*	*Bacillus alkalitolerans*
Bacillota	Bacillaceae	*Bacillus*	*Bacillus altitudinis*
Bacillota	Bacillaceae	*Bacillus*	*Bacillus alveayuensis*
Bacillota	Bacillaceae	*Bacillus*	*Bacillus amyloliquefaciens*
Bacillota	Bacillaceae	*Bacillus*	*Bacillus andreraoultii*
Bacillota	Bacillaceae	*Bacillus*	*Bacillus aquiflavi*
Bacillota	Bacillaceae	*Bacillus*	*Bacillus aquimaris*
Bacillota	Bacillaceae	*Bacillus*	*Bacillus aryabhattai*
Bacillota	Bacillaceae	*Bacillus*	*Bacillus asahii*
Bacillota	Bacillaceae	*Bacillus*	*Bacillus atrophaeus*
Bacillota	Bacillaceae	*Bacillus*	*Bacillus australimaris*
Bacillota	Bacillaceae	*Bacillus*	*Bacillus azotoformans*
Bacillota	Bacillaceae	*Bacillus*	*Bacillus badius*
Bacillota	Bacillaceae	*Bacillus*	*Bacillus bataviensis*
Bacillota	Bacillaceae	*Bacillus*	*Bacillus benzoevorans*
Bacillota	Bacillaceae	*Bacillus*	*Bacillus beringensis*
Bacillota	Bacillaceae	*Bacillus*	*Bacillus berkeleyi*
Bacillota	Bacillaceae	*Bacillus*	*Bacillus bingmayongensis*
Bacillota	Bacillaceae	*Bacillus*	*Bacillus borbori*
Bacillota	Bacillaceae	*Bacillus*	*Bacillus capparidis*
Bacillota	Bacillaceae	*Bacillus*	*Bacillus carboniphilus*

Bacillota	Bacillaceae	*Bacillus*	*Bacillus cavernae*
Bacillota	Bacillaceae	*Bacillus*	*Bacillus cecembensis*
Bacillota	Bacillaceae	*Bacillus*	*Bacillus cellulosilyticus*
Bacillota	Bacillaceae	*Bacillus*	*Bacillus cereus*
Bacillota	Bacillaceae	*Bacillus*	*Bacillus chungangensis*
Bacillota	Bacillaceae	*Bacillus*	*Bacillus ciccensis*
Bacillota	Bacillaceae	*Bacillus*	*Bacillus circulans*
Bacillota	Bacillaceae	*Bacillus*	*Bacillus clausii*
Bacillota	Bacillaceae	*Bacillus*	*Bacillus coagulans*
Bacillota	Bacillaceae	*Bacillus*	*Bacillus coahuilensis*
Bacillota	Bacillaceae	*Bacillus*	*Bacillus cohnii*
Bacillota	Bacillaceae	*Bacillus*	*Bacillus cytotoxicus*
Bacillota	Bacillaceae	*Bacillus*	*Bacillus dakarensis*
Bacillota	Bacillaceae	*Bacillus*	*Bacillus decolorationis*
Bacillota	Bacillaceae	*Bacillus*	*Bacillus deserti*
Bacillota	Bacillaceae	*Bacillus*	*Bacillus drentensis*
Bacillota	Bacillaceae	*Bacillus*	*Bacillus ectoiniformans*
Bacillota	Bacillaceae	*Bacillus*	*Bacillus eiseniae*
Bacillota	Bacillaceae	*Bacillus*	*Bacillus enclensis*
Bacillota	Bacillaceae	*Bacillus*	*Bacillus endoradicis*
Bacillota	Bacillaceae	*Bacillus*	*Bacillus fastidiosus*
Bacillota	Bacillaceae	*Bacillus*	*Bacillus fermenti*
Bacillota	Bacillaceae	*Bacillus*	*Bacillus firmus*
Bacillota	Bacillaceae	*Bacillus*	*Bacillus flexus*
Bacillota	Bacillaceae	*Bacillus*	*Bacillus foraminis*
Bacillota	Bacillaceae	*Bacillus*	*Bacillus fungorum*
Bacillota	Bacillaceae	*Bacillus*	*Bacillus funiculus*
Bacillota	Bacillaceae	*Bacillus*	*Bacillus gaemokensis*

Bacillota	Bacillaceae	*Bacillus*	*Bacillus galliciensis*
Bacillota	Bacillaceae	*Bacillus*	*Bacillus ginsengihumi*
Bacillota	Bacillaceae	*Bacillus*	*Bacillus ginsengisoli*
Bacillota	Bacillaceae	*Bacillus*	*Bacillus gossypii*
Bacillota	Bacillaceae	*Bacillus*	*Bacillus gottheilii*
Bacillota	Bacillaceae	*Bacillus*	*Bacillus haikouensis*
Bacillota	Bacillaceae	*Bacillus*	*Bacillus halmapalus*
Bacillota	Bacillaceae	*Bacillus*	*Bacillus halodurans*
Bacillota	Bacillaceae	*Bacillus*	*Bacillus halosaccharovorans*
Bacillota	Bacillaceae	*Bacillus*	*Bacillus halotolerans*
Bacillota	Bacillaceae	*Bacillus*	*Bacillus haynesii*
Bacillota	Bacillaceae	*Bacillus*	*Bacillus hemicellulosilyticus*
Bacillota	Bacillaceae	*Bacillus*	*Bacillus herbersteinensis*
Bacillota	Bacillaceae	*Bacillus*	*Bacillus horikoshii*
Bacillota	Bacillaceae	*Bacillus*	*Bacillus horneckiae*
Bacillota	Bacillaceae	*Bacillus*	*Bacillus horti*
Bacillota	Bacillaceae	*Bacillus*	*Bacillus huizhouensis*
Bacillota	Bacillaceae	*Bacillus*	*Bacillus humi*
Bacillota	Bacillaceae	*Bacillus*	*Bacillus hwajinpoensis*
Bacillota	Bacillaceae	*Bacillus*	*Bacillus idriensis*
Bacillota	Bacillaceae	*Bacillus*	*Bacillus indicus*
Bacillota	Bacillaceae	*Bacillus*	*Bacillus infantis*
Bacillota	Bacillaceae	*Bacillus*	*Bacillus infernus*
Bacillota	Bacillaceae	*Bacillus*	*Bacillus iocasae*
Bacillota	Bacillaceae	*Bacillus*	*Bacillus isabeliae*
Bacillota	Bacillaceae	*Bacillus*	*Bacillus kexueae*
Bacillota	Bacillaceae	*Bacillus*	*Bacillus kochii*
Bacillota	Bacillaceae	*Bacillus*	*Bacillus koreensis*

Bacillota	Bacillaceae	*Bacillus*	*Bacillus korlensis*
Bacillota	Bacillaceae	*Bacillus*	*Bacillus kribbensis*
Bacillota	Bacillaceae	*Bacillus*	*Bacillus lacus*
Bacillota	Bacillaceae	*Bacillus*	*Bacillus lehensis*
Bacillota	Bacillaceae	*Bacillus*	*Bacillus lentus*
Bacillota	Bacillaceae	*Bacillus*	*Bacillus licheniformis*
Bacillota	Bacillaceae	*Bacillus*	*Bacillus lindianensis*
Bacillota	Bacillaceae	*Bacillus*	*Bacillus litoralis*
Bacillota	Bacillaceae	*Bacillus*	*Bacillus lonarensis*
Bacillota	Bacillaceae	*Bacillus*	*Bacillus luciferensis*
Bacillota	Bacillaceae	*Bacillus*	*Bacillus lycopersici*
Bacillota	Bacillaceae	*Bacillus*	*Bacillus malikii*
Bacillota	Bacillaceae	*Bacillus*	*Bacillus mangrovi*
Bacillota	Bacillaceae	*Bacillus*	*Bacillus manliponensis*
Bacillota	Bacillaceae	*Bacillus*	*Bacillus mannanilyticus*
Bacillota	Bacillaceae	*Bacillus*	*Bacillus manusensis*
Bacillota	Bacillaceae	*Bacillus*	*Bacillus marasmi*
Bacillota	Bacillaceae	*Bacillus*	*Bacillus marcorestinctum*
Bacillota	Bacillaceae	*Bacillus*	*Bacillus marinisedimentorum*
Bacillota	Bacillaceae	*Bacillus*	*Bacillus marisflavi*
Bacillota	Bacillaceae	*Bacillus*	*Bacillus maritimus*
Bacillota	Bacillaceae	*Bacillus*	*Bacillus massilioanorexius*
Bacillota	Bacillaceae	*Bacillus*	*Bacillus massiliogorillae*
Bacillota	Bacillaceae	*Bacillus*	*Bacillus mediterraneensis*
Bacillota	Bacillaceae	*Bacillus*	*Bacillus megaterium*
Bacillota	Bacillaceae	*Bacillus*	*Bacillus mesophilus*
Bacillota	Bacillaceae	*Bacillus*	*Bacillus methanolicus*
Bacillota	Bacillaceae	*Bacillus*	*Bacillus mobilis*

Bacillota	Bacillaceae	*Bacillus*	*Bacillus muralis*
Bacillota	Bacillaceae	*Bacillus*	*Bacillus murimartini*
Bacillota	Bacillaceae	*Bacillus*	*Bacillus mycoides*
Bacillota	Bacillaceae	*Bacillus*	*Bacillus nakamurai*
Bacillota	Bacillaceae	*Bacillus*	*Bacillus nealsonii*
Bacillota	Bacillaceae	*Bacillus*	*Bacillus nematocida*
Bacillota	Bacillaceae	*Bacillus*	*Bacillus niabensis*
Bacillota	Bacillaceae	*Bacillus*	*Bacillus niacini*
Bacillota	Bacillaceae	*Bacillus*	*Bacillus niameyensis*
Bacillota	Bacillaceae	*Bacillus*	*Bacillus notoginsengisoli*
Bacillota	Bacillaceae	*Bacillus*	*Bacillus novalis*
Bacillota	Bacillaceae	*Bacillus*	*Bacillus oceani*
Bacillota	Bacillaceae	*Bacillus*	*Bacillus oceanisediminis*
Bacillota	Bacillaceae	*Bacillus*	*Bacillus okuhidensis*
Bacillota	Bacillaceae	*Bacillus*	*Bacillus oleivorans*
Bacillota	Bacillaceae	*Bacillus*	*Bacillus oleronius*
Bacillota	Bacillaceae	*Bacillus*	*Bacillus oryzaecorticis*
Bacillota	Bacillaceae	*Bacillus*	*Bacillus oryzisoli*
Bacillota	Bacillaceae	*Bacillus*	*Bacillus oryziterrae*
Bacillota	Bacillaceae	*Bacillus*	*Bacillus pacificus*
Bacillota	Bacillaceae	*Bacillus*	*Bacillus pakistanensis*
Bacillota	Bacillaceae	*Bacillus*	*Bacillus panacisoli*
Bacillota	Bacillaceae	*Bacillus*	*Bacillus panaciterrae*
Bacillota	Bacillaceae	*Bacillus*	*Bacillus paraflexus*
Bacillota	Bacillaceae	*Bacillus*	*Bacillus paramycoides*
Bacillota	Bacillaceae	*Bacillus*	*Bacillus paranthracis*
Bacillota	Bacillaceae	*Bacillus*	*Bacillus persicus*
Bacillota	Bacillaceae	*Bacillus*	*Bacillus pervagus*

Bacillota	Bacillaceae	*Bacillus*	*Bacillus pocheonensis*
Bacillota	Bacillaceae	*Bacillus*	*Bacillus polygoni*
Bacillota	Bacillaceae	*Bacillus*	*Bacillus praedii*
Bacillota	Bacillaceae	*Bacillus*	*Bacillus proteolyticus*
Bacillota	Bacillaceae	*Bacillus*	*Bacillus pseudalcaliphilus*
Bacillota	Bacillaceae	*Bacillus*	*Bacillus pseudofirmus*
Bacillota	Bacillaceae	*Bacillus*	*Bacillus pseudomycoides*
Bacillota	Bacillaceae	*Bacillus*	*Bacillus psychrosaccharolyticus*
Bacillota	Bacillaceae	*Bacillus*	*Bacillus purgationiresistens*
Bacillota	Bacillaceae	*Bacillus*	*Bacillus racemilacticus*
Bacillota	Bacillaceae	*Bacillus*	*Bacillus rubiinfantis*
Bacillota	Bacillaceae	*Bacillus*	*Bacillus safensis*
Bacillota	Bacillaceae	*Bacillus*	*Bacillus salarius*
Bacillota	Bacillaceae	*Bacillus*	*Bacillus salitolerans*
Bacillota	Bacillaceae	*Bacillus*	*Bacillus selenitireducens*
Bacillota	Bacillaceae	*Bacillus*	*Bacillus shacheensis*
Bacillota	Bacillaceae	*Bacillus*	*Bacillus shackletonii*
Bacillota	Bacillaceae	*Bacillus*	*Bacillus siamensis*
Bacillota	Bacillaceae	*Bacillus*	*Bacillus simplex*
Bacillota	Bacillaceae	*Bacillus*	*Bacillus smithii*
Bacillota	Bacillaceae	*Bacillus*	*Bacillus solisilvae*
Bacillota	Bacillaceae	*Bacillus*	*Bacillus songklensis*
Bacillota	Bacillaceae	*Bacillus*	*Bacillus spongiae*
Bacillota	Bacillaceae	*Bacillus*	*Bacillus sporothermodurans*
Bacillota	Bacillaceae	*Bacillus*	*Bacillus stamsii*
Bacillota	Bacillaceae	*Bacillus*	*Bacillus stratosphericus*
Bacillota	Bacillaceae	*Bacillus*	*Bacillus stratosphericus;Bacillus*
Bacillota	Bacillaceae	*Bacillus*	*Bacillus subtilis*

Bacillota	Bacillaceae	*Bacillus*	*Bacillus swezeyi*
Bacillota	Bacillaceae	*Bacillus*	*Bacillus taiwanensis*
Bacillota	Bacillaceae	*Bacillus*	*Bacillus tamaricis*
Bacillota	Bacillaceae	*Bacillus*	*Bacillus tequilensis*
Bacillota	Bacillaceae	*Bacillus*	*Bacillus terrae*
Bacillota	Bacillaceae	*Bacillus*	*Bacillus testis*
Bacillota	Bacillaceae	*Bacillus*	*Bacillus thermocloacae*
Bacillota	Bacillaceae	*Bacillus*	*Bacillus thermocopriae*
Bacillota	Bacillaceae	*Bacillus*	*Bacillus thuringiensis*
Bacillota	Bacillaceae	*Bacillus*	*Bacillus tianmuensis*
Bacillota	Bacillaceae	*Bacillus*	*Bacillus tianshenii*
Bacillota	Bacillaceae	*Bacillus*	*Bacillus timonensis*
Bacillota	Bacillaceae	*Bacillus*	*Bacillus toyonensis*
Bacillota	Bacillaceae	*Bacillus*	*Bacillus tropicus*
Bacillota	Bacillaceae	*Bacillus*	*Bacillus tropicus;Bacillus*
Bacillota	Bacillaceae	*Bacillus*	*Bacillus trypoxylicola*
Bacillota	Bacillaceae	*Bacillus*	*Bacillus vallismortis*
Bacillota	Bacillaceae	*Bacillus*	*Bacillus velezensis*
Bacillota	Bacillaceae	*Bacillus*	*Bacillus wakoensis*
Bacillota	Bacillaceae	*Bacillus*	*Bacillus wiedmannii*
Bacillota	Bacillaceae	*Bacillus*	*Bacillus wudalianchiensis*
Bacillota	Bacillaceae	*Bacillus*	*Bacillus wuyishanensis*
Bacillota	Bacillaceae	*Bacillus*	*Bacillus xiamenensis*
Bacillota	Bacillaceae	*Bacillus*	*Bacillus xiaoxiensis*
Bacillota	Bacillaceae	*Bacillus*	*Bacillus xiapuensis*
Bacillota	Bacillaceae	*Bacillus*	*Bacillus yapensis*
Bacillota	Bacillaceae	*Bacillus*	*Bacillus zeae*
Bacillota	Bacillaceae	*Bacillus*	*Bacillus zhangzhouensis*

Bacillota	Bacillaceae	*Bacillus*	*Bacillus zhanjiangensis*
Bacillota	Thermoactinomycetaceae	*Baia*	*Baia soyae*
Bacillota	Lachnospiraceae	*Bariatricus*	*Bariatricus massiliensis*
Bacillota	Aerococcaceae	*Bavariicoccus*	*Bavariicoccus seileri*
Bacillota	Planococcaceae	*Bhargavaea*	*Bhargavaea beijingensis*
Bacillota	Planococcaceae	*Bhargavaea*	*Bhargavaea cecembensis*
Bacillota	Planococcaceae	*Bhargavaea*	*Bhargavaea ginsengi*
Bacillota	Planococcaceae	*Bhargavaea*	*Bhargavaea indica*
Bacillota	Lachnospiraceae	*Blautia*	*Blautia argi*
Bacillota	Lachnospiraceae	*Blautia*	*Blautia coccoides*
Bacillota	Lachnospiraceae	*Blautia*	*Blautia faecicola*
Bacillota	Lachnospiraceae	*Blautia*	*Blautia glucerasea*
Bacillota	Lachnospiraceae	*Blautia*	*Blautia hansenii*
Bacillota	Lachnospiraceae	*Blautia*	*Blautia hominis*
Bacillota	Lachnospiraceae	*Blautia*	*Blautia hydrogenotrophica*
Bacillota	Lachnospiraceae	*Blautia*	*Blautia luti*
Bacillota	Lachnospiraceae	*Blautia*	*Blautia marasmi*
Bacillota	Lachnospiraceae	*Blautia*	*Blautia obeum*
Bacillota	Lachnospiraceae	*Blautia*	*Blautia producta*
Bacillota	Lachnospiraceae	*Blautia*	*Blautia schinkii*
Bacillota	Lachnospiraceae	*Blautia*	*Blautia stercoris*
Bacillota	Lactobacillaceae	*Bombilactobacillus*	*Bombilactobacillus bombi*
Bacillota	Lactobacillaceae	*Bombilactobacillus*	*Bombilactobacillus mellifer*
Bacillota	Lactobacillaceae	*Bombilactobacillus*	*Bombilactobacillus mellis*
Bacillota	Thermohalobacteraceae	*Brassicibacter*	*Brassicibacter mesophilus*
Bacillota	Thermohalobacteraceae	*Brassicibacter*	*Brassicibacter thermophilus*
Bacillota	Brevibacillaceae	*Brevibacillus*	*Brevibacillus borstelensis*
Bacillota	Brevibacillaceae	*Brevibacillus*	*Brevibacillus choshinensis*

Bacillota	Brevibacillaceae	*Brevibacillus*	*Brevibacillus laterosporus*
Bacillota	Brevibacillaceae	*Brevibacillus*	*Brevibacillus nitrificans*
Bacillota	Brevibacillaceae	*Brevibacillus*	*Brevibacillus reuszeri*
Bacillota	Brevibacillaceae	*Brevibacillus*	*Brevibacillus thermoruber*
Bacillota	Erysipelotrichaceae	*Breznakia*	*Breznakia blatticola*
Bacillota	Erysipelotrichaceae	*Breznakia*	*Breznakia pachnodae*
Bacillota	Brochotrichaceae	*Brochothrix*	*Brochothrix campestris*
Bacillota	Brochotrichaceae	*Brochothrix*	*Brochothrix thermosphacta*
Bacillota	Butyricicoccaceae	*Butyricicoccus*	*Butyricicoccus faecihominis*
Bacillota	Butyricicoccaceae	*Butyricicoccus*	*Butyricicoccus pullicaecorum*
Bacillota	Lachnospiraceae	*Caecibacterium*	*Caecibacterium sporoformans*
Bacillota	Caldalkalibacillaceae	*Caldalkalibacillus*	*Caldalkalibacillus mannanilyticus*
Bacillota	Caldalkalibacillaceae	*Caldalkalibacillus*	*Caldalkalibacillus thermarum*
Bacillota	Caldibacillaceae	*Caldibacillus*	*Caldibacillus kokeshiiformis*
Bacillota	Caldibacillaceae	*Caldibacillus*	*Caldibacillus thermoamylovorans*
Bacillota	Caldibacillaceae	*Caldibacillus*	*Caldibacillus thermolactis*
Bacillota	Caldicellulosiruptoraceae	*Caldicellulosiruptor*	*Caldicellulosiruptor acetigenus*
Bacillota	Caldicoprobacteraceae	*Caldicoprobacter*	*Caldicoprobacter faecalis*
Bacillota	Caldicoprobacteraceae	*Caldicoprobacter*	*Caldicoprobacter guelmensis*
Bacillota	Caloramatoraceae	*Caloramator*	*Caloramator australicus*
Bacillota	Caloramatoraceae	*Caloramator*	*Caloramator mitchellensis*
Bacillota	Caloranaerobacteraceae	*Caloranaerobacter*	*Caloranaerobacter azorensis*
Bacillota	Caloranaerobacteraceae	*Caloranaerobacter*	*Caloranaerobacter ferrireducens*
Bacillota	Caminicellaceae	*Caminicella*	*Caminicella sporogenes*
Bacillota	Acutalibacteraceae	*Caproiciproducens*	*Caproiciproducens galactitolivorans*
Bacillota	Carnobacteriaceae	*Carnobacterium*	*Carnobacterium alterfunditum*
Bacillota	Carnobacteriaceae	*Carnobacterium*	*Carnobacterium divergens*
Bacillota	Carnobacteriaceae	*Carnobacterium*	*Carnobacterium funditum*

Bacillota	Carnobacteriaceae	*Carnobacterium*	*Carnobacterium gallinarum*
Bacillota	Carnobacteriaceae	*Carnobacterium*	*Carnobacterium iners*
Bacillota	Carnobacteriaceae	*Carnobacterium*	*Carnobacterium inhibens*
Bacillota	Carnobacteriaceae	*Carnobacterium*	*Carnobacterium jeotgali*
Bacillota	Carnobacteriaceae	*Carnobacterium*	*Carnobacterium maltaromaticum*
Bacillota	Carnobacteriaceae	*Carnobacterium*	*Carnobacterium mobile*
Bacillota	Carnobacteriaceae	*Carnobacterium*	*Carnobacterium pleistocenium*
Bacillota	Carnobacteriaceae	*Carnobacterium*	*Carnobacterium viridans*
Bacillota	Planococcaceae	*Caryophanon*	*Caryophanon latum*
Bacillota	Eubacteriaceae	*Casaltella*	*Casaltella massiliensis*
Bacillota	Christensenellaceae	*Catabacter*	*Catabacter hongkongensis*
Bacillota	Catellicoccaceae	*Catellicoccus*	*Catellicoccus marimammalium*
Bacillota	Cellulosilyticaceae	*Cellulosilyticum*	*Cellulosilyticum lentocellum*
Bacillota	Cellulosilyticaceae	*Cellulosilyticum*	*Cellulosilyticum ruminicola*
Bacillota	Paenibacillaceae	*Chengkuizengella*	*Chengkuizengella sediminis*
Bacillota	Christensenellaceae	*Christensenella*	*Christensenella hongkongensis*
Bacillota	Christensenellaceae	*Christensenella*	*Christensenella massiliensis*
Bacillota	Christensenellaceae	*Christensenella*	*Christensenella minuta*
Bacillota	Christensenellaceae	*Christensenella*	*Christensenella timonensis*
Bacillota	Planococcaceae	*Chryseomicrobium*	*Chryseomicrobium deserti*
Bacillota	Planococcaceae	*Chryseomicrobium*	*Chryseomicrobium palamuruense*
Bacillota	Peptostreptococcaceae	*Clostridioides*	*Clostridioides difficile*
Bacillota	Peptostreptococcaceae	*Clostridioides*	*Clostridioides mangenotii*
Bacillota	Clostridiaceae	*Clostridium*	*Clostridium acetireducens*
Bacillota	Clostridiaceae	*Clostridium*	*Clostridium acidisoli*
Bacillota	Clostridiaceae	*Clostridium*	*Clostridium aciditolerans*
Bacillota	Clostridiaceae	*Clostridium*	*Clostridium aerotolerans*
Bacillota	Clostridiaceae	*Clostridium*	*Clostridium aestuarii*

Bacillota	Clostridiaceae	*Clostridium*	*Clostridium akagii*
Bacillota	Clostridiaceae	*Clostridium*	*Clostridium algidicarnis*
Bacillota	Clostridiaceae	*Clostridium*	*Clostridium algidixylanolyticum*
Bacillota	Clostridiaceae	*Clostridium*	*Clostridium algifaecis*
Bacillota	Clostridiaceae	*Clostridium*	*Clostridium amazonense*
Bacillota	Clostridiaceae	*Clostridium*	*Clostridium amygdalinum*
Bacillota	Clostridiaceae	*Clostridium*	*Clostridium amylolyticum*
Bacillota	Clostridiaceae	*Clostridium*	*Clostridium arbusti*
Bacillota	Clostridiaceae	*Clostridium*	*Clostridium argentinense*
Bacillota	Clostridiaceae	*Clostridium*	*Clostridium aurantibutyricum*
Bacillota	Clostridiaceae	*Clostridium*	*Clostridium autoethanogenum*
Bacillota	Clostridiaceae	*Clostridium*	*Clostridium baratii*
Bacillota	Clostridiaceae	*Clostridium*	*Clostridium beijerinckii*
Bacillota	Clostridiaceae	*Clostridium*	*Clostridium bolteae*
Bacillota	Clostridiaceae	*Clostridium*	*Clostridium bornimense*
Bacillota	Clostridiaceae	*Clostridium*	*Clostridium botulinum*
Bacillota	Clostridiaceae	*Clostridium*	*Clostridium bowmanii*
Bacillota	Clostridiaceae	*Clostridium*	*Clostridium budayi*
Bacillota	Clostridiaceae	*Clostridium*	*Clostridium butyricum*
Bacillota	Clostridiaceae	*Clostridium*	*Clostridium cadaveris*
Bacillota	Clostridiaceae	*Clostridium*	*Clostridium carboxidivorans*
Bacillota	Clostridiaceae	*Clostridium*	*Clostridium carnis*
Bacillota	Clostridiaceae	*Clostridium*	*Clostridium cavendishii*
Bacillota	Clostridiaceae	*Clostridium*	*Clostridium celatum*
Bacillota	Clostridiaceae	*Clostridium*	*Clostridium celerecrescens*
Bacillota	Clostridiaceae	*Clostridium*	*Clostridium cellulosi*
Bacillota	Clostridiaceae	*Clostridium*	*Clostridium cellulovorans*
Bacillota	Clostridiaceae	*Clostridium*	*Clostridium chartatabidum*

Bacillota	Clostridiaceae	*Clostridium*	*Clostridium chauvoei*
Bacillota	Clostridiaceae	*Clostridium*	*Clostridium chromiireducens*
Bacillota	Clostridiaceae	*Clostridium*	*Clostridium citroniae*
Bacillota	Clostridiaceae	*Clostridium*	*Clostridium clostridioforme*
Bacillota	Clostridiaceae	*Clostridium*	*Clostridium cochlearium*
Bacillota	Clostridiaceae	*Clostridium*	*Clostridium cocleatum*
Bacillota	Clostridiaceae	*Clostridium*	*Clostridium colicanis*
Bacillota	Clostridiaceae	*Clostridium*	*Clostridium colinum*
Bacillota	Clostridiaceae	*Clostridium*	*Clostridium collagenovorans*
Bacillota	Clostridiaceae	*Clostridium*	*Clostridium combesii*
Bacillota	Clostridiaceae	*Clostridium*	*Clostridium cylindrosporum*
Bacillota	Clostridiaceae	*Clostridium*	*Clostridium dakarense*
Bacillota	Clostridiaceae	*Clostridium*	*Clostridium diolis*
Bacillota	Clostridiaceae	*Clostridium*	*Clostridium disporicum*
Bacillota	Clostridiaceae	*Clostridium*	*Clostridium drakei*
Bacillota	Clostridiaceae	*Clostridium*	*Clostridium estertheticum*
Bacillota	Clostridiaceae	*Clostridium*	*Clostridium fallax*
Bacillota	Clostridiaceae	*Clostridium*	*Clostridium felsineum*
Bacillota	Clostridiaceae	*Clostridium*	*Clostridium fermenticellae*
Bacillota	Clostridiaceae	*Clostridium*	*Clostridium fimetarium*
Bacillota	Clostridiaceae	*Clostridium*	*Clostridium frigidicarnis*
Bacillota	Clostridiaceae	*Clostridium*	*Clostridium frigoris*
Bacillota	Clostridiaceae	*Clostridium*	*Clostridium ganghwense*
Bacillota	Clostridiaceae	*Clostridium*	*Clostridium gasigenes*
Bacillota	Clostridiaceae	*Clostridium*	*Clostridium glycyrrhizinilyticum*
Bacillota	Clostridiaceae	*Clostridium*	*Clostridium grantii*
Bacillota	Clostridiaceae	*Clostridium*	*Clostridium haemolyticum*
Bacillota	Clostridiaceae	*Clostridium*	*Clostridium herbivorans*

Bacillota	Clostridiaceae	*Clostridium*	*Clostridium hiranonis*
Bacillota	Clostridiaceae	*Clostridium*	*Clostridium homopropionicum*
Bacillota	Clostridiaceae	*Clostridium*	*Clostridium hydrogeniformans*
Bacillota	Clostridiaceae	*Clostridium*	*Clostridium hylemonae*
Bacillota	Clostridiaceae	*Clostridium*	*Clostridium ihumii*
Bacillota	Clostridiaceae	*Clostridium*	*Clostridium indolis*
Bacillota	Clostridiaceae	*Clostridium*	*Clostridium innocuum*
Bacillota	Clostridiaceae	*Clostridium*	*Clostridium intestinale*
Bacillota	Clostridiaceae	*Clostridium*	*Clostridium isatidis*
Bacillota	Clostridiaceae	*Clostridium*	*Clostridium jeddahense*
Bacillota	Clostridiaceae	*Clostridium*	*Clostridium kogasensis*
Bacillota	Clostridiaceae	*Clostridium*	*Clostridium lavalense*
Bacillota	Clostridiaceae	*Clostridium*	*Clostridium leptum*
Bacillota	Clostridiaceae	*Clostridium*	*Clostridium liquoris*
Bacillota	Clostridiaceae	*Clostridium*	*Clostridium ljungdahlii*
Bacillota	Clostridiaceae	*Clostridium*	*Clostridium lundense*
Bacillota	Clostridiaceae	*Clostridium*	*Clostridium magnum*
Bacillota	Clostridiaceae	*Clostridium*	*Clostridium malenominatum*
Bacillota	Clostridiaceae	*Clostridium*	*Clostridium massiliodielmoense*
Bacillota	Clostridiaceae	*Clostridium*	*Clostridium maximum*
Bacillota	Clostridiaceae	*Clostridium*	*Clostridium merdae*
Bacillota	Clostridiaceae	*Clostridium*	*Clostridium methylpentosum*
Bacillota	Clostridiaceae	*Clostridium*	*Clostridium moniliforme*
Bacillota	Clostridiaceae	*Clostridium*	*Clostridium nitritogenes*
Bacillota	Clostridiaceae	*Clostridium*	*Clostridium nitrophenolicum*
Bacillota	Clostridiaceae	*Clostridium*	*Clostridium novyi*
Bacillota	Clostridiaceae	*Clostridium*	*Clostridium oceanicum*
Bacillota	Clostridiaceae	*Clostridium*	*Clostridium oryzae*

Bacillota	Clostridiaceae	*Clostridium*	*Clostridium paradoxum*
Bacillota	Clostridiaceae	*Clostridium*	*Clostridium paraputrificum*
Bacillota	Clostridiaceae	*Clostridium*	*Clostridium pascui*
Bacillota	Clostridiaceae	*Clostridium*	*Clostridium pasteurianum*
Bacillota	Clostridiaceae	*Clostridium*	*Clostridium perfringens*
Bacillota	Clostridiaceae	*Clostridium*	*Clostridium polyendosporum*
Bacillota	Clostridiaceae	*Clostridium*	*Clostridium polynesiense*
Bacillota	Clostridiaceae	*Clostridium*	*Clostridium polysaccharolyticum*
Bacillota	Clostridiaceae	*Clostridium*	*Clostridium populeti*
Bacillota	Clostridiaceae	*Clostridium*	*Clostridium punense*
Bacillota	Clostridiaceae	*Clostridium*	*Clostridium puniceum*
Bacillota	Clostridiaceae	*Clostridium*	*Clostridium putrefaciens*
Bacillota	Clostridiaceae	*Clostridium*	*Clostridium quinii*
Bacillota	Clostridiaceae	*Clostridium*	*Clostridium roseum*
Bacillota	Clostridiaceae	*Clostridium*	*Clostridium saccharobutylicum*
Bacillota	Clostridiaceae	*Clostridium*	*Clostridium saccharogumia*
Bacillota	Clostridiaceae	*Clostridium*	*Clostridium saccharolyticum*
Bacillota	Clostridiaceae	*Clostridium*	*Clostridium saccharoperbutylacetonicum*
Bacillota	Clostridiaceae	*Clostridium*	*Clostridium sardiniense*
Bacillota	Clostridiaceae	*Clostridium*	*Clostridium sartagoforme*
Bacillota	Clostridiaceae	*Clostridium*	*Clostridium saudiense*
Bacillota	Clostridiaceae	*Clostridium*	*Clostridium scatologenes*
Bacillota	Clostridiaceae	*Clostridium*	*Clostridium schirmacherense*
Bacillota	Clostridiaceae	*Clostridium*	*Clostridium scindens*
Bacillota	Clostridiaceae	*Clostridium*	*Clostridium senegalense*
Bacillota	Clostridiaceae	*Clostridium*	*Clostridium septicum*
Bacillota	Clostridiaceae	*Clostridium*	*Clostridium sphenoides*
Bacillota	Clostridiaceae	*Clostridium*	*Clostridium spiroforme*

Bacillota	Clostridiaceae	*Clostridium*	*Clostridium sporogenes*
Bacillota	Clostridiaceae	*Clostridium*	*Clostridium subterminale*
Bacillota	Clostridiaceae	*Clostridium*	*Clostridium swellfunianum*
Bacillota	Clostridiaceae	*Clostridium*	*Clostridium symbiosum*
Bacillota	Clostridiaceae	*Clostridium*	*Clostridium tagluense*
Bacillota	Clostridiaceae	*Clostridium*	*Clostridium tarantellae*
Bacillota	Clostridiaceae	*Clostridium*	*Clostridium tepidiprofundi*
Bacillota	Clostridiaceae	*Clostridium*	*Clostridium tepidum*
Bacillota	Clostridiaceae	*Clostridium*	*Clostridium tertium*
Bacillota	Clostridiaceae	*Clostridium*	*Clostridium tetani*
Bacillota	Clostridiaceae	*Clostridium*	*Clostridium tetanomorphum*
Bacillota	Clostridiaceae	*Clostridium*	*Clostridium thermoalcaliphilum*
Bacillota	Clostridiaceae	*Clostridium*	*Clostridium thermobutyricum*
Bacillota	Clostridiaceae	*Clostridium*	*Clostridium thermopalmarium*
Bacillota	Clostridiaceae	*Clostridium*	*Clostridium tunisiense*
Bacillota	Clostridiaceae	*Clostridium*	*Clostridium uliginosum*
Bacillota	Clostridiaceae	*Clostridium*	*Clostridium ventriculi*
Bacillota	Clostridiaceae	*Clostridium*	*Clostridium vincentii*
Bacillota	Clostridiaceae	*Clostridium*	*Clostridium viride*
Bacillota	Clostridiaceae	*Clostridium*	*Clostridium vulturis*
Bacillota	Clostridiaceae	*Clostridium*	*Clostridium xylanolyticum*
Bacillota	Paenibacillaceae	*Cohnella*	*Cohnella boryungensis*
Bacillota	Paenibacillaceae	*Cohnella*	*Cohnella collisoli*
Bacillota	Paenibacillaceae	*Cohnella*	*Cohnella damuensis*
Bacillota	Paenibacillaceae	*Cohnella*	*Cohnella endophytica*
Bacillota	Paenibacillaceae	*Cohnella*	*Cohnella formosensis*
Bacillota	Paenibacillaceae	*Cohnella*	*Cohnella humi*
Bacillota	Paenibacillaceae	*Cohnella*	*Cohnella lubricantis*

Bacillota	Paenibacillaceae	*Cohnella*	*Cohnella lupini*
Bacillota	Paenibacillaceae	*Cohnella*	*Cohnella panacarvi*
Bacillota	Paenibacillaceae	*Cohnella*	*Cohnella phaseoli*
Bacillota	Paenibacillaceae	*Cohnella*	*Cohnella saccharovorans*
Bacillota	Eubacteriaceae	*Colidextribacter*	*Colidextribacter massiliensis*
Bacillota	Lactobacillaceae	*Companilactobacillus*	*Companilactobacillus alimentarius*
Bacillota	Lactobacillaceae	*Companilactobacillus*	*Companilactobacillus allii*
Bacillota	Lactobacillaceae	*Companilactobacillus*	*Companilactobacillus bobalius*
Bacillota	Lactobacillaceae	*Companilactobacillus*	*Companilactobacillus crustorum*
Bacillota	Lactobacillaceae	*Companilactobacillus*	*Companilactobacillus farciminis*
Bacillota	Lactobacillaceae	*Companilactobacillus*	*Companilactobacillus furfuricola*
Bacillota	Lactobacillaceae	*Companilactobacillus*	*Companilactobacillus futsaii*
Bacillota	Lactobacillaceae	*Companilactobacillus*	*Companilactobacillus ginsenosidimutans*
Bacillota	Lactobacillaceae	*Companilactobacillus*	*Companilactobacillus insicii*
Bacillota	Lactobacillaceae	*Companilactobacillus*	*Companilactobacillus kimchiensis*
Bacillota	Lactobacillaceae	*Companilactobacillus*	*Companilactobacillus kimchii*
Bacillota	Lactobacillaceae	*Companilactobacillus*	*Companilactobacillus mindensis*
Bacillota	Lactobacillaceae	*Companilactobacillus*	*Companilactobacillus musae*
Bacillota	Lactobacillaceae	*Companilactobacillus*	*Companilactobacillus nantensis*
Bacillota	Lactobacillaceae	*Companilactobacillus*	*Companilactobacillus nodensis*
Bacillota	Lactobacillaceae	*Companilactobacillus*	*Companilactobacillus paralimentarius*
Bacillota	Lactobacillaceae	*Companilactobacillus*	*Companilactobacillus tucceti*
Bacillota	Lactobacillaceae	*Companilactobacillus*	*Companilactobacillus versmoldensis*
Bacillota	Lactobacillaceae	*Convivina*	*Convivina intestini*
Bacillota	Erysipelatoclostridiaceae	*Coprobacillus*	*Coprobacillus cateniformis*
Bacillota	Lachnospiraceae	*Coprococcus*	*Coprococcus comes*
Bacillota	Staphylococcaceae	*Corticicoccus*	*Corticicoccus populi*
Bacillota	Clostridiaceae	*Crassaminicella*	*Crassaminicella profunda*

Bacillota	Thermoactinomycetaceae	*Croceifilum*	*Croceifilum oryzae*
Bacillota	Lachnospiraceae	*Cuneatibacter*	*Cuneatibacter caecimuris*
Bacillota	Bacillaceae	*Cytobacillus*	*Cytobacillus ciccensis*
Bacillota	Bacillaceae	*Cytobacillus*	*Cytobacillus depressus*
Bacillota	Bacillaceae	*Cytobacillus*	*Cytobacillus eiseniae*
Bacillota	Bacillaceae	*Cytobacillus*	*Cytobacillus firmus*
Bacillota	Bacillaceae	*Cytobacillus*	*Cytobacillus gottheilii*
Bacillota	Bacillaceae	*Cytobacillus*	*Cytobacillus horneckiae*
Bacillota	Bacillaceae	*Cytobacillus*	*Cytobacillus kochii*
Bacillota	Bacillaceae	*Cytobacillus*	*Cytobacillus purgationiresistens*
Bacillota	Defluviitaleaceae	*Defluviitalea*	*Defluviitalea raffinosedens*
Bacillota	Defluviitaleaceae	*Defluviitalea*	*Defluviitalea saccharophila*
Bacillota	Syntrophobotulaceae	*Dehalobacter*	*Dehalobacter restrictus*
Bacillota	Lactobacillaceae	*Dellaglioa*	*Dellaglioa algida*
Bacillota	Dendrosporobacteraceae	*Dendrosporobacter*	*Dendrosporobacter quercicolus*
Bacillota	Carnobacteriaceae	*Desemzia*	*Desemzia incerta*
Bacillota	Clostridiaceae	*Desnuesiella*	*Desnuesiella massiliensis*
Bacillota	Desulfallaceae	*Desulfallas*	*Desulfallas geothermicus*
Bacillota	Peptococcaceae	*Desulfitispora*	*Desulfitispora alkaliphila*
Bacillota	Peptococcaceae	*Desulfitispora*	*Desulfitispora elongata*
Bacillota	Desulfitobacteriaceae	*Desulfitobacterium*	*Desulfitobacterium chlororespirans*
Bacillota	Desulfitobacteriaceae	*Desulfitobacterium*	*Desulfitobacterium dehalogenans*
Bacillota	Desulfitobacteriaceae	*Desulfitobacterium*	*Desulfitobacterium hafniense*
Bacillota	Desulfitobacteriaceae	*Desulfitobacterium*	*Desulfitobacterium metallireducens*
Bacillota	Desulfocucumaceae	*Desulfocucumis*	*Desulfocucumis palustris*
Bacillota	Desulfofarciminaceae	*Desulfofarcimen*	*Desulfofarcimen intricatum*
Bacillota	Desulfovirgulaceae	*Desulfofundulus*	*Desulfofundulus thermobenzoicus*
Bacillota	Desulfotomaculaceae	*Desulfohalotomaculum*	*Desulfohalotomaculum alkaliphilum*

Bacillota	Desulfotomaculaceae	*Desulfohalotomaculum*	*Desulfohalotomaculum halophilum*
Bacillota	Desulfotomaculaceae	*Desulfohalotomaculum*	*Desulfohalotomaculum peckii*
Bacillota	Desulfonisporaceae	*Desulfonispora*	*Desulfonispora thiosulfatigenes*
Bacillota	Desulfitobacteriaceae	*Desulfosporosinus*	*Desulfosporosinus acididurans*
Bacillota	Desulfitobacteriaceae	*Desulfosporosinus*	*Desulfosporosinus acidiphilus*
Bacillota	Desulfitobacteriaceae	*Desulfosporosinus*	*Desulfosporosinus auripigmenti*
Bacillota	Desulfitobacteriaceae	*Desulfosporosinus*	*Desulfosporosinus fructosivorans*
Bacillota	Desulfitobacteriaceae	*Desulfosporosinus*	*Desulfosporosinus hippei*
Bacillota	Desulfitobacteriaceae	*Desulfosporosinus*	*Desulfosporosinus lacus*
Bacillota	Desulfitobacteriaceae	*Desulfosporosinus*	*Desulfosporosinus meridiei*
Bacillota	Desulfitobacteriaceae	*Desulfosporosinus*	*Desulfosporosinus nitroreducens*
Bacillota	Desulfitobacteriaceae	*Desulfosporosinus*	*Desulfosporosinus orientis*
Bacillota	Desulfitobacteriaceae	*Desulfosporosinus*	*Desulfosporosinus youngiae*
Bacillota	Thermoanaerobacteraceae	*Desulfothermobacter*	*Desulfothermobacter acidiphilus*
Bacillota	Desulfotomaculaceae	*Desulfotomaculum*	*Desulfotomaculum aeronauticum*
Bacillota	Desulfotomaculaceae	*Desulfotomaculum*	*Desulfotomaculum guttoideum*
Bacillota	Desulfotomaculaceae	*Desulfotomaculum*	*Desulfotomaculum reducens*
Bacillota	Desulfotomaculaceae	*Desulfotomaculum*	*Desulfotomaculum salinum*
Bacillota	Desulfuribacillaceae	*Desulfuribacillus*	*Desulfuribacillus alkaliarsenatis*
Bacillota	Dialisteraceae	*Dialister*	*Dialister invisus*
Bacillota	Dialisteraceae	*Dialister*	*Dialister micraerophilus*
Bacillota	Aerococcaceae	*Dolosicoccus*	*Dolosicoccus paucivorans*
Bacillota	Domibacillaceae	*Domibacillus*	*Domibacillus antri*
Bacillota	Domibacillaceae	*Domibacillus*	*Domibacillus epiphyticus*
Bacillota	Domibacillaceae	*Domibacillus*	*Domibacillus indicus*
Bacillota	Domibacillaceae	*Domibacillus*	*Domibacillus iocasae*
Bacillota	Domibacillaceae	*Domibacillus*	*Domibacillus mangrovi*
Bacillota	Domibacillaceae	*Domibacillus*	*Domibacillus robiginosus*

Bacillota	Domibacillaceae	*Domibacillus*	*Domibacillus tundrae*
Bacillota	Lachnospiraceae	*Dorea*	*Dorea formicigenerans*
Bacillota	Bacillaceae	*Ectobacillus*	*Ectobacillus funiculus*
Bacillota	Bacillaceae	*Ectobacillus*	*Ectobacillus panaciterrae*
Bacillota	Effusibacillaceae	*Effusibacillus*	*Effusibacillus consociatus*
Bacillota	Lachnospiraceae	*Eisenbergiella*	*Eisenbergiella massiliensis*
Bacillota	Lachnospiraceae	*Eisenbergiella*	*Eisenbergiella tayi*
Bacillota	Anaerovoracaceae	*Emergencia*	*Emergencia timonensis*
Bacillota	Lachnospiraceae	*Enterocloster*	*Enterocloster aldenensis*
Bacillota	Lachnospiraceae	*Enterocloster*	*Enterocloster asparagiformis*
Bacillota	Lachnospiraceae	*Enterocloster*	*Enterocloster bolteae*
Bacillota	Lachnospiraceae	*Enterocloster*	*Enterocloster citroniae*
Bacillota	Lachnospiraceae	*Enterocloster*	*Enterocloster clostridioformis*
Bacillota	Enterococcaceae	*Enterococcus*	*Enterococcus alcedinis*
Bacillota	Enterococcaceae	*Enterococcus*	*Enterococcus aquimarinus*
Bacillota	Enterococcaceae	*Enterococcus*	*Enterococcus asini*
Bacillota	Enterococcaceae	*Enterococcus*	*Enterococcus avium*
Bacillota	Enterococcaceae	*Enterococcus*	*Enterococcus bulliens*
Bacillota	Enterococcaceae	*Enterococcus*	*Enterococcus camelliae*
Bacillota	Enterococcaceae	*Enterococcus*	*Enterococcus canintestini*
Bacillota	Enterococcaceae	*Enterococcus*	*Enterococcus canis*
Bacillota	Enterococcaceae	*Enterococcus*	*Enterococcus casseliflavus*
Bacillota	Enterococcaceae	*Enterococcus*	*Enterococcus cecorum*
Bacillota	Enterococcaceae	*Enterococcus*	*Enterococcus columbae*
Bacillota	Enterococcaceae	*Enterococcus*	*Enterococcus crotali*
Bacillota	Enterococcaceae	*Enterococcus*	*Enterococcus devriesei*
Bacillota	Enterococcaceae	*Enterococcus*	*Enterococcus diestrammenae*
Bacillota	Enterococcaceae	*Enterococcus*	*Enterococcus dispar*

Bacillota	Enterococcaceae	*Enterococcus*	*Enterococcus durans*
Bacillota	Enterococcaceae	*Enterococcus*	*Enterococcus eurekensis*
Bacillota	Enterococcaceae	*Enterococcus*	*Enterococcus faecalis*
Bacillota	Enterococcaceae	*Enterococcus*	*Enterococcus faecium*
Bacillota	Enterococcaceae	*Enterococcus*	*Enterococcus gallinarum*
Bacillota	Enterococcaceae	*Enterococcus*	*Enterococcus gilvus*
Bacillota	Enterococcaceae	*Enterococcus*	*Enterococcus haemoperoxidus*
Bacillota	Enterococcaceae	*Enterococcus*	*Enterococcus hermanniensis*
Bacillota	Enterococcaceae	*Enterococcus*	*Enterococcus hirae*
Bacillota	Enterococcaceae	*Enterococcus*	*Enterococcus italicus*
Bacillota	Enterococcaceae	*Enterococcus*	*Enterococcus lactis*
Bacillota	Enterococcaceae	*Enterococcus*	*Enterococcus lemanii*
Bacillota	Enterococcaceae	*Enterococcus*	*Enterococcus malodoratus*
Bacillota	Enterococcaceae	*Enterococcus*	*Enterococcus massiliensis*
Bacillota	Enterococcaceae	*Enterococcus*	*Enterococcus moraviensis*
Bacillota	Enterococcaceae	*Enterococcus*	*Enterococcus mundtii*
Bacillota	Enterococcaceae	*Enterococcus*	*Enterococcus olivae*
Bacillota	Enterococcaceae	*Enterococcus*	*Enterococcus pallens*
Bacillota	Enterococcaceae	*Enterococcus*	*Enterococcus phoeniculicola*
Bacillota	Enterococcaceae	*Enterococcus*	*Enterococcus plantarum*
Bacillota	Enterococcaceae	*Enterococcus*	*Enterococcus pseudoavium*
Bacillota	Enterococcaceae	*Enterococcus*	*Enterococcus raffinosus*
Bacillota	Enterococcaceae	*Enterococcus*	*Enterococcus ratti*
Bacillota	Enterococcaceae	*Enterococcus*	*Enterococcus rivorum*
Bacillota	Enterococcaceae	*Enterococcus*	*Enterococcus rotai*
Bacillota	Enterococcaceae	*Enterococcus*	*Enterococcus saccharolyticus*
Bacillota	Enterococcaceae	*Enterococcus*	*Enterococcus saigonensis*
Bacillota	Enterococcaceae	*Enterococcus*	*Enterococcus silesiacus*

Bacillota	Enterococcaceae	*Enterococcus*	*Enterococcus sulfureus*
Bacillota	Enterococcaceae	*Enterococcus*	*Enterococcus termitis*
Bacillota	Enterococcaceae	*Enterococcus*	*Enterococcus thailandicus*
Bacillota	Enterococcaceae	*Enterococcus*	*Enterococcus ureasiticus*
Bacillota	Enterococcaceae	*Enterococcus*	*Enterococcus ureilyticus*
Bacillota	Enterococcaceae	*Enterococcus*	*Enterococcus viikkiensis*
Bacillota	Enterococcaceae	*Enterococcus*	*Enterococcus villorum*
Bacillota	Enterococcaceae	*Enterococcus*	*Enterococcus wangshanyuanii*
Bacillota	Enterococcaceae	*Enterococcus*	*Enterococcus xiangfangensis*
Bacillota	Oscillospiraceae	*Ercella*	*Ercella succinigenes*
Bacillota	Aerococcaceae	*Eremococcus*	*Eremococcus coleocola*
Bacillota	Erysipelatoclostridiaceae	*Erysipelatoclostridium*	*Erysipelatoclostridium ramosum*
Bacillota	Erysipelotrichaceae	*Erysipelothrix*	*Erysipelothrix inopinata*
Bacillota	Erysipelotrichaceae	*Erysipelothrix*	*Erysipelothrix larvae*
Bacillota	Ethanoligenenaceae	*Ethanoligenens*	*Ethanoligenens harbinense*
Bacillota	Eubacteriaceae	*Eubacterium*	*Eubacterium barkeri*
Bacillota	Eubacteriaceae	*Eubacterium*	*Eubacterium brachy*
Bacillota	Eubacteriaceae	*Eubacterium*	*Eubacterium callanderi*
Bacillota	Eubacteriaceae	*Eubacterium*	*Eubacterium coprostanoligenes*
Bacillota	Eubacteriaceae	*Eubacterium*	*Eubacterium eligens*
Bacillota	Eubacteriaceae	*Eubacterium*	*Eubacterium infirmum*
Bacillota	Eubacteriaceae	*Eubacterium*	*Eubacterium limosum*
Bacillota	Eubacteriaceae	*Eubacterium*	*Eubacterium minutum*
Bacillota	Eubacteriaceae	*Eubacterium*	*Eubacterium multiforme*
Bacillota	Eubacteriaceae	*Eubacterium*	*Eubacterium nodatum*
Bacillota	Eubacteriaceae	*Eubacterium*	*Eubacterium oxidoreducens*
Bacillota	Eubacteriaceae	*Eubacterium*	*Eubacterium pyruvativorans*
Bacillota	Eubacteriaceae	*Eubacterium*	*Eubacterium ramulus*

Bacillota	Eubacteriaceae	*Eubacterium*	*Eubacterium rectale*
Bacillota	Eubacteriaceae	*Eubacterium*	*Eubacterium ruminantium*
Bacillota	Eubacteriaceae	*Eubacterium*	*Eubacterium siraeum*
Bacillota	Eubacteriaceae	*Eubacterium*	*Eubacterium sulci*
Bacillota	Eubacteriaceae	*Eubacterium*	*Eubacterium tenue*
Bacillota	Eubacteriaceae	*Eubacterium*	*Eubacterium uniforme*
Bacillota	Eubacteriaceae	*Eubacterium*	*Eubacterium ventriosum*
Bacillota	Eubacteriaceae	*Eubacterium*	*Eubacterium xylanophilum*
Bacillota	Bacillaceae	*Evansella*	*Evansella cellulosilytica*
Bacillota	Bacillaceae	*Evansella*	*Evansella vedderi*
Bacillota	Exiguobacteraceae	*Exiguobacterium*	*Exiguobacterium acetylicum*
Bacillota	Exiguobacteraceae	*Exiguobacterium*	*Exiguobacterium antarcticum*
Bacillota	Exiguobacteraceae	*Exiguobacterium*	*Exiguobacterium artemiae*
Bacillota	Exiguobacteraceae	*Exiguobacterium*	*Exiguobacterium aurantiacum*
Bacillota	Exiguobacteraceae	*Exiguobacterium*	*Exiguobacterium soli*
Bacillota	Exiguobacteraceae	*Exiguobacterium*	*Exiguobacterium undae*
Bacillota	Incertae	*Exilispira*	*Exilispira thermophila*
Bacillota	Lachnospiraceae	*Extibacter*	*Extibacter muris*
Bacillota	Aerococcaceae	*Facklamia*	*Facklamia hominis*
Bacillota	Aerococcaceae	*Facklamia*	*Facklamia languida*
Bacillota	Aerococcaceae	*Facklamia*	*Facklamia miroungae*
Bacillota	Aerococcaceae	*Facklamia*	*Facklamia sourekii*
Bacillota	Aerococcaceae	*Facklamia*	*Facklamia tabacinasalis*
Bacillota	Ruminococcaceae	*Faecalibacterium*	*Faecalibacterium prausnitzii*
Bacillota	Lachnospiraceae	*Faecalicatena*	*Faecalicatena contorta*
Bacillota	Lachnospiraceae	*Faecalicatena*	*Faecalicatena fissicatena*
Bacillota	Lachnospiraceae	*Faecalicatena*	*Faecalicatena orotica*
Bacillota	Lachnospiraceae	*Faecalimonas*	*Faecalimonas umbilicata*

Bacillota	Clostridiaceae	*Falcatimonas*	*Falcatimonas natans*
Bacillota	Bacillaceae	*Falsibacillus*	*Falsibacillus albus*
Bacillota	Bacillaceae	*Falsibacillus*	*Falsibacillus pallidus*
Bacillota	Bacillaceae	*Fermentibacillus*	*Fermentibacillus polygoni*
Bacillota	Caloramatoraceae	*Fervidicella*	*Fervidicella metallireducens*
Bacillota	Fictibacillaceae	*Fictibacillus*	*Fictibacillus aquaticus*
Bacillota	Fictibacillaceae	*Fictibacillus*	*Fictibacillus arsenicus*
Bacillota	Fictibacillaceae	*Fictibacillus*	*Fictibacillus barbaricus*
Bacillota	Fictibacillaceae	*Fictibacillus*	*Fictibacillus gelatini*
Bacillota	Fictibacillaceae	*Fictibacillus*	*Fictibacillus halophilus*
Bacillota	Fictibacillaceae	*Fictibacillus*	*Fictibacillus iocasae*
Bacillota	Fictibacillaceae	*Fictibacillus*	*Fictibacillus phosphorivorans*
Bacillota	Fictibacillaceae	*Fictibacillus*	*Fictibacillus rigui*
Bacillota	Planococcaceae	*Filibacter*	*Filibacter limicola*
Bacillota	Oscillospiraceae	*Flavonifractor*	*Flavonifractor plautii*
Bacillota	Streptococcaceae	*Floricoccus*	*Floricoccus penangensis*
Bacillota	Streptococcaceae	*Floricoccus*	*Floricoccus tropicus*
Bacillota	Incertae	*Fodinicola*	*Fodinicola feengrottensis*
Bacillota	Paenibacillaceae	*Fontibacillus*	*Fontibacillus panacisegetis*
Bacillota	Paenibacillaceae	*Fontibacillus*	*Fontibacillus solani*
Bacillota	Caloramatoraceae	*Fonticella*	*Fonticella tunisiensis*
Bacillota	Ruminococcaceae	*Fournierella*	*Fournierella massiliensis*
Bacillota	Bacillaceae	*Fredinandcohnia*	*Fredinandcohnia humi*
Bacillota	Lachnospiraceae	*Frisingicoccus*	*Frisingicoccus caecimuris*
Bacillota	Lactobacillaceae	*Fructilactobacillus*	*Fructilactobacillus florum*
Bacillota	Lactobacillaceae	*Fructilactobacillus*	*Fructilactobacillus fructivorans*
Bacillota	Lactobacillaceae	*Fructilactobacillus*	*Fructilactobacillus ixorae*
Bacillota	Lactobacillaceae	*Fructilactobacillus*	*Fructilactobacillus lindneri*

Bacillota	Lactobacillaceae	*Fructilactobacillus*	*Fructilactobacillus sanfranciscensis*
Bacillota	Lactobacillaceae	*Fructilactobacillus*	*Fructilactobacillus vespulae*
Bacillota	Lactobacillaceae	*Fructobacillus*	*Fructobacillus durionis*
Bacillota	Lactobacillaceae	*Fructobacillus*	*Fructobacillus ficulneus*
Bacillota	Lactobacillaceae	*Fructobacillus*	*Fructobacillus fructosus*
Bacillota	Lactobacillaceae	*Fructobacillus*	*Fructobacillus pseudoficulneus*
Bacillota	Lactobacillaceae	*Fructobacillus*	*Fructobacillus tropaeoli*
Bacillota	Halobacteroidaceae	*Fuchsiella*	*Fuchsiella ferrireducens*
Bacillota	Lactobacillaceae	*Furfurilactobacillus*	*Furfurilactobacillus curtus*
Bacillota	Lactobacillaceae	*Furfurilactobacillus*	*Furfurilactobacillus rossiae*
Bacillota	Lactobacillaceae	*Furfurilactobacillus*	*Furfurilactobacillus siliginis*
Bacillota	Incertae	*Fusibacter*	*Fusibacter fontis*
Bacillota	Incertae	*Fusibacter*	*Fusibacter paucivorans*
Bacillota	Lachnospiraceae	*Fusicatenibacter*	*Fusicatenibacter saccharivorans*
Bacillota	Gemellaceae	*Gemella*	*Gemella bergeri*
Bacillota	Gemellaceae	*Gemella*	*Gemella cuniculi*
Bacillota	Gemellaceae	*Gemella*	*Gemella haemolysans*
Bacillota	Gemellaceae	*Gemella*	*Gemella morbillorum*
Bacillota	Gemellaceae	*Gemella*	*Gemella palaticanis*
Bacillota	Gemellaceae	*Gemella*	*Gemella sanguinis*
Bacillota	Ruminococcaceae	*Gemmiger*	*Gemmiger formicilis*
Bacillota	Anoxybacillaceae	*Geobacillus*	*Geobacillus kaustophilus*
Bacillota	Anoxybacillaceae	*Geobacillus*	*Geobacillus stearothermophilus*
Bacillota	Anoxybacillaceae	*Geobacillus*	*Geobacillus subterraneus*
Bacillota	Thermotaleaceae	*Geosporobacter*	*Geosporobacter subterraneus*
Bacillota	Aerococcaceae	*Globicatella*	*Globicatella sanguinis*
Bacillota	Aerococcaceae	*Globicatella*	*Globicatella sulfidifaciens*
Bacillota	Lachnospiraceae	*Gluceribacter*	*Gluceribacter canis*

Bacillota	Paenibacillaceae	*Gorillibacterium*	*Gorillibacterium massiliense*
Bacillota	Bacillaceae	*Gottfriedia*	*Gottfriedia luciferensis*
Bacillota	Bacillaceae	*Gottfriedia*	*Gottfriedia solisilvae*
Bacillota	Gottschalkiaceae	*Gottschalkia*	*Gottschalkia acidurici*
Bacillota	Gottschalkiaceae	*Gottschalkia*	*Gottschalkia purinilytica*
Bacillota	Amphibacillaceae	*Gracilibacillus*	*Gracilibacillus timonensis*
Bacillota	Amphibacillaceae	*Gracilibacillus*	*Gracilibacillus xinjiangensis*
Bacillota	Syntrophobotulaceae	*Gracilibacter*	*Gracilibacter thermotolerans*
Bacillota	Aerococcaceae	*Granulicatella*	*Granulicatella adiacens*
Bacillota	Aerococcaceae	*Granulicatella*	*Granulicatella balaenopterae*
Bacillota	Aerococcaceae	*Granulicatella*	*Granulicatella elegans*
Bacillota	Alkalibacillaceae	*Halalkalibacillus*	*Halalkalibacillus halophilus*
Bacillota	Halobacillaceae	*Halobacillus*	*Halobacillus andaensis*
Bacillota	Halobacillaceae	*Halobacillus*	*Halobacillus dabanensis*
Bacillota	Halobacillaceae	*Halobacillus*	*Halobacillus faecis*
Bacillota	Halobacillaceae	*Halobacillus*	*Halobacillus halophilus*
Bacillota	Halobacillaceae	*Halobacillus*	*Halobacillus sediminis*
Bacillota	Halobacillaceae	*Halobacillus*	*Halobacillus yeomjeoni*
Bacillota	Clostridiaceae	*Haloimpatiens*	*Haloimpatiens lingqiaonensis*
Bacillota	Amphibacillaceae	*Halolactibacillus*	*Halolactibacillus halophilus*
Bacillota	Amphibacillaceae	*Halolactibacillus*	*Halolactibacillus miurensis*
Bacillota	Haloplasmataceae	*Haloplasma*	*Haloplasma contractile*
Bacillota	Acholeplasmataceae	*Haploplasma*	*Haploplasma axanthum*
Bacillota	Ruminococcaceae	*Harryflintia*	*Harryflintia acetispora*
Bacillota	Clostridiaceae	*Hathewaya*	*Hathewaya histolytica*
Bacillota	Clostridiaceae	*Hathewaya*	*Hathewaya limosa*
Bacillota	Clostridiaceae	*Hathewaya*	*Hathewaya proteolytica*
Bacillota	Heliobacteriaceae	*Heliobacillus*	*Heliobacillus mobilis*

Bacillota	Heliobacteriaceae	*Heliophilum*	*Heliophilum fasciatum*
Bacillota	Heliobacteriaceae	*Heliorestis*	*Heliorestis acidaminivorans*
Bacillota	Heliobacteriaceae	*Heliorestis*	*Heliorestis baculata*
Bacillota	Lachnospiraceae	*Herbinix*	*Herbinix hemicellulosilytica*
Bacillota	Lachnospiraceae	*Herbinix*	*Herbinix luporum*
Bacillota	Lachnospiraceae	*Hespellia*	*Hespellia porcina*
Bacillota	Lachnospiraceae	*Hespellia*	*Hespellia stercorisuis*
Bacillota	Bacillaceae	*Heyndrickxia*	*Heyndrickxia oleronia*
Bacillota	Bacillaceae	*Heyndrickxia*	*Heyndrickxia sporothermodurans*
Bacillota	Erysipelotrichaceae	*Holdemania*	*Holdemania massiliensis*
Bacillota	Lactobacillaceae	*Holzapfelia*	*Holzapfelia floricola*
Bacillota	Eubacteriaceae	*Howardella*	*Howardella ureilytica*
Bacillota	Acetivibrionaceae	*Hungateiclostridium*	*Hungateiclostridium aldrichii*
Bacillota	Acetivibrionaceae	*Hungateiclostridium*	*Hungateiclostridium alkalicellulosi*
Bacillota	Acetivibrionaceae	*Hungateiclostridium*	*Hungateiclostridium cellulolyticum*
Bacillota	Acetivibrionaceae	*Hungateiclostridium*	*Hungateiclostridium clariflavum*
Bacillota	Acetivibrionaceae	*Hungateiclostridium*	*Hungateiclostridium saccincola*
Bacillota	Acetivibrionaceae	*Hungateiclostridium*	*Hungateiclostridium straminisolvens*
Bacillota	Acetivibrionaceae	*Hungateiclostridium*	*Hungateiclostridium thermocellum*
Bacillota	Lachnospiraceae	*Hungatella*	*Hungatella effluvii*
Bacillota	Lachnospiraceae	*Hungatella*	*Hungatella hathewayi*
Bacillota	Lachnospiraceae	*Hungatella*	*Hungatella xylanolytica*
Bacillota	UBA8346	*Hydrogenispora*	*Hydrogenispora ethanolica*
Bacillota	Ruminococcaceae	*Hydrogenoanaerobacterium*	*Hydrogenoanaerobacterium saccharovorans*
Bacillota	Aerococcaceae	*Ignavigranum*	*Ignavigranum ruoffiae*
Bacillota	Incertae	*Ihubacter*	*Ihubacter massiliensis*
Bacillota	Butyricicoccaceae	*Intestinibacillus*	*Intestinibacillus massiliensis*
Bacillota	Peptostreptococcaceae	*Intestinibacter*	*Intestinibacter bartlettii*

Bacillota	Oscillospiraceae	*Intestinimonas*	*Intestinimonas butyriciproducens*
Bacillota	Eubacteriaceae	*Irregularibacter*	*Irregularibacter muris*
Bacillota	Carnobacteriaceae	*Isobaculum*	*Isobaculum melis*
Bacillota	Aerococcaceae	*Jeotgalibaca*	*Jeotgalibaca arthritidis*
Bacillota	Aerococcaceae	*Jeotgalibaca*	*Jeotgalibaca dankookensis*
Bacillota	Aerococcaceae	*Jeotgalibaca*	*Jeotgalibaca porci*
Bacillota	Jeotgalibacillaceae	*Jeotgalibacillus*	*Jeotgalibacillus alimentarius*
Bacillota	Jeotgalibacillaceae	*Jeotgalibacillus*	*Jeotgalibacillus campisalis*
Bacillota	Jeotgalibacillaceae	*Jeotgalibacillus*	*Jeotgalibacillus malaysiensis*
Bacillota	Jeotgalibacillaceae	*Jeotgalibacillus*	*Jeotgalibacillus marinus*
Bacillota	Jeotgalibacillaceae	*Jeotgalibacillus*	*Jeotgalibacillus salarius*
Bacillota	Jeotgalibacillaceae	*Jeotgalibacillus*	*Jeotgalibacillus soli*
Bacillota	Jeotgalibacillaceae	*Jeotgalibacillus*	*Jeotgalibacillus terrae*
Bacillota	Salinicoccaceae	*Jeotgalicoccus*	*Jeotgalicoccus halotolerans*
Bacillota	Salinicoccaceae	*Jeotgalicoccus*	*Jeotgalicoccus huakuii*
Bacillota	Salinicoccaceae	*Jeotgalicoccus*	*Jeotgalicoccus nanhaiensis*
Bacillota	Salinicoccaceae	*Jeotgalicoccus*	*Jeotgalicoccus psychrophilus*
Bacillota	Salinicoccaceae	*Jeotgalicoccus*	*Jeotgalicoccus schoeneichii*
Bacillota	Lachnospiraceae	*Johnsonella*	*Johnsonella ignava*
Bacillota	Erysipelatoclostridiaceae	*Kandleria*	*Kandleria vitulina*
Bacillota	Tepidimicrobiaceae	*Keratinibaculum*	*Keratinibaculum paraultunense*
Bacillota	Lachnospiraceae	*Kineothrix*	*Kineothrix alysoides*
Bacillota	Planococcaceae	*Kurthia*	*Kurthia gibsonii*
Bacillota	Planococcaceae	*Kurthia*	*Kurthia massiliensis*
Bacillota	Planococcaceae	*Kurthia*	*Kurthia populi*
Bacillota	Planococcaceae	*Kurthia*	*Kurthia senegalensis*
Bacillota	Planococcaceae	*Kurthia*	*Kurthia sibirica*
Bacillota	Planococcaceae	*Kurthia*	*Kurthia zopfii*

Bacillota	Lachnospiraceae	*Lachnoanaerobaculum*	*Lachnoanaerobaculum gingivalis*
Bacillota	Lachnospiraceae	*Lachnoclostridium*	*Lachnoclostridium pacaense*
Bacillota	Lachnospiraceae	*Lachnoclostridium*	*Lachnoclostridium phytofermentans*
Bacillota	Lachnospiraceae	*Lachnospira*	*Lachnospira multipara*
Bacillota	Lachnospiraceae	*Lachnotalea*	*Lachnotalea glycerini*
Bacillota	Lachnospiraceae	*Lacrimispora*	*Lacrimispora aerotolerans*
Bacillota	Lachnospiraceae	*Lacrimispora*	*Lacrimispora algidixylanolytica*
Bacillota	Lachnospiraceae	*Lacrimispora*	*Lacrimispora amygdalina*
Bacillota	Lachnospiraceae	*Lacrimispora*	*Lacrimispora indolis*
Bacillota	Lachnospiraceae	*Lacrimispora*	*Lacrimispora saccharolytica*
Bacillota	Lachnospiraceae	*Lacrimispora*	*Lacrimispora sphenoides*
Bacillota	Lactobacillaceae	*Lacticaseibacillus*	*Lacticaseibacillus brantae*
Bacillota	Lactobacillaceae	*Lacticaseibacillus*	*Lacticaseibacillus camelliae*
Bacillota	Lactobacillaceae	*Lacticaseibacillus*	*Lacticaseibacillus casei*
Bacillota	Lactobacillaceae	*Lacticaseibacillus*	*Lacticaseibacillus hulanensis*
Bacillota	Lactobacillaceae	*Lacticaseibacillus*	*Lacticaseibacillus manihotivorans*
Bacillota	Lactobacillaceae	*Lacticaseibacillus*	*Lacticaseibacillus nasuensis*
Bacillota	Lactobacillaceae	*Lacticaseibacillus*	*Lacticaseibacillus pantheris*
Bacillota	Lactobacillaceae	*Lacticaseibacillus*	*Lacticaseibacillus paracasei*
Bacillota	Lactobacillaceae	*Lacticaseibacillus*	*Lacticaseibacillus porcinae*
Bacillota	Lactobacillaceae	*Lacticaseibacillus*	*Lacticaseibacillus rhamnosus*
Bacillota	Lactobacillaceae	*Lacticaseibacillus*	*Lacticaseibacillus saniviri*
Bacillota	Lactobacillaceae	*Lacticaseibacillus*	*Lacticaseibacillus sharpeae*
Bacillota	Lactobacillaceae	*Lacticaseibacillus*	*Lacticaseibacillus songhuajiangensis*
Bacillota	Lactobacillaceae	*Lacticaseibacillus*	*Lacticaseibacillus thailandensis*
Bacillota	Lactobacillaceae	*Lacticaseibacillus*	*Lacticaseibacillus zeae*
Bacillota	Carnobacteriaceae	*Lacticigenium*	*Lacticigenium naphtae*
Bacillota	Carnobacteriaceae	*Lacticigenium*	*Lacticigenium naphthae*

Bacillota	Lactobacillaceae	*Lactiplantibacillus*	*Lactiplantibacillus argentoratensis*
Bacillota	Lactobacillaceae	*Lactiplantibacillus*	*Lactiplantibacillus fabifermentans*
Bacillota	Lactobacillaceae	*Lactiplantibacillus*	*Lactiplantibacillus herbarum*
Bacillota	Lactobacillaceae	*Lactiplantibacillus*	*Lactiplantibacillus mudanjiangensis*
Bacillota	Lactobacillaceae	*Lactiplantibacillus*	*Lactiplantibacillus paraplantarum*
Bacillota	Lactobacillaceae	*Lactiplantibacillus*	*Lactiplantibacillus pentosus*
Bacillota	Lactobacillaceae	*Lactiplantibacillus*	*Lactiplantibacillus plajomi*
Bacillota	Lactobacillaceae	*Lactiplantibacillus*	*Lactiplantibacillus plantarum*
Bacillota	Lactobacillaceae	*Lactobacillus*	*Lactobacillus acetotolerans*
Bacillota	Lactobacillaceae	*Lactobacillus*	*Lactobacillus acidifarinae*
Bacillota	Lactobacillaceae	*Lactobacillus*	*Lactobacillus acidipiscis*
Bacillota	Lactobacillaceae	*Lactobacillus*	*Lactobacillus acidophilus*
Bacillota	Lactobacillaceae	*Lactobacillus*	*Lactobacillus agilis*
Bacillota	Lactobacillaceae	*Lactobacillus*	*Lactobacillus algidus*
Bacillota	Lactobacillaceae	*Lactobacillus*	*Lactobacillus allii*
Bacillota	Lactobacillaceae	*Lactobacillus*	*Lactobacillus amylolyticus*
Bacillota	Lactobacillaceae	*Lactobacillus*	*Lactobacillus amylophilus*
Bacillota	Lactobacillaceae	*Lactobacillus*	*Lactobacillus animalis*
Bacillota	Lactobacillaceae	*Lactobacillus*	*Lactobacillus antri*
Bacillota	Lactobacillaceae	*Lactobacillus*	*Lactobacillus apinorum*
Bacillota	Lactobacillaceae	*Lactobacillus*	*Lactobacillus apodemi*
Bacillota	Lactobacillaceae	*Lactobacillus*	*Lactobacillus aquaticus*
Bacillota	Lactobacillaceae	*Lactobacillus*	*Lactobacillus aviarius*
Bacillota	Lactobacillaceae	*Lactobacillus*	*Lactobacillus backii*
Bacillota	Lactobacillaceae	*Lactobacillus*	*Lactobacillus bifermentans*
Bacillota	Lactobacillaceae	*Lactobacillus*	*Lactobacillus brantae*
Bacillota	Lactobacillaceae	*Lactobacillus*	*Lactobacillus cacaonum*
Bacillota	Lactobacillaceae	*Lactobacillus*	*Lactobacillus camelliae*

Bacillota	Lactobacillaceae	*Lactobacillus*	*Lactobacillus capillatus*
Bacillota	Lactobacillaceae	*Lactobacillus*	*Lactobacillus casei*
Bacillota	Lactobacillaceae	*Lactobacillus*	*Lactobacillus caviae*
Bacillota	Lactobacillaceae	*Lactobacillus*	*Lactobacillus coleohominis*
Bacillota	Lactobacillaceae	*Lactobacillus*	*Lactobacillus colini*
Bacillota	Lactobacillaceae	*Lactobacillus*	*Lactobacillus composti*
Bacillota	Lactobacillaceae	*Lactobacillus*	*Lactobacillus concavus*
Bacillota	Lactobacillaceae	*Lactobacillus*	*Lactobacillus coryniformis*
Bacillota	Lactobacillaceae	*Lactobacillus*	*Lactobacillus crispatus*
Bacillota	Lactobacillaceae	*Lactobacillus*	*Lactobacillus curieae*
Bacillota	Lactobacillaceae	*Lactobacillus*	*Lactobacillus curtus*
Bacillota	Lactobacillaceae	*Lactobacillus*	*Lactobacillus curvatus*
Bacillota	Lactobacillaceae	*Lactobacillus*	*Lactobacillus delbrueckii*
Bacillota	Lactobacillaceae	*Lactobacillus*	*Lactobacillus dextrinicus*
Bacillota	Lactobacillaceae	*Lactobacillus*	*Lactobacillus equi*
Bacillota	Lactobacillaceae	*Lactobacillus*	*Lactobacillus equicursoris*
Bacillota	Lactobacillaceae	*Lactobacillus*	*Lactobacillus equigenerosi*
Bacillota	Lactobacillaceae	*Lactobacillus*	*Lactobacillus fabifermentans*
Bacillota	Lactobacillaceae	*Lactobacillus*	*Lactobacillus faecis*
Bacillota	Lactobacillaceae	*Lactobacillus*	*Lactobacillus farciminis*
Bacillota	Lactobacillaceae	*Lactobacillus*	*Lactobacillus farraginis*
Bacillota	Lactobacillaceae	*Lactobacillus*	*Lactobacillus fermentum*
Bacillota	Lactobacillaceae	*Lactobacillus*	*Lactobacillus floricola*
Bacillota	Lactobacillaceae	*Lactobacillus*	*Lactobacillus fructivorans*
Bacillota	Lactobacillaceae	*Lactobacillus*	*Lactobacillus fuchuensis*
Bacillota	Lactobacillaceae	*Lactobacillus*	*Lactobacillus furfuricola*
Bacillota	Lactobacillaceae	*Lactobacillus*	*Lactobacillus futsaii*
Bacillota	Lactobacillaceae	*Lactobacillus*	*Lactobacillus gallinarum*

Bacillota	Lactobacillaceae	*Lactobacillus*	*Lactobacillus gasseri*
Bacillota	Lactobacillaceae	*Lactobacillus*	*Lactobacillus gastricus*
Bacillota	Lactobacillaceae	*Lactobacillus*	*Lactobacillus ginsenosidimutans*
Bacillota	Lactobacillaceae	*Lactobacillus*	*Lactobacillus graminis*
Bacillota	Lactobacillaceae	*Lactobacillus*	*Lactobacillus hammesii*
Bacillota	Lactobacillaceae	*Lactobacillus*	*Lactobacillus hamsteri*
Bacillota	Lactobacillaceae	*Lactobacillus*	*Lactobacillus harbinensis*
Bacillota	Lactobacillaceae	*Lactobacillus*	*Lactobacillus hayakitensis*
Bacillota	Lactobacillaceae	*Lactobacillus*	*Lactobacillus helsingborgensis*
Bacillota	Lactobacillaceae	*Lactobacillus*	*Lactobacillus helveticus*
Bacillota	Lactobacillaceae	*Lactobacillus*	*Lactobacillus hilgardii*
Bacillota	Lactobacillaceae	*Lactobacillus*	*Lactobacillus hokkaidonensis*
Bacillota	Lactobacillaceae	*Lactobacillus*	*Lactobacillus hominis*
Bacillota	Lactobacillaceae	*Lactobacillus*	*Lactobacillus homohiochii*
Bacillota	Lactobacillaceae	*Lactobacillus*	*Lactobacillus iners*
Bacillota	Lactobacillaceae	*Lactobacillus*	*Lactobacillus insicii*
Bacillota	Lactobacillaceae	*Lactobacillus*	*Lactobacillus intestinalis*
Bacillota	Lactobacillaceae	*Lactobacillus*	*Lactobacillus iwatensis*
Bacillota	Lactobacillaceae	*Lactobacillus*	*Lactobacillus ixorae*
Bacillota	Lactobacillaceae	*Lactobacillus*	*Lactobacillus jensenii*
Bacillota	Lactobacillaceae	*Lactobacillus*	*Lactobacillus johnsonii*
Bacillota	Lactobacillaceae	*Lactobacillus*	*Lactobacillus kalixensis*
Bacillota	Lactobacillaceae	*Lactobacillus*	*Lactobacillus kefiranofaciens*
Bacillota	Lactobacillaceae	*Lactobacillus*	*Lactobacillus kimchiensis*
Bacillota	Lactobacillaceae	*Lactobacillus*	*Lactobacillus kisonensis*
Bacillota	Lactobacillaceae	*Lactobacillus*	*Lactobacillus kitasatonis*
Bacillota	Lactobacillaceae	*Lactobacillus*	*Lactobacillus kunkeei*
Bacillota	Lactobacillaceae	*Lactobacillus*	*Lactobacillus leichmannii*

Bacillota	Lactobacillaceae	*Lactobacillus*	*Lactobacillus lindneri*
Bacillota	Lactobacillaceae	*Lactobacillus*	*Lactobacillus malefermentans*
Bacillota	Lactobacillaceae	*Lactobacillus*	*Lactobacillus mali*
Bacillota	Lactobacillaceae	*Lactobacillus*	*Lactobacillus manihotivorans*
Bacillota	Lactobacillaceae	*Lactobacillus*	*Lactobacillus mellifer*
Bacillota	Lactobacillaceae	*Lactobacillus*	*Lactobacillus mellis*
Bacillota	Lactobacillaceae	*Lactobacillus*	*Lactobacillus melliventris*
Bacillota	Lactobacillaceae	*Lactobacillus*	*Lactobacillus mixtipabuli*
Bacillota	Lactobacillaceae	*Lactobacillus*	*Lactobacillus modestisalitolerans*
Bacillota	Lactobacillaceae	*Lactobacillus*	*Lactobacillus mucosae*
Bacillota	Lactobacillaceae	*Lactobacillus*	*Lactobacillus mudanjiangensis*
Bacillota	Lactobacillaceae	*Lactobacillus*	*Lactobacillus murinus*
Bacillota	Lactobacillaceae	*Lactobacillus*	*Lactobacillus nagelii*
Bacillota	Lactobacillaceae	*Lactobacillus*	*Lactobacillus namurensis*
Bacillota	Lactobacillaceae	*Lactobacillus*	*Lactobacillus nantensis*
Bacillota	Lactobacillaceae	*Lactobacillus*	*Lactobacillus nasuensis*
Bacillota	Lactobacillaceae	*Lactobacillus*	*Lactobacillus nenjiangensis*
Bacillota	Lactobacillaceae	*Lactobacillus*	*Lactobacillus nodensis*
Bacillota	Lactobacillaceae	*Lactobacillus*	*Lactobacillus odoratitofui*
Bacillota	Lactobacillaceae	*Lactobacillus*	*Lactobacillus oeni*
Bacillota	Lactobacillaceae	*Lactobacillus*	*Lactobacillus oligofermentans*
Bacillota	Lactobacillaceae	*Lactobacillus*	*Lactobacillus oris*
Bacillota	Lactobacillaceae	*Lactobacillus*	*Lactobacillus oryzae*
Bacillota	Lactobacillaceae	*Lactobacillus*	*Lactobacillus otakiensis*
Bacillota	Lactobacillaceae	*Lactobacillus*	*Lactobacillus ozensis*
Bacillota	Lactobacillaceae	*Lactobacillus*	*Lactobacillus panis*
Bacillota	Lactobacillaceae	*Lactobacillus*	*Lactobacillus pantheris*
Bacillota	Lactobacillaceae	*Lactobacillus*	*Lactobacillus parabrevis*

Bacillota	Lactobacillaceae	*Lactobacillus*	*Lactobacillus parabuchneri*
Bacillota	Lactobacillaceae	*Lactobacillus*	*Lactobacillus paracasei*
Bacillota	Lactobacillaceae	*Lactobacillus*	*Lactobacillus paracollinoides*
Bacillota	Lactobacillaceae	*Lactobacillus*	*Lactobacillus parafarraginis*
Bacillota	Lactobacillaceae	*Lactobacillus*	*Lactobacillus paralimentarius*
Bacillota	Lactobacillaceae	*Lactobacillus*	*Lactobacillus paraplantarum*
Bacillota	Lactobacillaceae	*Lactobacillus*	*Lactobacillus paucivorans*
Bacillota	Lactobacillaceae	*Lactobacillus*	*Lactobacillus pentosiphilus*
Bacillota	Lactobacillaceae	*Lactobacillus*	*Lactobacillus pentosus*
Bacillota	Lactobacillaceae	*Lactobacillus*	*Lactobacillus plajomi*
Bacillota	Lactobacillaceae	*Lactobacillus*	*Lactobacillus plantarum*
Bacillota	Lactobacillaceae	*Lactobacillus*	*Lactobacillus pobuzihii*
Bacillota	Lactobacillaceae	*Lactobacillus*	*Lactobacillus pontis*
Bacillota	Lactobacillaceae	*Lactobacillus*	*Lactobacillus porcinae*
Bacillota	Lactobacillaceae	*Lactobacillus*	*Lactobacillus rapi*
Bacillota	Lactobacillaceae	*Lactobacillus*	*Lactobacillus reuteri*
Bacillota	Lactobacillaceae	*Lactobacillus*	*Lactobacillus rhamnosus*
Bacillota	Lactobacillaceae	*Lactobacillus*	*Lactobacillus rodentium*
Bacillota	Lactobacillaceae	*Lactobacillus*	*Lactobacillus rogosae*
Bacillota	Lactobacillaceae	*Lactobacillus*	*Lactobacillus ruminis*
Bacillota	Lactobacillaceae	*Lactobacillus*	*Lactobacillus saerimneri*
Bacillota	Lactobacillaceae	*Lactobacillus*	*Lactobacillus sakei*
Bacillota	Lactobacillaceae	*Lactobacillus*	*Lactobacillus salivarius*
Bacillota	Lactobacillaceae	*Lactobacillus*	*Lactobacillus sanfranciscensis*
Bacillota	Lactobacillaceae	*Lactobacillus*	*Lactobacillus saniviri*
Bacillota	Lactobacillaceae	*Lactobacillus*	*Lactobacillus satsumensis*
Bacillota	Lactobacillaceae	*Lactobacillus*	*Lactobacillus secaliphilus*
Bacillota	Lactobacillaceae	*Lactobacillus*	*Lactobacillus selangorensis*

Bacillota	Lactobacillaceae	*Lactobacillus*	*Lactobacillus senioris*
Bacillota	Lactobacillaceae	*Lactobacillus*	*Lactobacillus senmaizukei*
Bacillota	Lactobacillaceae	*Lactobacillus*	*Lactobacillus sharpeae*
Bacillota	Lactobacillaceae	*Lactobacillus*	*Lactobacillus shenzhenensis*
Bacillota	Lactobacillaceae	*Lactobacillus*	*Lactobacillus silagincola*
Bacillota	Lactobacillaceae	*Lactobacillus*	*Lactobacillus siliginis*
Bacillota	Lactobacillaceae	*Lactobacillus*	*Lactobacillus similis*
Bacillota	Lactobacillaceae	*Lactobacillus*	*Lactobacillus songhuajiangensis*
Bacillota	Lactobacillaceae	*Lactobacillus*	*Lactobacillus sucicola*
Bacillota	Lactobacillaceae	*Lactobacillus*	*Lactobacillus suebicus*
Bacillota	Lactobacillaceae	*Lactobacillus*	*Lactobacillus sunkii*
Bacillota	Lactobacillaceae	*Lactobacillus*	*Lactobacillus taiwanensis*
Bacillota	Lactobacillaceae	*Lactobacillus*	*Lactobacillus tucceti*
Bacillota	Lactobacillaceae	*Lactobacillus*	*Lactobacillus ultunensis*
Bacillota	Lactobacillaceae	*Lactobacillus*	*Lactobacillus uvarum*
Bacillota	Lactobacillaceae	*Lactobacillus*	*Lactobacillus vaccinostercus*
Bacillota	Lactobacillaceae	*Lactobacillus*	*Lactobacillus vaginalis*
Bacillota	Lactobacillaceae	*Lactobacillus*	*Lactobacillus versmoldensis*
Bacillota	Lactobacillaceae	*Lactobacillus*	*Lactobacillus vespulae*
Bacillota	Lactobacillaceae	*Lactobacillus*	*Lactobacillus vini*
Bacillota	Lactobacillaceae	*Lactobacillus*	*Lactobacillus wasatchensis*
Bacillota	Lactobacillaceae	*Lactobacillus*	*Lactobacillus zeae*
Bacillota	Lactobacillaceae	*Lactobacillus*	*Lactobacillus zymae*
Bacillota	Streptococcaceae	*Lactococcus*	*Lactococcus chungangensis*
Bacillota	Streptococcaceae	*Lactococcus*	*Lactococcus cremoris*
Bacillota	Streptococcaceae	*Lactococcus*	*Lactococcus formosensis*
Bacillota	Streptococcaceae	*Lactococcus*	*Lactococcus fujiensis*
Bacillota	Streptococcaceae	*Lactococcus*	*Lactococcus garvieae*

Bacillota	Streptococcaceae	*Lactococcus*	*Lactococcus hircilactis*
Bacillota	Streptococcaceae	*Lactococcus*	*Lactococcus lactis*
Bacillota	Streptococcaceae	*Lactococcus*	*Lactococcus laudensis*
Bacillota	Streptococcaceae	*Lactococcus*	*Lactococcus nasutitermitis*
Bacillota	Streptococcaceae	*Lactococcus*	*Lactococcus piscium*
Bacillota	Streptococcaceae	*Lactococcus*	*Lactococcus plantarum*
Bacillota	Streptococcaceae	*Lactococcus*	*Lactococcus raffinolactis*
Bacillota	Streptococcaceae	*Lactococcus*	*Lactococcus taiwanensis*
Bacillota	Lachnospiraceae	*Lactonifactor*	*Lactonifactor longoviformis*
Bacillota	Streptococcaceae	*Lactovum*	*Lactovum miscens*
Bacillota	Lactobacillaceae	*Lapidilactobacillus*	*Lapidilactobacillus concavus*
Bacillota	Lactobacillaceae	*Lapidilactobacillus*	*Lapidilactobacillus dextrinicus*
Bacillota	Lactobacillaceae	*Latilactobacillus*	*Latilactobacillus curvatus*
Bacillota	Lactobacillaceae	*Latilactobacillus*	*Latilactobacillus fuchuensis*
Bacillota	Lactobacillaceae	*Latilactobacillus*	*Latilactobacillus graminis*
Bacillota	Lactobacillaceae	*Latilactobacillus*	*Latilactobacillus sakei*
Bacillota	Oscillospiraceae	*Lawsonibacter*	*Lawsonibacter asaccharolyticus*
Bacillota	Bacillaceae	*Lederbergia*	*Lederbergia galactosidilyticus*
Bacillota	Bacillaceae	*Lederbergia*	*Lederbergia lentus*
Bacillota	Bacillaceae	*Lederbergia*	*Lederbergia panacisoli*
Bacillota	Bacillaceae	*Lederbergia*	*Lederbergia ruris*
Bacillota	Bacillaceae	*Lederbergia*	*Lederbergia wuyishanensis*
Bacillota	Amphibacillaceae	*Lentibacillus*	*Lentibacillus alimentarius*
Bacillota	Amphibacillaceae	*Lentibacillus*	*Lentibacillus salicampi*
Bacillota	Amphibacillaceae	*Lentibacillus*	*Lentibacillus salis*
Bacillota	Lactobacillaceae	*Lentilactobacillus*	*Lentilactobacillus buchneri*
Bacillota	Lactobacillaceae	*Lentilactobacillus*	*Lentilactobacillus curieae*
Bacillota	Lactobacillaceae	*Lentilactobacillus*	*Lentilactobacillus diolivorans*

Bacillota	Lactobacillaceae	*Lentilactobacillus*	*Lentilactobacillus farraginis*
Bacillota	Lactobacillaceae	*Lentilactobacillus*	*Lentilactobacillus hilgardii*
Bacillota	Lactobacillaceae	*Lentilactobacillus*	*Lentilactobacillus kefiri*
Bacillota	Lactobacillaceae	*Lentilactobacillus*	*Lentilactobacillus kisonensis*
Bacillota	Lactobacillaceae	*Lentilactobacillus*	*Lentilactobacillus otakiensis*
Bacillota	Lactobacillaceae	*Lentilactobacillus*	*Lentilactobacillus parabuchneri*
Bacillota	Lactobacillaceae	*Lentilactobacillus*	*Lentilactobacillus parafarraginis*
Bacillota	Lactobacillaceae	*Lentilactobacillus*	*Lentilactobacillus parakefiri*
Bacillota	Lactobacillaceae	*Lentilactobacillus*	*Lentilactobacillus rapi*
Bacillota	Lactobacillaceae	*Lentilactobacillus*	*Lentilactobacillus senioris*
Bacillota	Lactobacillaceae	*Lentilactobacillus*	*Lentilactobacillus sunkii*
Bacillota	Lactobacillaceae	*Leuconostoc*	*Leuconostoc carnosum*
Bacillota	Lactobacillaceae	*Leuconostoc*	*Leuconostoc citreum*
Bacillota	Lactobacillaceae	*Leuconostoc*	*Leuconostoc fallax*
Bacillota	Lactobacillaceae	*Leuconostoc*	*Leuconostoc gelidum*
Bacillota	Lactobacillaceae	*Leuconostoc*	*Leuconostoc holzapfelii*
Bacillota	Lactobacillaceae	*Leuconostoc*	*Leuconostoc inhae*
Bacillota	Lactobacillaceae	*Leuconostoc*	*Leuconostoc kimchii*
Bacillota	Lactobacillaceae	*Leuconostoc*	*Leuconostoc lactis*
Bacillota	Lactobacillaceae	*Leuconostoc*	*Leuconostoc mesenteroides*
Bacillota	Lactobacillaceae	*Leuconostoc*	*Leuconostoc miyukkimchii*
Bacillota	Lactobacillaceae	*Leuconostoc*	*Leuconostoc palmae*
Bacillota	Lactobacillaceae	*Leuconostoc*	*Leuconostoc pseudomesenteroides*
Bacillota	Lactobacillaceae	*Leuconostoc*	*Leuconostoc rapi*
Bacillota	Lactobacillaceae	*Leuconostoc*	*Leuconostoc suionicum*
Bacillota	Lactobacillaceae	*Levilactobacillus*	*Levilactobacillus acidifarinae*
Bacillota	Lactobacillaceae	*Levilactobacillus*	*Levilactobacillus brevis*
Bacillota	Lactobacillaceae	*Levilactobacillus*	*Levilactobacillus cerevisiae*

Bacillota	Lactobacillaceae	*Levilactobacillus*	*Levilactobacillus hammesii*
Bacillota	Lactobacillaceae	*Levilactobacillus*	*Levilactobacillus koreensis*
Bacillota	Lactobacillaceae	*Levilactobacillus*	*Levilactobacillus namurensis*
Bacillota	Lactobacillaceae	*Levilactobacillus*	*Levilactobacillus parabrevis*
Bacillota	Lactobacillaceae	*Levilactobacillus*	*Levilactobacillus paucivorans*
Bacillota	Lactobacillaceae	*Levilactobacillus*	*Levilactobacillus senmaizukei*
Bacillota	Lactobacillaceae	*Levilactobacillus*	*Levilactobacillus spicheri*
Bacillota	Lactobacillaceae	*Levilactobacillus*	*Levilactobacillus zymae*
Bacillota	Lactobacillaceae	*Ligilactobacillus*	*Ligilactobacillus acidipiscis*
Bacillota	Lactobacillaceae	*Ligilactobacillus*	*Ligilactobacillus agilis*
Bacillota	Lactobacillaceae	*Ligilactobacillus*	*Ligilactobacillus animalis*
Bacillota	Lactobacillaceae	*Ligilactobacillus*	*Ligilactobacillus apodemi*
Bacillota	Lactobacillaceae	*Ligilactobacillus*	*Ligilactobacillus araffinosus*
Bacillota	Lactobacillaceae	*Ligilactobacillus*	*Ligilactobacillus aviarius*
Bacillota	Lactobacillaceae	*Ligilactobacillus*	*Ligilactobacillus ceti*
Bacillota	Lactobacillaceae	*Ligilactobacillus*	*Ligilactobacillus equi*
Bacillota	Lactobacillaceae	*Ligilactobacillus*	*Ligilactobacillus faecis*
Bacillota	Lactobacillaceae	*Ligilactobacillus*	*Ligilactobacillus hayakitensis*
Bacillota	Lactobacillaceae	*Ligilactobacillus*	*Ligilactobacillus murinus*
Bacillota	Lactobacillaceae	*Ligilactobacillus*	*Ligilactobacillus pobuzihii*
Bacillota	Lactobacillaceae	*Ligilactobacillus*	*Ligilactobacillus ruminis*
Bacillota	Lactobacillaceae	*Ligilactobacillus*	*Ligilactobacillus saerimneri*
Bacillota	Lactobacillaceae	*Ligilactobacillus*	*Ligilactobacillus salivarius*
Bacillota	Lactobacillaceae	*Limosilactobacillus*	*Limosilactobacillus alvi*
Bacillota	Lactobacillaceae	*Limosilactobacillus*	*Limosilactobacillus caviae*
Bacillota	Lactobacillaceae	*Limosilactobacillus*	*Limosilactobacillus coleohominis*
Bacillota	Lactobacillaceae	*Limosilactobacillus*	*Limosilactobacillus equigenerosi*
Bacillota	Lactobacillaceae	*Limosilactobacillus*	*Limosilactobacillus fermentum*

Bacillota	Lactobacillaceae	*Limosilactobacillus*	*Limosilactobacillus frumenti*
Bacillota	Lactobacillaceae	*Limosilactobacillus*	*Limosilactobacillus gastricus*
Bacillota	Lactobacillaceae	*Limosilactobacillus*	*Limosilactobacillus mucosae*
Bacillota	Lactobacillaceae	*Limosilactobacillus*	*Limosilactobacillus pontis*
Bacillota	Lactobacillaceae	*Limosilactobacillus*	*Limosilactobacillus reuteri*
Bacillota	Lactobacillaceae	*Limosilactobacillus*	*Limosilactobacillus secaliphilus*
Bacillota	Lactobacillaceae	*Liquorilactobacillus*	*Liquorilactobacillus aquaticus*
Bacillota	Lactobacillaceae	*Liquorilactobacillus*	*Liquorilactobacillus cacaonum*
Bacillota	Lactobacillaceae	*Liquorilactobacillus*	*Liquorilactobacillus capillatus*
Bacillota	Lactobacillaceae	*Liquorilactobacillus*	*Liquorilactobacillus ghanensis*
Bacillota	Lactobacillaceae	*Liquorilactobacillus*	*Liquorilactobacillus hordei*
Bacillota	Lactobacillaceae	*Liquorilactobacillus*	*Liquorilactobacillus mali*
Bacillota	Lactobacillaceae	*Liquorilactobacillus*	*Liquorilactobacillus nagelii*
Bacillota	Lactobacillaceae	*Liquorilactobacillus*	*Liquorilactobacillus oeni*
Bacillota	Lactobacillaceae	*Liquorilactobacillus*	*Liquorilactobacillus satsumensis*
Bacillota	Lactobacillaceae	*Liquorilactobacillus*	*Liquorilactobacillus sucicola*
Bacillota	Lactobacillaceae	*Liquorilactobacillus*	*Liquorilactobacillus uvarum*
Bacillota	Lactobacillaceae	*Liquorilactobacillus*	*Liquorilactobacillus vini*
Bacillota	Listeriaceae	*Listeria*	*Listeria aquatica*
Bacillota	Listeriaceae	*Listeria*	*Listeria fleischmannii*
Bacillota	Listeriaceae	*Listeria*	*Listeria floridensis*
Bacillota	Listeriaceae	*Listeria*	*Listeria grayi*
Bacillota	Listeriaceae	*Listeria*	*Listeria innocua*
Bacillota	Listeriaceae	*Listeria*	*Listeria ivanovii*
Bacillota	Listeriaceae	*Listeria*	*Listeria monocytogenes*
Bacillota	Listeriaceae	*Listeria*	*Listeria riparia*
Bacillota	Listeriaceae	*Listeria*	*Listeria rocourtiae*
Bacillota	Listeriaceae	*Listeria*	*Listeria seeligeri*

Bacillota	Listeriaceae	*Listeria*	*Listeria weihenstephanensis*
Bacillota	Listeriaceae	*Listeria*	*Listeria welshimeri*
Bacillota	Bacillaceae	*Litchfieldia*	*Litchfieldia salsus*
Bacillota	Bacillaceae	*Litoribacterium*	*Litoribacterium kuwaitense*
Bacillota	Lactobacillaceae	*Loigolactobacillus*	*Loigolactobacillus backii*
Bacillota	Lactobacillaceae	*Loigolactobacillus*	*Loigolactobacillus bifermentans*
Bacillota	Lactobacillaceae	*Loigolactobacillus*	*Loigolactobacillus coryniformis*
Bacillota	Lactobacillaceae	*Loigolactobacillus*	*Loigolactobacillus iwatensis*
Bacillota	Lactobacillaceae	*Loigolactobacillus*	*Loigolactobacillus rennini*
Bacillota	Bacillaceae	*Lottiidibacillus*	*Lottiidibacillus patelloidae*
Bacillota	Lutisporaceae	*Lutispora*	*Lutispora thermophila*
Bacillota	Planococcaceae	*Lysinibacillus*	*Lysinibacillus alkaliphilus*
Bacillota	Planococcaceae	*Lysinibacillus*	*Lysinibacillus boronitolerans*
Bacillota	Planococcaceae	*Lysinibacillus*	*Lysinibacillus endophyticus*
Bacillota	Planococcaceae	*Lysinibacillus*	*Lysinibacillus fusiformis*
Bacillota	Planococcaceae	*Lysinibacillus*	*Lysinibacillus halotolerans*
Bacillota	Planococcaceae	*Lysinibacillus*	*Lysinibacillus macroides*
Bacillota	Planococcaceae	*Lysinibacillus*	*Lysinibacillus odysseyi*
Bacillota	Planococcaceae	*Lysinibacillus*	*Lysinibacillus pakistanensis*
Bacillota	Planococcaceae	*Lysinibacillus*	*Lysinibacillus parviboronicapiens*
Bacillota	Planococcaceae	*Lysinibacillus*	*Lysinibacillus sphaericus*
Bacillota	Planococcaceae	*Lysinibacillus*	*Lysinibacillus tabacifolii*
Bacillota	Staphylococcaceae	*Macrococcus*	*Macrococcus bohemicus*
Bacillota	Staphylococcaceae	*Macrococcus*	*Macrococcus bovicus*
Bacillota	Staphylococcaceae	*Macrococcus*	*Macrococcus brunensis*
Bacillota	Staphylococcaceae	*Macrococcus*	*Macrococcus canis*
Bacillota	Staphylococcaceae	*Macrococcus*	*Macrococcus carouselicus*
Bacillota	Staphylococcaceae	*Macrococcus*	*Macrococcus caseolyticus*

Bacillota	Staphylococcaceae	*Macrococcus*	*Macrococcus equipercicus*
Bacillota	Staphylococcaceae	*Macrococcus*	*Macrococcus goetzii*
Bacillota	Staphylococcaceae	*Macrococcus*	*Macrococcus goetzii;Macrococcus*
Bacillota	Staphylococcaceae	*Macrococcus*	*Macrococcus hajekii*
Bacillota	Staphylococcaceae	*Macrococcus*	*Macrococcus lamae*
Bacillota	Mahellaceae	*Mahella*	*Mahella australiensis*
Bacillota	Staphylococcaceae	*Mammaliicoccus*	*Mammaliicoccus lentus*
Bacillota	Staphylococcaceae	*Mammaliicoccus*	*Mammaliicoccus sciuri*
Bacillota	Staphylococcaceae	*Mammaliicoccus*	*Mammaliicoccus stepanovicii*
Bacillota	Staphylococcaceae	*Mammaliicoccus*	*Mammaliicoccus vitulinus*
Bacillota	Bacillaceae	*Margalitia*	*Margalitia camelliae*
Bacillota	Bacillaceae	*Margalitia*	*Margalitia shackletonii*
Bacillota	Carnobacteriaceae	*Marinilactibacillus*	*Marinilactibacillus piezotolerans*
Bacillota	Carnobacteriaceae	*Marinilactibacillus*	*Marinilactibacillus psychrotolerans*
Bacillota	Thermotaleaceae	*Marinisporobacter*	*Marinisporobacter balticus*
Bacillota	Massilibacteriaceae	*Massilibacterium*	*Massilibacterium senegalense*
Bacillota	Lachnospiraceae	*Mediterraneibacter*	*Mediterraneibacter glycyrrhizinilyticus*
Bacillota	Megasphaeraceae	*Megasphaera*	*Megasphaera indica*
Bacillota	Megasphaeraceae	*Megasphaera*	*Megasphaera massiliensis*
Bacillota	Enterococcaceae	*Melissococcus*	*Melissococcus plutonius*
Bacillota	Bacillaceae	*Mesobacillus*	*Mesobacillus foraminis*
Bacillota	Bacillaceae	*Mesobacillus*	*Mesobacillus jeotgali*
Bacillota	Bacillaceae	*Mesobacillus*	*Mesobacillus maritimus*
Bacillota	Bacillaceae	*Mesobacillus*	*Mesobacillus persicus*
Bacillota	Bacillaceae	*Mesobacillus*	*Mesobacillus rigiliprofundi*
Bacillota	Bacillaceae	*Mesobacillus*	*Mesobacillus stamsii*
Bacillota	Bacillaceae	*Mesobacillus*	*Mesobacillus subterraneus*
Bacillota	Bacillaceae	*Metabacillus*	*Metabacillus fastidiosus*

Bacillota	Bacillaceae	*Metabacillus*	*Metabacillus galliciensis*
Bacillota	Bacillaceae	*Metabacillus*	*Metabacillus halosaccharovorans*
Bacillota	Bacillaceae	*Metabacillus*	*Metabacillus herbersteinensis*
Bacillota	Bacillaceae	*Metabacillus*	*Metabacillus indicus*
Bacillota	Bacillaceae	*Metabacillus*	*Metabacillus litoralis*
Bacillota	Bacillaceae	*Metabacillus*	*Metabacillus malikii*
Bacillota	Bacillaceae	*Metabacillus*	*Metabacillus niabensis*
Bacillota	Planococcaceae	*Metasolibacillus*	*Metasolibacillus fluoroglycofenilyticus*
Bacillota	Lachnospiraceae	*Mobilisporobacter*	*Mobilisporobacter senegalensis*
Bacillota	Lachnospiraceae	*Mobilitalea*	*Mobilitalea sibirica*
Bacillota	Anaerovoracaceae	*Mogibacterium*	*Mogibacterium neglectum*
Bacillota	Anaerovoracaceae	*Mogibacterium*	*Mogibacterium timidum*
Bacillota	Monoglobaceae	*Monoglobus*	*Monoglobus pectinilyticus*
Bacillota	Moorellaceae	*Moorella*	*Moorella humiferrea*
Bacillota	Moorellaceae	*Moorella*	*Moorella perchloratireducens*
Bacillota	Moorellaceae	*Moorella*	*Moorella thermoacetica*
Bacillota	Clostridiaceae	*Mordavella*	*Mordavella massiliensis*
Bacillota	Lachnospiraceae	*Murimonas*	*Murimonas intestini*
Bacillota	Mycoplasmataceae	*Mycoplasma*	*Mycoplasma alvi*
Bacillota	Mycoplasmataceae	*Mycoplasma*	*Mycoplasma capricolum*
Bacillota	Mycoplasmataceae	*Mycoplasma*	*Mycoplasma falconis*
Bacillota	Mycoplasmataceae	*Mycoplasma*	*Mycoplasma fastidiosum*
Bacillota	Mycoplasmataceae	*Mycoplasma*	*Mycoplasma faucium*
Bacillota	Mycoplasmataceae	*Mycoplasma*	*Mycoplasma gateae*
Bacillota	Mycoplasmataceae	*Mycoplasma*	*Mycoplasma genitalium*
Bacillota	Mycoplasmataceae	*Mycoplasma*	*Mycoplasma gypis*
Bacillota	Mycoplasmataceae	*Mycoplasma*	*Mycoplasma hominis*
Bacillota	Mycoplasmataceae	*Mycoplasma*	*Mycoplasma hyorhinis*

Bacillota	Mycoplasmataceae	*Mycoplasma*	*Mycoplasma iowae*
Bacillota	Mycoplasmataceae	*Mycoplasma*	*Mycoplasma lagogenitalium*
Bacillota	Mycoplasmataceae	*Mycoplasma*	*Mycoplasma microti*
Bacillota	Mycoplasmataceae	*Mycoplasma*	*Mycoplasma moatsii*
Bacillota	Mycoplasmataceae	*Mycoplasma*	*Mycoplasma mobile*
Bacillota	Mycoplasmataceae	*Mycoplasma*	*Mycoplasma molare*
Bacillota	Mycoplasmataceae	*Mycoplasma*	*Mycoplasma muris*
Bacillota	Mycoplasmataceae	*Mycoplasma*	*Mycoplasma mycoides*
Bacillota	Mycoplasmataceae	*Mycoplasma*	*Mycoplasma neophronis*
Bacillota	Mycoplasmataceae	*Mycoplasma*	*Mycoplasma orale*
Bacillota	Mycoplasmataceae	*Mycoplasma*	*Mycoplasma penetrans*
Bacillota	Mycoplasmataceae	*Mycoplasma*	*Mycoplasma pirum*
Bacillota	Mycoplasmataceae	*Mycoplasma*	*Mycoplasma sualvi*
Bacillota	Mycoplasmataceae	*Mycoplasma*	*Mycoplasma subdolum*
Bacillota	Mycoplasmataceae	*Mycoplasma*	*Mycoplasma testudinis*
Bacillota	Mycoplasmataceae	*Mycoplasma*	*Mycoplasma tullyi*
Bacillota	Metamycoplasmataceae	*Mycoplasmopsis*	*Mycoplasmopsis bovis*
Bacillota	Metamycoplasmataceae	*Mycoplasmopsis*	*Mycoplasmopsis edwardii*
Bacillota	Metamycoplasmataceae	*Mycoplasmopsis*	*Mycoplasmopsis gallinarum*
Bacillota	Metamycoplasmataceae	*Mycoplasmopsis*	*Mycoplasmopsis lipofaciens*
Bacillota	Metamycoplasmataceae	*Mycoplasmopsis*	*Mycoplasmopsis lipophila*
Bacillota	Metamycoplasmataceae	*Mycoplasmopsis*	*Mycoplasmopsis meleagridis*
Bacillota	Metamycoplasmataceae	*Mycoplasmopsis*	*Mycoplasmopsis pullorum*
Bacillota	Natranaerobiaceae	*Natranaerobaculum*	*Natranaerobaculum magadiense*
Bacillota	DSM-24629	*Natranaerovirga*	*Natranaerovirga hydrolytica*
Bacillota	DSM-24629	*Natranaerovirga*	*Natranaerovirga pectinivora*
Bacillota	Natronincolaceae	*Natronincola*	*Natronincola ferrireducens*
Bacillota	Natronincolaceae	*Natronincola*	*Natronincola peptidivorans*

Bacillota	Ruminococcaceae	*Negativibacillus*	*Negativibacillus massiliensis*
Bacillota	Oscillospiraceae	*Neglecta*	*Neglecta timonensis*
Bacillota	Bacillaceae	*Neobacillus*	*Neobacillus bataviensis*
Bacillota	Bacillaceae	*Neobacillus*	*Neobacillus circulans*
Bacillota	Bacillaceae	*Neobacillus*	*Neobacillus cucumis*
Bacillota	Bacillaceae	*Neobacillus*	*Neobacillus drentensis*
Bacillota	Bacillaceae	*Neobacillus*	*Neobacillus fumarioli*
Bacillota	Bacillaceae	*Neobacillus*	*Neobacillus jeddahensis*
Bacillota	Bacillaceae	*Neobacillus*	*Neobacillus mesonae*
Bacillota	Bacillaceae	*Neobacillus*	*Neobacillus niacini*
Bacillota	Bacillaceae	*Neobacillus*	*Neobacillus novalis*
Bacillota	Bacillaceae	*Neobacillus*	*Neobacillus thermocopriae*
Bacillota	Bacillaceae	*Niallia*	*Niallia circulans*
Bacillota	Bacillaceae	*Niallia*	*Niallia endozanthoxylicus*
Bacillota	Bacillaceae	*Niallia*	*Niallia nealsonii*
Bacillota	Incertae	*Nordella*	*Nordella oligomobilis*
Bacillota	Salinicoccaceae	*Nosocomiicoccus*	*Nosocomiicoccus ampullae*
Bacillota	Salinicoccaceae	*Nosocomiicoccus*	*Nosocomiicoccus massiliensis*
Bacillota	Clostridiaceae	*Oceanirhabdus*	*Oceanirhabdus sediminicola*
Bacillota	Amphibacillaceae	*Oceanobacillus*	*Oceanobacillus arenosus*
Bacillota	Amphibacillaceae	*Oceanobacillus*	*Oceanobacillus bengalensis*
Bacillota	Amphibacillaceae	*Oceanobacillus*	*Oceanobacillus caeni*
Bacillota	Amphibacillaceae	*Oceanobacillus*	*Oceanobacillus halophilum*
Bacillota	Amphibacillaceae	*Oceanobacillus*	*Oceanobacillus iheyensis*
Bacillota	Amphibacillaceae	*Oceanobacillus*	*Oceanobacillus indicireducens*
Bacillota	Amphibacillaceae	*Oceanobacillus*	*Oceanobacillus jeddahense*
Bacillota	Amphibacillaceae	*Oceanobacillus*	*Oceanobacillus kimchii*
Bacillota	Amphibacillaceae	*Oceanobacillus*	*Oceanobacillus limi*

Bacillota	Amphibacillaceae	*Oceanobacillus*	*Oceanobacillus massiliensis*
Bacillota	Amphibacillaceae	*Oceanobacillus*	*Oceanobacillus oncorhynchi*
Bacillota	Amphibacillaceae	*Oceanobacillus*	*Oceanobacillus picturae*
Bacillota	Amphibacillaceae	*Oceanobacillus*	*Oceanobacillus polygoni*
Bacillota	Amphibacillaceae	*Oceanobacillus*	*Oceanobacillus rekensis*
Bacillota	Amphibacillaceae	*Oceanobacillus*	*Oceanobacillus senegalensis*
Bacillota	Amphibacillaceae	*Oceanobacillus*	*Oceanobacillus sojae*
Bacillota	Amphibacillaceae	*Oceanobacillus*	*Oceanobacillus timonensis*
Bacillota	Lactobacillaceae	*Oenococcus*	*Oenococcus oeni*
Bacillota	Amphibacillaceae	*Ornithinibacillus*	*Ornithinibacillus bavariensis*
Bacillota	Amphibacillaceae	*Ornithinibacillus*	*Ornithinibacillus californiensis*
Bacillota	Amphibacillaceae	*Ornithinibacillus*	*Ornithinibacillus contaminans*
Bacillota	Amphibacillaceae	*Ornithinibacillus*	*Ornithinibacillus halophilus*
Bacillota	Amphibacillaceae	*Ornithinibacillus*	*Ornithinibacillus heyuanensis*
Bacillota	Amphibacillaceae	*Ornithinibacillus*	*Ornithinibacillus salinisoli*
Bacillota	Amphibacillaceae	*Ornithinibacillus*	*Ornithinibacillus scapharcae*
Bacillota	Oscillospiraceae	*Oscillibacter*	*Oscillibacter ruminantium*
Bacillota	Oscillospiraceae	*Oscillibacter*	*Oscillibacter valericigenes*
Bacillota	Paenibacillaceae	*Oxalophagus*	*Oxalophagus oxalicus*
Bacillota	Oxobacteraceae	*Oxobacter*	*Oxobacter pfennigii*
Bacillota	Paenibacillaceae	*Paenibacillus*	*Paenibacillus aceris*
Bacillota	Paenibacillaceae	*Paenibacillus*	*Paenibacillus aceti*
Bacillota	Paenibacillaceae	*Paenibacillus*	*Paenibacillus agaridevorans*
Bacillota	Paenibacillaceae	*Paenibacillus*	*Paenibacillus albus*
Bacillota	Paenibacillaceae	*Paenibacillus*	*Paenibacillus alginolyticus*
Bacillota	Paenibacillaceae	*Paenibacillus*	*Paenibacillus algorifonticola*
Bacillota	Paenibacillaceae	*Paenibacillus*	*Paenibacillus alvei*
Bacillota	Paenibacillaceae	*Paenibacillus*	*Paenibacillus amylolyticus*

Bacillota	Paenibacillaceae	*Paenibacillus*	*Paenibacillus anaericanus*
Bacillota	Paenibacillaceae	*Paenibacillus*	*Paenibacillus antarcticus*
Bacillota	Paenibacillaceae	*Paenibacillus*	*Paenibacillus antibioticophila*
Bacillota	Paenibacillaceae	*Paenibacillus*	*Paenibacillus aquistagni*
Bacillota	Paenibacillaceae	*Paenibacillus*	*Paenibacillus arcticus*
Bacillota	Paenibacillaceae	*Paenibacillus*	*Paenibacillus assamensis*
Bacillota	Paenibacillaceae	*Paenibacillus*	*Paenibacillus azoreducens*
Bacillota	Paenibacillaceae	*Paenibacillus*	*Paenibacillus azotifigens*
Bacillota	Paenibacillaceae	*Paenibacillus*	*Paenibacillus barcinonensis*
Bacillota	Paenibacillaceae	*Paenibacillus*	*Paenibacillus borealis*
Bacillota	Paenibacillaceae	*Paenibacillus*	*Paenibacillus bovis*
Bacillota	Paenibacillaceae	*Paenibacillus*	*Paenibacillus camelliae*
Bacillota	Paenibacillaceae	*Paenibacillus*	*Paenibacillus campinasensis*
Bacillota	Paenibacillaceae	*Paenibacillus*	*Paenibacillus castaneae*
Bacillota	Paenibacillaceae	*Paenibacillus*	*Paenibacillus catalpae*
Bacillota	Paenibacillaceae	*Paenibacillus*	*Paenibacillus cellulosilyticus*
Bacillota	Paenibacillaceae	*Paenibacillus*	*Paenibacillus chibensis*
Bacillota	Paenibacillaceae	*Paenibacillus*	*Paenibacillus chinjuensis*
Bacillota	Paenibacillaceae	*Paenibacillus*	*Paenibacillus chitinolyticus*
Bacillota	Paenibacillaceae	*Paenibacillus*	*Paenibacillus contaminans*
Bacillota	Paenibacillaceae	*Paenibacillus*	*Paenibacillus cookii*
Bacillota	Paenibacillaceae	*Paenibacillus*	*Paenibacillus daejeonensis*
Bacillota	Paenibacillaceae	*Paenibacillus*	*Paenibacillus dakarensis*
Bacillota	Paenibacillaceae	*Paenibacillus*	*Paenibacillus dendritiformis*
Bacillota	Paenibacillaceae	*Paenibacillus*	*Paenibacillus gansuensis*
Bacillota	Paenibacillaceae	*Paenibacillus*	*Paenibacillus gelatinilyticus*
Bacillota	Paenibacillaceae	*Paenibacillus*	*Paenibacillus ginsengihumi*
Bacillota	Paenibacillaceae	*Paenibacillus*	*Paenibacillus glacialis*

Bacillota	Paenibacillaceae	*Paenibacillus*	*Paenibacillus glucanolyticus*
Bacillota	Paenibacillaceae	*Paenibacillus*	*Paenibacillus glycanilyticus*
Bacillota	Paenibacillaceae	*Paenibacillus*	*Paenibacillus gorillae*
Bacillota	Paenibacillaceae	*Paenibacillus*	*Paenibacillus granivorans*
Bacillota	Paenibacillaceae	*Paenibacillus*	*Paenibacillus guangzhouensis*
Bacillota	Paenibacillaceae	*Paenibacillus*	*Paenibacillus harenae*
Bacillota	Paenibacillaceae	*Paenibacillus*	*Paenibacillus helianthi*
Bacillota	Paenibacillaceae	*Paenibacillus*	*Paenibacillus herberti*
Bacillota	Paenibacillaceae	*Paenibacillus*	*Paenibacillus hodogayensis*
Bacillota	Paenibacillaceae	*Paenibacillus*	*Paenibacillus hordei*
Bacillota	Paenibacillaceae	*Paenibacillus*	*Paenibacillus humicus*
Bacillota	Paenibacillaceae	*Paenibacillus*	*Paenibacillus ihumii*
Bacillota	Paenibacillaceae	*Paenibacillus*	*Paenibacillus illinoisensis*
Bacillota	Paenibacillaceae	*Paenibacillus*	*Paenibacillus jamilae*
Bacillota	Paenibacillaceae	*Paenibacillus*	*Paenibacillus kobensis*
Bacillota	Paenibacillaceae	*Paenibacillus*	*Paenibacillus koleovorans*
Bacillota	Paenibacillaceae	*Paenibacillus*	*Paenibacillus lactis*
Bacillota	Paenibacillaceae	*Paenibacillus*	*Paenibacillus lacus*
Bacillota	Paenibacillaceae	*Paenibacillus*	*Paenibacillus larvae*
Bacillota	Paenibacillaceae	*Paenibacillus*	*Paenibacillus lautus*
Bacillota	Paenibacillaceae	*Paenibacillus*	*Paenibacillus liaoningensis*
Bacillota	Paenibacillaceae	*Paenibacillus*	*Paenibacillus lupini*
Bacillota	Paenibacillaceae	*Paenibacillus*	*Paenibacillus lutimineralis*
Bacillota	Paenibacillaceae	*Paenibacillus*	*Paenibacillus lutrae*
Bacillota	Paenibacillaceae	*Paenibacillus*	*Paenibacillus macerans*
Bacillota	Paenibacillaceae	*Paenibacillus*	*Paenibacillus marchantiophytorum*
Bacillota	Paenibacillaceae	*Paenibacillus*	*Paenibacillus marinisediminis*
Bacillota	Paenibacillaceae	*Paenibacillus*	*Paenibacillus massiliensis*

Bacillota	Paenibacillaceae	*Paenibacillus*	*Paenibacillus medicaginis*
Bacillota	Paenibacillaceae	*Paenibacillus*	*Paenibacillus mucilaginosus*
Bacillota	Paenibacillaceae	*Paenibacillus*	*Paenibacillus nasutitermitis*
Bacillota	Paenibacillaceae	*Paenibacillus*	*Paenibacillus nebraskensis*
Bacillota	Paenibacillaceae	*Paenibacillus*	*Paenibacillus odorifer*
Bacillota	Paenibacillaceae	*Paenibacillus*	*Paenibacillus oralis*
Bacillota	Paenibacillaceae	*Paenibacillus*	*Paenibacillus oryzae*
Bacillota	Paenibacillaceae	*Paenibacillus*	*Paenibacillus pabuli*
Bacillota	Paenibacillaceae	*Paenibacillus*	*Paenibacillus panaciterrae*
Bacillota	Paenibacillaceae	*Paenibacillus*	*Paenibacillus peoriae*
Bacillota	Paenibacillaceae	*Paenibacillus*	*Paenibacillus pinihumi*
Bacillota	Paenibacillaceae	*Paenibacillus*	*Paenibacillus pinisoli*
Bacillota	Paenibacillaceae	*Paenibacillus*	*Paenibacillus polymyxa*
Bacillota	Paenibacillaceae	*Paenibacillus*	*Paenibacillus profundus*
Bacillota	Paenibacillaceae	*Paenibacillus*	*Paenibacillus prosopidis*
Bacillota	Paenibacillaceae	*Paenibacillus*	*Paenibacillus provencensis*
Bacillota	Paenibacillaceae	*Paenibacillus*	*Paenibacillus psychroresistens*
Bacillota	Paenibacillaceae	*Paenibacillus*	*Paenibacillus qingshengii*
Bacillota	Paenibacillaceae	*Paenibacillus*	*Paenibacillus quercus*
Bacillota	Paenibacillaceae	*Paenibacillus*	*Paenibacillus relictisesami*
Bacillota	Paenibacillaceae	*Paenibacillus*	*Paenibacillus rhizoryzae*
Bacillota	Paenibacillaceae	*Paenibacillus*	*Paenibacillus rigui*
Bacillota	Paenibacillaceae	*Paenibacillus*	*Paenibacillus sabinae*
Bacillota	Paenibacillaceae	*Paenibacillus*	*Paenibacillus sanguinis*
Bacillota	Paenibacillaceae	*Paenibacillus*	*Paenibacillus sediminis*
Bacillota	Paenibacillaceae	*Paenibacillus*	*Paenibacillus selenii*
Bacillota	Paenibacillaceae	*Paenibacillus*	*Paenibacillus selenitireducens*
Bacillota	Paenibacillaceae	*Paenibacillus*	*Paenibacillus senegalensis*

Bacillota	Paenibacillaceae	*Paenibacillus*	*Paenibacillus shunpengii*
Bacillota	Paenibacillaceae	*Paenibacillus*	*Paenibacillus silagei*
Bacillota	Paenibacillaceae	*Paenibacillus*	*Paenibacillus silvae*
Bacillota	Paenibacillaceae	*Paenibacillus*	*Paenibacillus sinopodophylli*
Bacillota	Paenibacillaceae	*Paenibacillus*	*Paenibacillus solani*
Bacillota	Paenibacillaceae	*Paenibacillus*	*Paenibacillus soli*
Bacillota	Paenibacillaceae	*Paenibacillus*	*Paenibacillus stellifer*
Bacillota	Paenibacillaceae	*Paenibacillus*	*Paenibacillus taichungensis*
Bacillota	Paenibacillaceae	*Paenibacillus*	*Paenibacillus taiwanensis*
Bacillota	Paenibacillaceae	*Paenibacillus*	*Paenibacillus telluris*
Bacillota	Paenibacillaceae	*Paenibacillus*	*Paenibacillus thailandensis*
Bacillota	Paenibacillaceae	*Paenibacillus*	*Paenibacillus thermoaerophilus*
Bacillota	Paenibacillaceae	*Paenibacillus*	*Paenibacillus thermophilus*
Bacillota	Paenibacillaceae	*Paenibacillus*	*Paenibacillus tianmuensis*
Bacillota	Paenibacillaceae	*Paenibacillus*	*Paenibacillus timonensis*
Bacillota	Paenibacillaceae	*Paenibacillus*	*Paenibacillus tritici*
Bacillota	Paenibacillaceae	*Paenibacillus*	*Paenibacillus tundrae*
Bacillota	Paenibacillaceae	*Paenibacillus*	*Paenibacillus turicensis*
Bacillota	Paenibacillaceae	*Paenibacillus*	*Paenibacillus typhae*
Bacillota	Paenibacillaceae	*Paenibacillus*	*Paenibacillus uliginis*
Bacillota	Paenibacillaceae	*Paenibacillus*	*Paenibacillus urinalis*
Bacillota	Paenibacillaceae	*Paenibacillus*	*Paenibacillus validus*
Bacillota	Paenibacillaceae	*Paenibacillus*	*Paenibacillus wooponensis*
Bacillota	Paenibacillaceae	*Paenibacillus*	*Paenibacillus wulumuqiensis*
Bacillota	Paenibacillaceae	*Paenibacillus*	*Paenibacillus xinjiangensis*
Bacillota	Paenibacillaceae	*Paenibacillus*	*Paenibacillus xylanilyticus*
Bacillota	Paenibacillaceae	*Paenibacillus*	*Paenibacillus yonginensis*
Bacillota	Paenibacillaceae	*Paenibacillus*	*Paenibacillus yunnanensis*

Bacillota	Paenibacillaceae	*Paenibacillus*	*Paenibacillus zeae*
Bacillota	Peptostreptococcaceae	*Paeniclostridium*	*Paeniclostridium ghonii*
Bacillota	Peptostreptococcaceae	*Paeniclostridium*	*Paeniclostridium sordellii*
Bacillota	Planococcaceae	*Paenisporosarcina*	*Paenisporosarcina indica*
Bacillota	Planococcaceae	*Paenisporosarcina*	*Paenisporosarcina macmurdoensis*
Bacillota	Planococcaceae	*Paenisporosarcina*	*Paenisporosarcina quisquiliarum*
Bacillota	Oscillospiraceae	*Paludicola*	*Paludicola psychrotolerans*
Bacillota	Thermoactinomycetaceae	*Paludifilum*	*Paludifilum halophilum*
Bacillota	Oscillospiraceae	*Papillibacter*	*Papillibacter cinnamivorans*
Bacillota	Peptostreptococcaceae	*Paraclostridium*	*Paraclostridium benzoelyticum*
Bacillota	Peptostreptococcaceae	*Paraclostridium*	*Paraclostridium bifermentans*
Bacillota	Anoxybacillaceae	*Parageobacillus*	*Parageobacillus thermantarcticus*
Bacillota	Lachnospiraceae	*Parasporobacterium*	*Parasporobacterium paucivorans*
Bacillota	Helcococcaceae	*Parvimonas*	*Parvimonas micra*
Bacillota	Lactobacillaceae	*Paucilactobacillus*	*Paucilactobacillus hokkaidonensis*
Bacillota	Lactobacillaceae	*Paucilactobacillus*	*Paucilactobacillus nenjiangensis*
Bacillota	Lactobacillaceae	*Paucilactobacillus*	*Paucilactobacillus oligofermentans*
Bacillota	Lactobacillaceae	*Paucilactobacillus*	*Paucilactobacillus suebicus*
Bacillota	Lactobacillaceae	*Paucilactobacillus*	*Paucilactobacillus vaccinostercus*
Bacillota	Lactobacillaceae	*Paucilactobacillus*	*Paucilactobacillus wasatchensis*
Bacillota	Amphibacillaceae	*Paucisalibacillus*	*Paucisalibacillus globulus*
Bacillota	Selenomonadaceae	*Pectinatus*	*Pectinatus haikarae*
Bacillota	Selenomonadaceae	*Pectinatus*	*Pectinatus sottacetonis*
Bacillota	Lactobacillaceae	*Pediococcus*	*Pediococcus acidilactici*
Bacillota	Lactobacillaceae	*Pediococcus*	*Pediococcus argentinicus*
Bacillota	Lactobacillaceae	*Pediococcus*	*Pediococcus cellicola*
Bacillota	Lactobacillaceae	*Pediococcus*	*Pediococcus claussenii*
Bacillota	Lactobacillaceae	*Pediococcus*	*Pediococcus damnosus*

Bacillota	Lactobacillaceae	*Pediococcus*	*Pediococcus ethanolidurans*
Bacillota	Lactobacillaceae	*Pediococcus*	*Pediococcus inopinatus*
Bacillota	Lactobacillaceae	*Pediococcus*	*Pediococcus parvulus*
Bacillota	Lactobacillaceae	*Pediococcus*	*Pediococcus pentosaceus*
Bacillota	Lactobacillaceae	*Pediococcus*	*Pediococcus siamensis*
Bacillota	Lactobacillaceae	*Pediococcus*	*Pediococcus stilesii*
Bacillota	Sporomusaceae	*Pelorhabdus*	*Pelorhabdus rhamnosifermentans*
Bacillota	Pelosinaceae	*Pelosinus*	*Pelosinus defluvii*
Bacillota	Pelosinaceae	*Pelosinus*	*Pelosinus propionicus*
Bacillota	Pelotomaculaceae	*Pelotomaculum*	*Pelotomaculum isophthalicicum*
Bacillota	Pelotomaculaceae	*Pelotomaculum*	*Pelotomaculum propionicicum*
Bacillota	Pelotomaculaceae	*Pelotomaculum*	*Pelotomaculum thermopropionicum*
Bacillota	Peptostreptococcaceae	*Peptacetobacter*	*Peptacetobacter hiranonis*
Bacillota	Peptoclostridiaceae	*Peptoclostridium*	*Peptoclostridium acidaminophilum*
Bacillota	Peptoclostridiaceae	*Peptoclostridium*	*Peptoclostridium litorale*
Bacillota	Peptoniphilaceae	*Peptoniphilus*	*Peptoniphilus asaccharolyticus*
Bacillota	Peptoniphilaceae	*Peptoniphilus*	*Peptoniphilus grossensis*
Bacillota	Peptoniphilaceae	*Peptoniphilus*	*Peptoniphilus koenoeneniae*
Bacillota	Peptoniphilaceae	*Peptoniphilus*	*Peptoniphilus methioninivorax*
Bacillota	Peptoniphilaceae	*Peptoniphilus*	*Peptoniphilus stercorisuis*
Bacillota	Peptostreptococcaceae	*Peptostreptococcus*	*Peptostreptococcus anaerobius*
Bacillota	Peptostreptococcaceae	*Peptostreptococcus*	*Peptostreptococcus canis*
Bacillota	Peptostreptococcaceae	*Peptostreptococcus*	*Peptostreptococcus russellii*
Bacillota	Peptostreptococcaceae	*Peptostreptococcus*	*Peptostreptococcus stomatis*
Bacillota	Bacillaceae	*Peribacillus*	*Peribacillus asahii*
Bacillota	Bacillaceae	*Peribacillus*	*Peribacillus deserti*
Bacillota	Bacillaceae	*Peribacillus*	*Peribacillus huizhouensis*
Bacillota	Bacillaceae	*Peribacillus*	*Peribacillus kribbensis*

Bacillota	Bacillaceae	*Peribacillus*	*Peribacillus loiseleuriae*
Bacillota	Bacillaceae	*Peribacillus*	*Peribacillus muralis*
Bacillota	Bacillaceae	*Peribacillus*	*Peribacillus psychrosaccharolyticus*
Bacillota	Bacillaceae	*Peribacillus*	*Peribacillus simplex*
Bacillota	Vallitaleaceae	*Petrocella*	*Petrocella atlantisensis*
Bacillota	Acidaminococcaceae	*Phascolarctobacterium*	*Phascolarctobacterium faecium*
Bacillota	Acidaminococcaceae	*Phascolarctobacterium*	*Phascolarctobacterium succinatutens*
Bacillota	Ruminococcaceae	*Phocea*	*Phocea massiliensis*
Bacillota	Streptococcaceae	*Pilibacter*	*Pilibacter termitis*
Bacillota	Alkalibacillaceae	*Piscibacillus*	*Piscibacillus salipiscarius*
Bacillota	Carnobacteriaceae	*Pisciglobus*	*Pisciglobus halotolerans*
Bacillota	Thermoactinomycetaceae	*Planifilum*	*Planifilum yunnanense*
Bacillota	Planococcaceae	*Planococcus*	*Planococcus chinensis*
Bacillota	Planococcaceae	*Planococcus*	*Planococcus dechangensis*
Bacillota	Planococcaceae	*Planococcus*	*Planococcus halocryophilus*
Bacillota	Planococcaceae	*Planococcus*	*Planococcus kocurii*
Bacillota	Planococcaceae	*Planococcus*	*Planococcus maitriensis*
Bacillota	Planococcaceae	*Planococcus*	*Planococcus maritimus*
Bacillota	Planococcaceae	*Planococcus*	*Planococcus plakortidis*
Bacillota	Planococcaceae	*Planococcus*	*Planococcus rifietoensis*
Bacillota	Planococcaceae	*Planococcus*	*Planococcus salinus*
Bacillota	Planococcaceae	*Planomicrobium*	*Planomicrobium chinense*
Bacillota	Planococcaceae	*Planomicrobium*	*Planomicrobium mcmeekinii*
Bacillota	Planococcaceae	*Planomicrobium*	*Planomicrobium okeanokoites*
Bacillota	Halobacillaceae	*Pontibacillus*	*Pontibacillus halophilus*
Bacillota	Halobacillaceae	*Pontibacillus*	*Pontibacillus litoralis*
Bacillota	Bacillaceae	*Pradoshia*	*Pradoshia eiseniae*
Bacillota	Bacillaceae	*Priestia*	*Priestia abyssalis*

Bacillota	Bacillaceae	*Priestia*	*Priestia aryabhattai*
Bacillota	Bacillaceae	*Priestia*	*Priestia endophytica*
Bacillota	Bacillaceae	*Priestia*	*Priestia filamentosa*
Bacillota	Bacillaceae	*Priestia*	*Priestia flexa*
Bacillota	Bacillaceae	*Priestia*	*Priestia koreensis*
Bacillota	Bacillaceae	*Priestia*	*Priestia megaterium*
Bacillota	Bacillaceae	*Priestia*	*Priestia paraflexa*
Bacillota	Selenomonadaceae	*Propionispira*	*Propionispira arcuata*
Bacillota	Selenomonadaceae	*Propionispira*	*Propionispira paucivorans*
Bacillota	Selenomonadaceae	*Propionispira*	*Propionispira raffinosivorans*
Bacillota	Propionisporaceae	*Propionispora*	*Propionispora hippei*
Bacillota	Propionisporaceae	*Propionispora*	*Propionispora vibrioides*
Bacillota	Proteiniboraceae	*Proteiniborus*	*Proteiniborus ethanoligenes*
Bacillota	Proteiniboraceae	*Proteiniborus*	*Proteiniborus indolifex*
Bacillota	Clostridiaceae	*Proteiniclasticum*	*Proteiniclasticum ruminis*
Bacillota	Proteinivoraceae	*Proteinivorax*	*Proteinivorax tanatarense*
Bacillota	Acetivibrionaceae	*Pseudobacteroides*	*Pseudobacteroides cellulosolvens*
Bacillota	Lachnospiraceae	*Pseudobutyrivibrio*	*Pseudobutyrivibrio ruminis*
Bacillota	Acetivibrionaceae	*Pseudoclostridium*	*Pseudoclostridium thermosuccinogenes*
Bacillota	Oscillospiraceae	*Pseudoflavonifractor*	*Pseudoflavonifractor capillosus*
Bacillota	Oscillospiraceae	*Pseudoflavonifractor*	*Pseudoflavonifractor phocaeensis*
Bacillota	Incertae	*Pseudoscillatoria*	*Pseudoscillatoria coralii*
Bacillota	Planococcaceae	*Psychrobacillus*	*Psychrobacillus lasiicapitis*
Bacillota	Sporomusaceae	*Psychrosinus*	*Psychrosinus fermentans*
Bacillota	Sporolactobacillaceae	*Pullulanibacillus*	*Pullulanibacillus camelliae*
Bacillota	Sporolactobacillaceae	*Pullulanibacillus*	*Pullulanibacillus naganoensis*
Bacillota	Sporolactobacillaceae	*Pullulanibacillus*	*Pullulanibacillus pueri*
Bacillota	Sporolactobacillaceae	*Pullulanibacillus*	*Pullulanibacillus uraniitolerans*

Bacillota	Domibacillaceae	*Quasibacillus*	*Quasibacillus thermotolerans*
Bacillota	Eubacteriaceae	*Rhabdanaerobium*	*Rhabdanaerobium thermarum*
Bacillota	Bacillaceae	*Robertmurraya*	*Robertmurraya beringensis*
Bacillota	Bacillaceae	*Robertmurraya*	*Robertmurraya crescens*
Bacillota	Bacillaceae	*Robertmurraya*	*Robertmurraya korlensis*
Bacillota	Bacillaceae	*Robertmurraya*	*Robertmurraya kyonggiensis*
Bacillota	Bacillaceae	*Robertmurraya*	*Robertmurraya massiliosenegalensis*
Bacillota	Bacillaceae	*Robertmurraya*	*Robertmurraya siralis*
Bacillota	Lachnospiraceae	*Robinsoniella*	*Robinsoniella peoriensis*
Bacillota	Peptostreptococcaceae	*Romboutsia*	*Romboutsia ilealis*
Bacillota	Peptostreptococcaceae	*Romboutsia*	*Romboutsia lituseburensis*
Bacillota	Peptostreptococcaceae	*Romboutsia*	*Romboutsia sedimentorum*
Bacillota	Peptostreptococcaceae	*Romboutsia*	*Romboutsia timonensis*
Bacillota	Lachnospiraceae	*Roseburia*	*Roseburia faecis*
Bacillota	Lachnospiraceae	*Roseburia*	*Roseburia hominis*
Bacillota	Lachnospiraceae	*Roseburia*	*Roseburia intestinalis*
Bacillota	Lachnospiraceae	*Roseburia*	*Roseburia inulinivorans*
Bacillota	Bacillaceae	*Rossellomorea*	*Rossellomorea aquimaris*
Bacillota	Bacillaceae	*Rossellomorea*	*Rossellomorea marisflavi*
Bacillota	Bacillaceae	*Rossellomorea*	*Rossellomorea oryzaecorticis*
Bacillota	Bacillaceae	*Rossellomorea*	*Rossellomorea vietnamensis*
Bacillota	Acetivibrionaceae	*Ruminiclostridium*	*Ruminiclostridium cellobioparum*
Bacillota	Acetivibrionaceae	*Ruminiclostridium*	*Ruminiclostridium cellulolyticum*
Bacillota	Acetivibrionaceae	*Ruminiclostridium*	*Ruminiclostridium hungatei*
Bacillota	Acetivibrionaceae	*Ruminiclostridium*	*Ruminiclostridium josui*
Bacillota	Acetivibrionaceae	*Ruminiclostridium*	*Ruminiclostridium papyrosolvens*
Bacillota	Acetivibrionaceae	*Ruminiclostridium*	*Ruminiclostridium sufflavum*
Bacillota	Oscillospiraceae	*Ruminococcoides*	*Ruminococcoides bili*

Bacillota	Ruminococcaceae	*Ruminococcus*	*Ruminococcus albus*
Bacillota	Ruminococcaceae	*Ruminococcus*	*Ruminococcus bromii*
Bacillota	Ruminococcaceae	*Ruminococcus*	*Ruminococcus callidus*
Bacillota	Ruminococcaceae	*Ruminococcus*	*Ruminococcus champanellensis*
Bacillota	Ruminococcaceae	*Ruminococcus*	*Ruminococcus faecis*
Bacillota	Ruminococcaceae	*Ruminococcus*	*Ruminococcus flavefaciens*
Bacillota	Ruminococcaceae	*Ruminococcus*	*Ruminococcus gauvreauii*
Bacillota	Ruminococcaceae	*Ruminococcus*	*Ruminococcus gnavus*
Bacillota	Ruminococcaceae	*Ruminococcus*	*Ruminococcus torques*
Bacillota	Planococcaceae	*Rummeliibacillus*	*Rummeliibacillus pycnus*
Bacillota	Planococcaceae	*Rummeliibacillus*	*Rummeliibacillus stabekisii*
Bacillota	Planococcaceae	*Rummeliibacillus*	*Rummeliibacillus suwonensis*
Bacillota	Ruminococcaceae	*Ruthenibacterium*	*Ruthenibacterium lactatiformans*
Bacillota	Bacillaceae	*Saccharococcus*	*Saccharococcus thermophilus*
Bacillota	Saccharofermentanaceae	*Saccharofermentans*	*Saccharofermentans acetigenes*
Bacillota	Clostridiaceae	*Salimesophilobacter*	*Salimesophilobacter vulgaris*
Bacillota	Alkalibacillaceae	*Salinibacillus*	*Salinibacillus aidingensis*
Bacillota	Alkalibacillaceae	*Salinibacillus*	*Salinibacillus kushneri*
Bacillota	Alkalibacillaceae	*Salinibacillus*	*Salinibacillus xinjiangensis*
Bacillota	Salinicoccaceae	*Salinicoccus*	*Salinicoccus albus*
Bacillota	Salinicoccaceae	*Salinicoccus*	*Salinicoccus alkaliphilus*
Bacillota	Salinicoccaceae	*Salinicoccus*	*Salinicoccus halodurans*
Bacillota	Salinicoccaceae	*Salinicoccus*	*Salinicoccus hispanicus*
Bacillota	Salinicoccaceae	*Salinicoccus*	*Salinicoccus kekensis*
Bacillota	Salinicoccaceae	*Salinicoccus*	*Salinicoccus kunmingensis*
Bacillota	Salinicoccaceae	*Salinicoccus*	*Salinicoccus roseus*
Bacillota	Salinicoccaceae	*Salinicoccus*	*Salinicoccus salitudinis*
Bacillota	Salinicoccaceae	*Salinicoccus*	*Salinicoccus salsiraiae*

Bacillota	Salinicoccaceae	*Salinicoccus*	*Salinicoccus sediminis*
Bacillota	Salinicoccaceae	*Salinicoccus*	*Salinicoccus sesuvii*
Bacillota	Salinicoccaceae	*Salinicoccus*	*Salinicoccus siamensis*
Bacillota	Alkalibacillaceae	*Salirhabdus*	*Salirhabdus euzebyi*
Bacillota	Alkalibacillaceae	*Salirhabdus*	*Salirhabdus salicampi*
Bacillota	Clostridiaceae	*Sarcina*	*Sarcina maxima*
Bacillota	Clostridiaceae	*Sarcina*	*Sarcina ventriculi*
Bacillota	Bacillaceae	*Schinkia*	*Schinkia azotoformans*
Bacillota	Bacillaceae	*Schinkia*	*Schinkia oryziterrae*
Bacillota	Lactobacillaceae	*Schleiferilactobacillus*	*Schleiferilactobacillus harbinensis*
Bacillota	Lactobacillaceae	*Schleiferilactobacillus*	*Schleiferilactobacillus kimchicus*
Bacillota	Lactobacillaceae	*Schleiferilactobacillus*	*Schleiferilactobacillus odoratitofui*
Bacillota	Lactobacillaceae	*Schleiferilactobacillus*	*Schleiferilactobacillus oryzae*
Bacillota	Lactobacillaceae	*Schleiferilactobacillus*	*Schleiferilactobacillus perolens*
Bacillota	Lactobacillaceae	*Schleiferilactobacillus*	*Schleiferilactobacillus shenzhenensis*
Bacillota	Lactobacillaceae	*Schleiferilactobacillus*	*Schleiferilactobacillus silagei*
Bacillota	Lactobacillaceae	*Schleiferilactobacillus*	*Schleiferilactobacillus similis*
Bacillota	Sporolactobacillaceae	*Scopulibacillus*	*Scopulibacillus daqui*
Bacillota	Lactobacillaceae	*Secundilactobacillus*	*Secundilactobacillus collinoides*
Bacillota	Lactobacillaceae	*Secundilactobacillus*	*Secundilactobacillus malefermentans*
Bacillota	Lactobacillaceae	*Secundilactobacillus*	*Secundilactobacillus mixtipabuli*
Bacillota	Lactobacillaceae	*Secundilactobacillus*	*Secundilactobacillus paracollinoides*
Bacillota	Lactobacillaceae	*Secundilactobacillus*	*Secundilactobacillus pentosiphilus*
Bacillota	Lactobacillaceae	*Secundilactobacillus*	*Secundilactobacillus silagincola*
Bacillota	Sedimentibacteraceae	*Sedimentibacter*	*Sedimentibacter hydroxybenzoicus*
Bacillota	Selenomonadaceae	*Selenomonas*	*Selenomonas lacticifex*
Bacillota	Clostridiaceae	*Senegalia*	*Senegalia massiliensis*
Bacillota	Natronincolaceae	*Serpentinicella*	*Serpentinicella alkaliphila*

Bacillota	Lachnospiraceae	*Shuttleworthia*	*Shuttleworthia satelles*
Bacillota	Bacillaceae	*Siminovitchia*	*Siminovitchia composti*
Bacillota	Bacillaceae	*Siminovitchia*	*Siminovitchia fortis*
Bacillota	Bacillaceae	*Siminovitchia*	*Siminovitchia terrae*
Bacillota	Incertae	*Sinanaerobacter*	*Sinanaerobacter chloroacetimidivorans*
Bacillota	Tissierellaceae	*Soehngenia*	*Soehngenia saccharolytica*
Bacillota	Planococcaceae	*Solibacillus*	*Solibacillus isronensis*
Bacillota	Planococcaceae	*Solibacillus*	*Solibacillus kalamii*
Bacillota	Oscillospiraceae	*Solibaculum*	*Solibaculum mannosilyticum*
Bacillota	Mycoplasmataceae	*Spiroplasma*	*Spiroplasma atrichopogonis*
Bacillota	Mycoplasmataceae	*Spiroplasma*	*Spiroplasma insolitum*
Bacillota	Mycoplasmataceae	*Spiroplasma*	*Spiroplasma lineolae*
Bacillota	Mycoplasmataceae	*Spiroplasma*	*Spiroplasma melliferum*
Bacillota	Mycoplasmataceae	*Spiroplasma*	*Spiroplasma mirum*
Bacillota	Mycoplasmataceae	*Spiroplasma*	*Spiroplasma montanense*
Bacillota	Mycoplasmataceae	*Spiroplasma*	*Spiroplasma phoeniceum*
Bacillota	Mycoplasmataceae	*Spiroplasma*	*Spiroplasma syrphidicola*
Bacillota	Peptostreptococcaceae	*Sporacetigenium*	*Sporacetigenium mesophilum*
Bacillota	Oscillospiraceae	*Sporobacter*	*Sporobacter termitidis*
Bacillota	Lachnospiraceae	*Sporobacterium*	*Sporobacterium olearium*
Bacillota	Sporolactobacillaceae	*Sporolactobacillus*	*Sporolactobacillus kofuensis*
Bacillota	Sporolactobacillaceae	*Sporolactobacillus*	*Sporolactobacillus laevolacticus*
Bacillota	Sporolactobacillaceae	*Sporolactobacillus*	*Sporolactobacillus nakayamae*
Bacillota	Sporolactobacillaceae	*Sporolactobacillus*	*Sporolactobacillus pectinivorans*
Bacillota	Sporolactobacillaceae	*Sporolactobacillus*	*Sporolactobacillus terrae*
Bacillota	Sporomusaceae	*Sporomusa*	*Sporomusa acidovorans*
Bacillota	Sporomusaceae	*Sporomusa*	*Sporomusa aerivorans*
Bacillota	Sporomusaceae	*Sporomusa*	*Sporomusa malonica*

Bacillota	Sporomusaceae	*Sporomusa*	*Sporomusa ovata*
Bacillota	Sporomusaceae	*Sporomusa*	*Sporomusa paucivorans*
Bacillota	Sporomusaceae	*Sporomusa*	*Sporomusa rhizae*
Bacillota	Sporomusaceae	*Sporomusa*	*Sporomusa silvacetica*
Bacillota	Sporomusaceae	*Sporomusa*	*Sporomusa sphaeroides*
Bacillota	Thermohalobacteraceae	*Sporosalibacterium*	*Sporosalibacterium tautonense*
Bacillota	Planococcaceae	*Sporosarcina*	*Sporosarcina aquimarina*
Bacillota	Planococcaceae	*Sporosarcina*	*Sporosarcina koreensis*
Bacillota	Planococcaceae	*Sporosarcina*	*Sporosarcina pasteurii*
Bacillota	Planococcaceae	*Sporosarcina*	*Sporosarcina psychrophila*
Bacillota	Planococcaceae	*Sporosarcina*	*Sporosarcina saromensis*
Bacillota	Planococcaceae	*Sporosarcina*	*Sporosarcina ureae*
Bacillota	Staphylococcaceae	*Staphylococcus*	*Staphylococcus agnetis*
Bacillota	Staphylococcaceae	*Staphylococcus*	*Staphylococcus arlettae*
Bacillota	Staphylococcaceae	*Staphylococcus*	*Staphylococcus aureus*
Bacillota	Staphylococcaceae	*Staphylococcus*	*Staphylococcus auricularis*
Bacillota	Staphylococcaceae	*Staphylococcus*	*Staphylococcus capitis*
Bacillota	Staphylococcaceae	*Staphylococcus*	*Staphylococcus caprae*
Bacillota	Staphylococcaceae	*Staphylococcus*	*Staphylococcus carnosus*
Bacillota	Staphylococcaceae	*Staphylococcus*	*Staphylococcus chromogenes*
Bacillota	Staphylococcaceae	*Staphylococcus*	*Staphylococcus coagulans*
Bacillota	Staphylococcaceae	*Staphylococcus*	*Staphylococcus cohnii*
Bacillota	Staphylococcaceae	*Staphylococcus*	*Staphylococcus condimenti*
Bacillota	Staphylococcaceae	*Staphylococcus*	*Staphylococcus croceilyticus*
Bacillota	Staphylococcaceae	*Staphylococcus*	*Staphylococcus delphini*
Bacillota	Staphylococcaceae	*Staphylococcus*	*Staphylococcus devriesei*
Bacillota	Staphylococcaceae	*Staphylococcus*	*Staphylococcus edaphicus*
Bacillota	Staphylococcaceae	*Staphylococcus*	*Staphylococcus epidermidis*

Bacillota	Staphylococcaceae	*Staphylococcus*	*Staphylococcus equorum*
Bacillota	Staphylococcaceae	*Staphylococcus*	*Staphylococcus felis*
Bacillota	Staphylococcaceae	*Staphylococcus*	*Staphylococcus gallinarum*
Bacillota	Staphylococcaceae	*Staphylococcus*	*Staphylococcus haemolyticus*
Bacillota	Staphylococcaceae	*Staphylococcus*	*Staphylococcus hominis*
Bacillota	Staphylococcaceae	*Staphylococcus*	*Staphylococcus hyicus*
Bacillota	Staphylococcaceae	*Staphylococcus*	*Staphylococcus kloosii*
Bacillota	Staphylococcaceae	*Staphylococcus*	*Staphylococcus lentus*
Bacillota	Staphylococcaceae	*Staphylococcus*	*Staphylococcus lugdunensis*
Bacillota	Staphylococcaceae	*Staphylococcus*	*Staphylococcus lutrae*
Bacillota	Staphylococcaceae	*Staphylococcus*	*Staphylococcus massiliensis*
Bacillota	Staphylococcaceae	*Staphylococcus*	*Staphylococcus microti*
Bacillota	Staphylococcaceae	*Staphylococcus*	*Staphylococcus muscae*
Bacillota	Staphylococcaceae	*Staphylococcus*	*Staphylococcus nepalensis*
Bacillota	Staphylococcaceae	*Staphylococcus*	*Staphylococcus pasteuri*
Bacillota	Staphylococcaceae	*Staphylococcus*	*Staphylococcus petrasii*
Bacillota	Staphylococcaceae	*Staphylococcus*	*Staphylococcus pettenkoferi*
Bacillota	Staphylococcaceae	*Staphylococcus*	*Staphylococcus piscifermentans*
Bacillota	Staphylococcaceae	*Staphylococcus*	*Staphylococcus pragensis*
Bacillota	Staphylococcaceae	*Staphylococcus*	*Staphylococcus pseudintermedius*
Bacillota	Staphylococcaceae	*Staphylococcus*	*Staphylococcus rostri*
Bacillota	Staphylococcaceae	*Staphylococcus*	*Staphylococcus saccharolyticus*
Bacillota	Staphylococcaceae	*Staphylococcus*	*Staphylococcus saprophyticus*
Bacillota	Staphylococcaceae	*Staphylococcus*	*Staphylococcus schleiferi*
Bacillota	Staphylococcaceae	*Staphylococcus*	*Staphylococcus sciuri*
Bacillota	Staphylococcaceae	*Staphylococcus*	*Staphylococcus simiae*
Bacillota	Staphylococcaceae	*Staphylococcus*	*Staphylococcus simulans*
Bacillota	Staphylococcaceae	*Staphylococcus*	*Staphylococcus stepanovicii*

Bacillota	Staphylococcaceae	*Staphylococcus*	*Staphylococcus succinus*
Bacillota	Staphylococcaceae	*Staphylococcus*	*Staphylococcus ureilyticus*
Bacillota	Staphylococcaceae	*Staphylococcus*	*Staphylococcus warneri*
Bacillota	Staphylococcaceae	*Staphylococcus*	*Staphylococcus xylosus*
Bacillota	Streptococcaceae	*Streptococcus*	*Streptococcus acidominimus*
Bacillota	Streptococcaceae	*Streptococcus*	*Streptococcus agalactiae*
Bacillota	Streptococcaceae	*Streptococcus*	*Streptococcus alactolyticus*
Bacillota	Streptococcaceae	*Streptococcus*	*Streptococcus anginosus*
Bacillota	Streptococcaceae	*Streptococcus*	*Streptococcus australis*
Bacillota	Streptococcaceae	*Streptococcus*	*Streptococcus azizii*
Bacillota	Streptococcaceae	*Streptococcus*	*Streptococcus caballi*
Bacillota	Streptococcaceae	*Streptococcus*	*Streptococcus cameli*
Bacillota	Streptococcaceae	*Streptococcus*	*Streptococcus canis*
Bacillota	Streptococcaceae	*Streptococcus*	*Streptococcus caprae*
Bacillota	Streptococcaceae	*Streptococcus*	*Streptococcus castoreus*
Bacillota	Streptococcaceae	*Streptococcus*	*Streptococcus chosunense*
Bacillota	Streptococcaceae	*Streptococcus*	*Streptococcus constellatus*
Bacillota	Streptococcaceae	*Streptococcus*	*Streptococcus criceti*
Bacillota	Streptococcaceae	*Streptococcus*	*Streptococcus cristatus*
Bacillota	Streptococcaceae	*Streptococcus*	*Streptococcus cuniculi*
Bacillota	Streptococcaceae	*Streptococcus*	*Streptococcus danieliae*
Bacillota	Streptococcaceae	*Streptococcus*	*Streptococcus dentapri*
Bacillota	Streptococcaceae	*Streptococcus*	*Streptococcus dentasini*
Bacillota	Streptococcaceae	*Streptococcus*	*Streptococcus dentiloxodontae*
Bacillota	Streptococcaceae	*Streptococcus*	*Streptococcus dentirousetti*
Bacillota	Streptococcaceae	*Streptococcus*	*Streptococcus devriesei*
Bacillota	Streptococcaceae	*Streptococcus*	*Streptococcus didelphis*
Bacillota	Streptococcaceae	*Streptococcus*	*Streptococcus downei*

Bacillota	Streptococcaceae	*Streptococcus*	*Streptococcus dysgalactiae*
Bacillota	Streptococcaceae	*Streptococcus*	*Streptococcus entericus*
Bacillota	Streptococcaceae	*Streptococcus*	*Streptococcus equi*
Bacillota	Streptococcaceae	*Streptococcus*	*Streptococcus equinus*
Bacillota	Streptococcaceae	*Streptococcus*	*Streptococcus ferus*
Bacillota	Streptococcaceae	*Streptococcus*	*Streptococcus fryi*
Bacillota	Streptococcaceae	*Streptococcus*	*Streptococcus gallinaceus*
Bacillota	Streptococcaceae	*Streptococcus*	*Streptococcus gallolyticus*
Bacillota	Streptococcaceae	*Streptococcus*	*Streptococcus gordonii*
Bacillota	Streptococcaceae	*Streptococcus*	*Streptococcus gwangjuense*
Bacillota	Streptococcaceae	*Streptococcus*	*Streptococcus halichoeri*
Bacillota	Streptococcaceae	*Streptococcus*	*Streptococcus halotolerans*
Bacillota	Streptococcaceae	*Streptococcus*	*Streptococcus henryi*
Bacillota	Streptococcaceae	*Streptococcus*	*Streptococcus himalayensis*
Bacillota	Streptococcaceae	*Streptococcus*	*Streptococcus hongkongensis*
Bacillota	Streptococcaceae	*Streptococcus*	*Streptococcus hyointestinalis*
Bacillota	Streptococcaceae	*Streptococcus*	*Streptococcus hyovaginalis*
Bacillota	Streptococcaceae	*Streptococcus*	*Streptococcus ictaluri*
Bacillota	Streptococcaceae	*Streptococcus*	*Streptococcus infantarius*
Bacillota	Streptococcaceae	*Streptococcus*	*Streptococcus infantis*
Bacillota	Streptococcaceae	*Streptococcus*	*Streptococcus iniae*
Bacillota	Streptococcaceae	*Streptococcus*	*Streptococcus intermedius*
Bacillota	Streptococcaceae	*Streptococcus*	*Streptococcus koreensis*
Bacillota	Streptococcaceae	*Streptococcus*	*Streptococcus lactarius*
Bacillota	Streptococcaceae	*Streptococcus*	*Streptococcus loxodontisalivarius*
Bacillota	Streptococcaceae	*Streptococcus*	*Streptococcus lutetiensis*
Bacillota	Streptococcaceae	*Streptococcus*	*Streptococcus macacae*
Bacillota	Streptococcaceae	*Streptococcus*	*Streptococcus macedonicus*

Bacillota	Streptococcaceae	*Streptococcus*	*Streptococcus marimammalium*
Bacillota	Streptococcaceae	*Streptococcus*	*Streptococcus marmotae*
Bacillota	Streptococcaceae	*Streptococcus*	*Streptococcus massiliensis*
Bacillota	Streptococcaceae	*Streptococcus*	*Streptococcus merionis*
Bacillota	Streptococcaceae	*Streptococcus*	*Streptococcus minor*
Bacillota	Streptococcaceae	*Streptococcus*	*Streptococcus mitis*
Bacillota	Streptococcaceae	*Streptococcus*	*Streptococcus moroccensis*
Bacillota	Streptococcaceae	*Streptococcus*	*Streptococcus mutans*
Bacillota	Streptococcaceae	*Streptococcus*	*Streptococcus oralis*
Bacillota	Streptococcaceae	*Streptococcus*	*Streptococcus oricebi*
Bacillota	Streptococcaceae	*Streptococcus*	*Streptococcus orisasini*
Bacillota	Streptococcaceae	*Streptococcus*	*Streptococcus orisratti*
Bacillota	Streptococcaceae	*Streptococcus*	*Streptococcus orisuis*
Bacillota	Streptococcaceae	*Streptococcus*	*Streptococcus ovis*
Bacillota	Streptococcaceae	*Streptococcus*	*Streptococcus ovuberis*
Bacillota	Streptococcaceae	*Streptococcus*	*Streptococcus panodentis*
Bacillota	Streptococcaceae	*Streptococcus*	*Streptococcus pantholopis*
Bacillota	Streptococcaceae	*Streptococcus*	*Streptococcus parasanguinis*
Bacillota	Streptococcaceae	*Streptococcus*	*Streptococcus parauberis*
Bacillota	Streptococcaceae	*Streptococcus*	*Streptococcus pasteurianus*
Bacillota	Streptococcaceae	*Streptococcus*	*Streptococcus periodonticum*
Bacillota	Streptococcaceae	*Streptococcus*	*Streptococcus pharyngis*
Bacillota	Streptococcaceae	*Streptococcus*	*Streptococcus phocae*
Bacillota	Streptococcaceae	*Streptococcus*	*Streptococcus pluranimalium*
Bacillota	Streptococcaceae	*Streptococcus*	*Streptococcus plurextorum*
Bacillota	Streptococcaceae	*Streptococcus*	*Streptococcus pneumoniae*
Bacillota	Streptococcaceae	*Streptococcus*	*Streptococcus porci*
Bacillota	Streptococcaceae	*Streptococcus*	*Streptococcus porcinus*

Bacillota	Streptococcaceae	*Streptococcus*	*Streptococcus porcorum*
Bacillota	Streptococcaceae	*Streptococcus*	*Streptococcus pseudopneumoniae*
Bacillota	Streptococcaceae	*Streptococcus*	*Streptococcus pseudoporcinus*
Bacillota	Streptococcaceae	*Streptococcus*	*Streptococcus pyogenes*
Bacillota	Streptococcaceae	*Streptococcus*	*Streptococcus rifensis*
Bacillota	Streptococcaceae	*Streptococcus*	*Streptococcus rubneri*
Bacillota	Streptococcaceae	*Streptococcus*	*Streptococcus ruminantium*
Bacillota	Streptococcaceae	*Streptococcus*	*Streptococcus rupicaprae*
Bacillota	Streptococcaceae	*Streptococcus*	*Streptococcus salivarius*
Bacillota	Streptococcaceae	*Streptococcus*	*Streptococcus saliviloxodontae*
Bacillota	Streptococcaceae	*Streptococcus*	*Streptococcus sanguinis*
Bacillota	Streptococcaceae	*Streptococcus*	*Streptococcus sinensis*
Bacillota	Streptococcaceae	*Streptococcus*	*Streptococcus sobrinus*
Bacillota	Streptococcaceae	*Streptococcus*	*Streptococcus suis*
Bacillota	Streptococcaceae	*Streptococcus*	*Streptococcus tangierensis*
Bacillota	Streptococcaceae	*Streptococcus*	*Streptococcus thermophilus*
Bacillota	Streptococcaceae	*Streptococcus*	*Streptococcus thoraltensis*
Bacillota	Streptococcaceae	*Streptococcus*	*Streptococcus troglodytae*
Bacillota	Streptococcaceae	*Streptococcus*	*Streptococcus troglodytidis*
Bacillota	Streptococcaceae	*Streptococcus*	*Streptococcus uberis*
Bacillota	Streptococcaceae	*Streptococcus*	*Streptococcus urinalis*
Bacillota	Streptococcaceae	*Streptococcus*	*Streptococcus ursoris*
Bacillota	Streptococcaceae	*Streptococcus*	*Streptococcus vestibularis*
Bacillota	Aerococcaceae	*Suicoccus*	*Suicoccus acidiformans*
Bacillota	Bacillaceae	*Sutcliffiella*	*Sutcliffiella cohnii*
Bacillota	Bacillaceae	*Sutcliffiella*	*Sutcliffiella horikoshii*
Bacillota	Bacillaceae	*Sutcliffiella*	*Sutcliffiella zhanjiangensis*
Bacillota	Bacillaceae	*Swionibacillus*	*Swionibacillus sediminis*

Bacillota	Thermacetogeniaceae	*Syntrophaceticus*	*Syntrophaceticus schinkii*
Bacillota	Syntrophobotulaceae	*Syntrophobotulus*	*Syntrophobotulus glycolicus*
Bacillota	Lachnospiraceae	*Syntrophococcus*	*Syntrophococcus sucromutans*
Bacillota	Syntrophomonadaceae	*Syntrophomonas*	*Syntrophomonas cellicola*
Bacillota	Syntrophomonadaceae	*Syntrophomonas*	*Syntrophomonas palmitatica*
Bacillota	Syntrophomonadaceae	*Syntrophomonas*	*Syntrophomonas wolfei*
Bacillota	Alkalibacillaceae	*Tenuibacillus*	*Tenuibacillus halotolerans*
Bacillota	Alkalibacillaceae	*Tenuibacillus*	*Tenuibacillus multivorans*
Bacillota	Peptostreptococcaceae	*Tepidibacter*	*Tepidibacter formicigenes*
Bacillota	Peptostreptococcaceae	*Tepidibacter*	*Tepidibacter mesophilus*
Bacillota	Peptostreptococcaceae	*Tepidibacter*	*Tepidibacter thalassicus*
Bacillota	Oscillospiraceae	*Tepidibaculum*	*Tepidibaculum saccharolyticum*
Bacillota	Amphibacillaceae	*Terribacillus*	*Terribacillus aidingensis*
Bacillota	Amphibacillaceae	*Terribacillus*	*Terribacillus halophilus*
Bacillota	Amphibacillaceae	*Terribacillus*	*Terribacillus saccharophilus*
Bacillota	Sporolactobacillaceae	*Terrilactibacillus*	*Terrilactibacillus laevilacticus*
Bacillota	Peptostreptococcaceae	*Terrisporobacter*	*Terrisporobacter glycolicus*
Bacillota	Peptostreptococcaceae	*Terrisporobacter*	*Terrisporobacter mayombei*
Bacillota	Peptostreptococcaceae	*Terrisporobacter*	*Terrisporobacter petrolearius*
Bacillota	Enterococcaceae	*Tetragenococcus*	*Tetragenococcus halophilus*
Bacillota	Enterococcaceae	*Tetragenococcus*	*Tetragenococcus koreensis*
Bacillota	Enterococcaceae	*Tetragenococcus*	*Tetragenococcus muriaticus*
Bacillota	Enterococcaceae	*Tetragenococcus*	*Tetragenococcus osmophilus*
Bacillota	Enterococcaceae	*Tetragenococcus*	*Tetragenococcus solitarius*
Bacillota	Thermacetogeniaceae	*Thermacetogenium*	*Thermacetogenium phaeum*
Bacillota	Thermincolaceae	*Thermincola*	*Thermincola carboxydiphila*
Bacillota	Thermoactinomycetaceae	*Thermoactinomyces*	*Thermoactinomyces daqus*
Bacillota	Thermoactinomycetaceae	*Thermoactinomyces*	*Thermoactinomyces khenchelensis*

Bacillota	Thermoanaerobacteraceae	*Thermoanaerobacter*	*Thermoanaerobacter brockii*
Bacillota	Thermoanaerobacteraceae	*Thermoanaerobacterium*	*Thermoanaerobacterium aciditolerans*
Bacillota	Thermoanaerobacteraceae	*Thermoanaerobacterium*	*Thermoanaerobacterium calidifontis*
Bacillota	Thermoanaerobacteraceae	*Thermoanaerobacterium*	*Thermoanaerobacterium xylanolyticum*
Bacillota	Paenibacillaceae	*Thermobacillus*	*Thermobacillus composti*
Bacillota	Paenibacillaceae	*Thermobacillus*	*Thermobacillus xylanilyticus*
Bacillota	DSM-8532	*Thermoclostridium*	*Thermoclostridium caenicola*
Bacillota	DSM-8532	*Thermoclostridium*	*Thermoclostridium stercorarium*
Bacillota	Thermolithobacteraceae	*Thermolithobacter*	*Thermolithobacter carboxydivorans*
Bacillota	Thermosinaceae	*Thermosinus*	*Thermosinus carboxydivorans*
Bacillota	Thermotaleaceae	*Thermotalea*	*Thermotalea metallivorans*
Bacillota	Tindalliaceae	*Tindallia*	*Tindallia texcoconensis*
Bacillota	Aerococcaceae	*Trichococcus*	*Trichococcus alkaliphilus*
Bacillota	Aerococcaceae	*Trichococcus*	*Trichococcus collinsii*
Bacillota	Aerococcaceae	*Trichococcus*	*Trichococcus flocculiformis*
Bacillota	Aerococcaceae	*Trichococcus*	*Trichococcus ilyis*
Bacillota	Aerococcaceae	*Trichococcus*	*Trichococcus paludicola*
Bacillota	Aerococcaceae	*Trichococcus*	*Trichococcus palustris*
Bacillota	Aerococcaceae	*Trichococcus*	*Trichococcus pasteurii*
Bacillota	Aerococcaceae	*Trichococcus*	*Trichococcus patagoniensis*
Bacillota	Aerococcaceae	*Trichococcus*	*Trichococcus shcherbakoviae*
Bacillota	Tumebacillaceae	*Tumebacillus*	*Tumebacillus algifaecis*
Bacillota	Tumebacillaceae	*Tumebacillus*	*Tumebacillus flagellatus*
Bacillota	Tumebacillaceae	*Tumebacillus*	*Tumebacillus ginsengisoli*
Bacillota	Tumebacillaceae	*Tumebacillus*	*Tumebacillus lipolyticus*
Bacillota	Tumebacillaceae	*Tumebacillus*	*Tumebacillus permanentifrigoris*
Bacillota	Tumebacillaceae	*Tumebacillus*	*Tumebacillus soli*
Bacillota	Turicibacteraceae	*Turicibacter*	*Turicibacter sanguinis*

Bacillota	Lachnospiraceae	*Tyzzerella*	*Tyzzerella nexilis*
Bacillota	Mycoplasmoidaceae	*Ureaplasma*	*Ureaplasma canigenitalium*
Bacillota	Mycoplasmoidaceae	*Ureaplasma*	*Ureaplasma diversum*
Bacillota	Mycoplasmoidaceae	*Ureaplasma*	*Ureaplasma felinum*
Bacillota	Mycoplasmoidaceae	*Ureaplasma*	*Ureaplasma gallorale*
Bacillota	Mycoplasmoidaceae	*Ureaplasma*	*Ureaplasma parvum*
Bacillota	Mycoplasmoidaceae	*Ureaplasma*	*Ureaplasma urealyticum*
Bacillota	Planococcaceae	*Ureibacillus*	*Ureibacillus chungkukjangi*
Bacillota	Planococcaceae	*Ureibacillus*	*Ureibacillus composti*
Bacillota	Planococcaceae	*Ureibacillus*	*Ureibacillus manganicus*
Bacillota	Planococcaceae	*Ureibacillus*	*Ureibacillus thermophilus*
Bacillota	Planococcaceae	*Ureibacillus*	*Ureibacillus thermosphaericus*
Bacillota	Tissierellaceae	*Urmitella*	*Urmitella timonensis*
Bacillota	Vagococcaceae	*Vagococcus*	*Vagococcus acidifermentans*
Bacillota	Vagococcaceae	*Vagococcus*	*Vagococcus carniphilus*
Bacillota	Vagococcaceae	*Vagococcus*	*Vagococcus entomophilus*
Bacillota	Vagococcaceae	*Vagococcus*	*Vagococcus fessus*
Bacillota	Vagococcaceae	*Vagococcus*	*Vagococcus fluvialis*
Bacillota	Vagococcaceae	*Vagococcus*	*Vagococcus humatus*
Bacillota	Vagococcaceae	*Vagococcus*	*Vagococcus lutrae*
Bacillota	Vagococcaceae	*Vagococcus*	*Vagococcus martis*
Bacillota	Vagococcaceae	*Vagococcus*	*Vagococcus penaei*
Bacillota	Vagococcaceae	*Vagococcus*	*Vagococcus salmoninarum*
Bacillota	Vagococcaceae	*Vagococcus*	*Vagococcus teuberi*
Bacillota	Vallitaleaceae	*Vallitalea*	*Vallitalea guaymasensis*
Bacillota	Vallitaleaceae	*Vallitalea*	*Vallitalea pronyensis*
Bacillota	Lachnospiraceae	*Variimorphobacter*	*Variimorphobacter saccharofermentans*
Bacillota	Veillonellaceae	*Veillonella*	*Veillonella atypica*

Bacillota	Veillonellaceae	*Veillonella*	*Veillonella caviae*
Bacillota	Veillonellaceae	*Veillonella*	*Veillonella criceti*
Bacillota	Veillonellaceae	*Veillonella*	*Veillonella denticariosi*
Bacillota	Veillonellaceae	*Veillonella*	*Veillonella dispar*
Bacillota	Veillonellaceae	*Veillonella*	*Veillonella infantium*
Bacillota	Veillonellaceae	*Veillonella*	*Veillonella magna*
Bacillota	Veillonellaceae	*Veillonella*	*Veillonella parvula*
Bacillota	Veillonellaceae	*Veillonella*	*Veillonella ratti*
Bacillota	Veillonellaceae	*Veillonella*	*Veillonella tobetsuensis*
Bacillota	Amphibacillaceae	*Virgibacillus*	*Virgibacillus campisalis*
Bacillota	Amphibacillaceae	*Virgibacillus*	*Virgibacillus dokdonensis*
Bacillota	Amphibacillaceae	*Virgibacillus*	*Virgibacillus halodenitrificans*
Bacillota	Amphibacillaceae	*Virgibacillus*	*Virgibacillus halophilus*
Bacillota	Amphibacillaceae	*Virgibacillus*	*Virgibacillus kekensis*
Bacillota	Amphibacillaceae	*Virgibacillus*	*Virgibacillus litoralis*
Bacillota	Amphibacillaceae	*Virgibacillus*	*Virgibacillus marismortui*
Bacillota	Amphibacillaceae	*Virgibacillus*	*Virgibacillus massiliensis*
Bacillota	Amphibacillaceae	*Virgibacillus*	*Virgibacillus necropolis*
Bacillota	Amphibacillaceae	*Virgibacillus*	*Virgibacillus olivae*
Bacillota	Amphibacillaceae	*Virgibacillus*	*Virgibacillus pantothenticus*
Bacillota	Amphibacillaceae	*Virgibacillus*	*Virgibacillus proomii*
Bacillota	Amphibacillaceae	*Virgibacillus*	*Virgibacillus soli*
Bacillota	Planococcaceae	*Viridibacillus*	*Viridibacillus arenosi*
Bacillota	Planococcaceae	*Viridibacillus*	*Viridibacillus arvi*
Bacillota	Lactobacillaceae	*Weissella*	*Weissella beninensis*
Bacillota	Lactobacillaceae	*Weissella*	*Weissella bombi*
Bacillota	Lactobacillaceae	*Weissella*	*Weissella ceti*
Bacillota	Lactobacillaceae	*Weissella*	*Weissella cibaria*

Bacillota	Lactobacillaceae	*Weissella*	*Weissella confusa*
Bacillota	Lactobacillaceae	*Weissella*	*Weissella cryptocerci*
Bacillota	Lactobacillaceae	*Weissella*	*Weissella diestrammenae*
Bacillota	Lactobacillaceae	*Weissella*	*Weissella fabalis*
Bacillota	Lactobacillaceae	*Weissella*	*Weissella fabaria*
Bacillota	Lactobacillaceae	*Weissella*	*Weissella ghanensis*
Bacillota	Lactobacillaceae	*Weissella*	*Weissella halotolerans*
Bacillota	Lactobacillaceae	*Weissella*	*Weissella hellenica*
Bacillota	Lactobacillaceae	*Weissella*	*Weissella jogaejeotgali*
Bacillota	Lactobacillaceae	*Weissella*	*Weissella kandleri*
Bacillota	Lactobacillaceae	*Weissella*	*Weissella koreensis*
Bacillota	Lactobacillaceae	*Weissella*	*Weissella minor*
Bacillota	Lactobacillaceae	*Weissella*	*Weissella oryzae*
Bacillota	Lactobacillaceae	*Weissella*	*Weissella paramesenteroides*
Bacillota	Lactobacillaceae	*Weissella*	*Weissella soli*
Bacillota	Lactobacillaceae	*Weissella*	*Weissella thailandensis*
Bacillota	Lactobacillaceae	*Weissella*	*Weissella uvarum*
Bacillota	Lactobacillaceae	*Weissella*	*Weissella viridescens*
Bacillota	Bacillaceae	*Weizmannia*	*Weizmannia ginsengihum*
Bacillota	Clostridiaceae	*Wukongibacter*	*Wukongibacter baidiensis*
Bacillota	Paenibacillaceae	*Xylanibacillus*	*Xylanibacillus composti*
Bacillota	Clostridiaceae	*Youngiibacter*	*Youngiibacter fragilis*
Bacillota	Clostridiaceae	*Youngiibacter*	*Youngiibacter multivorans*
Bacteroidota	Rikenellaceae	*Acetobacteroides*	*Acetobacteroides hydrogenigenes*
Bacteroidota	Rikenellaceae	*Alistipes*	*Alistipes finegoldii*
Bacteroidota	Rikenellaceae	*Alistipes*	*Alistipes ihumii*
Bacteroidota	Rikenellaceae	*Alistipes*	*Alistipes indistinctus*
Bacteroidota	Rikenellaceae	*Alistipes*	*Alistipes putredinis*

Bacteroidota	Rikenellaceae	*Alistipes*	*Alistipes shahii*
Bacteroidota	Rikenellaceae	*Alistipes*	*Alistipes timonensis*
Bacteroidota	Rikenellaceae	*Ammoniphilus*	*Ammoniphilus oxalaticus*
Bacteroidota	Rikenellaceae	*Ammoniphilus*	*Ammoniphilus oxalivorans*
Bacteroidota	Rikenellaceae	*Anaerocella*	*Anaerocella delicata*
Bacteroidota	Marinilabiliaceae	*Anaerophaga*	*Anaerophaga thermohalophila*
Bacteroidota	Bacteroidaceae	*Bacteroides*	*Bacteroides acidifaciens*
Bacteroidota	Bacteroidaceae	*Bacteroides*	*Bacteroides caecimuris*
Bacteroidota	Bacteroidaceae	*Bacteroides*	*Bacteroides cellulosilyticus*
Bacteroidota	Bacteroidaceae	*Bacteroides*	*Bacteroides coprophilus*
Bacteroidota	Bacteroidaceae	*Bacteroides*	*Bacteroides eggerthii*
Bacteroidota	Bacteroidaceae	*Bacteroides*	*Bacteroides faecichinchillae*
Bacteroidota	Bacteroidaceae	*Bacteroides*	*Bacteroides faecis*
Bacteroidota	Bacteroidaceae	*Bacteroides*	*Bacteroides finegoldii*
Bacteroidota	Bacteroidaceae	*Bacteroides*	*Bacteroides fragilis*
Bacteroidota	Bacteroidaceae	*Bacteroides*	*Bacteroides graminisolvens*
Bacteroidota	Bacteroidaceae	*Bacteroides*	*Bacteroides intestinalis*
Bacteroidota	Bacteroidaceae	*Bacteroides*	*Bacteroides luti*
Bacteroidota	Bacteroidaceae	*Bacteroides*	*Bacteroides nordii*
Bacteroidota	Bacteroidaceae	*Bacteroides*	*Bacteroides oleiciplenus*
Bacteroidota	Bacteroidaceae	*Bacteroides*	*Bacteroides pectinophilus*
Bacteroidota	Bacteroidaceae	*Bacteroides*	*Bacteroides reticulotermitis*
Bacteroidota	Bacteroidaceae	*Bacteroides*	*Bacteroides rodentium*
Bacteroidota	Bacteroidaceae	*Bacteroides*	*Bacteroides salyersiae*
Bacteroidota	Bacteroidaceae	*Bacteroides*	*Bacteroides stercorirosoris*
Bacteroidota	Bacteroidaceae	*Bacteroides*	*Bacteroides thetaiotaomicron*
Bacteroidota	Bacteroidaceae	*Bacteroides*	*Bacteroides uniformis*
Bacteroidota	Bacteroidaceae	*Bacteroides*	*Bacteroides xylanisolvens*

Bacteroidota	Bacteroidaceae	*Bacteroides*	*Bacteroides xylanolyticus*
Bacteroidota	Barnesiellaceae	*Barnesiella*	*Barnesiella viscericola*
Bacteroidota	Flavobacteriaceae	*Bizionia*	*Bizionia hallyeonensis*
Bacteroidota	Marinifilaceae	*Butyricimonas*	*Butyricimonas paravirosa*
Bacteroidota	Marinifilaceae	*Butyricimonas*	*Butyricimonas synergistica*
Bacteroidota	Marinifilaceae	*Butyricimonas*	*Butyricimonas virosa*
Bacteroidota	Weeksellaceae	*Chryseobacterium*	*Chryseobacterium joostei*
Bacteroidota	Weeksellaceae	*Chryseobacterium*	*Chryseobacterium montanum*
Bacteroidota	Weeksellaceae	*Chryseobacterium*	*Chryseobacterium shigense*
Bacteroidota	Odoribacteraceae	*Culturomica*	*Culturomica massiliensis*
Bacteroidota	Muribaculaceae	*Duncaniella*	*Duncaniella freteri*
Bacteroidota	Dysgonomonadaceae	*Dysgonomonas*	*Dysgonomonas alginatilytica*
Bacteroidota	Dysgonomonadaceae	*Dysgonomonas*	*Dysgonomonas capnocytophagoides*
Bacteroidota	Dysgonomonadaceae	*Dysgonomonas*	*Dysgonomonas gadei*
Bacteroidota	Dysgonomonadaceae	*Dysgonomonas*	*Dysgonomonas macrotermitis*
Bacteroidota	Dysgonomonadaceae	*Dysgonomonas*	*Dysgonomonas massiliensis*
Bacteroidota	Dysgonomonadaceae	*Dysgonomonas*	*Dysgonomonas mossii*
Bacteroidota	Dysgonomonadaceae	*Dysgonomonas*	*Dysgonomonas oryzarvi*
Bacteroidota	Weeksellaceae	*Elizabethkingia*	*Elizabethkingia anophelis*
Bacteroidota	Chitinophagaceae	*Flavisolibacter*	*Flavisolibacter metallilatus*
Bacteroidota	Chitinophagaceae	*Flavitalea*	*Flavitalea flava*
Bacteroidota	Flavobacteriaceae	*Flavobacterium*	*Flavobacterium acidificum*
Bacteroidota	Marinilabiliaceae	*Geofilum*	*Geofilum rhodophaeum*
Bacteroidota	Marinilabiliaceae	*Geofilum*	*Geofilum rubicundum*
Bacteroidota	Lentimicrobiaceae	*Lentimicrobium*	*Lentimicrobium saccharophilum*
Bacteroidota	Salinibacteraceae	*Longimonas*	*Longimonas halophila*
Bacteroidota	Porphyromonadaceae	*Macellibacteroides*	*Macellibacteroides fermentans*
Bacteroidota	Prolixibacteraceae	*Mangrovibacterium*	*Mangrovibacterium marinum*

Bacteroidota	Prolixibacteraceae	*Maribellus*	*Maribellus luteus*
Bacteroidota	Rikenellaceae	*Millionella*	*Millionella massiliensis*
Bacteroidota	Sphingobacteriaceae	*Mucilaginibacter*	*Mucilaginibacter craterilacus*
Bacteroidota	Sphingobacteriaceae	*Mucilaginibacter*	*Mucilaginibacter yixingensis*
Bacteroidota	Rikenellaceae	*Mucinivorans*	*Mucinivorans hirudinis*
Bacteroidota	Marinifilaceae	*Odoribacter*	*Odoribacter laneus*
Bacteroidota	Marinifilaceae	*Odoribacter*	*Odoribacter splanchnicus*
Bacteroidota	Sphingobacteriaceae	*Olivibacter*	*Olivibacter oleidegradans*
Bacteroidota	Paludibacteraceae	*Paludibacter*	*Paludibacter jiangxiensis*
Bacteroidota	Paludibacteraceae	*Paludibacter*	*Paludibacter propionicigenes*
Bacteroidota	Tannerellaceae	*Parabacteroides*	*Parabacteroides chinchillae*
Bacteroidota	Tannerellaceae	*Parabacteroides*	*Parabacteroides chongii*
Bacteroidota	Tannerellaceae	*Parabacteroides*	*Parabacteroides faecis*
Bacteroidota	Tannerellaceae	*Parabacteroides*	*Parabacteroides goldsteinii*
Bacteroidota	Tannerellaceae	*Parabacteroides*	*Parabacteroides gordonii*
Bacteroidota	Tannerellaceae	*Parabacteroides*	*Parabacteroides johnsonii*
Bacteroidota	Tannerellaceae	*Parabacteroides*	*Parabacteroides merdae*
Bacteroidota	Bacteroidaceae	*Paraprevotella*	*Paraprevotella clara*
Bacteroidota	Sphingobacteriaceae	*Pedobacter*	*Pedobacter agrisoli*
Bacteroidota	Bacteroidaceae	*Phocaeicola*	*Phocaeicola coprophilus*
Bacteroidota	Bacteroidaceae	*Phocaeicola*	*Phocaeicola paurosaccharolyticus*
Bacteroidota	Bacteroidaceae	*Phocaeicola*	*Phocaeicola vulgatus*
Bacteroidota	Hymenobacteraceae	*Pontibacter*	*Pontibacter mucosus*
Bacteroidota	Porphyromonadaceae	*Porphyromonas*	*Porphyromonas catoniae*
Bacteroidota	Porphyromonadaceae	*Porphyromonas*	*Porphyromonas circumdentaria*
Bacteroidota	Bacteroidaceae	*Prevotellamassilia*	*Prevotellamassilia timonensis*
Bacteroidota	Dysgonomonadaceae	*Proteiniphilum*	*Proteiniphilum acetatigenes*
Bacteroidota	Thermonemataceae	*Raineya*	*Raineya orbicola*

Bacteroidota	Rhodothermaceae	*Rhodothermus*	*Rhodothermus marinus*
Bacteroidota	Rikenellaceae	*Rikenella*	*Rikenella microfusus*
Bacteroidota	Marinilabiliaceae	*Saccharicrinis*	*Saccharicrinis aurantiacus*
Bacteroidota	Marinilabiliaceae	*Saccharicrinis*	*Saccharicrinis carchari*
Bacteroidota	Salibacteraceae	*Salibacter*	*Salibacter halophilus*
Bacteroidota	Flavobacteriaceae	*Sediminibacter*	*Sediminibacter furfurosus*
Bacteroidota	Tannerellaceae	*Tannerella*	*Tannerella forsythia*
Bacteroidota	Chitinophagaceae	*Terrimonas*	*Terrimonas terrae*
Bacteroidota	Rikenellaceae	*Tidjanibacter*	*Tidjanibacter massiliensis*
Bacteroidota	Williamwhitmaniaceae	*Williamwhitmania*	*Williamwhitmania taraxaci*
Bdellovibrionota	Bdellovibrionaceae	*Bdellovibrio*	*Bdellovibrio exovorus*
Campylobacterota	Arcobacteraceae	*Aliarcobacter*	*Aliarcobacter cibarius*
Campylobacterota	Arcobacteraceae	*Arcobacter*	*Arcobacter aquimarinus*
Campylobacterota	Campylobacteraceae	*Campylobacter*	*Campylobacter fetus*
Campylobacterota	Campylobacteraceae	*Campylobacter*	*Campylobacter iguaniorum*
Campylobacterota	Campylobacteraceae	*Campylobacter*	*Campylobacter insulaenigrae*
Campylobacterota	Campylobacteraceae	*Campylobacter*	*Campylobacter subantarcticus*
Campylobacterota	Campylobacteraceae	*Campylobacter*	*Campylobacter troglodytis*
Campylobacterota	Campylobacteraceae	*Campylobacter*	*Campylobacter volucris*
Campylobacterota	Helicobacteraceae	*Helicobacter*	*Helicobacter anseris*
Campylobacterota	Helicobacteraceae	*Helicobacter*	*Helicobacter aurati*
Campylobacterota	Helicobacteraceae	*Helicobacter*	*Helicobacter bilis*
Campylobacterota	Helicobacteraceae	*Helicobacter*	*Helicobacter brantae*
Campylobacterota	Helicobacteraceae	*Helicobacter*	*Helicobacter canadensis*
Campylobacterota	Helicobacteraceae	*Helicobacter*	*Helicobacter canis*
Campylobacterota	Helicobacteraceae	*Helicobacter*	*Helicobacter cetorum*
Campylobacterota	Helicobacteraceae	*Helicobacter*	*Helicobacter cholecystus*
Campylobacterota	Helicobacteraceae	*Helicobacter*	*Helicobacter cinaedi*

Campylobacterota	Helicobacteraceae	*Helicobacter*	*Helicobacter equorum*
Campylobacterota	Helicobacteraceae	*Helicobacter*	*Helicobacter fennelliae*
Campylobacterota	Helicobacteraceae	*Helicobacter*	*Helicobacter ganmani*
Campylobacterota	Helicobacteraceae	*Helicobacter*	*Helicobacter hepaticus*
Campylobacterota	Helicobacteraceae	*Helicobacter*	*Helicobacter himalayensis*
Campylobacterota	Helicobacteraceae	*Helicobacter*	*Helicobacter jaachi*
Campylobacterota	Helicobacteraceae	*Helicobacter*	*Helicobacter japonicus*
Campylobacterota	Helicobacteraceae	*Helicobacter*	*Helicobacter marmotae*
Campylobacterota	Helicobacteraceae	*Helicobacter*	*Helicobacter mastomyrinus*
Campylobacterota	Helicobacteraceae	*Helicobacter*	*Helicobacter mesocricetorum*
Campylobacterota	Helicobacteraceae	*Helicobacter*	*Helicobacter muridarum*
Campylobacterota	Helicobacteraceae	*Helicobacter*	*Helicobacter mustelae*
Campylobacterota	Helicobacteraceae	*Helicobacter*	*Helicobacter pametensis*
Campylobacterota	Helicobacteraceae	*Helicobacter*	*Helicobacter pullorum*
Campylobacterota	Helicobacteraceae	*Helicobacter*	*Helicobacter pylori*
Campylobacterota	Helicobacteraceae	*Helicobacter*	*Helicobacter rodentium*
Campylobacterota	Helicobacteraceae	*Helicobacter*	*Helicobacter trogontum*
Campylobacterota	Helicobacteraceae	*Helicobacter*	*Helicobacter typhlonius*
Campylobacterota	Helicobacteraceae	*Helicobacter*	*Helicobacter valdiviensis*
Campylobacterota	Helicobacteraceae	*Helicobacter*	*Helicobacter winghamensis*
Campylobacterota	Helicobacteraceae	*Heliomicrobium*	*Heliomicrobium modesticaldum*
Campylobacterota	Hippeaceae	*Hippea*	*Hippea jasoniae*
Campylobacterota	Hippeaceae	*Hippea*	*Hippea maritima*
Campylobacterota	Arcobacteraceae	*Malaciobacter*	*Malaciobacter mytili*
Campylobacterota	Sulfurospirillaceae	*Sulfurospirillum*	*Sulfurospirillum multivorans*
Campylobacterota	Helicobacteraceae	*Wolinella*	*Wolinella succinogenes*
Chloroflexota	Caldilineaceae	*Caldilinea*	*Caldilinea tarbellica*
Chloroflexota	Herpetosiphonaceae	*Herpetosiphon*	*Herpetosiphon giganteus*

Chloroflexota	Herpetosiphonaceae	*Herpetosiphon*	*Herpetosiphon gulosus*
Chloroflexota	Thermomicrobiaceae	*Sphaerobacter*	*Sphaerobacter thermophilus*
Chrysiogenetota	Chrysiogenaceae	*Chrysiogenes*	*Chrysiogenes arsenatis*
Chrysiogenetota	Chrysiogenaceae	*Desulfurispirillum*	*Desulfurispirillum indicum*
Cyanobacteriota	Thermosynechococcaceae	*Acaryochloris*	*Acaryochloris marina*
Cyanobacteriota	Oscillatoriaceae	*Aerosakkonema*	*Aerosakkonema funiforme*
Cyanobacteriota	Chroococcidiopsidaceae	*Aliterella*	*Aliterella antarctica*
Cyanobacteriota	Nostocaceae	*Anabaena*	*Anabaena cylindrica*
Cyanobacteriota	Scytonemataceae	*Brasilonema*	*Brasilonema angustatum*
Cyanobacteriota	Scytonemataceae	*Brasilonema*	*Brasilonema bromeliae*
Cyanobacteriota	Scytonemataceae	*Brasilonema*	*Brasilonema terrestre*
Cyanobacteriota	Nostocaceae	*Calothrix*	*Calothrix desertica*
Cyanobacteriota	Coleofasciculaceae	*Cephalothrix*	*Cephalothrix komarekiana*
Cyanobacteriota	Coleofasciculaceae	*Cephalothrix*	*Cephalothrix lacustris*
Cyanobacteriota	Chamaesiphonaceae	*Chamaesiphon*	*Chamaesiphon minutus*
Cyanobacteriota	Chroococcidiopsidaceae	*Chroococcidiopsis*	*Chroococcidiopsis thermalis*
Cyanobacteriota	Chamaesiphonaceae	*Crinalium*	*Crinalium epipsammum*
Cyanobacteriota	Nostocaceae	*Cylindrospermum*	*Cylindrospermum pellucidum*
Cyanobacteriota	Nostocaceae	*Cylindrospermum*	*Cylindrospermum stagnale*
Cyanobacteriota	Dapisostemonaceae	*Dapisostemon*	*Dapisostemon apicaliramis*
Cyanobacteriota	Calotrichaceae	*Dulcicalothrix*	*Dulcicalothrix necridiiformans*
Cyanobacteriota	Gloeobacteraceae	*Gloeobacter*	*Gloeobacter kilaueensis*
Cyanobacteriota	Gloeobacteraceae	*Gloeobacter*	*Gloeobacter violaceus*
Cyanobacteriota	Chroococcaceae	*Gloeocapsopsis*	*Gloeocapsopsis crepidinum*
Cyanobacteriota	Chroococcaceae	*Gloeocapsopsis*	*Gloeocapsopsis diffluens*
Cyanobacteriota	Microcystaceae	*Gloeothece*	*Gloeothece membranacea*
Cyanobacteriota	Prochlorotrichaceae	*Haloleptolyngbya*	*Haloleptolyngbya alcalis*
Cyanobacteriota	Prochlorotrichaceae	*Halomicronema*	*Halomicronema excentricum*

Cyanobacteriota	Nostocaceae	*Komarekiella*	*Komarekiella atlantica*
Cyanobacteriota	Tolypothrichaceae	*Kryptousia*	*Kryptousia macronema*
Cyanobacteriota	Tolypothrichaceae	*Kryptousia*	*Kryptousia microlepis*
Cyanobacteriota	Leptolyngbyaceae	*Leptodesmis*	*Leptodesmis alaskaensis*
Cyanobacteriota	Symphyonemataceae	*Loriellopsis*	*Loriellopsis cavernicola*
Cyanobacteriota	Phormidiaceae	*Lyngbya*	*Lyngbya aestuarii*
Cyanobacteriota	Phormidiaceae	*Microcoleus*	*Microcoleus anatoxicus*
Cyanobacteriota	Microcystaceae	*Microcystis*	*Microcystis aeruginosa*
Cyanobacteriota	Phormidiaceae	*Okeania*	*Okeania plumata*
Cyanobacteriota	Oscillatoriaceae	*Oscillatoria*	*Oscillatoria acuminata*
Cyanobacteriota	Oscillatoriaceae	*Oscillatoria*	*Oscillatoria nigro-viridis*
Cyanobacteriota	Oscillatoriaceae	*Oscillatoria*	*Oscillatoria sancta*
Cyanobacteriota	Phormidiaceae	*Planktothrix*	*Planktothrix agardhii*
Cyanobacteriota	Phormidiaceae	*Planktothrix*	*Planktothrix spiroides*
Cyanobacteriota	Prochlorotrichaceae	*Prochlorothrix*	*Prochlorothrix hollandica*
Cyanobacteriota	Chroococcaceae	*Pseudochroococcus*	*Pseudochroococcus couteii*
Cyanobacteriota	Spirulinaceae	*Spirulina*	*Spirulina major*
Cyanobacteriota	Xenococcaceae	*Stanieria*	*Stanieria cyanosphaera*
Cyanobacteriota	Symphyonemataceae	*Symphyonema*	*Symphyonema bifilamentata*
Cyanobacteriota	Microcoleaceae	*Tychonema*	*Tychonema bourrellyi*
Cyanobacteriota	Vampirovibrionaceae	*Vampirovibrio*	*Vampirovibrio chlorellavorus*
Deferribacterota	Calditerrivibrionaceae	*Calditerrivibrio*	*Calditerrivibrio nitroreducens*
Deferribacterota	Deferribacteraceae	*Deferribacter*	*Deferribacter autotrophicus*
Deferribacterota	Deferribacteraceae	*Deferribacter*	*Deferribacter desulfuricans*
Deferribacterota	Denitrovibrionaceae	*Geovibrio*	*Geovibrio ferrireducens*
Deferribacterota	Mucispirillaceae	*Mucispirillum*	*Mucispirillum schaedleri*
Desulfobacterota	Desulfobacteraceae	*Algorimarina*	*Algorimarina butyrica*
Desulfobacterota	Geobacteraceae	*Citrifermentans*	*Citrifermentans bemidjiense*

Desulfobacterota	Desulfovibrionaceae	*Cupidesulfovibrio*	*Cupidesulfovibrio oxamicus*
Desulfobacterota	Desulfovibrionaceae	*Cupidesulfovibrio*	*Cupidesulfovibrio termitidis*
Desulfobacterota	Desulfarculaceae	*Desulfarculus*	*Desulfarculus baarsii*
Desulfobacterota	Desulfatibacillaceae	*Desulfatibacillum*	*Desulfatibacillum alkenivorans*
Desulfobacterota	Desulfobacteraceae	*Desulfatiferula*	*Desulfatiferula berrensis*
Desulfobacterota	Desulfobacteraceae	*Desulfatiferula*	*Desulfatiferula olefinivorans*
Desulfobacterota	Desulfatiglandaceae	*Desulfatiglans*	*Desulfatiglans anilini*
Desulfobacterota	Desulfatiglandaceae	*Desulfatiglans*	*Desulfatiglans parachlorophenolica*
Desulfobacterota	Desulfatirhabdiaceae	*Desulfatirhabdium*	*Desulfatirhabdium butyrativorans*
Desulfobacterota	Desulfosarcinaceae	*Desulfatitalea*	*Desulfatitalea tepidiphila*
Desulfobacterota	Desulfobacteraceae	*Desulfobacter*	*Desulfobacter curvatus*
Desulfobacterota	Desulfobacteraceae	*Desulfobacter*	*Desulfobacter hydrogenophilus*
Desulfobacterota	Desulfobacteraceae	*Desulfobacter*	*Desulfobacter latus*
Desulfobacterota	Desulfobacteraceae	*Desulfobacterium*	*Desulfobacterium indolicum*
Desulfobacterota	Desulfobacteraceae	*Desulfobacula*	*Desulfobacula phenolica*
Desulfobacterota	Desulfobacteraceae	*Desulfobacula*	*Desulfobacula toluolica*
Desulfobacterota	Desulforegulaceae	*Desulfobotulus*	*Desulfobotulus mexicanus*
Desulfobacterota	Desulforegulaceae	*Desulfobotulus*	*Desulfobotulus sapovorans*
Desulfobacterota	Desulfobulbaceae	*Desulfobulbus*	*Desulfobulbus oligotrophicus*
Desulfobacterota	Desulfobulbaceae	*Desulfobulbus*	*Desulfobulbus propionicus*
Desulfobacterota	Desulfococcaceae	*Desulfococcus*	*Desulfococcus biacutus*
Desulfobacterota	Desulfococcaceae	*Desulfococcus*	*Desulfococcus multivorans*
Desulfobacterota	Desulfofabaceae	*Desulfofaba*	*Desulfofaba fastidiosa*
Desulfobacterota	Desulfofabaceae	*Desulfofaba*	*Desulfofaba gelida*
Desulfobacterota	Desulfofabaceae	*Desulfofaba*	*Desulfofaba hansenii*
Desulfobacterota	Desulfobacteraceae	*Desulfofrigus*	*Desulfofrigus oceanense*
Desulfobacterota	Syntrophobacteraceae	*Desulfoglaeba*	*Desulfoglaeba alkanexedens*
Desulfobacterota	Desulfolunaceae	*Desulfoluna*	*Desulfoluna spongiiphila*

Desulfobacterota	Desulfomicrobiaceae	*Desulfomicrobium*	*Desulfomicrobium baculatum*
Desulfobacterota	Desulfomonilaceae	*Desulfomonile*	*Desulfomonile limimaris*
Desulfobacterota	Desulfomonilaceae	*Desulfomonile*	*Desulfomonile tiedjei*
Desulfobacterota	Desulfobacteraceae	*Desulfonatronobacter*	*Desulfonatronobacter acetoxydans*
Desulfobacterota	Desulfobacteraceae	*Desulfonatronobacter*	*Desulfonatronobacter acidivorans*
Desulfobacterota	Desulfonatronovibrionaceae	*Desulfonatronovibrio*	*Desulfonatronovibrio halophilus*
Desulfobacterota	Desulfonatronovibrionaceae	*Desulfonatronovibrio*	*Desulfonatronovibrio hydrogenovorans*
Desulfobacterota	Desulfonatronovibrionaceae	*Desulfonatronovibrio*	*Desulfonatronovibrio magnus*
Desulfobacterota	Desulfonatronaceae	*Desulfonatronum*	*Desulfonatronum lacustre*
Desulfobacterota	Desulfonatronaceae	*Desulfonatronum*	*Desulfonatronum thioautotrophicum*
Desulfobacterota	Desulfococcaceae	*Desulfonema*	*Desulfonema ishimotonii*
Desulfobacterota	Desulfococcaceae	*Desulfonema*	*Desulfonema limicola*
Desulfobacterota	Desulfococcaceae	*Desulfonema*	*Desulfonema magnum*
Desulfobacterota	Desulfoplanaceae	*Desulfoplanes*	*Desulfoplanes formicivorans*
Desulfobacterota	Desulfobacteraceae	*Desulforapulum*	*Desulforapulum autotrophicum*
Desulfobacterota	Desulforegulaceae	*Desulforegula*	*Desulforegula conservatrix*
Desulfobacterota	Desulfosalsimonadaceae	*Desulfosalsimonas*	*Desulfosalsimonas propionicica*
Desulfobacterota	Desulfosarcinaceae	*Desulfosarcina*	*Desulfosarcina alkanivorans*
Desulfobacterota	Desulfosarcinaceae	*Desulfosarcina*	*Desulfosarcina cetonica*
Desulfobacterota	Desulfosarcinaceae	*Desulfosarcina*	*Desulfosarcina variabilis*
Desulfobacterota	Desulfosarcinaceae	*Desulfosarcina*	*Desulfosarcina widdelii*
Desulfobacterota	Desulfovibrionaceae	*Desulfovibrio*	*Desulfovibrio alaskensis*
Desulfobacterota	Desulfovibrionaceae	*Desulfovibrio*	*Desulfovibrio carbinoliphilus*
Desulfobacterota	Desulfovibrionaceae	*Desulfovibrio*	*Desulfovibrio cuneatus*
Desulfobacterota	Desulfovibrionaceae	*Desulfovibrio*	*Desulfovibrio desulfuricans*
Desulfobacterota	Desulfovibrionaceae	*Desulfovibrio*	*Desulfovibrio gigas*
Desulfobacterota	Desulfovibrionaceae	*Desulfovibrio*	*Desulfovibrio inopinatus*
Desulfobacterota	Desulfovibrionaceae	*Desulfovibrio*	*Desulfovibrio intestinalis*

Desulfobacterota	Desulfovibrionaceae	*Desulfovibrio*	*Desulfovibrio legallii*
Desulfobacterota	Desulfovibrionaceae	*Desulfovibrio*	*Desulfovibrio litoralis*
Desulfobacterota	Desulfovibrionaceae	*Desulfovibrio*	*Desulfovibrio longreachensis*
Desulfobacterota	Desulfovibrionaceae	*Desulfovibrio*	*Desulfovibrio oxamicus*
Desulfobacterota	Desulfovibrionaceae	*Desulfovibrio*	*Desulfovibrio piger*
Desulfobacterota	Desulfovibrionaceae	*Desulfovibrio*	*Desulfovibrio psychrotolerans*
Desulfobacterota	Desulfovibrionaceae	*Desulfovibrio*	*Desulfovibrio senegalensis*
Desulfobacterota	Desulfovibrionaceae	*Desulfovibrio*	*Desulfovibrio simplex*
Desulfobacterota	Desulfovibrionaceae	*Desulfovibrio*	*Desulfovibrio tunisiensis*
Desulfobacterota	Desulfovibrionaceae	*Desulfovibrio*	*Desulfovibrio vietnamensis*
Desulfobacterota	Desulfovibrionaceae	*Desulfovibrio*	*Desulfovibrio vulgaris*
Desulfobacterota	Syntrophobacteraceae	*Desulfovirga*	*Desulfovirga adipica*
Desulfobacterota	Desulfuromonadaceae	*Desulfuromonas*	*Desulfuromonas acetoxidans*
Desulfobacterota	Desulfuromonadaceae	*Desulfuromonas*	*Desulfuromonas michiganensis*
Desulfobacterota	Desulfuromonadaceae	*Desulfuromonas*	*Desulfuromonas palmitatis*
Desulfobacterota	Desulfuromonadaceae	*Desulfuromonas*	*Desulfuromonas svalbardensis*
Desulfobacterota	Desulfuromonadaceae	*Desulfuromonas*	*Desulfuromonas thiophila*
Desulfobacterota	Desulfarculaceae	*Dethiosulfatarculus*	*Dethiosulfatarculus sandiegensis*
Desulfobacterota	Geoalkalibacteraceae	*Geoalkalibacter*	*Geoalkalibacter ferrihydriticus*
Desulfobacterota	Geobacteraceae	*Geobacter*	*Geobacter anodireducens*
Desulfobacterota	Geobacteraceae	*Geobacter*	*Geobacter chapellei*
Desulfobacterota	Geobacteraceae	*Geobacter*	*Geobacter grbiciae*
Desulfobacterota	Geobacteraceae	*Geobacter*	*Geobacter lovleyi*
Desulfobacterota	Geobacteraceae	*Geobacter*	*Geobacter luticola*
Desulfobacterota	Geobacteraceae	*Geobacter*	*Geobacter metallireducens*
Desulfobacterota	Geobacteraceae	*Geobacter*	*Geobacter pickeringii*
Desulfobacterota	Geobacteraceae	*Geobacter*	*Geobacter psychrophilus*
Desulfobacterota	Geobacteraceae	*Geobacter*	*Geobacter soli*

Desulfobacterota	Geobacteraceae	*Geobacter*	*Geobacter sulfurreducens*
Desulfobacterota	Geobacteraceae	*Geobacter*	*Geobacter thiogenes*
Desulfobacterota	Geobacteraceae	*Geobacter*	*Geobacter uraniireducens*
Desulfobacterota	Desulfovibrionaceae	*Lawsonia*	*Lawsonia intracellularis*
Desulfobacterota	Desulfovibrionaceae	*Mailhella*	*Mailhella massiliensis*
Desulfobacterota	Desulfovibrionaceae	*Maridesulfovibrio*	*Maridesulfovibrio hydrothermalis*
Desulfobacterota	Desulfuromonadaceae	*Pelobacter*	*Pelobacter carbinolicus*
Desulfobacterota	Desulfuromonadaceae	*Pelobacter*	*Pelobacter propionicus*
Desulfobacterota	Desulfuromonadaceae	*Pelobacter*	*Pelobacter seleniigenes*
Desulfobacterota	Syntrophobacteraceae	*Pelospora*	*Pelospora glutarica*
Desulfobacterota	Desulfovibrionaceae	*Pseudodesulfovibrio*	*Pseudodesulfovibrio aespoeensis*
Desulfobacterota	Desulfovibrionaceae	*Pseudodesulfovibrio*	*Pseudodesulfovibrio alkaliphilus*
Desulfobacterota	Syntrophobacteraceae	*Syntrophobacter*	*Syntrophobacter fumaroxidans*
Desulfobacterota	Syntrophobacteraceae	*Syntrophobacter*	*Syntrophobacter sulfatireducens*
Desulfobacterota	Syntrophorhabdaceae	*Syntrophorhabdus*	*Syntrophorhabdus aromaticivorans*
Desulfobacterota	Syntrophotaleaceae	*Syntrophotalea*	*Syntrophotalea acetylenica*
Desulfobacterota	Syntrophotaleaceae	*Syntrophotalea*	*Syntrophotalea carbinolica*
Desulfobacterota	Syntrophaceae	*Syntrophus*	*Syntrophus aciditrophicus*
Desulfobacterota	Syntrophaceae	*Syntrophus*	*Syntrophus buswellii*
Desulfobacterota	Syntrophaceae	*Syntrophus*	*Syntrophus gentianae*
Fusobacteriota	Fusobacteriaceae	*Cetobacterium*	*Cetobacterium somerae*
Fusobacteriota	Fusobacteriaceae	*Fusobacterium*	*Fusobacterium gastrosuis*
Fusobacteriota	Fusobacteriaceae	*Fusobacterium*	*Fusobacterium mortiferum*
Fusobacteriota	Fusobacteriaceae	*Fusobacterium*	*Fusobacterium necrophorum*
Fusobacteriota	Fusobacteriaceae	*Fusobacterium*	*Fusobacterium nucleatum*
Fusobacteriota	Fusobacteriaceae	*Fusobacterium*	*Fusobacterium periodonticum*
Fusobacteriota	Fusobacteriaceae	*Fusobacterium*	*Fusobacterium varium*
Fusobacteriota	Fusobacteriaceae	*Ilyobacter*	*Ilyobacter delafieldii*

Fusobacteriota	Leptotrichiaceae	*Sebaldella*	*Sebaldella termitidis*
Gemmatimonadetes	Longimicrobiaceae	*Longimicrobium*	*Longimicrobium terrae*
Gemmatimonadota	Gemmatimonadaceae	*Gemmatimonas*	*Gemmatimonas phototrophica*
Myxococcota	Myxococcaceae	*Corallococcus*	*Corallococcus coralloides*
Myxococcota	Myxococcaceae	*Corallococcus*	*Corallococcus exiguus*
Myxococcota	Myxococcaceae	*Myxococcus*	*Myxococcus fulvus*
Myxococcota	Myxococcaceae	*Vitiosangium*	*Vitiosangium cumulatum*
Nitrospirota	Thermodesulfovibrionaceae	*Thermodesulfovibrio*	*Thermodesulfovibrio aggregans*
Planctomycetota	Isosphaeraceae	*Aquisphaera*	*Aquisphaera giovannonii*
Planctomycetota	Pirellulaceae	*Aureliella*	*Aureliella helgolandensis*
Planctomycetota	Pirellulaceae	*Blastopirellula*	*Blastopirellula marina*
Planctomycetota	Lacipirellulaceae	*Botrimarina*	*Botrimarina hoheduenensis*
Planctomycetota	Lacipirellulaceae	*Bythopirellula*	*Bythopirellula goksoyri*
Planctomycetota	Planctomycetaceae	*Crateriforma*	*Crateriforma conspicua*
Planctomycetota	Gemmataceae	*Gemmata*	*Gemmata massiliana*
Planctomycetota	Gemmataceae	*Gemmata*	*Gemmata obscuriglobus*
Planctomycetota	Isosphaeraceae	*Paludisphaera*	*Paludisphaera borealis*
Planctomycetota	Lacipirellulaceae	*Pirellulimonas*	*Pirellulimonas nuda*
Planctomycetota	Lacipirellulaceae	*Posidoniimonas*	*Posidoniimonas corsicana*
Planctomycetota	Planctomycetaceae	*Rubinisphaera*	*Rubinisphaera brasiliensis*
Planctomycetota	Planctomycetaceae	*Rubinisphaera*	*Rubinisphaera italica*
Planctomycetota	Isosphaeraceae	*Singulisphaera*	*Singulisphaera acidiphila*
Planctomycetota	Isosphaeraceae	*Singulisphaera*	*Singulisphaera rosea*
Planctomycetota	Planctomycetaceae	*Symmachiella*	*Symmachiella macrocystis*
Planctomycetota	Gemmataceae	*Telmatocola*	*Telmatocola sphagniphila*
Planctomycetota	Thermoguttaceae	*Thermogutta*	*Thermogutta hypogea*
Planctomycetota	Thermoguttaceae	*Thermogutta*	*Thermogutta terrifontis*
Planctomycetota	Thermoguttaceae	*Thermostilla*	*Thermostilla marina*

Planctomycetota	Isosphaeraceae	*Tundrisphaera*	*Tundrisphaera lichenicola*
Planctomycetota	Gemmataceae	*Urbifossiella*	*Urbifossiella limnaea*
Pseudomonadota	Acetobacteraceae	*Acetobacter*	*Acetobacter aceti*
Pseudomonadota	Acetobacteraceae	*Acetobacter*	*Acetobacter cerevisiae*
Pseudomonadota	Acetobacteraceae	*Acetobacter*	*Acetobacter cibinongensis*
Pseudomonadota	Acetobacteraceae	*Acetobacter*	*Acetobacter estunensis*
Pseudomonadota	Acetobacteraceae	*Acetobacter*	*Acetobacter fabarum*
Pseudomonadota	Acetobacteraceae	*Acetobacter*	*Acetobacter ghanensis*
Pseudomonadota	Acetobacteraceae	*Acetobacter*	*Acetobacter indonesiensis*
Pseudomonadota	Acetobacteraceae	*Acetobacter*	*Acetobacter lambici*
Pseudomonadota	Acetobacteraceae	*Acetobacter*	*Acetobacter lovaniensis*
Pseudomonadota	Acetobacteraceae	*Acetobacter*	*Acetobacter okinawensis*
Pseudomonadota	Acetobacteraceae	*Acetobacter*	*Acetobacter orientalis*
Pseudomonadota	Acetobacteraceae	*Acetobacter*	*Acetobacter orleanensis*
Pseudomonadota	Acetobacteraceae	*Acetobacter*	*Acetobacter papayae*
Pseudomonadota	Acetobacteraceae	*Acetobacter*	*Acetobacter peroxydans*
Pseudomonadota	Acetobacteraceae	*Acetobacter*	*Acetobacter persici*
Pseudomonadota	Acetobacteraceae	*Acetobacter*	*Acetobacter pomorum*
Pseudomonadota	Acetobacteraceae	*Acetobacter*	*Acetobacter sicerae*
Pseudomonadota	Acetobacteraceae	*Acetobacter*	*Acetobacter syzygii*
Pseudomonadota	Acetobacteraceae	*Acetobacter*	*Acetobacter thailandicus*
Pseudomonadota	Acetobacteraceae	*Acetobacter*	*Acetobacter tropicalis*
Pseudomonadota	Burkholderiaceae	*Achromobacter*	*Achromobacter aegrifaciens*
Pseudomonadota	Burkholderiaceae	*Achromobacter*	*Achromobacter deleyi*
Pseudomonadota	Burkholderiaceae	*Achromobacter*	*Achromobacter denitrificans*
Pseudomonadota	Burkholderiaceae	*Achromobacter*	*Achromobacter insuavis*
Pseudomonadota	Burkholderiaceae	*Achromobacter*	*Achromobacter kerstersii*
Pseudomonadota	Burkholderiaceae	*Achromobacter*	*Achromobacter marplatensis*

Pseudomonadota	Burkholderiaceae	*Achromobacter*	*Achromobacter mucicolens*
Pseudomonadota	Burkholderiaceae	*Achromobacter*	*Achromobacter pestifer*
Pseudomonadota	Burkholderiaceae	*Achromobacter*	*Achromobacter pulmonis*
Pseudomonadota	Acidiferrobacteraceae	*Acidiferrobacter*	*Acidiferrobacter thiooxydans*
Pseudomonadota	Acetobacteraceae	*Acidiphilium*	*Acidiphilium multivorum*
Pseudomonadota	Acetobacteraceae	*Acidisphaera*	*Acidisphaera rubrifaciens*
Pseudomonadota	Comamonadaceae	*Acidovorax*	*Acidovorax caeni*
Pseudomonadota	Comamonadaceae	*Acidovorax*	*Acidovorax citrulli*
Pseudomonadota	Comamonadaceae	*Acidovorax*	*Acidovorax temperans*
Pseudomonadota	Comamonadaceae	*Acidovorax*	*Acidovorax wautersii*
Pseudomonadota	Moraxellaceae	*Acinetobacter*	*Acinetobacter albensis*
Pseudomonadota	Moraxellaceae	*Acinetobacter*	*Acinetobacter antiviralis*
Pseudomonadota	Moraxellaceae	*Acinetobacter*	*Acinetobacter apis*
Pseudomonadota	Moraxellaceae	*Acinetobacter*	*Acinetobacter baumannii*
Pseudomonadota	Moraxellaceae	*Acinetobacter*	*Acinetobacter baylyi*
Pseudomonadota	Moraxellaceae	*Acinetobacter*	*Acinetobacter calcoaceticus*
Pseudomonadota	Moraxellaceae	*Acinetobacter*	*Acinetobacter chinensis*
Pseudomonadota	Moraxellaceae	*Acinetobacter*	*Acinetobacter colistiniresistens*
Pseudomonadota	Moraxellaceae	*Acinetobacter*	*Acinetobacter courvalinii*
Pseudomonadota	Moraxellaceae	*Acinetobacter*	*Acinetobacter defluvii*
Pseudomonadota	Moraxellaceae	*Acinetobacter*	*Acinetobacter dispersus*
Pseudomonadota	Moraxellaceae	*Acinetobacter*	*Acinetobacter equi*
Pseudomonadota	Moraxellaceae	*Acinetobacter*	*Acinetobacter gerneri*
Pseudomonadota	Moraxellaceae	*Acinetobacter*	*Acinetobacter haemolyticus*
Pseudomonadota	Moraxellaceae	*Acinetobacter*	*Acinetobacter halotolerans*
Pseudomonadota	Moraxellaceae	*Acinetobacter*	*Acinetobacter indicus*
Pseudomonadota	Moraxellaceae	*Acinetobacter*	*Acinetobacter johnsonii*
Pseudomonadota	Moraxellaceae	*Acinetobacter*	*Acinetobacter lactucae*

Pseudomonadota	Moraxellaceae	*Acinetobacter*	*Acinetobacter lwoffii*
Pseudomonadota	Moraxellaceae	*Acinetobacter*	*Acinetobacter modestus*
Pseudomonadota	Moraxellaceae	*Acinetobacter*	*Acinetobacter nosocomialis*
Pseudomonadota	Moraxellaceae	*Acinetobacter*	*Acinetobacter oleivorans*
Pseudomonadota	Moraxellaceae	*Acinetobacter*	*Acinetobacter piscicola*
Pseudomonadota	Moraxellaceae	*Acinetobacter*	*Acinetobacter pittii*
Pseudomonadota	Moraxellaceae	*Acinetobacter*	*Acinetobacter populi*
Pseudomonadota	Moraxellaceae	*Acinetobacter*	*Acinetobacter pragensis*
Pseudomonadota	Moraxellaceae	*Acinetobacter*	*Acinetobacter proteolyticus*
Pseudomonadota	Moraxellaceae	*Acinetobacter*	*Acinetobacter radioresistens*
Pseudomonadota	Moraxellaceae	*Acinetobacter*	*Acinetobacter seifertii*
Pseudomonadota	Moraxellaceae	*Acinetobacter*	*Acinetobacter septicus*
Pseudomonadota	Moraxellaceae	*Acinetobacter*	*Acinetobacter soli*
Pseudomonadota	Moraxellaceae	*Acinetobacter*	*Acinetobacter tandoii*
Pseudomonadota	Moraxellaceae	*Acinetobacter*	*Acinetobacter tjernbergiae*
Pseudomonadota	Moraxellaceae	*Acinetobacter*	*Acinetobacter variabilis*
Pseudomonadota	Moraxellaceae	*Acinetobacter*	*Acinetobacter venetianus*
Pseudomonadota	Moraxellaceae	*Acinetobacter*	*Acinetobacter vivianii*
Pseudomonadota	Pasteurellaceae	*Actinobacillus*	*Actinobacillus anseriformium*
Pseudomonadota	Pasteurellaceae	*Actinobacillus*	*Actinobacillus arthritidis*
Pseudomonadota	Pasteurellaceae	*Actinobacillus*	*Actinobacillus capsulatus*
Pseudomonadota	Pasteurellaceae	*Actinobacillus*	*Actinobacillus delphinicola*
Pseudomonadota	Pasteurellaceae	*Actinobacillus*	*Actinobacillus equuli*
Pseudomonadota	Pasteurellaceae	*Actinobacillus*	*Actinobacillus hominis*
Pseudomonadota	Pasteurellaceae	*Actinobacillus*	*Actinobacillus indolicus*
Pseudomonadota	Pasteurellaceae	*Actinobacillus*	*Actinobacillus lignieresii*
Pseudomonadota	Pasteurellaceae	*Actinobacillus*	*Actinobacillus minor*
Pseudomonadota	Pasteurellaceae	*Actinobacillus*	*Actinobacillus pleuropneumoniae*

Pseudomonadota	Pasteurellaceae	*Actinobacillus*	*Actinobacillus porcinus*
Pseudomonadota	Pasteurellaceae	*Actinobacillus*	*Actinobacillus rossii*
Pseudomonadota	Pasteurellaceae	*Actinobacillus*	*Actinobacillus scotiae*
Pseudomonadota	Pasteurellaceae	*Actinobacillus*	*Actinobacillus seminis*
Pseudomonadota	Pasteurellaceae	*Actinobacillus*	*Actinobacillus succinogenes*
Pseudomonadota	Pasteurellaceae	*Actinobacillus*	*Actinobacillus suis*
Pseudomonadota	Pasteurellaceae	*Actinobacillus*	*Actinobacillus ureae*
Pseudomonadota	Burkholderiaceae	*Advenella*	*Advenella kashmirensis*
Pseudomonadota	Burkholderiaceae	*Advenella*	*Advenella mimigardefordensis*
Pseudomonadota	Aeromonadaceae	*Aeromonas*	*Aeromonas cavernicola*
Pseudomonadota	Aeromonadaceae	*Aeromonas*	*Aeromonas caviae*
Pseudomonadota	Aeromonadaceae	*Aeromonas*	*Aeromonas dhakensis*
Pseudomonadota	Aeromonadaceae	*Aeromonas*	*Aeromonas diversa*
Pseudomonadota	Aeromonadaceae	*Aeromonas*	*Aeromonas encheleia*
Pseudomonadota	Aeromonadaceae	*Aeromonas*	*Aeromonas enteropelogenes*
Pseudomonadota	Aeromonadaceae	*Aeromonas*	*Aeromonas hydrophila*
Pseudomonadota	Aeromonadaceae	*Aeromonas*	*Aeromonas jandaei*
Pseudomonadota	Aeromonadaceae	*Aeromonas*	*Aeromonas media*
Pseudomonadota	Aeromonadaceae	*Aeromonas*	*Aeromonas molluscorum*
Pseudomonadota	Aeromonadaceae	*Aeromonas*	*Aeromonas rivipollensis*
Pseudomonadota	Aeromonadaceae	*Aeromonas*	*Aeromonas rivuli*
Pseudomonadota	Aeromonadaceae	*Aeromonas*	*Aeromonas salmonicida*
Pseudomonadota	Aeromonadaceae	*Aeromonas*	*Aeromonas sanarellii*
Pseudomonadota	Aeromonadaceae	*Aeromonas*	*Aeromonas schubertii*
Pseudomonadota	Aeromonadaceae	*Aeromonas*	*Aeromonas simiae*
Pseudomonadota	Aeromonadaceae	*Aeromonas*	*Aeromonas taiwanensis*
Pseudomonadota	Aeromonadaceae	*Aeromonas*	*Aeromonas tecta*
Pseudomonadota	Pectobacteriaceae	*Affinibrenneria*	*Affinibrenneria salicis*

Pseudomonadota	Xanthobacteraceae	*Afipia*	*Afipia clevelandensis*
Pseudomonadota	Xanthobacteraceae	*Afipia*	*Afipia felis*
Pseudomonadota	Psychromonadaceae	*Agarivorans*	*Agarivorans aestuarii*
Pseudomonadota	Pasteurellaceae	*Aggregatibacter*	*Aggregatibacter actinomycetemcomitans*
Pseudomonadota	Pasteurellaceae	*Aggregatibacter*	*Aggregatibacter aphrophilus*
Pseudomonadota	Pasteurellaceae	*Aggregatibacter*	*Aggregatibacter segnis*
Pseudomonadota	Rhizobiaceae	*Agrobacterium*	*Agrobacterium salinitolerans*
Pseudomonadota	Rhizobiaceae	*Ahrensia*	*Ahrensia kielensis*
Pseudomonadota	Rhizobiaceae	*Ahrensia*	*Ahrensia marina*
Pseudomonadota	Rhodobacteraceae	*Albirhodobacter*	*Albirhodobacter confluentis*
Pseudomonadota	Burkholderiaceae	*Alcaligenes*	*Alcaligenes aquatilis*
Pseudomonadota	Burkholderiaceae	*Alcaligenes*	*Alcaligenes endophyticus*
Pseudomonadota	Burkholderiaceae	*Alcaligenes*	*Alcaligenes faecalis*
Pseudomonadota	Alcanivoracaceae	*Alcanivorax*	*Alcanivorax jadensis*
Pseudomonadota	Alcanivoracaceae	*Alcanivorax*	*Alcanivorax marinus*
Pseudomonadota	Alteromonadaceae	*Algicola*	*Algicola bacteriolytica*
Pseudomonadota	Psychromonadaceae	*Alginatibacterium*	*Alginatibacterium sediminis*
Pseudomonadota	Alteromonadaceae	*Aliidiomarina*	*Aliidiomarina maris*
Pseudomonadota	Alteromonadaceae	*Aliidiomarina*	*Aliidiomarina soli*
Pseudomonadota	Vibrionaceae	*Aliivibrio*	*Aliivibrio finisterrensis*
Pseudomonadota	Vibrionaceae	*Aliivibrio*	*Aliivibrio fischeri*
Pseudomonadota	Vibrionaceae	*Aliivibrio*	*Aliivibrio logei*
Pseudomonadota	Vibrionaceae	*Aliivibrio*	*Aliivibrio salmonicida*
Pseudomonadota	Vibrionaceae	*Aliivibrio*	*Aliivibrio wodanis*
Pseudomonadota	Alteromonadaceae	*Alishewanella*	*Alishewanella alkalitolerans*
Pseudomonadota	Alteromonadaceae	*Alishewanella*	*Alishewanella jeotgali*
Pseudomonadota	Alteromonadaceae	*Alkalimonas*	*Alkalimonas amylolytica*
Pseudomonadota	Alteromonadaceae	*Alkalimonas*	*Alkalimonas collagenimarina*

Pseudomonadota	Alteromonadaceae	*Alkalimonas*	*Alkalimonas delamerensis*
Pseudomonadota	Alteromonadaceae	*Alteromonas*	*Alteromonas mediterranea*
Pseudomonadota	Rhodobacteraceae	*Amaricoccus*	*Amaricoccus kaplicensis*
Pseudomonadota	Rhodobacteraceae	*Amaricoccus*	*Amaricoccus macauensis*
Pseudomonadota	Rhodobacteraceae	*Amaricoccus*	*Amaricoccus tamworthensis*
Pseudomonadota	Rhodobacteraceae	*Amaricoccus*	*Amaricoccus veronensis*
Pseudomonadota	Acetobacteraceae	*Ameyamaea*	*Ameyamaea chiangmaiensis*
Pseudomonadota	Rhizobiaceae	*Aminobacter*	*Aminobacter aminovorans*
Pseudomonadota	Rhizobiaceae	*Aminobacter*	*Aminobacter anthyllidis*
Pseudomonadota	Amorphaceae	*Amorphus*	*Amorphus coralli*
Pseudomonadota	Nitrincolaceae	*Amphritea*	*Amphritea spongicola*
Pseudomonadota	Succinivibrionaceae	*Anaerobiospirillum*	*Anaerobiospirillum thomasii*
Pseudomonadota	Anaplasmataceae	*Anaplasma*	*Anaplasma phagocytophilum*
Pseudomonadota	Xanthobacteraceae	*Ancylobacter*	*Ancylobacter rudongensis*
Pseudomonadota	Burkholderiaceae	*Aquabacterium*	*Aquabacterium commune*
Pseudomonadota	Phyllobacteriaceae	*Aquamicrobium*	*Aquamicrobium segne*
Pseudomonadota	Phyllobacteriaceae	*Aquamicrobium*	*Aquamicrobium terrae*
Pseudomonadota	Coxiellaceae	*Aquicella*	*Aquicella lusitana*
Pseudomonadota	Burkholderiaceae	*Aquincola*	*Aquincola amnicola*
Pseudomonadota	Aquisalimonadaceae	*Aquisalimonas*	*Aquisalimonas halophila*
Pseudomonadota	Arenicellaceae	*Arenicella*	*Arenicella chitinivorans*
Pseudomonadota	Xanthomonadaceae	*Arenimonas*	*Arenimonas aquatica*
Pseudomonadota	Xanthomonadaceae	*Arenimonas*	*Arenimonas metalli*
Pseudomonadota	Rhodocyclaceae	*Aromatoleum*	*Aromatoleum bremense*
Pseudomonadota	Rhodocyclaceae	*Aromatoleum*	*Aromatoleum pumilum*
Pseudomonadota	Alteromonadaceae	*Arsukibacterium*	*Arsukibacterium ikkense*
Pseudomonadota	Acetobacteraceae	*Asaia*	*Asaia astilbis*
Pseudomonadota	Acetobacteraceae	*Asaia*	*Asaia bogorensis*

Pseudomonadota	Acetobacteraceae	*Asaia*	*Asaia krungthepensis*
Pseudomonadota	Acetobacteraceae	*Asaia*	*Asaia lannensis*
Pseudomonadota	Acetobacteraceae	*Asaia*	*Asaia platycodi*
Pseudomonadota	Acetobacteraceae	*Asaia*	*Asaia prunellae*
Pseudomonadota	Acetobacteraceae	*Asaia*	*Asaia siamensis*
Pseudomonadota	Acetobacteraceae	*Asaia*	*Asaia spathodeae*
Pseudomonadota	Enterobacteriaceae	*Atlantibacter*	*Atlantibacter hermannii*
Pseudomonadota	Enterobacteriaceae	*Atlantibacter*	*Atlantibacter subterranea*
Pseudomonadota	Pseudohongiellaceae	*Atopomonas*	*Atopomonas hussainii*
Pseudomonadota	Rhizobiaceae	*Aurantimonas*	*Aurantimonas endophytica*
Pseudomonadota	Pasteurellaceae	*Avibacterium*	*Avibacterium avium*
Pseudomonadota	Pasteurellaceae	*Avibacterium*	*Avibacterium gallinarum*
Pseudomonadota	Pasteurellaceae	*Avibacterium*	*Avibacterium paragallinarum*
Pseudomonadota	Pasteurellaceae	*Avibacterium*	*Avibacterium volantium*
Pseudomonadota	Rhodocyclaceae	*Azoarcus*	*Azoarcus anaerobius*
Pseudomonadota	Rhodocyclaceae	*Azoarcus*	*Azoarcus evansii*
Pseudomonadota	Rhodocyclaceae	*Azoarcus*	*Azoarcus indigens*
Pseudomonadota	Rhodocyclaceae	*Azoarcus*	*Azoarcus toluclasticus*
Pseudomonadota	Pseudomonadaceae	*Azomonas*	*Azomonas agilis*
Pseudomonadota	Xanthobacteraceae	*Azorhizobium*	*Azorhizobium caulinodans*
Pseudomonadota	Xanthobacteraceae	*Azorhizobium*	*Azorhizobium doebereinerae*
Pseudomonadota	Xanthobacteraceae	*Azorhizobium*	*Azorhizobium oxalatiphilum*
Pseudomonadota	Pseudohongiellaceae	*Azorhizophilus*	*Azorhizophilus paspali*
Pseudomonadota	Azospirillaceae	*Azospirillum*	*Azospirillum thermophilum*
Pseudomonadota	Pseudomonadaceae	*Azotobacter*	*Azotobacter nigricans*
Pseudomonadota	Rhizobiaceae	*Bartonella*	*Bartonella ancashensis*
Pseudomonadota	Rhizobiaceae	*Bartonella*	*Bartonella apis*
Pseudomonadota	Rhizobiaceae	*Bartonella*	*Bartonella australis*

Pseudomonadota	Rhizobiaceae	*Bartonella*	*Bartonella bacilliformis*
Pseudomonadota	Rhizobiaceae	*Bartonella*	*Bartonella birtlesii*
Pseudomonadota	Rhizobiaceae	*Bartonella*	*Bartonella capreoli*
Pseudomonadota	Rhizobiaceae	*Bartonella*	*Bartonella chomelii*
Pseudomonadota	Rhizobiaceae	*Bartonella*	*Bartonella coopersplainsensis*
Pseudomonadota	Rhizobiaceae	*Bartonella*	*Bartonella elizabethae*
Pseudomonadota	Rhizobiaceae	*Bartonella*	*Bartonella fuyuanensis*
Pseudomonadota	Rhizobiaceae	*Bartonella*	*Bartonella henselae*
Pseudomonadota	Rhizobiaceae	*Bartonella*	*Bartonella koehlerae*
Pseudomonadota	Rhizobiaceae	*Bartonella*	*Bartonella pachyuromydis*
Pseudomonadota	Rhizobiaceae	*Bartonella*	*Bartonella phoceensis*
Pseudomonadota	Rhizobiaceae	*Bartonella*	*Bartonella queenslandensis*
Pseudomonadota	Rhizobiaceae	*Bartonella*	*Bartonella quintana*
Pseudomonadota	Rhizobiaceae	*Bartonella*	*Bartonella rattaustraliani*
Pseudomonadota	Rhizobiaceae	*Bartonella*	*Bartonella rattimassiliensis*
Pseudomonadota	Rhizobiaceae	*Bartonella*	*Bartonella rochalimae*
Pseudomonadota	Rhizobiaceae	*Bartonella*	*Bartonella taylorii*
Pseudomonadota	Rhizobiaceae	*Bartonella*	*Bartonella tribocorum*
Pseudomonadota	Rhizobiaceae	*Bartonella*	*Bartonella vinsonii*
Pseudomonadota	Pasteurellaceae	*Basfia*	*Basfia succiniciproducens*
Pseudomonadota	Kaistiaceae	*Bauldia*	*Bauldia litoralis*
Pseudomonadota	Beijerinckiaceae	*Beijerinckia*	*Beijerinckia doebereinerae*
Pseudomonadota	Beijerinckiaceae	*Beijerinckia*	*Beijerinckia fluminensis*
Pseudomonadota	Beijerinckiaceae	*Beijerinckia*	*Beijerinckia indica*
Pseudomonadota	Beijerinckiaceae	*Beijerinckia*	*Beijerinckia mobilis*
Pseudomonadota	Neisseriaceae	*Bergeriella*	*Bergeriella denitrificans*
Pseudomonadota	Enterobacteriaceae	*Biostraticola*	*Biostraticola tofi*
Pseudomonadota	Pasteurellaceae	*Bisgaardia*	*Bisgaardia hudsonensis*

Pseudomonadota	Burkholderiaceae	*Bordetella*	*Bordetella avium*
Pseudomonadota	Burkholderiaceae	*Bordetella*	*Bordetella trematum*
Pseudomonadota	Beijerinckiaceae	*Bosea*	*Bosea massiliensis*
Pseudomonadota	Beijerinckiaceae	*Bosea*	*Bosea minatitlanensis*
Pseudomonadota	Beijerinckiaceae	*Bosea*	*Bosea robiniae*
Pseudomonadota	Beijerinckiaceae	*Bosea*	*Bosea thiooxidans*
Pseudomonadota	Beijerinckiaceae	*Bosea*	*Bosea vestrisii*
Pseudomonadota	Alteromonadaceae	*Bowmanella*	*Bowmanella denitrificans*
Pseudomonadota	Alteromonadaceae	*Bowmanella*	*Bowmanella pacifica*
Pseudomonadota	Burkholderiaceae	*Brackiella*	*Brackiella oedipodis*
Pseudomonadota	Xanthobacteraceae	*Bradyrhizobium*	*Bradyrhizobium americanum*
Pseudomonadota	Xanthobacteraceae	*Bradyrhizobium*	*Bradyrhizobium cajani*
Pseudomonadota	Xanthobacteraceae	*Bradyrhizobium*	*Bradyrhizobium centrosematis*
Pseudomonadota	Xanthobacteraceae	*Bradyrhizobium*	*Bradyrhizobium diazoefficiens*
Pseudomonadota	Xanthobacteraceae	*Bradyrhizobium*	*Bradyrhizobium elkanii*
Pseudomonadota	Xanthobacteraceae	*Bradyrhizobium*	*Bradyrhizobium iriomotense*
Pseudomonadota	Xanthobacteraceae	*Bradyrhizobium*	*Bradyrhizobium japonicum*
Pseudomonadota	Xanthobacteraceae	*Bradyrhizobium*	*Bradyrhizobium jicamae*
Pseudomonadota	Xanthobacteraceae	*Bradyrhizobium*	*Bradyrhizobium liaoningense*
Pseudomonadota	Xanthobacteraceae	*Bradyrhizobium*	*Bradyrhizobium lupini*
Pseudomonadota	Xanthobacteraceae	*Bradyrhizobium*	*Bradyrhizobium oligotrophicum*
Pseudomonadota	Xanthobacteraceae	*Bradyrhizobium*	*Bradyrhizobium pachyrhizi*
Pseudomonadota	Xanthobacteraceae	*Bradyrhizobium*	*Bradyrhizobium rifense*
Pseudomonadota	Xanthobacteraceae	*Bradyrhizobium*	*Bradyrhizobium valentinum*
Pseudomonadota	Xanthobacteraceae	*Bradyrhizobium*	*Bradyrhizobium viridifuturi;Bradyrhizobium*
Pseudomonadota	Enterobacteriaceae	*Brenneria*	*Brenneria alni*
Pseudomonadota	Enterobacteriaceae	*Brenneria*	*Brenneria corticis*
Pseudomonadota	Enterobacteriaceae	*Brenneria*	*Brenneria goodwinii*

Pseudomonadota	Enterobacteriaceae	*Brenneria*	*Brenneria nigrifluens*
Pseudomonadota	Enterobacteriaceae	*Brenneria*	*Brenneria populi*
Pseudomonadota	Enterobacteriaceae	*Brenneria*	*Brenneria roseae*
Pseudomonadota	Enterobacteriaceae	*Brenneria*	*Brenneria rubrifaciens*
Pseudomonadota	Enterobacteriaceae	*Brenneria*	*Brenneria salicis*
Pseudomonadota	Caulobacteraceae	*Brevundimonas*	*Brevundimonas nasdae*
Pseudomonadota	Brucellaceae	*Brucella*	*Brucella anthropi*
Pseudomonadota	Brucellaceae	*Brucella*	*Brucella ciceri*
Pseudomonadota	Brucellaceae	*Brucella*	*Brucella endophytica*
Pseudomonadota	Brucellaceae	*Brucella*	*Brucella gallinifaecis*
Pseudomonadota	Brucellaceae	*Brucella*	*Brucella haematophila*
Pseudomonadota	Brucellaceae	*Brucella*	*Brucella inopinata*
Pseudomonadota	Brucellaceae	*Brucella*	*Brucella intermedia*
Pseudomonadota	Brucellaceae	*Brucella*	*Brucella lupini*
Pseudomonadota	Brucellaceae	*Brucella*	*Brucella microti*
Pseudomonadota	Brucellaceae	*Brucella*	*Brucella oryzae*
Pseudomonadota	Brucellaceae	*Brucella*	*Brucella papionis*
Pseudomonadota	Brucellaceae	*Brucella*	*Brucella pituitosa*
Pseudomonadota	Brucellaceae	*Brucella*	*Brucella pseudintermedia*
Pseudomonadota	Enterobacteriaceae	*Budvicia*	*Budvicia aquatica*
Pseudomonadota	Enterobacteriaceae	*Budvicia*	*Budvicia diplopodorum*
Pseudomonadota	Burkholderiaceae	*Burkholderia*	*Burkholderia pseudomultivorans*
Pseudomonadota	Burkholderiaceae	*Burkholderia*	*Burkholderia rinojensis*
Pseudomonadota	Burkholderiaceae	*Burkholderia*	*Burkholderia stabilis*
Pseudomonadota	Burkholderiaceae	*Burkholderia*	*Burkholderia thailandensis*
Pseudomonadota	Enterobacteriaceae	*Buttiauxella*	*Buttiauxella agrestis*
Pseudomonadota	Enterobacteriaceae	*Buttiauxella*	*Buttiauxella brennerae*
Pseudomonadota	Enterobacteriaceae	*Buttiauxella*	*Buttiauxella ferragutiae*

Pseudomonadota	Enterobacteriaceae	*Buttiauxella*	*Buttiauxella gaviniae*
Pseudomonadota	Enterobacteriaceae	*Buttiauxella*	*Buttiauxella izardii*
Pseudomonadota	Enterobacteriaceae	*Buttiauxella*	*Buttiauxella noackiae*
Pseudomonadota	Enterobacteriaceae	*Buttiauxella*	*Buttiauxella warmboldiae*
Pseudomonadota	Tepidamorphaceae	*Butyratibacter*	*Butyratibacter algicola*
Pseudomonadota	Burkholderiaceae	*Caballeronia*	*Caballeronia arationis*
Pseudomonadota	Burkholderiaceae	*Caballeronia*	*Caballeronia arvi*
Pseudomonadota	Burkholderiaceae	*Caballeronia*	*Caballeronia calidae*
Pseudomonadota	Burkholderiaceae	*Caballeronia*	*Caballeronia concitans*
Pseudomonadota	Burkholderiaceae	*Caballeronia*	*Caballeronia cordobensis*
Pseudomonadota	Burkholderiaceae	*Caballeronia*	*Caballeronia fortuita*
Pseudomonadota	Burkholderiaceae	*Caballeronia*	*Caballeronia grimmiae*
Pseudomonadota	Burkholderiaceae	*Caballeronia*	*Caballeronia jiangsuensis*
Pseudomonadota	Burkholderiaceae	*Caballeronia*	*Caballeronia megalochromosomata*
Pseudomonadota	Burkholderiaceae	*Caballeronia*	*Caballeronia pedi*
Pseudomonadota	Burkholderiaceae	*Caballeronia*	*Caballeronia ptereochthonis*
Pseudomonadota	Burkholderiaceae	*Caballeronia*	*Caballeronia temeraria*
Pseudomonadota	Burkholderiaceae	*Caballeronia*	*Caballeronia terrestris*
Pseudomonadota	Burkholderiaceae	*Caballeronia*	*Caballeronia turbans*
Pseudomonadota	Burkholderiaceae	*Caballeronia*	*Caballeronia zhejiangensis*
Pseudomonadota	Francisellaceae	*Caedibacter*	*Caedibacter taeniospiralis*
Pseudomonadota	Caedimonadaceae	*Caedimonas*	*Caedimonas varicaedens*
Pseudomonadota	Burkholderiaceae	*Caenibaculum*	*Caenibaculum baiyandianus*
Pseudomonadota	Rhodospirillaceae	*Caenispirillum*	*Caenispirillum bisanense*
Pseudomonadota	Rhodospirillaceae	*Caenispirillum*	*Caenispirillum deserti*
Pseudomonadota	Rhodospirillaceae	*Caenispirillum*	*Caenispirillum salinarum*
Pseudomonadota	Burkholderiaceae	*Caldimonas*	*Caldimonas hydrothermale*
Pseudomonadota	Pasteurellaceae	*Canicola*	*Canicola haemoglobinophilus*

Pseudomonadota	Caulobacteraceae	*Caulobacter*	*Caulobacter hibisci*
Pseudomonadota	Caulobacteraceae	*Caulobacter*	*Caulobacter mirabilis*
Pseudomonadota	Caulobacteraceae	*Caulobacter*	*Caulobacter vibrioides*
Pseudomonadota	Moraxellaceae	*Cavicella*	*Cavicella subterranea*
Pseudomonadota	Pasteurellaceae	*Caviibacterium*	*Caviibacterium pharyngocola*
Pseudomonadota	Enterobacteriaceae	*Cedecea*	*Cedecea davisae*
Pseudomonadota	Enterobacteriaceae	*Cedecea*	*Cedecea lapagei*
Pseudomonadota	Enterobacteriaceae	*Cedecea*	*Cedecea neteri*
Pseudomonadota	Psychromonadaceae	*Celerinatantimonas*	*Celerinatantimonas diazotrophica*
Pseudomonadota	Cellvibrionaceae	*Cellvibrio*	*Cellvibrio fontiphilus*
Pseudomonadota	Enterobacteriaceae	*Chania*	*Chania multitudinisentens*
Pseudomonadota	Beijerinckiaceae	*Chelatococcus*	*Chelatococcus asaccharovorans*
Pseudomonadota	Beijerinckiaceae	*Chelatococcus*	*Chelatococcus caeni*
Pseudomonadota	Beijerinckiaceae	*Chelatococcus*	*Chelatococcus composti*
Pseudomonadota	Beijerinckiaceae	*Chelatococcus*	*Chelatococcus daeguensis*
Pseudomonadota	Beijerinckiaceae	*Chelatococcus*	*Chelatococcus reniformis*
Pseudomonadota	Methylocystaceae	*Chenggangzhangella*	*Chenggangzhangella methanolivorans*
Pseudomonadota	Chitinibacteraceae	*Chitinilyticum*	*Chitinilyticum litopenaei*
Pseudomonadota	Chromobacteriaceae	*Chromobacterium*	*Chromobacterium alkanivorans*
Pseudomonadota	Chromobacteriaceae	*Chromobacterium*	*Chromobacterium aquaticum*
Pseudomonadota	Chromobacteriaceae	*Chromobacterium*	*Chromobacterium haemolyticum*
Pseudomonadota	Pleomorphomonadaceae	*Chthonobacter*	*Chthonobacter albigriseus*
Pseudomonadota	Xanthomonadaceae	*Chujaibacter*	*Chujaibacter soli*
Pseudomonadota	Rhizobiaceae	*Ciceribacter*	*Ciceribacter thiooxidans*
Pseudomonadota	Enterobacteriaceae	*Citrobacter*	*Citrobacter amalonaticus*
Pseudomonadota	Enterobacteriaceae	*Citrobacter*	*Citrobacter braakii*
Pseudomonadota	Enterobacteriaceae	*Citrobacter*	*Citrobacter cronae*
Pseudomonadota	Enterobacteriaceae	*Citrobacter*	*Citrobacter europaeus*

Pseudomonadota	Enterobacteriaceae	*Citrobacter*	*Citrobacter farmeri*
Pseudomonadota	Enterobacteriaceae	*Citrobacter*	*Citrobacter freundii*
Pseudomonadota	Enterobacteriaceae	*Citrobacter*	*Citrobacter gillenii*
Pseudomonadota	Enterobacteriaceae	*Citrobacter*	*Citrobacter koseri*
Pseudomonadota	Enterobacteriaceae	*Citrobacter*	*Citrobacter murliniae*
Pseudomonadota	Enterobacteriaceae	*Citrobacter*	*Citrobacter rodentium*
Pseudomonadota	Enterobacteriaceae	*Citrobacter*	*Citrobacter sedlakii*
Pseudomonadota	Enterobacteriaceae	*Citrobacter*	*Citrobacter werkmanii*
Pseudomonadota	Enterobacteriaceae	*Citrobacter*	*Citrobacter youngae*
Pseudomonadota	Halomonadaceae	*Cobetia*	*Cobetia marina*
Pseudomonadota	Thiotrichaceae	*Cocleimonas*	*Cocleimonas flava*
Pseudomonadota	Thorselliaceae	*Coetzeea*	*Coetzeea brasiliensis*
Pseudomonadota	Colwelliaceae	*Cognaticolwellia*	*Cognaticolwellia beringensis*
Pseudomonadota	Alteromonadaceae	*Colwellia*	*Colwellia asteriadis*
Pseudomonadota	Alteromonadaceae	*Colwellia*	*Colwellia meonggei*
Pseudomonadota	Alteromonadaceae	*Colwellia*	*Colwellia piezophila*
Pseudomonadota	Alteromonadaceae	*Colwellia*	*Colwellia sediminilitoris*
Pseudomonadota	Burkholderiaceae	*Comamonas*	*Comamonas aquatica*
Pseudomonadota	Burkholderiaceae	*Comamonas*	*Comamonas badia*
Pseudomonadota	Burkholderiaceae	*Comamonas*	*Comamonas phosphati*
Pseudomonadota	Burkholderiaceae	*Comamonas*	*Comamonas terrae*
Pseudomonadota	Burkholderiaceae	*Comamonas*	*Comamonas terrigena*
Pseudomonadota	Burkholderiaceae	*Comamonas*	*Comamonas testosteroni*
Pseudomonadota	Acetobacteraceae	*Commensalibacter*	*Commensalibacter intestini*
Pseudomonadota	Pasteurellaceae	*Conservatibacter*	*Conservatibacter flavescens*
Pseudomonadota	Vibrionaceae	*Corallibacterium*	*Corallibacterium pacifica*
Pseudomonadota	Neiellaceae	*Corallincola*	*Corallincola luteus*
Pseudomonadota	Neiellaceae	*Corallincola*	*Corallincola platygyrae*

Pseudomonadota	Neiellaceae	*Corallincola*	*Corallincola spongiicola*
Pseudomonadota	Comamonadaceae	*Corticibacter*	*Corticibacter populi*
Pseudomonadota	Morganellaceae	*Cosenzaea*	*Cosenzaea myxofaciens*
Pseudomonadota	Coxiellaceae	*Coxiella*	*Coxiella burnetii*
Pseudomonadota	Enterobacteriaceae	*Cronobacter*	*Cronobacter condimenti*
Pseudomonadota	Enterobacteriaceae	*Cronobacter*	*Cronobacter dublinensis*
Pseudomonadota	Enterobacteriaceae	*Cronobacter*	*Cronobacter malonaticus*
Pseudomonadota	Enterobacteriaceae	*Cronobacter*	*Cronobacter muytjensii*
Pseudomonadota	Enterobacteriaceae	*Cronobacter*	*Cronobacter sakazakii*
Pseudomonadota	Enterobacteriaceae	*Cronobacter*	*Cronobacter turicensis*
Pseudomonadota	Enterobacteriaceae	*Cronobacter*	*Cronobacter universalis*
Pseudomonadota	Burkholderiaceae	*Cupriavidus*	*Cupriavidus metallidurans*
Pseudomonadota	Burkholderiaceae	*Cupriavidus*	*Cupriavidus oxalaticus*
Pseudomonadota	Burkholderiaceae	*Curvibacter*	*Curvibacter fontanus*
Pseudomonadota	Cycloclasticaceae	*Cycloclasticus*	*Cycloclasticus pugetii*
Pseudomonadota	Fastidiosibacteraceae	*Cysteiniphilum*	*Cysteiniphilum litorale*
Pseudomonadota	Comamonadaceae	*Delftia*	*Delftia tsuruhatensis*
Pseudomonadota	Burkholderiaceae	*Derxia*	*Derxia gummosa*
Pseudomonadota	Devosiaceae	*Devosia*	*Devosia chinhatensis*
Pseudomonadota	Devosiaceae	*Devosia*	*Devosia enhydra*
Pseudomonadota	Devosiaceae	*Devosia*	*Devosia insulae*
Pseudomonadota	Devosiaceae	*Devosia*	*Devosia limi*
Pseudomonadota	Devosiaceae	*Devosia*	*Devosia mishustinii*
Pseudomonadota	Devosiaceae	*Devosia*	*Devosia riboflavina*
Pseudomonadota	Devosiaceae	*Devosia*	*Devosia soli*
Pseudomonadota	Devosiaceae	*Devosia*	*Devosia submarina*
Pseudomonadota	Cardiobacteriaceae	*Dichelobacter*	*Dichelobacter nodosus*
Pseudomonadota	Enterobacteriaceae	*Dickeya*	*Dickeya aquatica*

Pseudomonadota	Enterobacteriaceae	*Dickeya*	*Dickeya chrysanthemi*
Pseudomonadota	Enterobacteriaceae	*Dickeya*	*Dickeya dadantii*
Pseudomonadota	Enterobacteriaceae	*Dickeya*	*Dickeya dianthicola*
Pseudomonadota	Enterobacteriaceae	*Dickeya*	*Dickeya fangzhongdai*
Pseudomonadota	Enterobacteriaceae	*Dickeya*	*Dickeya paradisiaca*
Pseudomonadota	Enterobacteriaceae	*Dickeya*	*Dickeya zeae*
Pseudomonadota	Diplorickettsiaceae	*Diplorickettsia*	*Diplorickettsia massiliensis*
Pseudomonadota	Dissulfurirhabdaceae	*Dissulfurirhabdus*	*Dissulfurirhabdus thermomarina*
Pseudomonadota	Rhodanobacteraceae	*Dokdonella*	*Dokdonella kunshanensis*
Pseudomonadota	Rhodanobacteraceae	*Dokdonella*	*Dokdonella soli*
Pseudomonadota	Dongiaceae	*Dongia*	*Dongia mobilis*
Pseudomonadota	Dongiaceae	*Dongia*	*Dongia soli*
Pseudomonadota	Burkholderiaceae	*Duodenibacillus*	*Duodenibacillus massiliensis*
Pseudomonadota	Rhodanobacteraceae	*Dyella*	*Dyella caseinilytica*
Pseudomonadota	Rhodanobacteraceae	*Dyella*	*Dyella dinghuensis*
Pseudomonadota	Rhodanobacteraceae	*Dyella*	*Dyella jiangningensis*
Pseudomonadota	Rhodanobacteraceae	*Dyella*	*Dyella mobilis*
Pseudomonadota	Ectothiorhodospiraceae	*Ectothiorhodosinus*	*Ectothiorhodosinus mongolicus*
Pseudomonadota	Ectothiorhodospiraceae	*Ectothiorhodospira*	*Ectothiorhodospira haloalkaliphila*
Pseudomonadota	Ectothiorhodospiraceae	*Ectothiorhodospira*	*Ectothiorhodospira magna*
Pseudomonadota	Ectothiorhodospiraceae	*Ectothiorhodospira*	*Ectothiorhodospira variabilis*
Pseudomonadota	Enterobacteriaceae	*Edwardsiella*	*Edwardsiella anguillarum*
Pseudomonadota	Enterobacteriaceae	*Edwardsiella*	*Edwardsiella hoshinae*
Pseudomonadota	Enterobacteriaceae	*Edwardsiella*	*Edwardsiella ictaluri*
Pseudomonadota	Enterobacteriaceae	*Edwardsiella*	*Edwardsiella tarda*
Pseudomonadota	Anaplasmataceae	*Ehrlichia*	*Ehrlichia chaffeensis*
Pseudomonadota	Anaplasmataceae	*Ehrlichia*	*Ehrlichia rickettsia*
Pseudomonadota	Neisseriaceae	*Eikenella*	*Eikenella corrodens*

Pseudomonadota	Thioalkalispiraceae	*Endothiovibrio*	*Endothiovibrio diazotrophicus*
Pseudomonadota	Endozoicomonadaceae	*Endozoicomonas*	*Endozoicomonas ascidiicola*
Pseudomonadota	Rhizobiaceae	*Ensifer*	*Ensifer adhaerens*
Pseudomonadota	Enterobacteriaceae	*Enterobacillus*	*Enterobacillus tribolii*
Pseudomonadota	Enterobacteriaceae	*Enterobacter*	*Enterobacter asburiae*
Pseudomonadota	Enterobacteriaceae	*Enterobacter*	*Enterobacter bugandensis*
Pseudomonadota	Enterobacteriaceae	*Enterobacter*	*Enterobacter cancerogenus*
Pseudomonadota	Enterobacteriaceae	*Enterobacter*	*Enterobacter cloacae*
Pseudomonadota	Enterobacteriaceae	*Enterobacter*	*Enterobacter hormaechei*
Pseudomonadota	Enterobacteriaceae	*Enterobacter*	*Enterobacter kobei*
Pseudomonadota	Enterobacteriaceae	*Enterobacter*	*Enterobacter ludwigii*
Pseudomonadota	Enterobacteriaceae	*Enterobacter*	*Enterobacter mori*
Pseudomonadota	Enterobacteriaceae	*Enterobacter*	*Enterobacter soli*
Pseudomonadota	Enterobacteriaceae	*Enterobacter*	*Enterobacter tabaci*
Pseudomonadota	Vibrionaceae	*Enterovibrio*	*Enterovibrio nigricans*
Pseudomonadota	Vibrionaceae	*Enterovibrio*	*Enterovibrio norvegicus*
Pseudomonadota	Vibrionaceae	*Enterovibrio*	*Enterovibrio pacificus*
Pseudomonadota	Beijerinckiaceae	*Enterovirga*	*Enterovirga rhinocerotis*
Pseudomonadota	Pseudomonadaceae	*Entomomonas*	*Entomomonas moraniae*
Pseudomonadota	Enterobacteriaceae	*Erwinia*	*Erwinia amylovora*
Pseudomonadota	Enterobacteriaceae	*Erwinia*	*Erwinia aphidicola*
Pseudomonadota	Enterobacteriaceae	*Erwinia*	*Erwinia billingiae*
Pseudomonadota	Enterobacteriaceae	*Erwinia*	*Erwinia endophytica*
Pseudomonadota	Enterobacteriaceae	*Erwinia*	*Erwinia gerundensis*
Pseudomonadota	Enterobacteriaceae	*Erwinia*	*Erwinia iniecta*
Pseudomonadota	Enterobacteriaceae	*Erwinia*	*Erwinia mallotivora*
Pseudomonadota	Enterobacteriaceae	*Erwinia*	*Erwinia oleae*
Pseudomonadota	Enterobacteriaceae	*Erwinia*	*Erwinia papayae*

Pseudomonadota	Enterobacteriaceae	*Erwinia*	*Erwinia persiciErwinia*
Pseudomonadota	Enterobacteriaceae	*Erwinia*	*Erwinia persicina*
Pseudomonadota	Enterobacteriaceae	*Erwinia*	*Erwinia piriflorinigrans*
Pseudomonadota	Enterobacteriaceae	*Erwinia*	*Erwinia psidii*
Pseudomonadota	Enterobacteriaceae	*Erwinia*	*Erwinia pyrifoliae*
Pseudomonadota	Enterobacteriaceae	*Erwinia*	*Erwinia rhapontici*
Pseudomonadota	Enterobacteriaceae	*Erwinia*	*Erwinia tasmaniensis*
Pseudomonadota	Enterobacteriaceae	*Erwinia*	*Erwinia teleogrylli*
Pseudomonadota	Enterobacteriaceae	*Erwinia*	*Erwinia toletana*
Pseudomonadota	Enterobacteriaceae	*Erwinia*	*Erwinia tracheiphila*
Pseudomonadota	Enterobacteriaceae	*Erwinia*	*Erwinia typographi*
Pseudomonadota	Enterobacteriaceae	*Erwinia*	*Erwinia uzenensis*
Pseudomonadota	Sphingomonadaceae	*Erythrobacter*	*Erythrobacter lutimaris*
Pseudomonadota	Sphingomonadaceae	*Erythrobacter*	*Erythrobacter odishensis*
Pseudomonadota	Enterobacteriaceae	*Escherichia*	*Escherichia albertii*
Pseudomonadota	Enterobacteriaceae	*Escherichia*	*Escherichia coli*
Pseudomonadota	Enterobacteriaceae	*Escherichia*	*Escherichia fergusonii*
Pseudomonadota	Enterobacteriaceae	*Escherichia*	*Escherichia marmotae*
Pseudomonadota	Enterobacteriaceae	*Ewingella*	*Ewingella americana*
Pseudomonadota	Burkholderiaceae	*Extensimonas*	*Extensimonas perlucida*
Pseudomonadota	Francisellaceae	*Fangia*	*Fangia hongkongensis*
Pseudomonadota	Francisellaceae	*Fastidiosibacter*	*Fastidiosibacter lacustris*
Pseudomonadota	Shewanellaceae	*Ferrimonas*	*Ferrimonas balearica*
Pseudomonadota	Shewanellaceae	*Ferrimonas*	*Ferrimonas futtsuensis*
Pseudomonadota	Shewanellaceae	*Ferrimonas*	*Ferrimonas marina*
Pseudomonadota	Shewanellaceae	*Ferrimonas*	*Ferrimonas pelagia*
Pseudomonadota	Hyphomicrobiaceae	*Filomicrobium*	*Filomicrobium insigne*
Pseudomonadota	Fluviibactteraceae	*Fluviibacter*	*Fluviibacter phosphoraccumulans*

Pseudomonadota	Francisellaceae	*Francisella*	*Francisella persica*
Pseudomonadota	Enterobacteriaceae	*Franconibacter*	*Franconibacter daqui*
Pseudomonadota	Enterobacteriaceae	*Franconibacter*	*Franconibacter helveticus*
Pseudomonadota	Enterobacteriaceae	*Franconibacter*	*Franconibacter pulveris*
Pseudomonadota	Rhodanobacteraceae	*Frateuria*	*Frateuria aurantia*
Pseudomonadota	Pasteurellaceae	*Frederiksenia*	*Frederiksenia canicola*
Pseudomonadota	Enterobacteriaceae	*Frischella*	*Frischella perrara*
Pseudomonadota	Gallaecimonadaceae	*Gallaecimonas*	*Gallaecimonas pentaromativorans*
Pseudomonadota	Gallaecimonadaceae	*Gallaecimonas*	*Gallaecimonas xiamenensis*
Pseudomonadota	Pasteurellaceae	*Gallibacterium*	*Gallibacterium salpingitidis*
Pseudomonadota	Pasteurellaceae	*Gallibacterium*	*Gallibacterium trehalosifermentans*
Pseudomonadota	Gallionellaceae	*Gallionella*	*Gallionella capsiferriformans*
Pseudomonadota	Rhodobacteraceae	*Gemmobacter*	*Gemmobacter aquatilis*
Pseudomonadota	Sterolibacteriaceae	*Georgfuchsia*	*Georgfuchsia toluolica*
Pseudomonadota	Enterobacteriaceae	*Gibbsiella*	*Gibbsiella dentisursi*
Pseudomonadota	Enterobacteriaceae	*Gibbsiella*	*Gibbsiella greigii*
Pseudomonadota	Enterobacteriaceae	*Gibbsiella*	*Gibbsiella quercinecans*
Pseudomonadota	Enterobacteriaceae	*Gilliamella*	*Gilliamella apicola*
Pseudomonadota	Enterobacteriaceae	*Gilliamella*	*Gilliamella bombi*
Pseudomonadota	Enterobacteriaceae	*Gilliamella*	*Gilliamella bombicola*
Pseudomonadota	Enterobacteriaceae	*Gilliamella*	*Gilliamella intestini*
Pseudomonadota	Enterobacteriaceae	*Gilliamella*	*Gilliamella mensalis*
Pseudomonadota	Pasteurellaceae	*Glaesserella*	*Glaesserella parasuis*
Pseudomonadota	Acetobacteraceae	*Gluconacetobacter*	*Gluconacetobacter johannae*
Pseudomonadota	Acetobacteraceae	*Gluconacetobacter*	*Gluconacetobacter liquefaciens*
Pseudomonadota	Acetobacteraceae	*Gluconacetobacter*	*Gluconacetobacter sacchari*
Pseudomonadota	Acetobacteraceae	*Gluconacetobacter*	*Gluconacetobacter takamatsuzukensis*
Pseudomonadota	Acetobacteraceae	*Gluconobacter*	*Gluconobacter cerevisiae*

Pseudomonadota	Acetobacteraceae	*Gluconobacter*	*Gluconobacter cerinus*
Pseudomonadota	Acetobacteraceae	*Gluconobacter*	*Gluconobacter frateurii*
Pseudomonadota	Acetobacteraceae	*Gluconobacter*	*Gluconobacter japonicus*
Pseudomonadota	Acetobacteraceae	*Gluconobacter*	*Gluconobacter kanchanaburiensis*
Pseudomonadota	Acetobacteraceae	*Gluconobacter*	*Gluconobacter kondonii*
Pseudomonadota	Acetobacteraceae	*Gluconobacter*	*Gluconobacter oxydans*
Pseudomonadota	Acetobacteraceae	*Gluconobacter*	*Gluconobacter sphaericus*
Pseudomonadota	Acetobacteraceae	*Gluconobacter*	*Gluconobacter thailandicus*
Pseudomonadota	Acetobacteraceae	*Gluconobacter*	*Gluconobacter wancherniae*
Pseudomonadota	Maricaulaceae	*Glycocaulis*	*Glycocaulis albus*
Pseudomonadota	Granulosicoccaceae	*Granulosicoccus*	*Granulosicoccus antarcticus*
Pseudomonadota	Granulosicoccaceae	*Granulosicoccus*	*Granulosicoccus coccoides*
Pseudomonadota	Vibrionaceae	*Grimontia*	*Grimontia celer*
Pseudomonadota	Vibrionaceae	*Grimontia*	*Grimontia hollisae*
Pseudomonadota	Vibrionaceae	*Grimontia*	*Grimontia marina*
Pseudomonadota	Rhodobacteraceae	*Haematobacter*	*Haematobacter massiliensis*
Pseudomonadota	Rhodobacteraceae	*Haematobacter*	*Haematobacter missouriensis*
Pseudomonadota	Pasteurellaceae	*Haemophilus*	*Haemophilus ducreyi*
Pseudomonadota	Pasteurellaceae	*Haemophilus*	*Haemophilus felis*
Pseudomonadota	Pasteurellaceae	*Haemophilus*	*Haemophilus haemoglobinophilus*
Pseudomonadota	Pasteurellaceae	*Haemophilus*	*Haemophilus haemolyticus*
Pseudomonadota	Pasteurellaceae	*Haemophilus*	*Haemophilus influenzae*
Pseudomonadota	Pasteurellaceae	*Haemophilus*	*Haemophilus massiliensis*
Pseudomonadota	Pasteurellaceae	*Haemophilus*	*Haemophilus paracuniculus*
Pseudomonadota	Pasteurellaceae	*Haemophilus*	*Haemophilus parahaemolyticus*
Pseudomonadota	Pasteurellaceae	*Haemophilus*	*Haemophilus parainfluenzae*
Pseudomonadota	Pasteurellaceae	*Haemophilus*	*Haemophilus paraphrohaemolyticus*
Pseudomonadota	Pasteurellaceae	*Haemophilus*	*Haemophilus piscium*

Pseudomonadota	Pasteurellaceae	*Haemophilus*	*Haemophilus pittmaniae*
Pseudomonadota	Pasteurellaceae	*Haemophilus*	*Haemophilus sputorum*
Pseudomonadota	Enterobacteriaceae	*Hafnia*	*Hafnia alvei*
Pseudomonadota	Enterobacteriaceae	*Hafnia*	*Hafnia paralvei*
Pseudomonadota	Enterobacteriaceae	*Hafnia*	*Hafnia psychrotolerans*
Pseudomonadota	Oleiphilaceae	*Hahella*	*Hahella ganghwensis*
Pseudomonadota	Halieaceae	*Haliea*	*Haliea salexigens*
Pseudomonadota	Halomonadaceae	*Halomonas*	*Halomonas flava*
Pseudomonadota	Halomonadaceae	*Halomonas*	*Halomonas meridiana*
Pseudomonadota	Halomonadaceae	*Halomonas*	*Halomonas pacifica*
Pseudomonadota	Halomonadaceae	*Halomonas*	*Halomonas sabkhae*
Pseudomonadota	Halomonadaceae	*Halomonas*	*Halomonas titanicae*
Pseudomonadota	Pseudomonadaceae	*Halopseudomonas*	*Halopseudomonas bauzanensis*
Pseudomonadota	Pseudomonadaceae	*Halopseudomonas*	*Halopseudomonas oceani*
Pseudomonadota	Pseudomonadaceae	*Halopseudomonas*	*Halopseudomonas pachastrellae*
Pseudomonadota	Pseudomonadaceae	*Halopseudomonas*	*Halopseudomonas pertucinogena*
Pseudomonadota	Pseudomonadaceae	*Halopseudomonas*	*Halopseudomonas salina*
Pseudomonadota	Pseudomonadaceae	*Halopseudomonas*	*Halopseudomonas xiamenensis*
Pseudomonadota	Pseudomonadaceae	*Halopseudomonas*	*Halopseudomonas xinjiangensis*
Pseudomonadota	Burkholderiaceae	*Herbaspirillum*	*Herbaspirillum seropedicae*
Pseudomonadota	Burkholderiaceae	*Herminiimonas*	*Herminiimonas saxobsidens*
Pseudomonadota	Rhizobiaceae	*Hoeflea*	*Hoeflea marina*
Pseudomonadota	Burkholderiaceae	*Hydrogenophaga*	*Hydrogenophaga aquatica*
Pseudomonadota	Thiomicrospiraceae	*Hydrogenovibrio*	*Hydrogenovibrio kuenenii*
Pseudomonadota	Hyphomicrobiaceae	*Hyphomicrobium*	*Hyphomicrobium chloromethanicum*
Pseudomonadota	Hyphomicrobiaceae	*Hyphomicrobium*	*Hyphomicrobium denitrificans*
Pseudomonadota	Hyphomicrobiaceae	*Hyphomicrobium*	*Hyphomicrobium facile*
Pseudomonadota	Hyphomicrobiaceae	*Hyphomicrobium*	*Hyphomicrobium hollandicum*

Pseudomonadota	Hyphomicrobiaceae	*Hyphomicrobium*	*Hyphomicrobium methylovorum*
Pseudomonadota	Hyphomicrobiaceae	*Hyphomicrobium*	*Hyphomicrobium nitrativorans*
Pseudomonadota	Hyphomicrobiaceae	*Hyphomicrobium*	*Hyphomicrobium sulfonivorans*
Pseudomonadota	Hyphomicrobiaceae	*Hyphomicrobium*	*Hyphomicrobium vulgare*
Pseudomonadota	Hyphomicrobiaceae	*Hyphomicrobium*	*Hyphomicrobium zavarzinii*
Pseudomonadota	Alteromonadaceae	*Idiomarina*	*Idiomarina andamanensis*
Pseudomonadota	Alteromonadaceae	*Idiomarina*	*Idiomarina aquatica*
Pseudomonadota	Alteromonadaceae	*Idiomarina*	*Idiomarina baltica*
Pseudomonadota	Alteromonadaceae	*Idiomarina*	*Idiomarina loihiensis*
Pseudomonadota	Alteromonadaceae	*Idiomarina*	*Idiomarina piscisalsi*
Pseudomonadota	Alteromonadaceae	*Idiomarina*	*Idiomarina seosinensis*
Pseudomonadota	Alteromonadaceae	*Idiomarina*	*Idiomarina tyrosinivorans*
Pseudomonadota	Alteromonadaceae	*Idiomarina*	*Idiomarina xiamenensis*
Pseudomonadota	Immundisolibacteraceae	*Immundisolibacter*	*Immundisolibacter cernigliae*
Pseudomonadota	Chitinibacteraceae	*Iodobacter*	*Iodobacter arcticus*
Pseudomonadota	Enterobacteriaceae	*Izhakiella*	*Izhakiella australiensis*
Pseudomonadota	Enterobacteriaceae	*Izhakiella*	*Izhakiella capsodis*
Pseudomonadota	Kaistiaceae	*Kaistia*	*Kaistia adipata*
Pseudomonadota	Kaistiaceae	*Kaistia*	*Kaistia dalseonensis*
Pseudomonadota	Kaistiaceae	*Kaistia*	*Kaistia geumhonensis*
Pseudomonadota	Kangiellaceae	*Kangiella*	*Kangiella sediminilitoris*
Pseudomonadota	Kiloniellaceae	*Kiloniella*	*Kiloniella antarctica*
Pseudomonadota	Kiloniellaceae	*Kiloniella*	*Kiloniella majae*
Pseudomonadota	Neisseriaceae	*Kingella*	*Kingella negevensis*
Pseudomonadota	Enterobacteriaceae	*Klebsiella*	*Klebsiella aerogenes*
Pseudomonadota	Enterobacteriaceae	*Klebsiella*	*Klebsiella grimontii*
Pseudomonadota	Enterobacteriaceae	*Klebsiella*	*Klebsiella huaxiensis*
Pseudomonadota	Enterobacteriaceae	*Klebsiella*	*Klebsiella michiganensis*

Pseudomonadota	Enterobacteriaceae	*Klebsiella*	*Klebsiella oxytoca*
Pseudomonadota	Enterobacteriaceae	*Klebsiella*	*Klebsiella pneumoniae*
Pseudomonadota	Enterobacteriaceae	*Klebsiella*	*Klebsiella quasipneumoniae*
Pseudomonadota	Enterobacteriaceae	*Klebsiella*	*Klebsiella variicola*
Pseudomonadota	Enterobacteriaceae	*Kluyvera*	*Kluyvera ascorbata*
Pseudomonadota	Enterobacteriaceae	*Kluyvera*	*Kluyvera cryocrescens*
Pseudomonadota	Enterobacteriaceae	*Kluyvera*	*Kluyvera georgiaKluyvera*
Pseudomonadota	Enterobacteriaceae	*Kluyvera*	*Kluyvera georgiana*
Pseudomonadota	Enterobacteriaceae	*Kluyvera*	*Kluyvera intermedia*
Pseudomonadota	Acetobacteraceae	*Komagataeibacter*	*Komagataeibacter europaeus*
Pseudomonadota	Acetobacteraceae	*Komagataeibacter*	*Komagataeibacter intermedius*
Pseudomonadota	Acetobacteraceae	*Komagataeibacter*	*Komagataeibacter medellinensis*
Pseudomonadota	Acetobacteraceae	*Komagataeibacter*	*Komagataeibacter saccharivorans*
Pseudomonadota	Acetobacteraceae	*Komagataeibacter*	*Komagataeibacter sucrofermentans*
Pseudomonadota	Enterobacteriaceae	*Kosakonia*	*Kosakonia arachidis*
Pseudomonadota	Enterobacteriaceae	*Kosakonia*	*Kosakonia cowanii*
Pseudomonadota	Enterobacteriaceae	*Kosakonia*	*Kosakonia oryzae*
Pseudomonadota	Enterobacteriaceae	*Kosakonia*	*Kosakonia oryzendophytica*
Pseudomonadota	Enterobacteriaceae	*Kosakonia*	*Kosakonia oryziphila*
Pseudomonadota	Enterobacteriaceae	*Kosakonia*	*Kosakonia pseudosacchari*
Pseudomonadota	Enterobacteriaceae	*Kosakonia*	*Kosakonia quasisacchari*
Pseudomonadota	Enterobacteriaceae	*Kosakonia*	*Kosakonia radicincitans*
Pseudomonadota	Enterobacteriaceae	*Kosakonia*	*Kosakonia sacchari*
Pseudomonadota	Halomonadaceae	*Kushneria*	*Kushneria indalinina*
Pseudomonadota	Labraceae	*Labrys*	*Labrys miyagiensis*
Pseudomonadota	Labraceae	*Labrys*	*Labrys monachus*
Pseudomonadota	Labraceae	*Labrys*	*Labrys neptuniae*
Pseudomonadota	Labraceae	*Labrys*	*Labrys okinawensis*

Pseudomonadota	Labraceae	*Labrys*	*Labrys portucalensis*
Pseudomonadota	Labraceae	*Labrys*	*Labrys wisconsinensis*
Pseudomonadota	Chromatiaceae	*Lamprocystis*	*Lamprocystis purpurea*
Pseudomonadota	Burkholderiaceae	*Lampropedia*	*Lampropedia aestuarii*
Pseudomonadota	Burkholderiaceae	*Lampropedia*	*Lampropedia hyalina*
Pseudomonadota	Burkholderiaceae	*Lampropedia*	*Lampropedia puyangensis*
Pseudomonadota	Burkholderiaceae	*Lautropia*	*Lautropia mirabilis*
Pseudomonadota	Enterobacteriaceae	*Leclercia*	*Leclercia adecarboxylata*
Pseudomonadota	Legionellaceae	*Legionella*	*Legionella beliardensis*
Pseudomonadota	Legionellaceae	*Legionella*	*Legionella brunensis*
Pseudomonadota	Legionellaceae	*Legionella*	*Legionella busanensis*
Pseudomonadota	Legionellaceae	*Legionella*	*Legionella cincinnatiensis*
Pseudomonadota	Legionellaceae	*Legionella*	*Legionella drancourtii*
Pseudomonadota	Legionellaceae	*Legionella*	*Legionella dresdenensis*
Pseudomonadota	Legionellaceae	*Legionella*	*Legionella geestiana*
Pseudomonadota	Legionellaceae	*Legionella*	*Legionella gresilensis*
Pseudomonadota	Legionellaceae	*Legionella*	*Legionella lansingensis*
Pseudomonadota	Legionellaceae	*Legionella*	*Legionella longbeachae*
Pseudomonadota	Legionellaceae	*Legionella*	*Legionella pneumophila*
Pseudomonadota	Legionellaceae	*Legionella*	*Legionella qingyii*
Pseudomonadota	Legionellaceae	*Legionella*	*Legionella rowbothamii*
Pseudomonadota	Legionellaceae	*Legionella*	*Legionella sainthelensi*
Pseudomonadota	Legionellaceae	*Legionella*	*Legionella saoudiensis*
Pseudomonadota	Legionellaceae	*Legionella*	*Legionella shakespearei*
Pseudomonadota	Legionellaceae	*Legionella*	*Legionella taurinensis*
Pseudomonadota	Legionellaceae	*Legionella*	*Legionella tunisiensis*
Pseudomonadota	Enterobacteriaceae	*Lelliottia*	*Lelliottia amnigena*
Pseudomonadota	Enterobacteriaceae	*Lelliottia*	*Lelliottia nimipressuralis*

Pseudomonadota	Enterobacteriaceae	*Leminorella*	*Leminorella grimontii*
Pseudomonadota	Phyllobacteriaceae	*Lentilitoribacter*	*Lentilitoribacter donghaensis*
Pseudomonadota	Burkholderiaceae	*Leptothrix*	*Leptothrix mobilis*
Pseudomonadota	Rhizobiaceae	*Liberibacter*	*Liberibacter crescens*
Pseudomonadota	Lichenibacteriaceae	*Lichenibacterium*	*Lichenibacterium ramalinae*
Pseudomonadota	Rhodovibrionaceae	*Limibacillus*	*Limibacillus halophilus*
Pseudomonadota	Burkholderiaceae	*Limnobacter*	*Limnobacter humi*
Pseudomonadota	Oceanospirillaceae	*Litoribacillus*	*Litoribacillus peritrichatus*
Pseudomonadota	Alteromonadaceae	*Litorilituus*	*Litorilituus lipolyticus*
Pseudomonadota	Pasteurellaceae	*Lonepinella*	*Lonepinella koalarum*
Pseudomonadota	Enterobacteriaceae	*Lonsdalea*	*Lonsdalea britannica*
Pseudomonadota	Enterobacteriaceae	*Lonsdalea*	*Lonsdalea iberica*
Pseudomonadota	Enterobacteriaceae	*Lonsdalea*	*Lonsdalea populi*
Pseudomonadota	Enterobacteriaceae	*Lonsdalea*	*Lonsdalea quercina*
Pseudomonadota	Rhodanobacteraceae	*Luteibacter*	*Luteibacter yeojuensis*
Pseudomonadota	Xanthomonadaceae	*Luteimonas*	*Luteimonas abyssi*
Pseudomonadota	Xanthomonadaceae	*Luteimonas*	*Luteimonas aquatica*
Pseudomonadota	Xanthomonadaceae	*Luteimonas*	*Luteimonas chenhongjianii*
Pseudomonadota	Xanthomonadaceae	*Luteimonas*	*Luteimonas huabeiensis*
Pseudomonadota	Tepidamorphaceae	*Lutibaculum*	*Lutibaculum baratangense*
Pseudomonadota	Xanthomonadaceae	*Lysobacter*	*Lysobacter brunescens*
Pseudomonadota	Xanthomonadaceae	*Lysobacter*	*Lysobacter concretionis*
Pseudomonadota	Xanthomonadaceae	*Lysobacter*	*Lysobacter firmicutimachus*
Pseudomonadota	Xanthomonadaceae	*Lysobacter*	*Lysobacter tyrosinelyticus*
Pseudomonadota	Magnetospiraceae	*Magnetospira*	*Magnetospira thiophila*
Pseudomonadota	Magnetospirillaceae	*Magnetospirillum*	*Magnetospirillum bellicus*
Pseudomonadota	Magnetovibrionaceae	*Magnetovibrio*	*Magnetovibrio blakemorei*
Pseudomonadota	Burkholderiaceae	*Malikia*	*Malikia granosa*

Pseudomonadota	Enterobacteriaceae	*Mangrovibacter*	*Mangrovibacter phragmitis*
Pseudomonadota	Enterobacteriaceae	*Mangrovibacter*	*Mangrovibacter plantisponsor*
Pseudomonadota	Enterobacteriaceae	*Mangrovibacter*	*Mangrovibacter yixingensis*
Pseudomonadota	Oleiphilaceae	*Mangrovitalea*	*Mangrovitalea sediminis*
Pseudomonadota	Pasteurellaceae	*Mannheimia*	*Mannheimia glucosida*
Pseudomonadota	Pasteurellaceae	*Mannheimia*	*Mannheimia granulomatis*
Pseudomonadota	Pasteurellaceae	*Mannheimia*	*Mannheimia haemolytica*
Pseudomonadota	Pasteurellaceae	*Mannheimia*	*Mannheimia ruminalis*
Pseudomonadota	Pasteurellaceae	*Mannheimia*	*Mannheimia succiniciproducens*
Pseudomonadota	Pasteurellaceae	*Mannheimia*	*Mannheimia varigena*
Pseudomonadota	Roseobacteraceae	*Maribius*	*Maribius salinus*
Pseudomonadota	Chromatiaceae	*Marichromatium*	*Marichromatium purpuratum*
Pseudomonadota	Halieaceae	*Marimicrobium*	*Marimicrobium arenosum*
Pseudomonadota	Alteromonadaceae	*Marinifaba*	*Marinifaba aquimaris*
Pseudomonadota	Oleiphilaceae	*Marinobacter*	*Marinobacter excellens*
Pseudomonadota	Oleiphilaceae	*Marinobacter*	*Marinobacter gudaonensis*
Pseudomonadota	Oleiphilaceae	*Marinobacter*	*Marinobacter lutaoensis*
Pseudomonadota	Oleiphilaceae	*Marinobacter*	*Marinobacter nauticus*
Pseudomonadota	Oleiphilaceae	*Marinobacter*	*Marinobacter profundi*
Pseudomonadota	Oleiphilaceae	*Marinobacter*	*Marinobacter vinifirmus*
Pseudomonadota	Nitrincolaceae	*Marinobacterium*	*Marinobacterium nitratireducens*
Pseudomonadota	Nitrincolaceae	*Marinobacterium*	*Marinobacterium zhoushanense*
Pseudomonadota	Marinomonadaceae	*Marinomonas*	*Marinomonas aquimarina*
Pseudomonadota	Marinomonadaceae	*Marinomonas*	*Marinomonas epiphytica*
Pseudomonadota	Marinomonadaceae	*Marinomonas*	*Marinomonas mediterranea*
Pseudomonadota	Marinomonadaceae	*Marinomonas*	*Marinomonas ostreistagni*
Pseudomonadota	Marinomonadaceae	*Marinomonas*	*Marinomonas polaris*
Pseudomonadota	Marinomonadaceae	*Marinomonas*	*Marinomonas pontica*

Pseudomonadota	Marinomonadaceae	*Marinomonas*	*Marinomonas vaga*
Pseudomonadota	Rhodobacteraceae	*Marinosulfonomonas*	*Marinosulfonomonas methylotropha*
Pseudomonadota	Alteromonadaceae	*Marisediminitalea*	*Marisediminitalea mangrovi*
Pseudomonadota	Rhodospirillaceae	*Marispirillum*	*Marispirillum indicum*
Pseudomonadota	Burkholderiaceae	*Massilia*	*Massilia agri*
Pseudomonadota	Burkholderiaceae	*Massilia*	*Massilia albidiflava*
Pseudomonadota	Burkholderiaceae	*Massilia*	*Massilia phosphatilytica*
Pseudomonadota	Burkholderiaceae	*Massilia*	*Massilia pinisoli*
Pseudomonadota	Rhizobiaceae	*Mesorhizobium*	*Mesorhizobium abyssinicae*
Pseudomonadota	Rhizobiaceae	*Mesorhizobium*	*Mesorhizobium acaciae*
Pseudomonadota	Rhizobiaceae	*Mesorhizobium*	*Mesorhizobium albiziae*
Pseudomonadota	Rhizobiaceae	*Mesorhizobium*	*Mesorhizobium amorphae*
Pseudomonadota	Rhizobiaceae	*Mesorhizobium*	*Mesorhizobium australicum*
Pseudomonadota	Rhizobiaceae	*Mesorhizobium*	*Mesorhizobium carbonis*
Pseudomonadota	Rhizobiaceae	*Mesorhizobium*	*Mesorhizobium chacoense*
Pseudomonadota	Rhizobiaceae	*Mesorhizobium*	*Mesorhizobium ciceri*
Pseudomonadota	Rhizobiaceae	*Mesorhizobium*	*Mesorhizobium delmotii*
Pseudomonadota	Rhizobiaceae	*Mesorhizobium*	*Mesorhizobium denitrificans*
Pseudomonadota	Rhizobiaceae	*Mesorhizobium*	*Mesorhizobium erdmanii*
Pseudomonadota	Rhizobiaceae	*Mesorhizobium*	*Mesorhizobium hankyongi*
Pseudomonadota	Rhizobiaceae	*Mesorhizobium*	*Mesorhizobium helmanticense*
Pseudomonadota	Rhizobiaceae	*Mesorhizobium*	*Mesorhizobium huakuii*
Pseudomonadota	Rhizobiaceae	*Mesorhizobium*	*Mesorhizobium jarvisii*
Pseudomonadota	Rhizobiaceae	*Mesorhizobium*	*Mesorhizobium loti*
Pseudomonadota	Rhizobiaceae	*Mesorhizobium*	*Mesorhizobium mediterraneum*
Pseudomonadota	Rhizobiaceae	*Mesorhizobium*	*Mesorhizobium muleiense*
Pseudomonadota	Rhizobiaceae	*Mesorhizobium*	*Mesorhizobium norvegicum*
Pseudomonadota	Rhizobiaceae	*Mesorhizobium*	*Mesorhizobium oceanicum*

Pseudomonadota	Rhizobiaceae	*Mesorhizobium*	*Mesorhizobium olivaresii*
Pseudomonadota	Rhizobiaceae	*Mesorhizobium*	*Mesorhizobium opportunistum*
Pseudomonadota	Rhizobiaceae	*Mesorhizobium*	*Mesorhizobium plurifarium*
Pseudomonadota	Rhizobiaceae	*Mesorhizobium*	*Mesorhizobium septentrionale*
Pseudomonadota	Rhizobiaceae	*Mesorhizobium*	*Mesorhizobium shonense*
Pseudomonadota	Rhizobiaceae	*Mesorhizobium*	*Mesorhizobium soli*
Pseudomonadota	Rhizobiaceae	*Mesorhizobium*	*Mesorhizobium sophorae*
Pseudomonadota	Rhizobiaceae	*Mesorhizobium*	*Mesorhizobium tamadayense*
Pseudomonadota	Rhizobiaceae	*Mesorhizobium*	*Mesorhizobium tianshanense*
Pseudomonadota	Enterobacteriaceae	*Metakosakonia*	*Metakosakonia massiliensis*
Pseudomonadota	Methylomonadaceae	*Methylobacter*	*Methylobacter marinus*
Pseudomonadota	Methylomonadaceae	*Methylobacter*	*Methylobacter tundripaludum*
Pseudomonadota	Beijerinckiaceae	*Methylobacterium*	*Methylobacterium aerolatum*
Pseudomonadota	Beijerinckiaceae	*Methylobacterium*	*Methylobacterium aquaticum*
Pseudomonadota	Beijerinckiaceae	*Methylobacterium*	*Methylobacterium brachiatum*
Pseudomonadota	Beijerinckiaceae	*Methylobacterium*	*Methylobacterium brachythecii*
Pseudomonadota	Beijerinckiaceae	*Methylobacterium*	*Methylobacterium dankookense*
Pseudomonadota	Beijerinckiaceae	*Methylobacterium*	*Methylobacterium durans*
Pseudomonadota	Beijerinckiaceae	*Methylobacterium*	*Methylobacterium gnaphalii*
Pseudomonadota	Beijerinckiaceae	*Methylobacterium*	*Methylobacterium goesingense*
Pseudomonadota	Beijerinckiaceae	*Methylobacterium*	*Methylobacterium haplocladii*
Pseudomonadota	Beijerinckiaceae	*Methylobacterium*	*Methylobacterium hispanicum*
Pseudomonadota	Beijerinckiaceae	*Methylobacterium*	*Methylobacterium isbiliense*
Pseudomonadota	Beijerinckiaceae	*Methylobacterium*	*Methylobacterium komagatae*
Pseudomonadota	Beijerinckiaceae	*Methylobacterium*	*Methylobacterium longum*
Pseudomonadota	Beijerinckiaceae	*Methylobacterium*	*Methylobacterium mesophilicum*
Pseudomonadota	Beijerinckiaceae	*Methylobacterium*	*Methylobacterium nodulans*
Pseudomonadota	Beijerinckiaceae	*Methylobacterium*	*Methylobacterium organophilum*

Pseudomonadota	Beijerinckiaceae	*Methylobacterium*	*Methylobacterium oryzae*
Pseudomonadota	Beijerinckiaceae	*Methylobacterium*	*Methylobacterium persicinum*
Pseudomonadota	Beijerinckiaceae	*Methylobacterium*	*Methylobacterium radiotolerans*
Pseudomonadota	Beijerinckiaceae	*Methylobacterium*	*Methylobacterium soli*
Pseudomonadota	Beijerinckiaceae	*Methylobacterium*	*Methylobacterium tardum*
Pseudomonadota	Beijerinckiaceae	*Methylocapsa*	*Methylocapsa acidiphila*
Pseudomonadota	Methyloligellaceae	*Methyloceanibacter*	*Methyloceanibacter caenitepidi*
Pseudomonadota	Beijerinckiaceae	*Methylocella*	*Methylocella silvestris*
Pseudomonadota	Beijerinckiaceae	*Methylocella*	*Methylocella tundrae*
Pseudomonadota	Methylococcaceae	*Methylococcus*	*Methylococcus capsulatus*
Pseudomonadota	Methylococcaceae	*Methylococcus*	*Methylococcus thermophilus*
Pseudomonadota	Beijerinckiaceae	*Methylocystis*	*Methylocystis heyeri*
Pseudomonadota	Methylococcaceae	*Methylogaea*	*Methylogaea oryzae*
Pseudomonadota	Methylomonadaceae	*Methylomicrobium*	*Methylomicrobium alcaliphilum*
Pseudomonadota	Methylomonadaceae	*Methylomonas*	*Methylomonas koyamae*
Pseudomonadota	Methylomonadaceae	*Methylomonas*	*Methylomonas lenta*
Pseudomonadota	Methylomonadaceae	*Methylomonas*	*Methylomonas methanica*
Pseudomonadota	Methylomonadaceae	*Methylomonas*	*Methylomonas rubra*
Pseudomonadota	Methylococcaceae	*Methyloparacoccus*	*Methyloparacoccus murrellii*
Pseudomonadota	Methylophagaceae	*Methylophaga*	*Methylophaga aminisulfidivorans*
Pseudomonadota	Methylophagaceae	*Methylophaga*	*Methylophaga thalassica*
Pseudomonadota	Methylopilaceae	*Methylopila*	*Methylopila jiangsuensis*
Pseudomonadota	Methylopilaceae	*Methylopila*	*Methylopila oligotropha*
Pseudomonadota	Methylobacteriaceae	*Methylorubrum*	*Methylorubrum extorquens*
Pseudomonadota	Methylobacteriaceae	*Methylorubrum*	*Methylorubrum podarium*
Pseudomonadota	Methylobacteriaceae	*Methylorubrum*	*Methylorubrum populi*
Pseudomonadota	Methylobacteriaceae	*Methylorubrum*	*Methylorubrum salsuginis*
Pseudomonadota	Methylobacteriaceae	*Methylorubrum*	*Methylorubrum suomiense*

Pseudomonadota	Methylomonadaceae	*Methylosarcina*	*Methylosarcina quisquiliarum*
Pseudomonadota	Beijerinckiaceae	*Methylosinus*	*Methylosinus trichosporium*
Pseudomonadota	Methylococcaceae	*Methylosphaera*	*Methylosphaera hansonii*
Pseudomonadota	Methylococcaceae	*Methylotuvimicrobium*	*Methylotuvimicrobium alcaliphilum*
Pseudomonadota	Methylococcaceae	*Methylotuvimicrobium*	*Methylotuvimicrobium japanense*
Pseudomonadota	Methylococcaceae	*Methylotuvimicrobium*	*Methylotuvimicrobium kenyense*
Pseudomonadota	Rhodocyclaceae	*Methyloversatilis*	*Methyloversatilis discipulorum*
Pseudomonadota	Methylophilaceae	*Methylovorus*	*Methylovorus mays*
Pseudomonadota	Tepidamorphaceae	*Microbaculum*	*Microbaculum marinum*
Pseudomonadota	Cellvibrionaceae	*Microbulbifer*	*Microbulbifer arenaceous*
Pseudomonadota	Cellvibrionaceae	*Microbulbifer*	*Microbulbifer flavimaris*
Pseudomonadota	Cellvibrionaceae	*Microbulbifer*	*Microbulbifer halophilus*
Pseudomonadota	Cellvibrionaceae	*Microbulbifer*	*Microbulbifer salipaludis*
Pseudomonadota	Beijerinckiaceae	*Microvirga*	*Microvirga aerilata*
Pseudomonadota	Beijerinckiaceae	*Microvirga*	*Microvirga aerophila*
Pseudomonadota	Beijerinckiaceae	*Microvirga*	*Microvirga lotononidis*
Pseudomonadota	Beijerinckiaceae	*Microvirga*	*Microvirga lupini*
Pseudomonadota	Beijerinckiaceae	*Microvirga*	*Microvirga makkahensis*
Pseudomonadota	Beijerinckiaceae	*Microvirga*	*Microvirga massiliensis*
Pseudomonadota	Beijerinckiaceae	*Microvirga*	*Microvirga soli*
Pseudomonadota	Beijerinckiaceae	*Microvirga*	*Microvirga zambiensis*
Pseudomonadota	Aquaspirillaceae	*Microvirgula*	*Microvirgula aerodenitrificans*
Pseudomonadota	Burkholderiaceae	*Mitsuaria*	*Mitsuaria chitosanitabida*
Pseudomonadota	Enterobacteriaceae	*Mixta*	*Mixta calida*
Pseudomonadota	Enterobacteriaceae	*Mixta*	*Mixta gaviniae*
Pseudomonadota	Enterobacteriaceae	*Mixta*	*Mixta intestinalis*
Pseudomonadota	Enterobacteriaceae	*Mixta*	*Mixta tenebrionis*
Pseudomonadota	Enterobacteriaceae	*Mixta*	*Mixta theicola*

Pseudomonadota	Halomonadaceae	*Modicisalibacter*	*Modicisalibacter tunisiensis*
Pseudomonadota	Enterobacteriaceae	*Moellerella*	*Moellerella wisconsensis*
Pseudomonadota	Pleomorphomonadaceae	*Mongoliimonas*	*Mongoliimonas terrestris*
Pseudomonadota	Moraxellaceae	*Moraxella*	*Moraxella bovoculi*
Pseudomonadota	Moraxellaceae	*Moraxella*	*Moraxella catarrhalis*
Pseudomonadota	Moraxellaceae	*Moraxella*	*Moraxella lacunata*
Pseudomonadota	Moraxellaceae	*Moraxella*	*Moraxella nonliquefaciens*
Pseudomonadota	Moraxellaceae	*Moraxella*	*Moraxella osloensis*
Pseudomonadota	Moraxellaceae	*Moraxella*	*Moraxella porci*
Pseudomonadota	Enterobacteriaceae	*Morganella*	*Morganella morganii*
Pseudomonadota	Enterobacteriaceae	*Morganella*	*Morganella psychrotolerans*
Pseudomonadota	Psychromonadaceae	*Moritella*	*Moritella marina*
Pseudomonadota	Psychromonadaceae	*Moritella*	*Moritella viscosa*
Pseudomonadota	Psychromonadaceae	*Motilimonas*	*Motilimonas eburnea*
Pseudomonadota	Psychromonadaceae	*Motilimonas*	*Motilimonas pumilua*
Pseudomonadota	Pasteurellaceae	*Muribacter*	*Muribacter muris*
Pseudomonadota	Pectobacteriaceae	*Musicola*	*Musicola keenii*
Pseudomonadota	Pectobacteriaceae	*Musicola*	*Musicola paradisiaca*
Pseudomonadota	Rhizobiaceae	*Mycoplana*	*Mycoplana dimorpha*
Pseudomonadota	Rhizobiaceae	*Mycoplana*	*Mycoplana ramosa*
Pseudomonadota	Pasteurellaceae	*Necropsobacter*	*Necropsobacter rosorum*
Pseudomonadota	Neiellaceae	*Neiella*	*Neiella marina*
Pseudomonadota	Neisseriaceae	*Neisseria*	*Neisseria animaloris*
Pseudomonadota	Neisseriaceae	*Neisseria*	*Neisseria canis*
Pseudomonadota	Neisseriaceae	*Neisseria*	*Neisseria cinerea*
Pseudomonadota	Neisseriaceae	*Neisseria*	*Neisseria dumasiana*
Pseudomonadota	Neisseriaceae	*Neisseria*	*Neisseria perflava*
Pseudomonadota	Neisseriaceae	*Neisseria*	*Neisseria shayeganii*

Pseudomonadota	Neisseriaceae	*Neisseria*	*Neisseria sicca*
Pseudomonadota	Neisseriaceae	*Neisseria*	*Neisseria wadsworthii*
Pseudomonadota	Neisseriaceae	*Neisseria*	*Neisseria weaveri*
Pseudomonadota	Neisseriaceae	*Neisseria*	*Neisseria zalophi*
Pseudomonadota	Rhizobiaceae	*Neorhizobium*	*Neorhizobium galegae*
Pseudomonadota	Nitrincolaceae	*Neptunomonas*	*Neptunomonas acidivorans*
Pseudomonadota	Enterobacteriaceae	*Nissabacter*	*Nissabacter archeti*
Pseudomonadota	Nitrincolaceae	*Nitrincola*	*Nitrincola alkalilacustris*
Pseudomonadota	Nitrincolaceae	*Nitrincola*	*Nitrincola alkalisediminis*
Pseudomonadota	Nitrincolaceae	*Nitrincola*	*Nitrincola schmidtii*
Pseudomonadota	Xanthobacteraceae	*Nitrobacter*	*Nitrobacter vulgaris*
Pseudomonadota	Nitrococcaceae	*Nitrococcus*	*Nitrococcus mobilis*
Pseudomonadota	Zoogloeaceae	*Nitrogeniibacter*	*Nitrogeniibacter mangrovi*
Pseudomonadota	Nitrosococcaceae	*Nitrosococcus*	*Nitrosococcus halophilus*
Pseudomonadota	Nitrosomonadaceae	*Nitrosomonas*	*Nitrosomonas communis*
Pseudomonadota	Nitrosomonadaceae	*Nitrosospira*	*Nitrosospira multiformis*
Pseudomonadota	Azospirillaceae	*Niveispirillum*	*Niveispirillum irakense*
Pseudomonadota	Burkholderiaceae	*Noviherbaspirillum*	*Noviherbaspirillum aurantiacum*
Pseudomonadota	Burkholderiaceae	*Noviherbaspirillum*	*Noviherbaspirillum canariense*
Pseudomonadota	Burkholderiaceae	*Noviherbaspirillum*	*Noviherbaspirillum soli*
Pseudomonadota	Rhodospirillaceae	*Novispirillum*	*Novispirillum itersonii*
Pseudomonadota	Sphingomonadaceae	*Novosphingobium*	*Novosphingobium aquaticum*
Pseudomonadota	Sphingomonadaceae	*Novosphingobium*	*Novosphingobium aquiterrae*
Pseudomonadota	Sphingomonadaceae	*Novosphingobium*	*Novosphingobium capsulatum*
Pseudomonadota	Sphingomonadaceae	*Novosphingobium*	*Novosphingobium fuchskuhlense*
Pseudomonadota	Sphingomonadaceae	*Novosphingobium*	*Novosphingobium guangzhouense*
Pseudomonadota	Sphingomonadaceae	*Novosphingobium*	*Novosphingobium humi*
Pseudomonadota	Sphingomonadaceae	*Novosphingobium*	*Novosphingobium indicum*

Pseudomonadota	Sphingomonadaceae	*Novosphingobium*	*Novosphingobium lotistagni*
Pseudomonadota	Sphingomonadaceae	*Novosphingobium*	*Novosphingobium pokkalii*
Pseudomonadota	Sphingomonadaceae	*Novosphingobium*	*Novosphingobium rosa*
Pseudomonadota	Sphingomonadaceae	*Novosphingobium*	*Novosphingobium sediminis*
Pseudomonadota	Hafniaceae	*Obesumbacterium*	*Obesumbacterium proteus*
Pseudomonadota	Rickettsiaceae	*Occidentia*	*Occidentia massiliensis*
Pseudomonadota	Sneathiellaceae	*Oceanibacterium*	*Oceanibacterium hippocampi*
Pseudomonadota	Oceanibaculaceae	*Oceanibaculum*	*Oceanibaculum nanhaiense*
Pseudomonadota	Oceanibaculaceae	*Oceanibaculum*	*Oceanibaculum pacificum*
Pseudomonadota	Rhodobacteraceae	*Oceanicella*	*Oceanicella actignis*
Pseudomonadota	Aeromonadaceae	*Oceanimonas*	*Oceanimonas doudoroffii*
Pseudomonadota	Aeromonadaceae	*Oceanimonas*	*Oceanimonas smirnovii*
Pseudomonadota	Aeromonadaceae	*Oceanisphaera*	*Oceanisphaera arctica*
Pseudomonadota	Aeromonadaceae	*Oceanisphaera*	*Oceanisphaera donghaensis*
Pseudomonadota	Aeromonadaceae	*Oceanisphaera*	*Oceanisphaera litoralis*
Pseudomonadota	Aeromonadaceae	*Oceanisphaera*	*Oceanisphaera marina*
Pseudomonadota	Aeromonadaceae	*Oceanisphaera*	*Oceanisphaera ostreae*
Pseudomonadota	Aeromonadaceae	*Oceanisphaera*	*Oceanisphaera profunda*
Pseudomonadota	Aeromonadaceae	*Oceanisphaera*	*Oceanisphaera psychrotolerans*
Pseudomonadota	Halomonadaceae	*Oceanospirillum*	*Oceanospirillum maris*
Pseudomonadota	Rhizobiaceae	*Ochrobactrum*	*Ochrobactrum grignonense*
Pseudomonadota	Rhizobiaceae	*Ochrobactrum*	*Ochrobactrum haematophilum*
Pseudomonadota	Rhizobiaceae	*Ochrobactrum*	*Ochrobactrum pseudogrignonense*
Pseudomonadota	Rhizobiaceae	*Ochrobactrum*	*Ochrobactrum tritici*
Pseudomonadota	Nitrobacteraceae	*Oligotropha*	*Oligotropha carboxidovorans*
Pseudomonadota	Enterobacteriaceae	*Orbus*	*Orbus hercynius*
Pseudomonadota	Enterobacteriaceae	*Orbus*	*Orbus sasakiae*
Pseudomonadota	Rickettsiaceae	*Orientia*	*Orientia chuto*

Pseudomonadota	Burkholderiaceae	*Orrella*	*Orrella dioscoreae*
Pseudomonadota	Ostreibacteriaceae	*Ostreibacterium*	*Ostreibacterium oceani*
Pseudomonadota	Pasteurellaceae	*Otariodibacter*	*Otariodibacter oris*
Pseudomonadota	Oxalobacteraceae	*Oxalicibacterium*	*Oxalicibacterium flavum*
Pseudomonadota	Burkholderiaceae	*Oxalobacter*	*Oxalobacter formigenes*
Pseudomonadota	Burkholderiaceae	*Oxalobacter*	*Oxalobacter vibrioformis*
Pseudomonadota	Burkholderiaceae	*Paenalcaligenes*	*Paenalcaligenes hominis*
Pseudomonadota	Rhodobacteraceae	*Paenirhodobacter*	*Paenirhodobacter enshiensis*
Pseudomonadota	Chromobacteriaceae	*Paludibacterium*	*Paludibacterium paludis*
Pseudomonadota	Chromobacteriaceae	*Paludibacterium*	*Paludibacterium purpuratum*
Pseudomonadota	Burkholderiaceae	*Pandoraea*	*Pandoraea aquatica*
Pseudomonadota	Burkholderiaceae	*Pandoraea*	*Pandoraea capi*
Pseudomonadota	Enterobacteriaceae	*Pantoea*	*Pantoea agglomerans*
Pseudomonadota	Enterobacteriaceae	*Pantoea*	*Pantoea allii*
Pseudomonadota	Enterobacteriaceae	*Pantoea*	*Pantoea ananatis*
Pseudomonadota	Enterobacteriaceae	*Pantoea*	*Pantoea anthophila*
Pseudomonadota	Enterobacteriaceae	*Pantoea*	*Pantoea beijingensis*
Pseudomonadota	Enterobacteriaceae	*Pantoea*	*Pantoea brenneri*
Pseudomonadota	Enterobacteriaceae	*Pantoea*	*Pantoea cypripedii*
Pseudomonadota	Enterobacteriaceae	*Pantoea*	*Pantoea deleyi*
Pseudomonadota	Enterobacteriaceae	*Pantoea*	*Pantoea dispersa*
Pseudomonadota	Enterobacteriaceae	*Pantoea*	*Pantoea eucalypti*
Pseudomonadota	Enterobacteriaceae	*Pantoea*	*Pantoea eucrina*
Pseudomonadota	Enterobacteriaceae	*Pantoea*	*Pantoea rodasii*
Pseudomonadota	Enterobacteriaceae	*Pantoea*	*Pantoea rwandensis*
Pseudomonadota	Enterobacteriaceae	*Pantoea*	*Pantoea septica*
Pseudomonadota	Enterobacteriaceae	*Pantoea*	*Pantoea stewartii*
Pseudomonadota	Enterobacteriaceae	*Pantoea*	*Pantoea vagans*

Pseudomonadota	Enterobacteriaceae	*Pantoea*	*Pantoea wallisii*
Pseudomonadota	Burkholderiaceae	*Paraburkholderia*	*Paraburkholderia acidipaludis*
Pseudomonadota	Burkholderiaceae	*Paraburkholderia*	*Paraburkholderia azotifigens*
Pseudomonadota	Burkholderiaceae	*Paraburkholderia*	*Paraburkholderia bannensis*
Pseudomonadota	Burkholderiaceae	*Paraburkholderia*	*Paraburkholderia caffeinilytica*
Pseudomonadota	Burkholderiaceae	*Paraburkholderia*	*Paraburkholderia dipogonis*
Pseudomonadota	Burkholderiaceae	*Paraburkholderia*	*Paraburkholderia ginsengisoli*
Pseudomonadota	Burkholderiaceae	*Paraburkholderia*	*Paraburkholderia metalliresistens*
Pseudomonadota	Burkholderiaceae	*Paraburkholderia*	*Paraburkholderia oxyphila*
Pseudomonadota	Burkholderiaceae	*Paraburkholderia*	*Paraburkholderia phymatum*
Pseudomonadota	Burkholderiaceae	*Paraburkholderia*	*Paraburkholderia sacchari*
Pseudomonadota	Burkholderiaceae	*Paraburkholderia*	*Paraburkholderia solisilvae*
Pseudomonadota	Burkholderiaceae	*Paraburkholderia*	*Paraburkholderia unamae*
Pseudomonadota	Burkholderiaceae	*Paracandidimonas*	*Paracandidimonas soli*
Pseudomonadota	Rhodobacteraceae	*Paracoccus*	*Paracoccus alimentarius*
Pseudomonadota	Rhodobacteraceae	*Paracoccus*	*Paracoccus denitrificans*
Pseudomonadota	Rhodobacteraceae	*Paracoccus*	*Paracoccus homiensis*
Pseudomonadota	Rhodobacteraceae	*Paracoccus*	*Paracoccus huijuniae*
Pseudomonadota	Rhodobacteraceae	*Paracoccus*	*Paracoccus limosus*
Pseudomonadota	Rhodobacteraceae	*Paracoccus*	*Paracoccus lutimaris*
Pseudomonadota	Rhodobacteraceae	*Paracoccus*	*Paracoccus solventivorans*
Pseudomonadota	Shewanellaceae	*Paraferrimonas*	*Paraferrimonas haliotis*
Pseudomonadota	Shewanellaceae	*Paraferrimonas*	*Paraferrimonas sedimenticola*
Pseudomonadota	Alteromonadaceae	*Paraglaciecola*	*Paraglaciecola aestuariivivens*
Pseudomonadota	Alteromonadaceae	*Paraglaciecola*	*Paraglaciecola agarilytica*
Pseudomonadota	Alteromonadaceae	*Paraglaciecola*	*Paraglaciecola arctica*
Pseudomonadota	Alteromonadaceae	*Paraglaciecola*	*Paraglaciecola hydrolytica*
Pseudomonadota	Alteromonadaceae	*Paraglaciecola*	*Paraglaciecola mesophila*

Pseudomonadota	Alteromonadaceae	*Paraglaciecola*	*Paraglaciecola oceanifecundans*
Pseudomonadota	Moritellaceae	*Paramoritella*	*Paramoritella alkaliphila*
Pseudomonadota	Vibrionaceae	*Paraphotobacterium*	*Paraphotobacterium marinum*
Pseudomonadota	Alcaligenaceae	*Parapusillimonas*	*Parapusillimonas granuli*
Pseudomonadota	Alteromonadaceae	*Pararheinheimera*	*Pararheinheimera aquatica*
Pseudomonadota	Alteromonadaceae	*Pararheinheimera*	*Pararheinheimera mesophila*
Pseudomonadota	Alteromonadaceae	*Pararheinheimera*	*Pararheinheimera texasensis*
Pseudomonadota	Shewanellaceae	*Parashewanella*	*Parashewanella spongiae*
Pseudomonadota	Endozoicomonadaceae	*Parendozoicomonas*	*Parendozoicomonas haliclonae*
Pseudomonadota	Pasteurellaceae	*Pasteurella*	*Pasteurella aerogenes*
Pseudomonadota	Pasteurellaceae	*Pasteurella*	*Pasteurella bettyae*
Pseudomonadota	Pasteurellaceae	*Pasteurella*	*Pasteurella canis*
Pseudomonadota	Pasteurellaceae	*Pasteurella*	*Pasteurella dagmatis*
Pseudomonadota	Pasteurellaceae	*Pasteurella*	*Pasteurella langaaensis*
Pseudomonadota	Pasteurellaceae	*Pasteurella*	*Pasteurella mairii*
Pseudomonadota	Pasteurellaceae	*Pasteurella*	*Pasteurella multocida*
Pseudomonadota	Pasteurellaceae	*Pasteurella*	*Pasteurella multocida;Pasteurella*
Pseudomonadota	Pasteurellaceae	*Pasteurella*	*Pasteurella oralis*
Pseudomonadota	Pasteurellaceae	*Pasteurella*	*Pasteurella skyensis*
Pseudomonadota	Pasteurellaceae	*Pasteurella*	*Pasteurella stomatis*
Pseudomonadota	Pasteurellaceae	*Pasteurella*	*Pasteurella testudinis*
Pseudomonadota	Enterobacteriaceae	*Pectobacterium*	*Pectobacterium actinidiae*
Pseudomonadota	Enterobacteriaceae	*Pectobacterium*	*Pectobacterium aroidearum*
Pseudomonadota	Enterobacteriaceae	*Pectobacterium*	*Pectobacterium atrosepticum*
Pseudomonadota	Enterobacteriaceae	*Pectobacterium*	*Pectobacterium betavasculorum*
Pseudomonadota	Enterobacteriaceae	*Pectobacterium*	*Pectobacterium brasiliense*
Pseudomonadota	Enterobacteriaceae	*Pectobacterium*	*Pectobacterium cacticida*
Pseudomonadota	Enterobacteriaceae	*Pectobacterium*	*Pectobacterium carotovorum*

Pseudomonadota	Enterobacteriaceae	*Pectobacterium*	*Pectobacterium odoriferum*
Pseudomonadota	Enterobacteriaceae	*Pectobacterium*	*Pectobacterium parmentieri*
Pseudomonadota	Enterobacteriaceae	*Pectobacterium*	*Pectobacterium polaris*
Pseudomonadota	Enterobacteriaceae	*Pectobacterium*	*Pectobacterium wasabiae*
Pseudomonadota	Hyphomicrobiaceae	*Pedomicrobium*	*Pedomicrobium americanum*
Pseudomonadota	Hyphomicrobiaceae	*Pedomicrobium*	*Pedomicrobium ferrugineum*
Pseudomonadota	Hyphomicrobiaceae	*Pedomicrobium*	*Pedomicrobium manganicum*
Pseudomonadota	Devosiaceae	*Pelagibacterium*	*Pelagibacterium halotolerans*
Pseudomonadota	Burkholderiaceae	*Pelistega*	*Pelistega indica*
Pseudomonadota	Burkholderiaceae	*Pelistega*	*Pelistega suis*
Pseudomonadota	Burkholderiaceae	*Pelomonas*	*Pelomonas saccharophila*
Pseudomonadota	Moraxellaceae	*Perlucidibaca*	*Perlucidibaca piscinae*
Pseudomonadota	Pseudomonadaceae	*Permianibacter*	*Permianibacter aggregans*
Pseudomonadota	Enterobacteriaceae	*Phaseolibacter*	*Phaseolibacter flectens*
Pseudomonadota	Pasteurellaceae	*Phocoenobacter*	*Phocoenobacter uteri*
Pseudomonadota	Vibrionaceae	*Photobacterium*	*Photobacterium aestuarii*
Pseudomonadota	Vibrionaceae	*Photobacterium*	*Photobacterium angustum*
Pseudomonadota	Vibrionaceae	*Photobacterium*	*Photobacterium aphoticum*
Pseudomonadota	Vibrionaceae	*Photobacterium*	*Photobacterium aplysiae*
Pseudomonadota	Vibrionaceae	*Photobacterium*	*Photobacterium aquimaris*
Pseudomonadota	Vibrionaceae	*Photobacterium*	*Photobacterium atrarenae*
Pseudomonadota	Vibrionaceae	*Photobacterium*	*Photobacterium carnosum*
Pseudomonadota	Vibrionaceae	*Photobacterium*	*Photobacterium chitinilyticum*
Pseudomonadota	Vibrionaceae	*Photobacterium*	*Photobacterium damselae*
Pseudomonadota	Vibrionaceae	*Photobacterium*	*Photobacterium frigidiphilum*
Pseudomonadota	Vibrionaceae	*Photobacterium*	*Photobacterium gaetbulicola*
Pseudomonadota	Vibrionaceae	*Photobacterium*	*Photobacterium galatheae*
Pseudomonadota	Vibrionaceae	*Photobacterium*	*Photobacterium halotolerans*

Pseudomonadota	Vibrionaceae	*Photobacterium*	*Photobacterium iliopiscarium*
Pseudomonadota	Vibrionaceae	*Photobacterium*	*Photobacterium leiognathi*
Pseudomonadota	Vibrionaceae	*Photobacterium*	*Photobacterium lipolyticum*
Pseudomonadota	Vibrionaceae	*Photobacterium*	*Photobacterium panuliri*
Pseudomonadota	Vibrionaceae	*Photobacterium*	*Photobacterium phosphoreum*
Pseudomonadota	Vibrionaceae	*Photobacterium*	*Photobacterium piscicola*
Pseudomonadota	Vibrionaceae	*Photobacterium*	*Photobacterium profundum*
Pseudomonadota	Vibrionaceae	*Photobacterium*	*Photobacterium proteolyticum*
Pseudomonadota	Vibrionaceae	*Photobacterium*	*Photobacterium rosenbergii*
Pseudomonadota	Vibrionaceae	*Photobacterium*	*Photobacterium sanctipauli*
Pseudomonadota	Vibrionaceae	*Photobacterium*	*Photobacterium sanguinicancri*
Pseudomonadota	Enterobacteriaceae	*Photorhabdus*	*Photorhabdus akhurstii*
Pseudomonadota	Enterobacteriaceae	*Photorhabdus*	*Photorhabdus asymbiotica*
Pseudomonadota	Enterobacteriaceae	*Photorhabdus*	*Photorhabdus caribbeanensis*
Pseudomonadota	Enterobacteriaceae	*Photorhabdus*	*Photorhabdus kayaii*
Pseudomonadota	Enterobacteriaceae	*Photorhabdus*	*Photorhabdus khanii*
Pseudomonadota	Enterobacteriaceae	*Photorhabdus*	*Photorhabdus kleinii*
Pseudomonadota	Enterobacteriaceae	*Photorhabdus*	*Photorhabdus luminescens*
Pseudomonadota	Enterobacteriaceae	*Photorhabdus*	*Photorhabdus stackebrandtii*
Pseudomonadota	Enterobacteriaceae	*Photorhabdus*	*Photorhabdus temperata*
Pseudomonadota	Enterobacteriaceae	*Photorhabdus*	*Photorhabdus thracensis*
Pseudomonadota	Phreatobacteraceae	*Phreatobacter*	*Phreatobacter stygius*
Pseudomonadota	Rhizobiaceae	*Phyllobacterium*	*Phyllobacterium bourgognense*
Pseudomonadota	Rhizobiaceae	*Phyllobacterium*	*Phyllobacterium salinisoli*
Pseudomonadota	Enterobacteriaceae	*Phytobacter*	*Phytobacter diazotrophicus*
Pseudomonadota	Enterobacteriaceae	*Phytobacter*	*Phytobacter ursingii*
Pseudomonadota	Alteromonadaceae	*Planctobacterium*	*Planctobacterium marinum*
Pseudomonadota	Competibacteraceae	*Plasticicumulans*	*Plasticicumulans acidivorans*

Pseudomonadota	Competibacteraceae	*Plasticicumulans*	*Plasticicumulans lactativorans*
Pseudomonadota	Pleomorphomonadaceae	*Pleomorphomonas*	*Pleomorphomonas diazotrophica*
Pseudomonadota	Enterobacteriaceae	*Plesiomonas*	*Plesiomonas shigelloides*
Pseudomonadota	Enterobacteriaceae	*Pluralibacter*	*Pluralibacter gergoviae*
Pseudomonadota	Enterobacteriaceae	*Pluralibacter*	*Pluralibacter pyrinus*
Pseudomonadota	Burkholderiaceae	*Polynucleobacter*	*Polynucleobacter asymbioticus*
Pseudomonadota	Erythrobacteraceae	*Pontixanthobacter*	*Pontixanthobacter rizhaonensis*
Pseudomonadota	Enterobacteriaceae	*Pragia*	*Pragia fontium*
Pseudomonadota	Rhodocyclaceae	*Propionivibrio*	*Propionivibrio limicola*
Pseudomonadota	Enterobacteriaceae	*Proteus*	*Proteus cibarius*
Pseudomonadota	Enterobacteriaceae	*Proteus*	*Proteus hauseri*
Pseudomonadota	Enterobacteriaceae	*Proteus*	*Proteus mirabilis*
Pseudomonadota	Enterobacteriaceae	*Proteus*	*Proteus myxofaciens*
Pseudomonadota	Enterobacteriaceae	*Proteus*	*Proteus penneri*
Pseudomonadota	Enterobacteriaceae	*Proteus*	*Proteus terrae*
Pseudomonadota	Enterobacteriaceae	*Proteus*	*Proteus vulgaris*
Pseudomonadota	Enterobacteriaceae	*Providencia*	*Providencia alcalifaciens*
Pseudomonadota	Enterobacteriaceae	*Providencia*	*Providencia burhodogranariea*
Pseudomonadota	Enterobacteriaceae	*Providencia*	*Providencia heimbachae*
Pseudomonadota	Enterobacteriaceae	*Providencia*	*Providencia rettgeri*
Pseudomonadota	Enterobacteriaceae	*Providencia*	*Providencia rustigianii*
Pseudomonadota	Enterobacteriaceae	*Providencia*	*Providencia sneebia*
Pseudomonadota	Enterobacteriaceae	*Providencia*	*Providencia stuartii*
Pseudomonadota	Enterobacteriaceae	*Providencia*	*Providencia thailandensis*
Pseudomonadota	Enterobacteriaceae	*Providencia*	*Providencia vermicola*
Pseudomonadota	Enterobacteriaceae	*Pseudescherichia*	*Pseudescherichia vulneris*
Pseudomonadota	Idiomarinaceae	*Pseudidiomarina*	*Pseudidiomarina aquimaris*
Pseudomonadota	Idiomarinaceae	*Pseudidiomarina*	*Pseudidiomarina atlantica*

Pseudomonadota	Idiomarinaceae	*Pseudidiomarina*	*Pseudidiomarina donghaiensis*
Pseudomonadota	Idiomarinaceae	*Pseudidiomarina*	*Pseudidiomarina indica*
Pseudomonadota	Idiomarinaceae	*Pseudidiomarina*	*Pseudidiomarina maritima*
Pseudomonadota	Alteromonadaceae	*Pseudoalteromonas*	*Pseudoalteromonas aestuariivivens*
Pseudomonadota	Alteromonadaceae	*Pseudoalteromonas*	*Pseudoalteromonas arctica*
Pseudomonadota	Alteromonadaceae	*Pseudoalteromonas*	*Pseudoalteromonas carrageenovora*
Pseudomonadota	Alteromonadaceae	*Pseudoalteromonas*	*Pseudoalteromonas haloplanktis*
Pseudomonadota	Alteromonadaceae	*Pseudoalteromonas*	*Pseudoalteromonas lipolytica*
Pseudomonadota	Alteromonadaceae	*Pseudoalteromonas*	*Pseudoalteromonas luteoviolacea*
Pseudomonadota	Alteromonadaceae	*Pseudoalteromonas*	*Pseudoalteromonas nigrifaciens*
Pseudomonadota	Alteromonadaceae	*Pseudoalteromonas*	*Pseudoalteromonas prydzensis*
Pseudomonadota	Alteromonadaceae	*Pseudoalteromonas*	*Pseudoalteromonas ruthenica*
Pseudomonadota	Alteromonadaceae	*Pseudoalteromonas*	*Pseudoalteromonas shioyasakiensis*
Pseudomonadota	Alteromonadaceae	*Pseudoalteromonas*	*Pseudoalteromonas spongiae*
Pseudomonadota	Alteromonadaceae	*Pseudoalteromonas*	*Pseudoalteromonas xiamenensis*
Pseudomonadota	Alteromonadaceae	*Pseudobowmanella*	*Pseudobowmanella zhangzhouensis*
Pseudomonadota	Enterobacteriaceae	*Pseudocitrobacter*	*Pseudocitrobacter anthropi*
Pseudomonadota	Enterobacteriaceae	*Pseudocitrobacter*	*Pseudocitrobacter faecalis*
Pseudomonadota	Rhodobacteraceae	*Pseudogemmobacter*	*Pseudogemmobacter bohemicus*
Pseudomonadota	Pseudohongiellaceae	*Pseudohongiella*	*Pseudohongiella nitratireducens*
Pseudomonadota	Xanthobacteraceae	*Pseudolabrys*	*Pseudolabrys taiwanensis*
Pseudomonadota	Cellvibrionaceae	*Pseudomaricurvus*	*Pseudomaricurvus alcaniphilus*
Pseudomonadota	Pseudomonadaceae	*Pseudomonas*	*Pseudomonas aeruginosa*
Pseudomonadota	Pseudomonadaceae	*Pseudomonas*	*Pseudomonas aestus*
Pseudomonadota	Pseudomonadaceae	*Pseudomonas*	*Pseudomonas alcaligenes*
Pseudomonadota	Pseudomonadaceae	*Pseudomonas*	*Pseudomonas argentinensis*
Pseudomonadota	Pseudomonadaceae	*Pseudomonas*	*Pseudomonas azotifigens*
Pseudomonadota	Pseudomonadaceae	*Pseudomonas*	*Pseudomonas bohemica*

Pseudomonadota	Pseudomonadaceae	*Pseudomonas*	*Pseudomonas borbori*
Pseudomonadota	Pseudomonadaceae	*Pseudomonas*	*Pseudomonas cannabina*
Pseudomonadota	Pseudomonadaceae	*Pseudomonas*	*Pseudomonas carboxydohydrogena*
Pseudomonadota	Pseudomonadaceae	*Pseudomonas*	*Pseudomonas cedrina*
Pseudomonadota	Pseudomonadaceae	*Pseudomonas*	*Pseudomonas chlororaphis*
Pseudomonadota	Pseudomonadaceae	*Pseudomonas*	*Pseudomonas citronellolis*
Pseudomonadota	Pseudomonadaceae	*Pseudomonas*	*Pseudomonas delhiensis*
Pseudomonadota	Pseudomonadaceae	*Pseudomonas*	*Pseudomonas donghuensis*
Pseudomonadota	Pseudomonadaceae	*Pseudomonas*	*Pseudomonas duriflava*
Pseudomonadota	Pseudomonadaceae	*Pseudomonas*	*Pseudomonas entomophila*
Pseudomonadota	Pseudomonadaceae	*Pseudomonas*	*Pseudomonas flavescens*
Pseudomonadota	Pseudomonadaceae	*Pseudomonas*	*Pseudomonas flexibilis*
Pseudomonadota	Pseudomonadaceae	*Pseudomonas*	*Pseudomonas fragi*
Pseudomonadota	Pseudomonadaceae	*Pseudomonas*	*Pseudomonas frederiksbergensis*
Pseudomonadota	Pseudomonadaceae	*Pseudomonas*	*Pseudomonas gessardii*
Pseudomonadota	Pseudomonadaceae	*Pseudomonas*	*Pseudomonas glareae*
Pseudomonadota	Pseudomonadaceae	*Pseudomonas*	*Pseudomonas guariconensis*
Pseudomonadota	Pseudomonadaceae	*Pseudomonas*	*Pseudomonas helleri*
Pseudomonadota	Pseudomonadaceae	*Pseudomonas*	*Pseudomonas helmanticensis*
Pseudomonadota	Pseudomonadaceae	*Pseudomonas*	*Pseudomonas hussainii*
Pseudomonadota	Pseudomonadaceae	*Pseudomonas*	*Pseudomonas indica*
Pseudomonadota	Pseudomonadaceae	*Pseudomonas*	*Pseudomonas jinjuensis*
Pseudomonadota	Pseudomonadaceae	*Pseudomonas*	*Pseudomonas knackmussii*
Pseudomonadota	Pseudomonadaceae	*Pseudomonas*	*Pseudomonas kunmingensis*
Pseudomonadota	Pseudomonadaceae	*Pseudomonas*	*Pseudomonas marincola*
Pseudomonadota	Pseudomonadaceae	*Pseudomonas*	*Pseudomonas matsuisoli*
Pseudomonadota	Pseudomonadaceae	*Pseudomonas*	*Pseudomonas migulae*
Pseudomonadota	Pseudomonadaceae	*Pseudomonas*	*Pseudomonas monteilii*

Pseudomonadota	Pseudomonadaceae	*Pseudomonas*	*Pseudomonas moraviensis*
Pseudomonadota	Pseudomonadaceae	*Pseudomonas*	*Pseudomonas mosselii*
Pseudomonadota	Pseudomonadaceae	*Pseudomonas*	*Pseudomonas mucidolens*
Pseudomonadota	Pseudomonadaceae	*Pseudomonas*	*Pseudomonas multiresinivorans*
Pseudomonadota	Pseudomonadaceae	*Pseudomonas*	*Pseudomonas nitritireducens*
Pseudomonadota	Pseudomonadaceae	*Pseudomonas*	*Pseudomonas nitroreducens*
Pseudomonadota	Pseudomonadaceae	*Pseudomonas*	*Pseudomonas oceani*
Pseudomonadota	Pseudomonadaceae	*Pseudomonas*	*Pseudomonas oleovorans*
Pseudomonadota	Pseudomonadaceae	*Pseudomonas*	*Pseudomonas oryzihabitans*
Pseudomonadota	Pseudomonadaceae	*Pseudomonas*	*Pseudomonas otitidis*
Pseudomonadota	Pseudomonadaceae	*Pseudomonas*	*Pseudomonas panacis*
Pseudomonadota	Pseudomonadaceae	*Pseudomonas*	*Pseudomonas panipatensis*
Pseudomonadota	Pseudomonadaceae	*Pseudomonas*	*Pseudomonas parafulva*
Pseudomonadota	Pseudomonadaceae	*Pseudomonas*	*Pseudomonas peli*
Pseudomonadota	Pseudomonadaceae	*Pseudomonas*	*Pseudomonas plecoglossicida*
Pseudomonadota	Pseudomonadaceae	*Pseudomonas*	*Pseudomonas populi*
Pseudomonadota	Pseudomonadaceae	*Pseudomonas*	*Pseudomonas psychrophila*
Pseudomonadota	Pseudomonadaceae	*Pseudomonas*	*Pseudomonas punonensis*
Pseudomonadota	Pseudomonadaceae	*Pseudomonas*	*Pseudomonas reidholzensis*
Pseudomonadota	Pseudomonadaceae	*Pseudomonas*	*Pseudomonas reinekei*
Pseudomonadota	Pseudomonadaceae	*Pseudomonas*	*Pseudomonas resinovorans*
Pseudomonadota	Pseudomonadaceae	*Pseudomonas*	*Pseudomonas silesiensis*
Pseudomonadota	Pseudomonadaceae	*Pseudomonas*	*Pseudomonas soli*
Pseudomonadota	Pseudomonadaceae	*Pseudomonas*	*Pseudomonas taiwanensis*
Pseudomonadota	Pseudomonadaceae	*Pseudomonas*	*Pseudomonas tarimensis*
Pseudomonadota	Pseudomonadaceae	*Pseudomonas*	*Pseudomonas tolaasii*
Pseudomonadota	Pseudomonadaceae	*Pseudomonas*	*Pseudomonas urumqiensis*
Pseudomonadota	Pseudomonadaceae	*Pseudomonas*	*Pseudomonas vranovensis*

Pseudomonadota	Rhizobiaceae	*Pseudorhizobium*	*Pseudorhizobium endolithicum*
Pseudomonadota	Xanthobacteraceae	*Pseudorhodoplanes*	*Pseudorhodoplanes sinuspersici*
Pseudomonadota	Cellvibrionaceae	*Pseudoteredinibacter*	*Pseudoteredinibacter isoporae*
Pseudomonadota	Stappiaceae	*Pseudovibrio*	*Pseudovibrio stylochi*
Pseudomonadota	Xanthomonadaceae	*Pseudoxanthomonas*	*Pseudoxanthomonas icgebensis*
Pseudomonadota	Xanthomonadaceae	*Pseudoxanthomonas*	*Pseudoxanthomonas japonensis*
Pseudomonadota	Xanthomonadaceae	*Pseudoxanthomonas*	*Pseudoxanthomonas suwonensis*
Pseudomonadota	Moraxellaceae	*Psychrobacter*	*Psychrobacter fulvigenes*
Pseudomonadota	Moraxellaceae	*Psychrobacter*	*Psychrobacter maritimus*
Pseudomonadota	Moraxellaceae	*Psychrobacter*	*Psychrobacter pacificensis*
Pseudomonadota	Moraxellaceae	*Psychrobacter*	*Psychrobacter pasteurii*
Pseudomonadota	Psychrobiaceae	*Psychrobium*	*Psychrobium conchae*
Pseudomonadota	Psychromonadaceae	*Psychromonas*	*Psychromonas aquatilis*
Pseudomonadota	Psychromonadaceae	*Psychromonas*	*Psychromonas heitensis*
Pseudomonadota	Psychromonadaceae	*Psychromonas*	*Psychromonas ingrahamii*
Pseudomonadota	Psychromonadaceae	*Psychromonas*	*Psychromonas macrocephali*
Pseudomonadota	Psychromonadaceae	*Psychromonas*	*Psychromonas profunda*
Pseudomonadota	Alteromonadaceae	*Psychrosphaera*	*Psychrosphaera aestuarii*
Pseudomonadota	Alteromonadaceae	*Psychrosphaera*	*Psychrosphaera aquimarina*
Pseudomonadota	Alteromonadaceae	*Psychrosphaera*	*Psychrosphaera saromensis*
Pseudomonadota	Burkholderiaceae	*Pusillimonas*	*Pusillimonas ginsengisoli*
Pseudomonadota	Burkholderiaceae	*Pusillimonas*	*Pusillimonas noertemannii*
Pseudomonadota	Burkholderiaceae	*Pusillimonas*	*Pusillimonas soli*
Pseudomonadota	Burkholderiaceae	*Pusillimonas*	*Pusillimonas thiosulfatoxidans*
Pseudomonadota	Enterobacteriaceae	*Rahnella*	*Rahnella aquatilis*
Pseudomonadota	Enterobacteriaceae	*Rahnella*	*Rahnella inusitata*
Pseudomonadota	Burkholderiaceae	*Ramlibacter*	*Ramlibacter solisilvae*
Pseudomonadota	Enterobacteriaceae	*Raoultella*	*Raoultella electrica*

Pseudomonadota	Enterobacteriaceae	*Raoultella*	*Raoultella ornithinolytica*
Pseudomonadota	Enterobacteriaceae	*Raoultella*	*Raoultella planticola*
Pseudomonadota	Enterobacteriaceae	*Raoultella*	*Raoultella terrigena*
Pseudomonadota	Reyranellaceae	*Reyranella*	*Reyranella graminifolii*
Pseudomonadota	Reyranellaceae	*Reyranella*	*Reyranella massiliensis*
Pseudomonadota	Reyranellaceae	*Reyranella*	*Reyranella soli*
Pseudomonadota	Reyranellaceae	*Reyranella*	*Reyranella terrae*
Pseudomonadota	Alteromonadaceae	*Rheinheimera*	*Rheinheimera aquimaris*
Pseudomonadota	Alteromonadaceae	*Rheinheimera*	*Rheinheimera hassiensis*
Pseudomonadota	Alteromonadaceae	*Rheinheimera*	*Rheinheimera pacifica*
Pseudomonadota	Alteromonadaceae	*Rheinheimera*	*Rheinheimera perlucida*
Pseudomonadota	Alteromonadaceae	*Rheinheimera*	*Rheinheimera riviphila*
Pseudomonadota	Alteromonadaceae	*Rheinheimera*	*Rheinheimera salexigens*
Pseudomonadota	Alteromonadaceae	*Rheinheimera*	*Rheinheimera sediminis*
Pseudomonadota	Alteromonadaceae	*Rheinheimera*	*Rheinheimera tuosuensis*
Pseudomonadota	Burkholderiaceae	*Rhizobacter*	*Rhizobacter dauci*
Pseudomonadota	Rhizobiaceae	*Rhizobium*	*Rhizobium acidisoli*
Pseudomonadota	Rhizobiaceae	*Rhizobium*	*Rhizobium aegyptiacum*
Pseudomonadota	Rhizobiaceae	*Rhizobium*	*Rhizobium aethiopicum*
Pseudomonadota	Rhizobiaceae	*Rhizobium*	*Rhizobium alamii*
Pseudomonadota	Rhizobiaceae	*Rhizobium*	*Rhizobium altiplani*
Pseudomonadota	Rhizobiaceae	*Rhizobium*	*Rhizobium azibense*
Pseudomonadota	Rhizobiaceae	*Rhizobium*	*Rhizobium capsici*
Pseudomonadota	Rhizobiaceae	*Rhizobium*	*Rhizobium cauense*
Pseudomonadota	Rhizobiaceae	*Rhizobium*	*Rhizobium cellulosilyticum*
Pseudomonadota	Rhizobiaceae	*Rhizobium*	*Rhizobium etli*
Pseudomonadota	Rhizobiaceae	*Rhizobium*	*Rhizobium gallicum*
Pseudomonadota	Rhizobiaceae	*Rhizobium*	*Rhizobium giardinii*

Pseudomonadota	Rhizobiaceae	*Rhizobium*	*Rhizobium halotolerans*
Pseudomonadota	Rhizobiaceae	*Rhizobium*	*Rhizobium jaguaris*
Pseudomonadota	Rhizobiaceae	*Rhizobium*	*Rhizobium kunmingense*
Pseudomonadota	Rhizobiaceae	*Rhizobium*	*Rhizobium leguminosarum*
Pseudomonadota	Rhizobiaceae	*Rhizobium*	*Rhizobium lusitanum*
Pseudomonadota	Rhizobiaceae	*Rhizobium*	*Rhizobium mesosinicum*
Pseudomonadota	Rhizobiaceae	*Rhizobium*	*Rhizobium naphthalenivorans*
Pseudomonadota	Rhizobiaceae	*Rhizobium*	*Rhizobium petrolearium*
Pseudomonadota	Rhizobiaceae	*Rhizobium*	*Rhizobium phaseoli*
Pseudomonadota	Rhizobiaceae	*Rhizobium*	*Rhizobium populi*
Pseudomonadota	Rhizobiaceae	*Rhizobium*	*Rhizobium pseudoryzae*
Pseudomonadota	Rhizobiaceae	*Rhizobium*	*Rhizobium tropici*
Pseudomonadota	Rhizobiaceae	*Rhizobium*	*Rhizobium undicola*
Pseudomonadota	Rhizobiaceae	*Rhizobium*	*Rhizobium viscosum*
Pseudomonadota	Micropepsaceae	*Rhizomicrobium*	*Rhizomicrobium palustre*
Pseudomonadota	Rhodanobacteraceae	*Rhodanobacter*	*Rhodanobacter denitrificans*
Pseudomonadota	Rhodanobacteraceae	*Rhodanobacter*	*Rhodanobacter terrae*
Pseudomonadota	Rhodanobacteraceae	*Rhodanobacter*	*Rhodanobacter umsongensis*
Pseudomonadota	Rhodobacteraceae	*Rhodobacter*	*Rhodobacter blasticus*
Pseudomonadota	Rhodobacteraceae	*Rhodobacter*	*Rhodobacter capsulatus*
Pseudomonadota	Rhodobacteraceae	*Rhodobacter*	*Rhodobacter sediminis*
Pseudomonadota	Beijerinckiaceae	*Rhodoblastus*	*Rhodoblastus acidophilus*
Pseudomonadota	Beijerinckiaceae	*Rhodoblastus*	*Rhodoblastus sphagnicola*
Pseudomonadota	Parvibaculaceae	*Rhodoligotrophos*	*Rhodoligotrophos appendicifer*
Pseudomonadota	Parvibaculaceae	*Rhodoligotrophos*	*Rhodoligotrophos jinshengii*
Pseudomonadota	Rhodomicrobiaceae	*Rhodomicrobium*	*Rhodomicrobium lacus*
Pseudomonadota	Rhodomicrobiaceae	*Rhodomicrobium*	*Rhodomicrobium vannielii*
Pseudomonadota	Acetobacteraceae	*Rhodopila*	*Rhodopila globiformis*

Pseudomonadota	Xanthobacteraceae	*Rhodoplanes*	*Rhodoplanes azumiensis*
Pseudomonadota	Xanthobacteraceae	*Rhodoplanes*	*Rhodoplanes piscinae*
Pseudomonadota	Xanthobacteraceae	*Rhodoplanes*	*Rhodoplanes roseus*
Pseudomonadota	Xanthobacteraceae	*Rhodoplanes*	*Rhodoplanes tepidicaeni*
Pseudomonadota	Xanthobacteraceae	*Rhodopseudomonas*	*Rhodopseudomonas boonkerdii*
Pseudomonadota	Xanthobacteraceae	*Rhodopseudomonas*	*Rhodopseudomonas faecalis*
Pseudomonadota	Acetobacteraceae	*Rhodovastum*	*Rhodovastum atsumiense*
Pseudomonadota	Rhodovibrionaceae	*Rhodovibrio*	*Rhodovibrio salinarum*
Pseudomonadota	Rickettsiaceae	*Rickettsia*	*Rickettsia bellii*
Pseudomonadota	Rickettsiaceae	*Rickettsia*	*Rickettsia conorii*
Pseudomonadota	Rickettsiaceae	*Rickettsia*	*Rickettsia honei*
Pseudomonadota	Rickettsiaceae	*Rickettsia*	*Rickettsia hulinii*
Pseudomonadota	Rickettsiaceae	*Rickettsia*	*Rickettsia parkeri*
Pseudomonadota	Burkholderiaceae	*Rivibacter*	*Rivibacter subsaxonicus*
Pseudomonadota	Pasteurellaceae	*Rodentibacter*	*Rodentibacter heylii*
Pseudomonadota	Pasteurellaceae	*Rodentibacter*	*Rodentibacter myodis*
Pseudomonadota	Pasteurellaceae	*Rodentibacter*	*Rodentibacter pneumotropicus*
Pseudomonadota	Pasteurellaceae	*Rodentibacter*	*Rodentibacter rarus*
Pseudomonadota	Pasteurellaceae	*Rodentibacter*	*Rodentibacter ratti*
Pseudomonadota	Beijerinckiaceae	*Roseiarcus*	*Roseiarcus fermentans*
Pseudomonadota	Acetobacteraceae	*Roseicella*	*Roseicella frigidaeris*
Pseudomonadota	Enterobacteriaceae	*Rosenbergiella*	*Rosenbergiella australiborealis*
Pseudomonadota	Enterobacteriaceae	*Rosenbergiella*	*Rosenbergiella collisarenosi*
Pseudomonadota	Enterobacteriaceae	*Rosenbergiella*	*Rosenbergiella epipactidis*
Pseudomonadota	Enterobacteriaceae	*Rosenbergiella*	*Rosenbergiella nectarea*
Pseudomonadota	Acetobacteraceae	*Roseomonas*	*Roseomonas aeriglobus*
Pseudomonadota	Acetobacteraceae	*Roseomonas*	*Roseomonas aerofrigidensis*
Pseudomonadota	Acetobacteraceae	*Roseomonas*	*Roseomonas gilardii*

Pseudomonadota	Acetobacteraceae	*Roseomonas*	*Roseomonas sediminicola*
Pseudomonadota	Acetobacteraceae	*Roseomonas*	*Roseomonas terrae*
Pseudomonadota	Acetobacteraceae	*Roseomonas*	*Roseomonas terricola*
Pseudomonadota	Acetobacteraceae	*Roseomonas*	*Roseomonas wooponensis*
Pseudomonadota	Enterobacteriaceae	*Rouxiella*	*Rouxiella chamberiensis*
Pseudomonadota	Enterobacteriaceae	*Rouxiella*	*Rouxiella silvae*
Pseudomonadota	Rhodobacteraceae	*Rubellimicrobium*	*Rubellimicrobium mesophilum*
Pseudomonadota	Rhodobacteraceae	*Rubellimicrobium*	*Rubellimicrobium roseum*
Pseudomonadota	Burkholderiaceae	*Rugamonas*	*Rugamonas rubra*
Pseudomonadota	Succinivibrionaceae	*Ruminobacter*	*Ruminobacter amylophilus*
Pseudomonadota	Acetobacteraceae	*Saccharibacter*	*Saccharibacter floricola*
Pseudomonadota	Halomonadaceae	*Salicola*	*Salicola marasensis*
Pseudomonadota	Halomonadaceae	*Salinicola*	*Salinicola acroporae*
Pseudomonadota	Halomonadaceae	*Salinicola*	*Salinicola halophilus*
Pseudomonadota	Vibrionaceae	*Salinivibrio*	*Salinivibrio proteolyticus*
Pseudomonadota	Vibrionaceae	*Salinivibrio*	*Salinivibrio siamensis*
Pseudomonadota	Enterobacteriaceae	*Salmonella*	*Salmonella bongori*
Pseudomonadota	Enterobacteriaceae	*Salmonella*	*Salmonella enterica*
Pseudomonadota	Pseudoalteromonadaceae	*Salsuginimonas*	*Salsuginimonas clara*
Pseudomonadota	Enterobacteriaceae	*Samsonia*	*Samsonia erythrinae*
Pseudomonadota	Sedimenticolaceae	*Sedimenticola*	*Sedimenticola selenatireducens*
Pseudomonadota	Enterobacteriaceae	*Serratia*	*Serratia aquatilis*
Pseudomonadota	Enterobacteriaceae	*Serratia*	*Serratia bozhouensis*
Pseudomonadota	Enterobacteriaceae	*Serratia*	*Serratia entomophila*
Pseudomonadota	Enterobacteriaceae	*Serratia*	*Serratia ficaria*
Pseudomonadota	Enterobacteriaceae	*Serratia*	*Serratia fonticola*
Pseudomonadota	Enterobacteriaceae	*Serratia*	*Serratia grimesii*
Pseudomonadota	Enterobacteriaceae	*Serratia*	*Serratia liquefaciens*

Pseudomonadota	Enterobacteriaceae	*Serratia*	*Serratia marcescens*
Pseudomonadota	Enterobacteriaceae	*Serratia*	*Serratia microhaemolytica*
Pseudomonadota	Enterobacteriaceae	*Serratia*	*Serratia nematodiphila*
Pseudomonadota	Enterobacteriaceae	*Serratia*	*Serratia odorifera*
Pseudomonadota	Enterobacteriaceae	*Serratia*	*Serratia oryzae*
Pseudomonadota	Enterobacteriaceae	*Serratia*	*Serratia plymuthica*
Pseudomonadota	Enterobacteriaceae	*Serratia*	*Serratia proteamaculans*
Pseudomonadota	Enterobacteriaceae	*Serratia*	*Serratia quinivorans*
Pseudomonadota	Enterobacteriaceae	*Serratia*	*Serratia rubidaea*
Pseudomonadota	Enterobacteriaceae	*Serratia*	*Serratia surfactantfaciens*
Pseudomonadota	Enterobacteriaceae	*Serratia*	*Serratia symbiotica*
Pseudomonadota	Enterobacteriaceae	*Serratia*	*Serratia ureilytica*
Pseudomonadota	Shewanellaceae	*Shewanella*	*Shewanella algae*
Pseudomonadota	Shewanellaceae	*Shewanella*	*Shewanella algidipiscicola*
Pseudomonadota	Shewanellaceae	*Shewanella*	*Shewanella amazonensis*
Pseudomonadota	Shewanellaceae	*Shewanella*	*Shewanella carassii*
Pseudomonadota	Shewanellaceae	*Shewanella*	*Shewanella decolorationis*
Pseudomonadota	Shewanellaceae	*Shewanella*	*Shewanella dokdonensis*
Pseudomonadota	Shewanellaceae	*Shewanella*	*Shewanella donghaensis*
Pseudomonadota	Shewanellaceae	*Shewanella*	*Shewanella fodinae*
Pseudomonadota	Shewanellaceae	*Shewanella*	*Shewanella gelidii*
Pseudomonadota	Shewanellaceae	*Shewanella*	*Shewanella indica*
Pseudomonadota	Shewanellaceae	*Shewanella*	*Shewanella intestini*
Pseudomonadota	Shewanellaceae	*Shewanella*	*Shewanella irciniae*
Pseudomonadota	Shewanellaceae	*Shewanella*	*Shewanella japonica*
Pseudomonadota	Shewanellaceae	*Shewanella*	*Shewanella khirikhana*
Pseudomonadota	Shewanellaceae	*Shewanella*	*Shewanella litorisediminis*
Pseudomonadota	Shewanellaceae	*Shewanella*	*Shewanella livingstonensis*

Pseudomonadota	Shewanellaceae	*Shewanella*	*Shewanella loihica*
Pseudomonadota	Shewanellaceae	*Shewanella*	*Shewanella mangrovi*
Pseudomonadota	Shewanellaceae	*Shewanella*	*Shewanella marina*
Pseudomonadota	Shewanellaceae	*Shewanella*	*Shewanella marisflavi*
Pseudomonadota	Shewanellaceae	*Shewanella*	*Shewanella oneidensis*
Pseudomonadota	Shewanellaceae	*Shewanella*	*Shewanella seohaensis*
Pseudomonadota	Shewanellaceae	*Shewanella*	*Shewanella surugensis*
Pseudomonadota	Shewanellaceae	*Shewanella*	*Shewanella violacea*
Pseudomonadota	Shewanellaceae	*Shewanella*	*Shewanella waksmanii*
Pseudomonadota	Shewanellaceae	*Shewanella*	*Shewanella woodyi*
Pseudomonadota	Enterobacteriaceae	*Shigella*	*Shigella boydii*
Pseudomonadota	Enterobacteriaceae	*Shigella*	*Shigella dysenteriae*
Pseudomonadota	Enterobacteriaceae	*Shigella*	*Shigella flexneri*
Pseudomonadota	Enterobacteriaceae	*Shigella*	*Shigella sonnei*
Pseudomonadota	Enterobacteriaceae	*Shimwellia*	*Shimwellia blattae*
Pseudomonadota	Enterobacteriaceae	*Shimwellia*	*Shimwellia pseudoproteus*
Pseudomonadota	Rhizobiaceae	*Shinella*	*Shinella curvata*
Pseudomonadota	Rhizobiaceae	*Shinella*	*Shinella granuli*
Pseudomonadota	Rhizobiaceae	*Shinella*	*Shinella kummerowiae*
Pseudomonadota	Rhizobiaceae	*Shinella*	*Shinella yambaruensis*
Pseudomonadota	Rhizobiaceae	*Shinella*	*Shinella zoogloeoides*
Pseudomonadota	Enterobacteriaceae	*Siccibacter*	*Siccibacter colletis*
Pseudomonadota	Enterobacteriaceae	*Siccibacter*	*Siccibacter turicensis*
Pseudomonadota	Cellvibrionaceae	*Simiduia*	*Simiduia agarivorans*
Pseudomonadota	Rhizobiaceae	*Sinorhizobium*	*Sinorhizobium kostiense*
Pseudomonadota	Rhizobiaceae	*Sinorhizobium*	*Sinorhizobium saheli*
Pseudomonadota	Azospirillaceae	*Skermanella*	*Skermanella rosea*
Pseudomonadota	Sneathiellaceae	*Sneathiella*	*Sneathiella chinensis*

Pseudomonadota	Neisseriaceae	*Snodgrassella*	*Snodgrassella alvi*
Pseudomonadota	Sphingomonadaceae	*Sphingobium*	*Sphingobium aromaticiconvertens*
Pseudomonadota	Sphingomonadaceae	*Sphingobium*	*Sphingobium chinhatense*
Pseudomonadota	Sphingomonadaceae	*Sphingobium*	*Sphingobium fluviale*
Pseudomonadota	Sphingomonadaceae	*Sphingobium*	*Sphingobium fontiphilum*
Pseudomonadota	Sphingomonadaceae	*Sphingobium*	*Sphingobium phenoxybenzoativorans*
Pseudomonadota	Sphingomonadaceae	*Sphingomonas*	*Sphingomonas changbaiensis*
Pseudomonadota	Sphingomonadaceae	*Sphingomonas*	*Sphingomonas colocasiae*
Pseudomonadota	Sphingomonadaceae	*Sphingomonas*	*Sphingomonas crusticola*
Pseudomonadota	Sphingomonadaceae	*Sphingomonas*	*Sphingomonas haloaromaticamans*
Pseudomonadota	Sphingomonadaceae	*Sphingomonas*	*Sphingomonas japonica*
Pseudomonadota	Sphingomonadaceae	*Sphingomonas*	*Sphingomonas jatrophae*
Pseudomonadota	Sphingomonadaceae	*Sphingomonas*	*Sphingomonas kyeonggiensis*
Pseudomonadota	Sphingomonadaceae	*Sphingomonas*	*Sphingomonas laterariae*
Pseudomonadota	Sphingomonadaceae	*Sphingomonas*	*Sphingomonas lutea*
Pseudomonadota	Sphingomonadaceae	*Sphingomonas*	*Sphingomonas mali*
Pseudomonadota	Sphingomonadaceae	*Sphingomonas*	*Sphingomonas metalli*
Pseudomonadota	Sphingomonadaceae	*Sphingomonas*	*Sphingomonas mucosissima*
Pseudomonadota	Sphingomonadaceae	*Sphingomonas*	*Sphingomonas naasensis*
Pseudomonadota	Sphingomonadaceae	*Sphingomonas*	*Sphingomonas panacis*
Pseudomonadota	Sphingomonadaceae	*Sphingomonas*	*Sphingomonas parapaucimobilis*
Pseudomonadota	Sphingomonadaceae	*Sphingomonas*	*Sphingomonas roseiflava*
Pseudomonadota	Sphingomonadaceae	*Sphingomonas*	*Sphingomonas rubra*
Pseudomonadota	Sphingomonadaceae	*Sphingomonas*	*Sphingomonas yabuuchiae*
Pseudomonadota	Sphingomonadaceae	*Sphingomonas*	*Sphingomonas zeicaulis*
Pseudomonadota	Sphingomonadaceae	*Sphingopyxis*	*Sphingopyxis solisilvae*
Pseudomonadota	Sphingomonadaceae	*Sphingorhabdus*	*Sphingorhabdus buctiana*
Pseudomonadota	Sphingomonadaceae	*Sphingorhabdus*	*Sphingorhabdus contaminans*

Pseudomonadota	Sphingomonadaceae	*Sphingosinicella*	*Sphingosinicella soli*
Pseudomonadota	Nitrococcaceae	*Spiribacter*	*Spiribacter salinus*
Pseudomonadota	Stellaceae	*Stella*	*Stella humosa*
Pseudomonadota	Stellaceae	*Stella*	*Stella vacuolata*
Pseudomonadota	Xanthomonadaceae	*Stenotrophomonas*	*Stenotrophomonas maltophilia*
Pseudomonadota	Xanthomonadaceae	*Stenotrophomonas*	*Stenotrophomonas pavanii*
Pseudomonadota	Xanthomonadaceae	*Stenotrophomonas*	*Stenotrophomonas rhizophila*
Pseudomonadota	Steroidobacteraceae	*Steroidobacter*	*Steroidobacter denitrificans*
Pseudomonadota	Sulfuricellaceae	*Sulfuricella*	*Sulfuricella denitrificans*
Pseudomonadota	Sulfuriferulaceae	*Sulfuriferula*	*Sulfuriferula thiophila*
Pseudomonadota	Granulosicoccaceae	*Sulfuriflexus*	*Sulfuriflexus mobilis*
Pseudomonadota	Burkholderiaceae	*Sutterella*	*Sutterella massiliensis*
Pseudomonadota	Burkholderiaceae	*Sutterella*	*Sutterella stercoricanis*
Pseudomonadota	Rhodanobacteraceae	*Tahibacter*	*Tahibacter aquaticus*
Pseudomonadota	Enterobacteriaceae	*Tatumella*	*Tatumella citrea*
Pseudomonadota	Enterobacteriaceae	*Tatumella*	*Tatumella morbirosei*
Pseudomonadota	Enterobacteriaceae	*Tatumella*	*Tatumella ptyseos*
Pseudomonadota	Enterobacteriaceae	*Tatumella*	*Tatumella punctata*
Pseudomonadota	Enterobacteriaceae	*Tatumella*	*Tatumella saanichensis*
Pseudomonadota	Enterobacteriaceae	*Tatumella*	*Tatumella terrea*
Pseudomonadota	Burkholderiaceae	*Taylorella*	*Taylorella asinigenitalis*
Pseudomonadota	Burkholderiaceae	*Taylorella*	*Taylorella equigenitalis*
Pseudomonadota	Temperatibacteraceae	*Temperatibacter*	*Temperatibacter marinus*
Pseudomonadota	Tepidamorphaceae	*Tepidamorphus*	*Tepidamorphus gemmatus*
Pseudomonadota	Alteromonadaceae	*Thalassomonas*	*Thalassomonas actiniarum*
Pseudomonadota	Alteromonadaceae	*Thalassomonas*	*Thalassomonas haliotis*
Pseudomonadota	Alteromonadaceae	*Thalassomonas*	*Thalassomonas viridans*
Pseudomonadota	Thalassospiraceae	*Thalassospira*	*Thalassospira profundimaris*

Pseudomonadota	Alteromonadaceae	*Thalassotalea*	*Thalassotalea agariperforans*
Pseudomonadota	Alteromonadaceae	*Thalassotalea*	*Thalassotalea atypica*
Pseudomonadota	Alteromonadaceae	*Thalassotalea*	*Thalassotalea fusca*
Pseudomonadota	Alteromonadaceae	*Thalassotalea*	*Thalassotalea ganghwensis*
Pseudomonadota	Alteromonadaceae	*Thalassotalea*	*Thalassotalea litorea*
Pseudomonadota	Alteromonadaceae	*Thalassotalea*	*Thalassotalea piscium*
Pseudomonadota	Alteromonadaceae	*Thalassotalea*	*Thalassotalea ponticola*
Pseudomonadota	Alteromonadaceae	*Thalassotalea*	*Thalassotalea profundi*
Pseudomonadota	Rhodocyclaceae	*Thauera*	*Thauera linaloolentis*
Pseudomonadota	Rhodocyclaceae	*Thauera*	*Thauera phenylacetica*
Pseudomonadota	Rhodocyclaceae	*Thauera*	*Thauera sinica*
Pseudomonadota	Vibrionaceae	*Thaumasiovibrio*	*Thaumasiovibrio occultus*
Pseudomonadota	Vibrionaceae	*Thaumasiovibrio*	*Thaumasiovibrio subtropicus*
Pseudomonadota	Xanthomonadaceae	*Thermomonas*	*Thermomonas carbonis*
Pseudomonadota	Thioalkalivibrionaceae	*Thioalkalivibrio*	*Thioalkalivibrio nitratireducens*
Pseudomonadota	Thioalkalivibrionaceae	*Thioalkalivibrio*	*Thioalkalivibrio sulfidiphilus*
Pseudomonadota	Thiobacillaceae	*Thiobacillus*	*Thiobacillus thiophilus*
Pseudomonadota	Chromatiaceae	*Thiocapsa*	*Thiocapsa imhoffii*
Pseudomonadota	Thiohalomonadaceae	*Thiohalomonas*	*Thiohalomonas nitratireducens*
Pseudomonadota	Sedimenticolaceae	*Thiolapillus*	*Thiolapillus brandeum*
Pseudomonadota	Thiomicrospiraceae	*Thiomicrorhabdus*	*Thiomicrorhabdus psychrophila*
Pseudomonadota	Thiomicrospiraceae	*Thiomicrospira*	*Thiomicrospira sibirica*
Pseudomonadota	Chromatiaceae	*Thiorhodococcus*	*Thiorhodococcus mannitoliphagus*
Pseudomonadota	Ectothiorhodospiraceae	*Thiorhodospira*	*Thiorhodospira sibirica*
Pseudomonadota	Thiotrichaceae	*Thiothrix*	*Thiothrix defluvii*
Pseudomonadota	Enterobacteriaceae	*Thorsellia*	*Thorsellia anophelis*
Pseudomonadota	Phyllobacteriaceae	*Tianweitania*	*Tianweitania sediminis*
Pseudomonadota	Aeromonadaceae	*Tolumonas*	*Tolumonas auensis*

Pseudomonadota	Aeromonadaceae	*Tolumonas*	*Tolumonas osonensis*
Pseudomonadota	Enterobacteriaceae	*Trabulsiella*	*Trabulsiella guamensis*
Pseudomonadota	Enterobacteriaceae	*Trabulsiella*	*Trabulsiella odontotermitis*
Pseudomonadota	Rhodocyclaceae	*Uliginosibacterium*	*Uliginosibacterium sediminicola*
Pseudomonadota	Burkholderiaceae	*Undibacterium*	*Undibacterium terreum*
Pseudomonadota	Pasteurellaceae	*Ursidibacter*	*Ursidibacter maritimus*
Pseudomonadota	Burkholderiaceae	*Variovorax*	*Variovorax boronicumulans*
Pseudomonadota	Burkholderiaceae	*Variovorax*	*Variovorax defluvii*
Pseudomonadota	Burkholderiaceae	*Variovorax*	*Variovorax ginsengisoli*
Pseudomonadota	Burkholderiaceae	*Variovorax*	*Variovorax humicola*
Pseudomonadota	Burkholderiaceae	*Variovorax*	*Variovorax soli*
Pseudomonadota	Pasteurellaceae	*Vespertiliibacter*	*Vespertiliibacter pulmonis*
Pseudomonadota	Vibrionaceae	*Vibrio*	*Vibrio aerogenes*
Pseudomonadota	Vibrionaceae	*Vibrio*	*Vibrio aestuarianus*
Pseudomonadota	Vibrionaceae	*Vibrio*	*Vibrio agarivorans*
Pseudomonadota	Vibrionaceae	*Vibrio*	*Vibrio alfacsensis*
Pseudomonadota	Vibrionaceae	*Vibrio*	*Vibrio alginolyticus*
Pseudomonadota	Vibrionaceae	*Vibrio*	*Vibrio algivorus*
Pseudomonadota	Vibrionaceae	*Vibrio*	*Vibrio anguillarum*
Pseudomonadota	Vibrionaceae	*Vibrio*	*Vibrio aphrogenes*
Pseudomonadota	Vibrionaceae	*Vibrio*	*Vibrio astriarenae*
Pseudomonadota	Vibrionaceae	*Vibrio*	*Vibrio atlanticus*
Pseudomonadota	Vibrionaceae	*Vibrio*	*Vibrio atypicus*
Pseudomonadota	Vibrionaceae	*Vibrio*	*Vibrio azureus*
Pseudomonadota	Vibrionaceae	*Vibrio*	*Vibrio bivalvicida*
Pseudomonadota	Vibrionaceae	*Vibrio*	*Vibrio brasiliensis*
Pseudomonadota	Vibrionaceae	*Vibrio*	*Vibrio breoganii*
Pseudomonadota	Vibrionaceae	*Vibrio*	*Vibrio campbellii*

Pseudomonadota	Vibrionaceae	*Vibrio*	*Vibrio caribbeanicus*
Pseudomonadota	Vibrionaceae	*Vibrio*	*Vibrio celticus*
Pseudomonadota	Vibrionaceae	*Vibrio*	*Vibrio cholerae*
Pseudomonadota	Vibrionaceae	*Vibrio*	*Vibrio cincinnatiensis*
Pseudomonadota	Vibrionaceae	*Vibrio*	*Vibrio comitans*
Pseudomonadota	Vibrionaceae	*Vibrio*	*Vibrio coralliilyticus*
Pseudomonadota	Vibrionaceae	*Vibrio*	*Vibrio crassostreae*
Pseudomonadota	Vibrionaceae	*Vibrio*	*Vibrio crosai*
Pseudomonadota	Vibrionaceae	*Vibrio*	*Vibrio diazotrophicus*
Pseudomonadota	Vibrionaceae	*Vibrio*	*Vibrio europaeus*
Pseudomonadota	Vibrionaceae	*Vibrio*	*Vibrio ezurae*
Pseudomonadota	Vibrionaceae	*Vibrio*	*Vibrio fluvialis*
Pseudomonadota	Vibrionaceae	*Vibrio*	*Vibrio fortis*
Pseudomonadota	Vibrionaceae	*Vibrio*	*Vibrio furnissii*
Pseudomonadota	Vibrionaceae	*Vibrio*	*Vibrio gallaecicus*
Pseudomonadota	Vibrionaceae	*Vibrio*	*Vibrio gallicus*
Pseudomonadota	Vibrionaceae	*Vibrio*	*Vibrio gazogenes*
Pseudomonadota	Vibrionaceae	*Vibrio*	*Vibrio gigantis*
Pseudomonadota	Vibrionaceae	*Vibrio*	*Vibrio halioticoli*
Pseudomonadota	Vibrionaceae	*Vibrio*	*Vibrio hangzhouensis*
Pseudomonadota	Vibrionaceae	*Vibrio*	*Vibrio harveyi*
Pseudomonadota	Vibrionaceae	*Vibrio*	*Vibrio hepatarius*
Pseudomonadota	Vibrionaceae	*Vibrio*	*Vibrio hippocampi*
Pseudomonadota	Vibrionaceae	*Vibrio*	*Vibrio hispanicus*
Pseudomonadota	Vibrionaceae	*Vibrio*	*Vibrio injenensis*
Pseudomonadota	Vibrionaceae	*Vibrio*	*Vibrio inusitatus*
Pseudomonadota	Vibrionaceae	*Vibrio*	*Vibrio ishigakensis*
Pseudomonadota	Vibrionaceae	*Vibrio*	*Vibrio japonicus*

Pseudomonadota	Vibrionaceae	*Vibrio*	*Vibrio jasicida*
Pseudomonadota	Vibrionaceae	*Vibrio*	*Vibrio kanaloae*
Pseudomonadota	Vibrionaceae	*Vibrio*	*Vibrio lentus*
Pseudomonadota	Vibrionaceae	*Vibrio*	*Vibrio litoralis*
Pseudomonadota	Vibrionaceae	*Vibrio*	*Vibrio mangrovi*
Pseudomonadota	Vibrionaceae	*Vibrio*	*Vibrio maritimus*
Pseudomonadota	Vibrionaceae	*Vibrio*	*Vibrio mediterranei*
Pseudomonadota	Vibrionaceae	*Vibrio*	*Vibrio metoecus*
Pseudomonadota	Vibrionaceae	*Vibrio*	*Vibrio metschnikovii*
Pseudomonadota	Vibrionaceae	*Vibrio*	*Vibrio mimicus*
Pseudomonadota	Vibrionaceae	*Vibrio*	*Vibrio mytili*
Pseudomonadota	Vibrionaceae	*Vibrio*	*Vibrio natriegens*
Pseudomonadota	Vibrionaceae	*Vibrio*	*Vibrio neocaledonicus*
Pseudomonadota	Vibrionaceae	*Vibrio*	*Vibrio neonatus*
Pseudomonadota	Vibrionaceae	*Vibrio*	*Vibrio nereis*
Pseudomonadota	Vibrionaceae	*Vibrio*	*Vibrio nigripulchritudo*
Pseudomonadota	Vibrionaceae	*Vibrio*	*Vibrio olivae*
Pseudomonadota	Vibrionaceae	*Vibrio*	*Vibrio orientalis*
Pseudomonadota	Vibrionaceae	*Vibrio*	*Vibrio ostreicida*
Pseudomonadota	Vibrionaceae	*Vibrio*	*Vibrio panuliri*
Pseudomonadota	Vibrionaceae	*Vibrio*	*Vibrio parahaemolyticus*
Pseudomonadota	Vibrionaceae	*Vibrio*	*Vibrio pelagius*
Pseudomonadota	Vibrionaceae	*Vibrio*	*Vibrio penaeicida*
Pseudomonadota	Vibrionaceae	*Vibrio*	*Vibrio ponticus*
Pseudomonadota	Vibrionaceae	*Vibrio*	*Vibrio porteresiae*
Pseudomonadota	Vibrionaceae	*Vibrio*	*Vibrio proteolyticus*
Pseudomonadota	Vibrionaceae	*Vibrio*	*Vibrio quintilis*
Pseudomonadota	Vibrionaceae	*Vibrio*	*Vibrio rarus*

Pseudomonadota	Vibrionaceae	*Vibrio*	*Vibrio rotiferianus*
Pseudomonadota	Vibrionaceae	*Vibrio*	*Vibrio ruber*
Pseudomonadota	Vibrionaceae	*Vibrio*	*Vibrio rumoiensis*
Pseudomonadota	Vibrionaceae	*Vibrio*	*Vibrio salilacus*
Pseudomonadota	Vibrionaceae	*Vibrio*	*Vibrio scophthalmi*
Pseudomonadota	Vibrionaceae	*Vibrio*	*Vibrio spartinae*
Pseudomonadota	Vibrionaceae	*Vibrio*	*Vibrio splendidus*
Pseudomonadota	Vibrionaceae	*Vibrio*	*Vibrio stylophorae*
Pseudomonadota	Vibrionaceae	*Vibrio*	*Vibrio superstes*
Pseudomonadota	Vibrionaceae	*Vibrio*	*Vibrio tapetis*
Pseudomonadota	Vibrionaceae	*Vibrio*	*Vibrio thalassae*
Pseudomonadota	Vibrionaceae	*Vibrio*	*Vibrio toranzoniae*
Pseudomonadota	Vibrionaceae	*Vibrio*	*Vibrio tritonius*
Pseudomonadota	Vibrionaceae	*Vibrio*	*Vibrio tubiashii*
Pseudomonadota	Vibrionaceae	*Vibrio*	*Vibrio vulnificus*
Pseudomonadota	Vibrionaceae	*Vibrio*	*Vibrio xiamenensis*
Pseudomonadota	Vibrionaceae	*Vibrio*	*Vibrio xuii*
Pseudomonadota	Vibrionaceae	*Vibrio*	*Vibrio zhugei*
Pseudomonadota	Vibrionaceae	*Vibrio*	*Vibrio zhuhaiensis*
Pseudomonadota	Chromobacteriaceae	*Vogesella*	*Vogesella amnigena*
Pseudomonadota	Wenzhouxiangellaceae	*Wenzhouxiangella*	*Wenzhouxiangella salilacus*
Pseudomonadota	Wohlfahrtiimonadaceae	*Wohlfahrtiimonas*	*Wohlfahrtiimonas larvae*
Pseudomonadota	Xanthobacteraceae	*Xanthobacter*	*Xanthobacter agilis*
Pseudomonadota	Xanthomonadaceae	*Xanthomonas*	*Xanthomonas alfalfae*
Pseudomonadota	Xanthomonadaceae	*Xanthomonas*	*Xanthomonas campestris*
Pseudomonadota	Xanthomonadaceae	*Xanthomonas*	*Xanthomonas citri*
Pseudomonadota	Enterobacteriaceae	*Xenorhabdus*	*Xenorhabdus beddingii*
Pseudomonadota	Enterobacteriaceae	*Xenorhabdus*	*Xenorhabdus bovienii*

Pseudomonadota	Enterobacteriaceae	*Xenorhabdus*	*Xenorhabdus cabanillasii*
Pseudomonadota	Enterobacteriaceae	*Xenorhabdus*	*Xenorhabdus doucetiae*
Pseudomonadota	Enterobacteriaceae	*Xenorhabdus*	*Xenorhabdus eapokensis*
Pseudomonadota	Enterobacteriaceae	*Xenorhabdus*	*Xenorhabdus ehlersii*
Pseudomonadota	Enterobacteriaceae	*Xenorhabdus*	*Xenorhabdus griffiniae*
Pseudomonadota	Enterobacteriaceae	*Xenorhabdus*	*Xenorhabdus hominickii*
Pseudomonadota	Enterobacteriaceae	*Xenorhabdus*	*Xenorhabdus indica*
Pseudomonadota	Enterobacteriaceae	*Xenorhabdus*	*Xenorhabdus innexi*
Pseudomonadota	Enterobacteriaceae	*Xenorhabdus*	*Xenorhabdus ishibashii*
Pseudomonadota	Enterobacteriaceae	*Xenorhabdus*	*Xenorhabdus japonica*
Pseudomonadota	Enterobacteriaceae	*Xenorhabdus*	*Xenorhabdus khoisanae*
Pseudomonadota	Enterobacteriaceae	*Xenorhabdus*	*Xenorhabdus koppenhoeferi*
Pseudomonadota	Enterobacteriaceae	*Xenorhabdus*	*Xenorhabdus kozodoii*
Pseudomonadota	Enterobacteriaceae	*Xenorhabdus*	*Xenorhabdus magdalenensis*
Pseudomonadota	Enterobacteriaceae	*Xenorhabdus*	*Xenorhabdus mauleonii*
Pseudomonadota	Enterobacteriaceae	*Xenorhabdus*	*Xenorhabdus miraniensis*
Pseudomonadota	Enterobacteriaceae	*Xenorhabdus*	*Xenorhabdus nematophila*
Pseudomonadota	Enterobacteriaceae	*Xenorhabdus*	*Xenorhabdus poinarii*
Pseudomonadota	Enterobacteriaceae	*Xenorhabdus*	*Xenorhabdus romanii*
Pseudomonadota	Enterobacteriaceae	*Xenorhabdus*	*Xenorhabdus stockiae*
Pseudomonadota	Enterobacteriaceae	*Xenorhabdus*	*Xenorhabdus szentirmaii*
Pseudomonadota	Enterobacteriaceae	*Xenorhabdus*	*Xenorhabdus thuongxuanensis*
Pseudomonadota	Enterobacteriaceae	*Xenorhabdus*	*Xenorhabdus vietnamensis*
Pseudomonadota	Rhodobacteraceae	*Xinfangfangia*	*Xinfangfangia humi*
Pseudomonadota	Enterobacteriaceae	*Yersinia*	*Yersinia aldovae*
Pseudomonadota	Enterobacteriaceae	*Yersinia*	*Yersinia aleksiciae*
Pseudomonadota	Enterobacteriaceae	*Yersinia*	*Yersinia bercovieri*
Pseudomonadota	Enterobacteriaceae	*Yersinia*	*Yersinia enterocolitica*

Pseudomonadota	Enterobacteriaceae	*Yersinia*	*Yersinia entomophaga*
Pseudomonadota	Enterobacteriaceae	*Yersinia*	*Yersinia frederiksenii*
Pseudomonadota	Enterobacteriaceae	*Yersinia*	*Yersinia intermedia*
Pseudomonadota	Enterobacteriaceae	*Yersinia*	*Yersinia kristensenii*
Pseudomonadota	Enterobacteriaceae	*Yersinia*	*Yersinia massiliensis*
Pseudomonadota	Enterobacteriaceae	*Yersinia*	*Yersinia mollaretii*
Pseudomonadota	Enterobacteriaceae	*Yersinia*	*Yersinia nurmii*
Pseudomonadota	Enterobacteriaceae	*Yersinia*	*Yersinia pestis*
Pseudomonadota	Enterobacteriaceae	*Yersinia*	*Yersinia pseudotuberculosis*
Pseudomonadota	Enterobacteriaceae	*Yersinia*	*Yersinia rohdei*
Pseudomonadota	Enterobacteriaceae	*Yersinia*	*Yersinia ruckeri*
Pseudomonadota	Enterobacteriaceae	*Yersinia*	*Yersinia similis*
Pseudomonadota	Enterobacteriaceae	*Yersinia*	*Yersinia wautersii*
Pseudomonadota	Enterobacteriaceae	*Yokenella*	*Yokenella regensburgei*
Pseudomonadota	Burkholderiaceae	*Zhizhongheella*	*Zhizhongheella caldifontis*
Pseudomonadota	Aeromonadaceae	*Zobellella*	*Zobellella aerophila*
Pseudomonadota	Aeromonadaceae	*Zobellella*	*Zobellella denitrificans*
Pseudomonadota	Aeromonadaceae	*Zobellella*	*Zobellella taiwanensis*
Pseudomonadota	Rhodocyclaceae	*Zoogloea*	*Zoogloea oryzae*
Pseudomonadota	Zooshikellaceae	*Zooshikella*	*Zooshikella ganghwensis*
Spirochaetota	Alkalispirochaetaceae	*Alkalispirochaeta*	*Alkalispirochaeta alkalica*
Spirochaetota	Alkalispirochaetaceae	*Alkalispirochaeta*	*Alkalispirochaeta sphaeroplastigenens*
Spirochaetota	Breznakiellaceae	*Breznakiella*	*Breznakiella homolactica*
Spirochaetota	Spirochaetaceae	*Spirochaeta*	*Spirochaeta halophila*
Spirochaetota	Spirochaetaceae	*Spirochaeta*	*Spirochaeta isovalerica*
Spirochaetota	Treponemataceae	*Treponema*	*Treponema denticola*
Spirochaetota	Treponemataceae	*Treponema*	*Treponema putidum*
Synergistota	Acetomicrobiaceae	*Acetomicrobium*	*Acetomicrobium mobile*

Synergistota	Aminobacteriaceae	*Aminobacterium*	*Aminobacterium mobile*
Synergistota	Synergistaceae	*Cloacibacillus*	*Cloacibacillus porcorum*
Synergistota	Aminobacteriaceae	*Fretibacterium*	*Fretibacterium fastidiosum*
Synergistota	Synergistaceae	*Thermanaerovibrio*	*Thermanaerovibrio acidaminovorans*
Synergistota	Synergistaceae	*Thermanaerovibrio*	*Thermanaerovibrio velox*
Terrabacteria	Synechococcales	*Cymatolege*	*Cymatolege isodiametrica*
Terrabacteria	Entomoplasmataceae	*Mesoplasma*	*Mesoplasma syrphidae*
Terrabacteria	Synechococcales	*Metis*	*Metis fasciculata*
Terrabacteria	Oculatellaceae	*Oculatella*	*Oculatella cataractarum*
Terrabacteria	Oculatellaceae	*Timaviella*	*Timaviella obliquedivisa*
Terrabacteria	Trichocoleusaceae	*Trichocoleus*	*Trichocoleus caatingensis*
Terrabacteria	Trichocoleusaceae	*Trichocoleus*	*Trichocoleus desertorum*
Thermodesulfobiota	Thermodesulfobiaceae	*Geothermobacterium*	*Geothermobacterium ferrireducens*
Thermotogota	Fervidobacteriaceae	*Thermosipho*	*Thermosipho melanesiensis*
Verrucomicrobiota	Akkermansiaceae	*Akkermansia*	*Akkermansia glycaniphila*
Verrucomicrobiota	Akkermansiaceae	*Akkermansia*	*Akkermansia muciniphila*
Verrucomicrobiota	Victivallaceae	*Victivallis*	*Victivallis vadensis*
